# Adipose stem cells derived extracellular vesicles alleviate retinal excitotoxicity via miR-23a-5p/PLCD1/PKCA/GluA2 axis: a potential therapeutic strategy

**DOI:** 10.1186/s13287-026-05140-z

**Published:** 2026-06-30

**Authors:** Duan Tianqi, Xu Huizhuo, Tang Shibo, Yu Haiyang, Luo Aixiang, Li Ming, Huang Jufang

**Affiliations:** 1 Guangzhou Aier Eye Hospital, Jinan University, Guangzhou, 510010 Guangdong China; 2 Changsha Aier Eye Hospital, Aier Eye Institute, Changsha, 410004 Hunan China; 3 Eye Center of Xiangya Hospital, Central South University, Changsha, 410008 Hunan China; 4 National Clinical Research Center for Geriatric Disorders, Xiangya Hospital, Central South University, Changsha, 410008 Hunan China; 5 Department of Anatomy and Neurobiology, Xiangya School of Basic Medical Sciences, Central South University, No.172 Tongzipo Road, Changsha, 410013 Hunan China

**Keywords:** Adipose stem cell-derived extracellular vesicles (ADSC-EVs), Excitotoxicity, miR-23a-5p, Phospholipase C delta 1 (PLCD1), PKCA pathway, AMPARs subunit 2 (GluA2)

## Abstract

**Background:**

Despite excitotoxicity being a pivotal pathological mechanism in various retinal diseases, effective clinical interventions remain limited. Previous study has shown that adipose stem cell-derived extracellular vesicles (ADSC-EVs) can alleviate glutamate-induced retinal ganglion cells (RGCs) death by suppressing protein kinase C alpha (PKCA) pathway and increasing the expression of α-amino-3-hydroxy-5-methyl-4-isoxazoleproprionic acid receptors (AMPARs) subunit 2 (GluA2) on the cell membrane, but the mechanisms remain unexplored.

**Methods:**

To clarify the molecular processes involved in ADSC-EVs-mediated intracellular calcium balance, we isolated ADSC-EVs using ultrafiltration and ultracentrifugation, and characterized these vesicles by transmission electron microscopy, nanoparticle tracking analysis, and flow cytometry. Small RNA sequencing was performed on glutamate-injured rat retinal precursor (R28) cells pre-treated with ADSC-EVs or PBS. Through bioinformatic analysis, we identified candidate microRNAs and predicted their potential target genes. The regulatory effects of microRNA were confirmed using propidium iodide staining, Fluo-4AM staining, western blotting, and immunofluorescence. Additionally, the RGCs counting and visual function tests were employed to evaluate the therapeutic efficacy of the microRNA in the glutamate-induced SD rat - animal model.

**Results:**

Our results demonstrated that pre-treatment with ADSC-EVs led to a significant increase in the expression levels of miR-127-3p, miR-181b-1-3p, miR-199a-3p/5p, miR-23a-5p, miR-23b-5p, and miR-486 in R28 cells. Bioinformatic analyses suggest that miR-23a-5p may contribute to regulating the calcium overload by inhibiting the expression of phospholipase C delta 1 (PLCD1). Overexpression of miR-23a-5p or pre-treatment with ADSC-EVs modulated the expression of GluA2 on the cell membrane through inhibiting the PLCD1/PKCA/GluA2 axis, thereby reducing intracellular calcium levels and diminishing R28 cell death. In contrast, inhibition of miR-23a-5p expression partially reversed the regulatory effects of ADSC-EVs on calcium concentration and cell viability. Furthermore, our findings suggest that overexpression of miR-23a-5p in retina alleviated glutamate-induced RGCs death and visual function impairment, whereas suppression of miR-23a-5p exacerbated glutamate-induced RGCs death.

**Conclusion:**

ADSC-EVs delived miR-23a-5p mitigate glutamate-induced RGCs damage by inhibiting the PLCD1/PKCA/GluA2 axis. Targeting this miR-23a-5p-mediated axis may therefore represent a promising therapeutic approach for excitotoxic RGCs injury.

**Graphical Abstract:**

**Figure:**
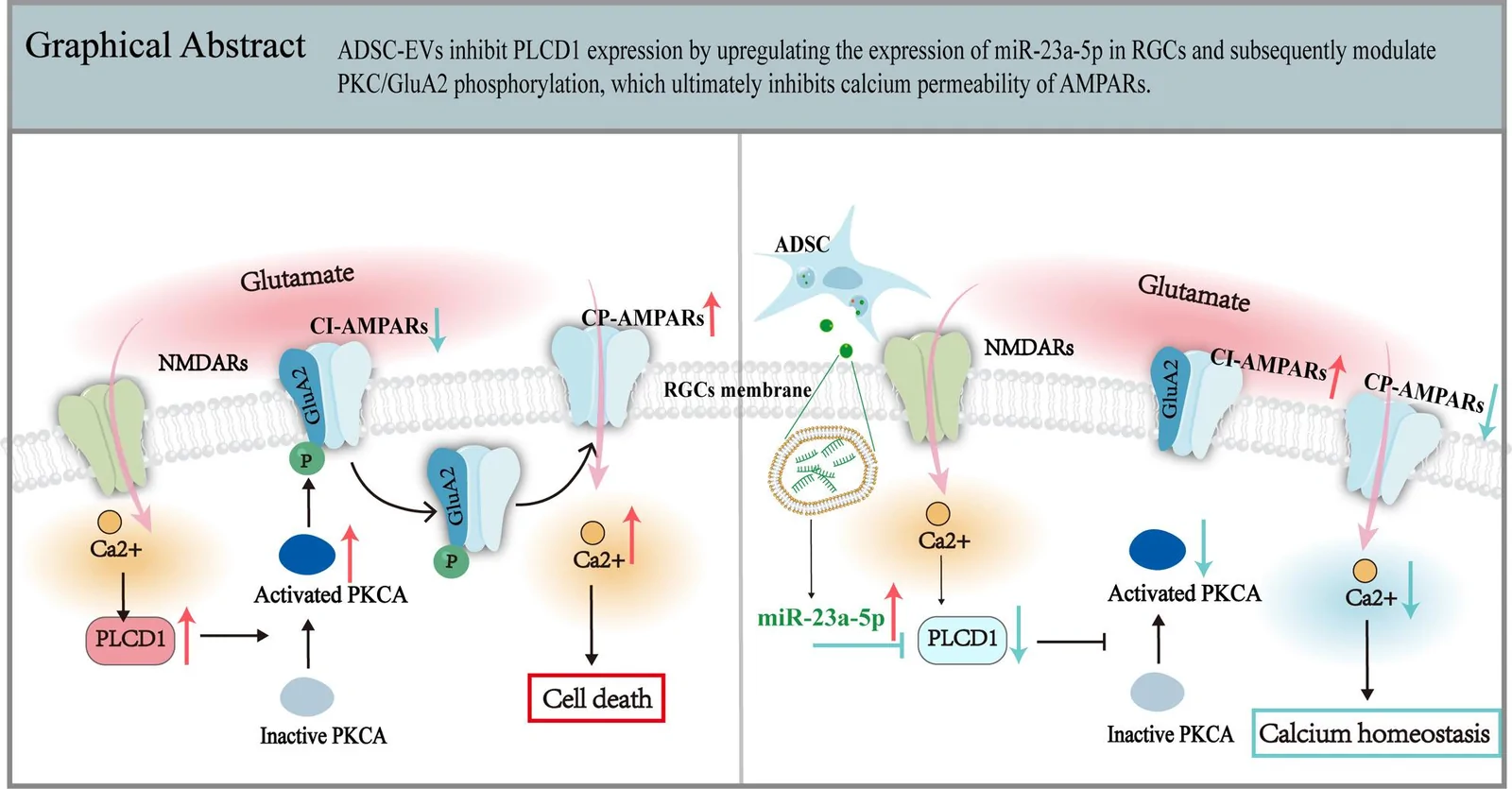

**Supplementary Information:**

The online version contains supplementary material available at https://doi.org/10.1186/s13287-026-05140-z.

## Background

Excitotoxicity is a fundamental pathological process in various retinal diseases, such as normal-tension glaucoma [1], diabetic retinopathy [2], and retinal vein occlusion [3]. Ion-associated glutamate receptors antagonists, are primarily used in clinical settings to reduce excitotoxic effects associated with neurodegenerative diseases [4]. However, the systemic application of these antagonists may result in side effects that impact both the nervous and digestive systems [5]. Therefore, mitigating excitotoxicity in retinal diseases remains an essential objective to be accomplished in clinical treatment.

In retinal diseases, pathological mechanisms such as ischemia, hypoxia, and inflammatory responses often coincide with the disruption of glutamate metabolism [6–8]. The excessive accumulation of glutamate subsequently increases the calcium permeability of ion-associated glutamate receptors on the RGCs membrane. Modulating the ion-associated glutamate receptors is a crucial method for lessening excitotoxicity [9]. Among all glutamate receptors, AMPAR is typically impermeable to calcium ion under physiological conditions [10, 11]. However, in pathological states, the GluA2 subunit of AMPAR is internalized into the intracellular compartment. The increase of GluA2-lacking AMPAR leads to calcium overload [12–14]. Hence, reducing the expression of GluA2-lacking AMPAR may be an efficient method in mitigating glutamate-induced excitotoxicity.

In recent years, mesenchymal stem cell-derived extracellular vesicles (MSC-EVs) have attracted considerable attention for their potential in treating retinal diseases [15, 16]. MSC-EVs preserve the therapeutic advantages of MSCs while circumventing risks such as immunogenicity, tumorigenicity, and teratoma formation [17, 18]. Adipose mesenchymal stem cells (ADSCs) have demonstrated remarkable anti-excitotoxic effects [19–21]. Our previous study confirmed that ADSC-EVs can mitigate glutamate-induced calcium overload in RGCs through the inhibition of PKCA/GluA2 phosphorylation, which perform the neuroprotective effect [22]. Nevertheless, the instability of ADSC-EVs treatment effects requires further exploration of the key regulators and primary regulatory mechanisms involved. Previous works have suggested that MSC-EVs participate in intercellular communication and regulate target gene expression by regulating functional microRNA levels [23, 24]. However, the key microRNAs involved in attenuating glutamate-induced intracellular calcium overload are still unclear.

Here, we identify the critical mechanisms of ADSC-EVs that modulate glutamate-induced excitotoxicity, offering insights into potential therapeutic strategies for alleviating excitotoxicity when treating retinal diseases.

## Methods

### Animals

The 6–8 weeks old male Sprague-Dawley (SD) rats used for in vivo experiments were purchased from Hunan Slaughter Kingda Laboratory Animal Co., Ltd. All rats were kept in a specific-pathogen-free environment with appropriate temperature and humidity and had free access to drinking water and rat chow. For terminal procedures, rats received zoletil/xylazine anesthesia (1.5%/0.2%, 1 µL/g i.p.) followed by CO₂ euthanasia, with their death confirmed by cessation of respiration, reflexes, and cardiac activity, according to ethical guidelines. All animal experiments were recorded following the guidelines set by the Animal Research: Reporting of In Vivo Experiments (ARRIVE) [25].

### Isolation and characterization of ADSCs

In our previous studies, ADCSs were collected and isolated from human adipose tissue. These tissues were collected from a cohort of multiple donors (“Xiangya Sample Bank project of Central South University”, Approved number: 202111001), and after being passaged to the third generation, they were preserved in liquid nitrogen. ADSCs were characterized following established protocols from our previous studies [22]. Initial morphological assessment was performed using light microscopy, and flow cytometry was used to assess the biomarkers of ADSCs (Changsha Golden Medical Clinical Laboratory Center). To quantify differentiation capacity, ADSCs were plated on 1% gelatin-coated 6-well plates and induced to differentiate into adipogenic and osteogenic lineages using specialized differentiation media (Cyagen Biosciences; HUXMD-90031, HUXMD-90021) at 70% confluency. After 4 weeks of induction, successful differentiation was confirmed through histological staining with Oil Red O (adipogenesis) and Alizarin Red (osteogenesis), followed by microscopic evaluation of phenotypic changes.

### Cell culture

The rat retinal precursor (R28) cell line was received as a gift from the Xiong Lab (Department of Human Anatomy and Neurobiology, Central South University). Both the R28 cell line and the ADSCs were maintained in a sterile, humidified incubator at 37 °C with 5% CO₂. Upon reaching 80–100% confluency, they were passaged using 0.25% trypsin (Gibco, USA) and cultured in low-glucose Dulbecco’s Modified Eagle’s Medium (DMEM; Hyclone, SH30021.01) that were supplemented with 10% fetal bovine serum (ExCell Bio, China).

### Extracellular vesicles isolation and characterization

We first collected the supernatant of ADSCs after 48 h culture with Excel 10% exosome-free FBS. The supernatant was sequentially processed by centrifugation (200 × g, 10 mins, 4 °C) to remove debris, followed by 0.45-µm filtration and 100-kDa ultrafiltration (Millipore UFC9100), and finally subjected to ultra-high-speed centrifugation at 100,000 × g for 70 mins. Purified EVs were obtained by resuspending the pellet in PBS. To characterize the isolated EVs, we employed nanoparticle tracking analysis to determine particle concentration and size distribution profiles, transmission electron microscopy to evaluate ultrastructural morphology, and flow cytometry to quantify the EVs surface markers (Fortune Flow Biotechnology Co.). This comprehensive characterization strategy enabled systematic quality control of EVs preparations, verified their physical properties, structural integrity, and molecular signatures. Thus, batch-to-batch consistency is assured for subsequent experimental applications.

### Internalization assay

According to the guidelines of the PKH26 Red Fluorescent Cell Ligation Kit (Umibio, Shanghai, China; UR52302), EVs were labeled. Briefly, EVs were immersed in a 10 µL solution of diluted PKH26 (comprising 1 µL PKH26 and 9 µL PBS) for a duration of 10 mins. Subsequently, the mixture was rinsed with 10 mL of 1× PBS and then concentrated using a 100-kDa ultrafiltration tube (3000 × g, 20 mins, 4 °C). The pellets were resuspended (10 µL) and added to R28 cells (a 6-well plate, 2 mL medium) at 25 µg/mL following our previous study [22]. After an 8-hour incubation period with the PKH26-labeled EVs, the cells were stained with 4′,6-diamidino- 2-phenylindole (DAPI, Solarbio, Beijing, China) and observed under a fluorescence microscope (Zeiss, Jena, Germany, AXIO Vert.A1).

### Cell interference, glutamate-injured model, and groups in vitro

**Fig. 1:**
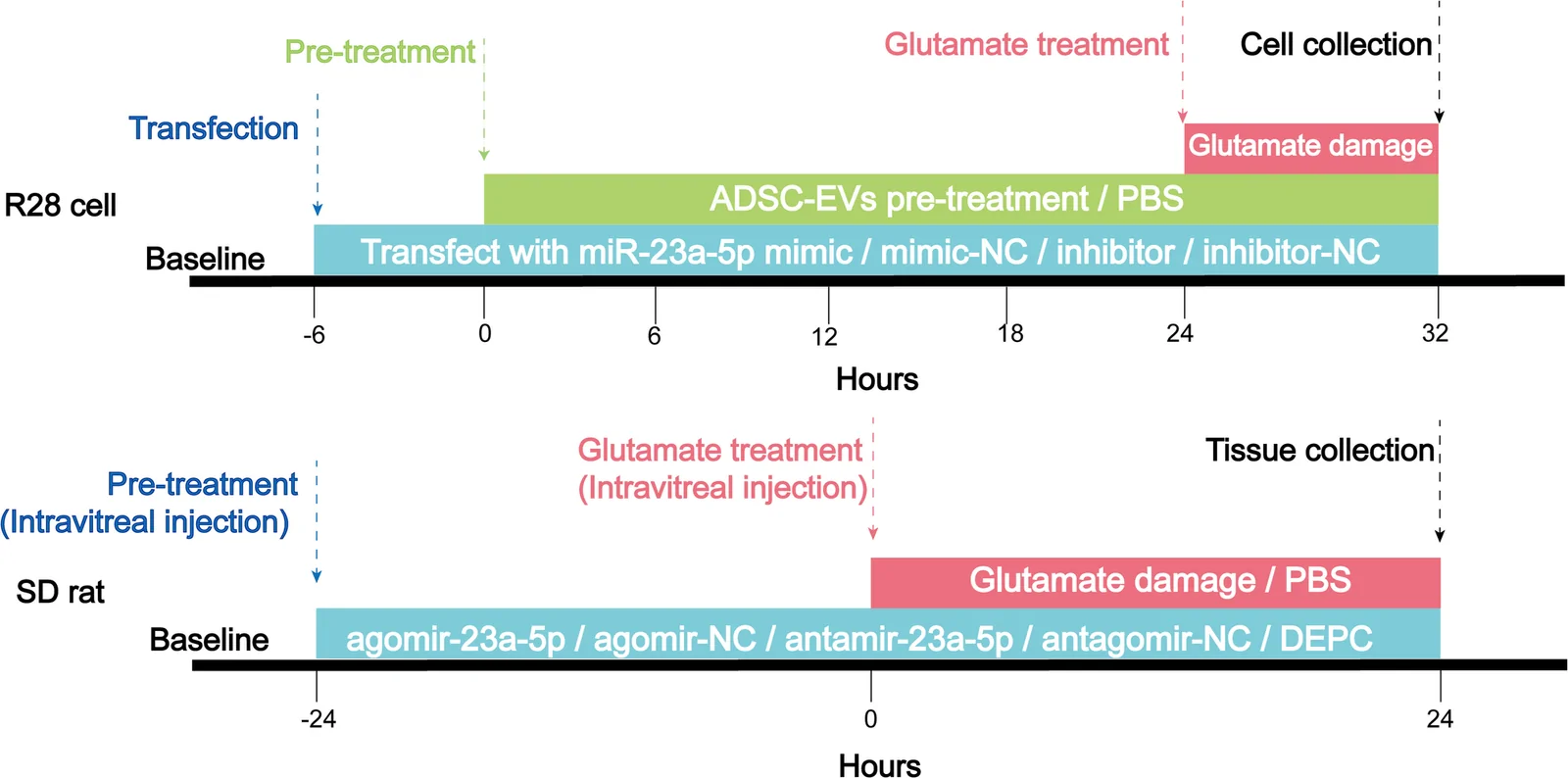
Schematic of the experimental timeline of in vitro and in vivo modeling. In the in vitro model (top panel), and in the in vivo model (bottom panel). ADSC-EVs: Adipose mesenchymal stem cell-extracellular vesicles; PBS: phosphate buffered solution; R28: rat retinal precursor cells; DEPC: diethy pyrocarbonate. SD: Sprague-Dawley

The glutamate-induced injury model in R28 cells was established by treating the cells with 34 mM glutamate for 8 h, following previously established protocols [22]. Prior to injury induction, R28 cells were transfected respectively with 30 nM miR-23a-5p mimic, mimic negative control (NC), inhibitor, or inhibitor NC using Lipofectamine 2000 (Supplementary Fig. 1E and F; Thermo Fisher Scientific, USA, L3000008), with some groups additionally pretreated with ADSC-EVs for 24 h (Fig. 1). The experiment comprised seven groups: a control group (Con), a glutamate-injured group (Glu), groups transfected with miR-23a-5p mimic (Glu + mimic) or mimic NC (Glu + mimic NC) before glutamate exposure, an ADSC-EVs pretreated group (Glu + EVs), and groups receiving both ADSC-EVs pretreatment combined with either miR-23a-5p inhibitor (Glu + EVs + inhibitor) or inhibitor NC (Glu + EVs + inhibitor NC) transfection prior to glutamate damage. The sequences of miR-23a-5p mimics and inhibitors used in in vitro experiments are provided in Table 1.

**Table 1:** MicroRNA sequence

Sequence name	Sequence (5′→3′)	
miR-23a-5p mimic	Sense	GGGGUUCCUGGGGAUGGGAUUU
	Antisense	AUCCCAUCCCCAGGAACCCCUU
mimic-NC	Sense	UUGUACUACACAAAAGUACUG
	Antisense	GUACUUUUGUGUAGUACAAUU
miR-23a-5 inhibitor	Sense	AAAUCCCAUCCCCAGGAACCCC
inhibitor NC	Sense	CAGUACUUUUGUGUAGUACAA

### Small RNA sequencing and data analysis

Total RNA was extracted from the Glu + PBS (n = 3) and Glu + EVs (n = 3) groups of R28 cells using the mirVana miRNA Isolation Kit (Ambion, USA). RNA quantification was performed using a Nanodrop 2000 spectrophotometer (Thermo Fisher Scientific, USA), followed by quality assessment using an Agilent 2100 Bioanalyzer (Agilent Technologies, USA). For small RNA library preparation, 1 µg of total RNA per sample was processed using the TruSeq Small RNA Sample Prep Kit (Illumina, USA, RS-200-0012). Subsequent small RNA sequencing and bioinformatics analysis were carried out by OE Biotech (Shanghai, China).

### Establishment of glutamate-injured model and groups in vivo

We established an animal model of glutamate-induced injury through intravitreal injection. After pupil dilation with compound tropicamide (Bausch & Lomb), we injected 3 µL of DEPC, agomir-23a-5p, agomir-NC, antagomir-23a-5p, or antagomir-NC into the vitreous cavity using a micro syringe (Hamilton) respectively, with the injection point located 1 mm behind the corneal limbus. One day later, 4 µL PBS or 100 mM glutamate was injected into the same vitreous cavity (Fig. 1). The animals were euthanized 24 h after the final intravitreal injection [26]. Animals with severe infection or traumatic cataract were excluded from data collection. No animals required exclusion in this experiment. Rats were randomly divided into six experimental groups using a computer-generated randomization schedule: a control group with DEPC and PBS treatment (Con), a DEPC and glutamate treatment group (Glu), an agomir-23a-5p and glutamate treatment group (Glu + agomir), an agomir-NC and glutamate treatment group (Glu + agomir-NC), an antagomir-23a-5p and glutamate treatment group (Glu + antagomir), and an antagomir-NC and glutamate treatment group (Glu + antagomir-NC). A total of 14 SD rats were assigned to each group for the evaluation of visual function (*n* = 6), quantification of RGCs in retinal whole-mount preparations (*n* = 5), and Western blot analysis (*n* = 3). Blinding was not employed in this study. However, to ensure accuracy in data recording, all primary outcome data were independently extracted and verified by two researchers prior to statistical analysis. The sequences of miR-23a-5p agomir and antagomir used in in vivo experiments are provided in Table 2.

**Table 2:** MicroRNA sequence

Sequence name	Sequence (5′→3′)	
miR-23a-5p agomir (cy3)	Sense	GGGGUUCCUGGGGAUGGAUUU
	Antisense	AUCCCAUCCCCAGGAACCCCUU
agomir NC	Sense	UUCUCCGAACGUGUCACG
	Antisense	ACGUGACACGUUCGGAGAATT
miR-23a-5p antagomir (cy3)	Sense	AAAUCCCAUCCCCAGGAACCCC
antagomir NC	Sense	CAGUACUUUUGUGUAGUACAA

### Dual-luciferase reporter assay

Bioinformatic analysis using RNAhybrid 2.2 and miRanda predicted potential binding sites between PLCD1 and miR-23a-5p. Synthetic miR-23a-5p mimics/inhibitors and negative controls (NC) were obtained from Beijing Kinko Biotechnology. Sequences containing the binding sites were subsequently synthesized, and wild-type and mutant sequences were constructed into a dual-luciferase reporter gene vector (pmirGLO) to obtain pmirGLO-PLCD1-wt and pmirGLO-PLCD1-mut. HEK293T cells, selected for their low endogenous miRNA expression, were co-transfected with 0.15 µg reporter constructs and 20 µM miR-23a-5p mimics/inhibitors using the Hieff Trans Liposomal Transfection Reagent (Yisheng Biotechnology). After 24 h of transfection, Firefly & Renilla luciferase activities were evaluated in the lysed HEK293T cells utilizing a dual-luciferase reporter detection system (Promega, USA).

### Quantitative real-time polymerase chain reaction (qRT-PCR)

We used qRT-PCR to measure miRNA levels. Relative quantification was calculated using the 2 − ΔΔCT method, with experiments conducted in triplicate. U6 served as the endogenous control for miR-23a-5p normalization. Three independent experimental units were used per group for statistical analysis. Detailed primer sequences are given in Table 3.

**Table 3:** Primer sequences for qRT-PCR

Targets	Primer sequences	
miR-23a-5p	Forward (5′→3′)	ATTAGTGGGGTTCCTGGGGAT
	Reverse (5′→3′)	Provided by Sangon Biotech (Shanghai) Co., Ltd.
U6	Forward (5′→3′)	Provided by Sangon Biotech (Shanghai) Co., Ltd.
	Reverse (5′→3′)	Provided by Sangon Biotech (Shanghai) Co., Ltd.

### Propidium iodide (PI) staining

PI staining serves as a reliable indicator of necrotic cell death [27]. The R28 cells were washed twice with PBS and then treated with PI solution (10 µg/mL; Sigma, USA, Cat# P4170) for 10 mins. Subsequently, the cells were fixed with 4% paraformaldehyde (PFA) for 15 mins and stained with DAPI for 5 mins. Three independent experimental units were used per group for statistical analysis. Finally, we used fluorescence microscopy to measure necrosis by calculating the percentage of PI-positive cells compared to those stained with DAPI.

### Intracellular calcium ion detection

Following three washes with calcium-free HBSS (Solarbio, China), the cells were incubated with a 4 µM Fluo-4 AM fluorescent probe (Solarbio, China) at 37 °C for 30 mins to facilitate calcium indicator loading. After fixation with 4% PFA and nuclear counterstaining with DAPI, intracellular calcium levels were evaluated using fluorescence microscopy. Quantitative analysis was conducted based on the established methodology [28], where the relative calcium ion concentration was determined by calculating the ratio of the Fluo-4 AM fluorescence signal area to the number of DAPI-positive R28 cells. Six independent experimental units were used per group for statistical analysis. All image analyses, including fluorescence area quantification and cell counting, were performed using ImageJ software (v.1.5.1; National Institutes of Health, Baltimore, MD, USA).

### Western blotting

Retinal tissues and cultured R28 cells were lysed using radioimmunoprecipitation assay (RIPA) buffer. The protein concentrations were measured using the BCA kit (Solarbio, China). The cell and tissue samples were electrophoresed on polyacrylamide gels and transferred to PVDF membranes. After blocking the membranes with 5% bovine serum albumin (BSA), they were incubated overnight at 4 °C with the following primary antibodies: GluA2 (1:1000, Abclonal, Wuhan, China, Cat# A0111; RRID: AB_2756954), p-GluA2 (1:500, Affinity Bioscience, Beijing, China, Cat# AF3307, RRID: AB_2834726), PKCA (1:1000, Wanleibio, Cat# WL02234, RRID: AB_2925211), p-PKCA (1:1000, Abcam, Cambridge, UK, Cat# ab32502, RRID: AB_777295), Cleaved-C3 (1:2000, Cell Signaling Technology, Danvers, MA, USA, Cat# 9661, RRID: AB_2341188), PLCD1 (1:1500, Huabio, Hangzhou, China, Cat# ER60337), or beta-tubulin (1: 2000, Proteintech, Wuhan, China, Cat# 66240-1-Ig, RRID: AB_2881629). The following day, the membrane was washed with PBS-T (Tween 20), and subsequently incubated with an HRP-conjugated goat anti-rabbit secondary antibody (1:10,000, Abbkine, Wuhan, China, Cat# A25222, RRID: AB_2922982) for 2 h at room temperature. Protein expression was visualized via an ECL kit, and band intensity was quantitatively analyzed using ImageJ software. Every experiment of these proteins was independently repeated in three independent experimental units.

### Immunofluorescence staining

After fixation with 4% PFA for 20 mins, R28 cells were washed three times with 0.01 M PBS. To block nonspecific binding sites, 5% BSA was applied for 1 h. Immunofluorescence staining was performed by sequentially incubating the cells with a rabbit anti-GluA2 primary antibody (1:50 dilution; ABclonal A0111) at 4 °C overnight and an Alexa Fluor 594-conjugated goat anti-rabbit IgG secondary antibody (1:200; Abcam ab150160) for 2 h at room temperature. The nuclei were counterstained with DAPI for 5 mins before imaging. Each experiment was independently repeated three times. Fluorescence signals were captured using laser scanning confocal microscopy and quantified using ImageJ software.

### Retinal flat-mounted immunofluorescence

For retinal whole-mount immunofluorescence analysis, enucleated rat eyes were fixed in 4% PFA for 2 h at room temperature. After fixation, the cornea and lens were carefully removed, and the retina was dissected and flattened onto glass slides before being radially sectioned into four quadrants. The retinas were permeabilized in PBS-T (0.3% Triton X-100, v/v) for 30 mins, then blocked in a PBST-diluted blocking solution (5% BSA, m/v) for 1.5 h. Following this retinal samples were incubated with anti-RBPMS (1:500; Abcam; Cat# ab152101; RRID: AB_2923082) for at least 24 h at 4℃. After three washes in PBST, the retinas were stained with an Alexa Fluor 488-conjugated goat anti-rabbit secondary antibody for 2 h at room temperature and then mounted. Images were captured using a fluorescence microscope (Zeiss, Jena, Germany, AXIO Vert.A1). Each retina was divided into four quadrants, sharing a central quadrant, and each retinal quadrant was further subdivided into peripheral and mid-peripheral regions, with a 1 mm² sampling area per zone. Five independent experimental units were used per group for statistical analysis. The number of RBPMS-positive cells representing RGCs were counted using the ImageJ software. The average number of RGCs in the peripheral and mid-peripheral regions of each group was calculated separately.

### Electroretinography visual function tests

Sprague-Dawley rats were dark-adapted for 24 h after modeling, and thereafter all operations were performed in a dark room or under red light. Rats were anesthetized with Zoletil/xylazine (1.5%/0.2%, 1 µL/g i.p.) before connecting the electrodes, and the electrodes were inserted subcutaneously between the eyes (needle electrodes), at the root of the tail (pin electrodes), and placed on the corneal surfaces of both eyes (round electrodes). The setting modes included Scotopic 0.01, Scotopic 3.0, and Scotopic 3.0 Oscillatory Potential ERG. We assessed the a-wave amplitude by measuring from the baseline before stimulation to the wave’s lowest point. The b-wave amplitude was determined by measuring from the lowest point of the a-wave to the waveform’s highest point. The evaluation and description of the scotopic 3.0 oscillatory potentials (SOPs) primarily focused on the peaks of the three main oscillatory waveforms present in the signal. Afterwards, we set the photopic negative response (PhNR) mode and measured the slow potential with negative polarity that occurs after the b-wave. Six independent experimental units were used per group for statistical analysis.

### Statistical analysis

All quantitative data are expressed as the mean ± standard deviation (SD). Statistical analyses were conducted using the SPSS software (version 26.0; IBM Corp.). Independent samples t-tests were used for comparisons between the two groups. Multiple group comparisons were performed using one-way ANOVA, followed by Tukey’s or Games-Howell post hoc test. Welch’s test was applied to address potential variance inhomogeneity. Statistical significance was set at *p* < 0.05. All data visualizations were created using GraphPad Prism version 8 (GraphPad Software Inc., CA), with quantitative results presented as bar graphs to facilitate visual comparison of experimental outcomes.

## Results

### Characterization of ADSCs and ADSC-EVs

**Fig. 2:**
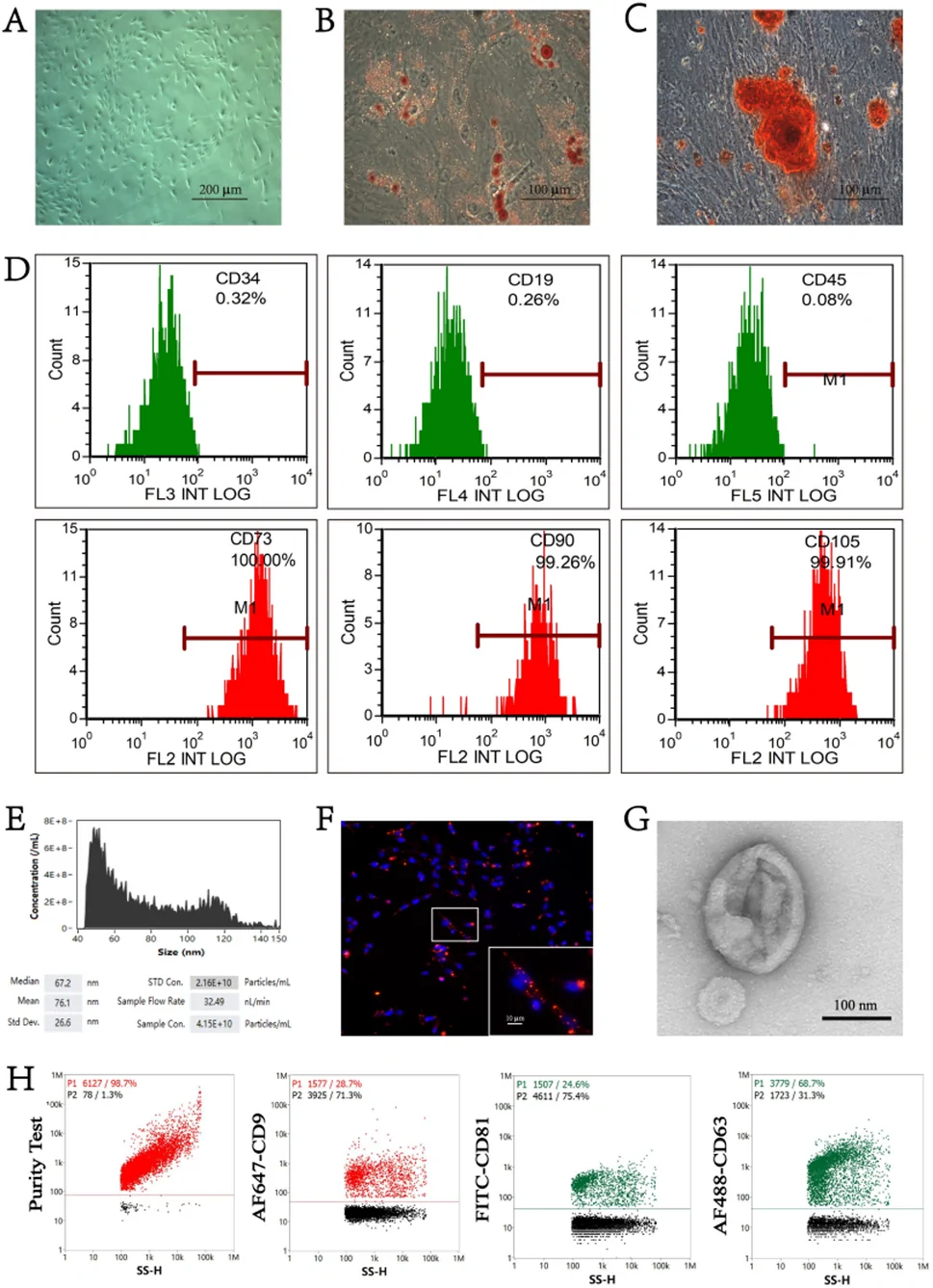
Identification of ADSCs and ADSC-EVs. **A** Morphology of ADSCs was spindle-shaped. Scale bars: 200 μm. **B**, ** C** After induced differentiation, red lipid drops (stained by Oil Red O staining) and red calcium nodules (stained by Alizarin Red staining) were found in cells. Scale bars: 200 μm. **D** Flow cytometry was used to analyze the surface markers of ADSCs. **E** Particle size distribution measured using NTA. **F** PKH-26-labeled exosomes (red) in R28 cells. Scale bars: 20 μm. **G** Under electron microscopy, the morphological observation revealed that the EVs were cup-shaped. Scale bars: 100 nm. **H** The purity of EVs and protein expression of the surface markers CD9, CD63, and CD81 on EVs and ADSCs were detected by flow cytometry analysis

In this study, adipose-derived mesenchymal stem cells were isolated and extracted from human adipose tissue according to the protocol of Anastasios et al. [29]. When cultured to the third passage, these cells displayed a characteristic spindle-shaped morphology (Fig. 2A). The multipotent differentiation capacity was verified through adipogenic and osteogenic induction assays (Fig. 2B and C). Flow cytometric analysis revealed high expression levels (> 95%) of the positive markers (CD73, CD105, and CD90) and minimal expression levels (< 5%) of the negative markers (CD34, CD45, and CD19), as illustrated in Fig. 2D.

Purified from ADSC-conditioned medium, the EVs displayed a typical cup-shaped morphology, an average size of 76.1 ± 26.6 nm, and a concentration of 4.15E + 10 particles/mL. Additionally, we observed that PKH26-labeled EVs were internalized by R28 cells (Fig. 2E-G). Figure 2H showed that the purity of EVs was 98.7%, with positive rates for the surface markers CD9 (28.7%), CD81 (24.6%), and CD63 (68.7%), consistent with established EVs characteristics [30]. Collectively, these data confirmed the successful isolation and characterization of ADSCs and their derived EVs.

### ADSC-EVs may attenuate excitotoxicity through miR-23a-5p mediated PLCD1 suppression

**Fig. 3:**
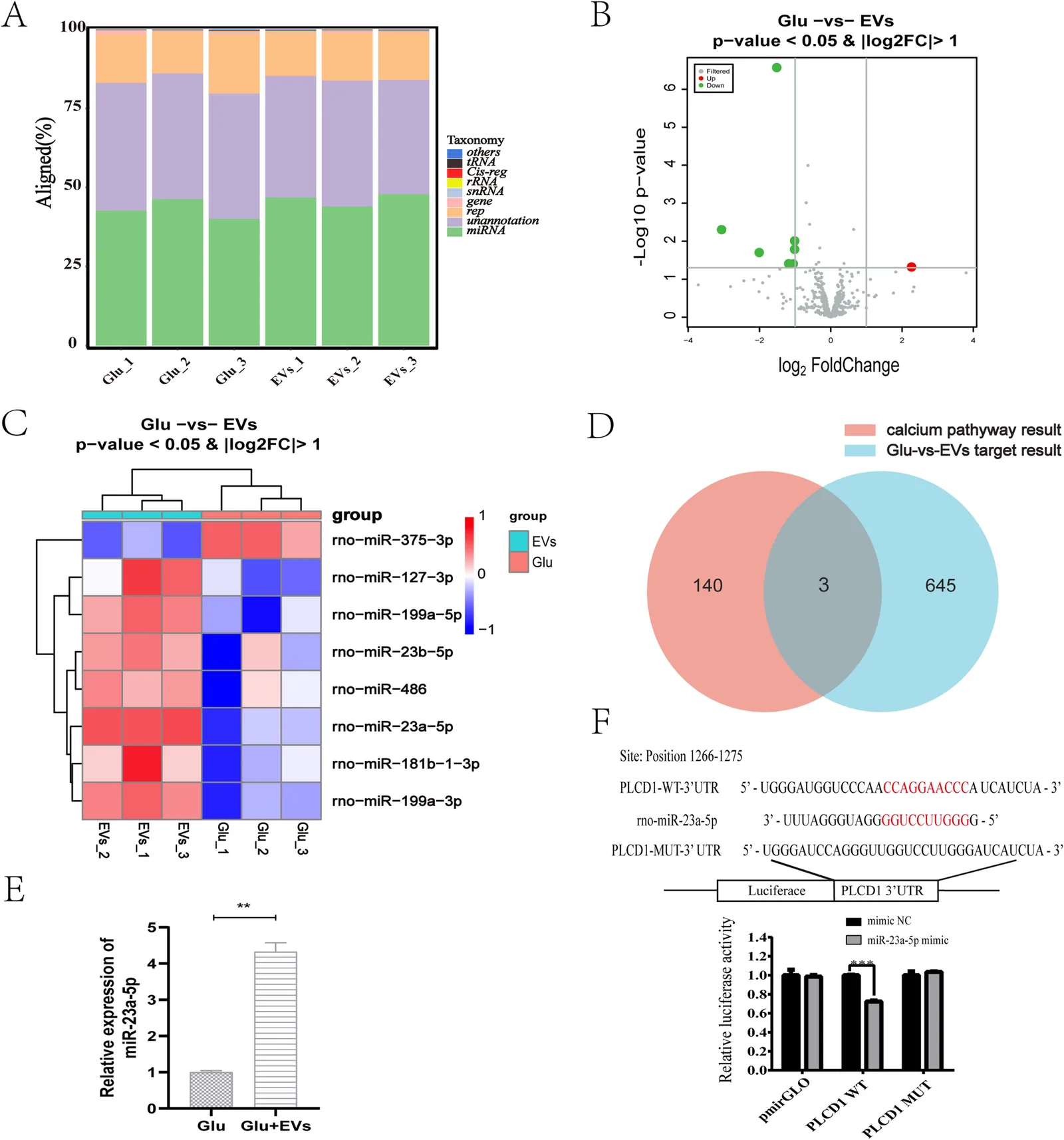
Differential analysis of microRNA expression and prediction of target genes. **A** Length distribution of small RNAs in each sample; green represents microRNAs. **B**, ** C** Volcano plots and heatmaps showing eight microRNAs with significant differentiation (|log2FoldChange|>1 and p < 0.05). **D** Venn diagram showing the correlation between differential microRNAs and calcium overload genes. **E** The differential expression of miR-23a-5p between the two groups. **p < 0.01 versus Glu group, sample size = 3. **F** Dual-luciferase reporter assay confirming direct binding. Mutation of the putative miR-23a-5p binding site in the 3’-UTR of PLCD1 mRNA abrogated the repression of luciferase activity induced by miR-23a-5p mimic. ***p < 0.001 versus miR-NC group. Values are expressed as mean ± SD

To further elucidate the mechanism of anti-excitotoxicity in ADSC-EVs, we established a glutamate-injured R28 cells model and performed small RNA sequencing analysis between the ADSC-EVs and PBS treatment groups. The small RNA expression profile confirmed that microRNAs were the predominant RNA species (Fig. 3A). And, the expression of miR-127-3p, miR-181b-1-3p, miR-199a-3p/5p, miR-23a-5p, miR-23b-5p, and miR-486 were significantly upregulated (*p* < 0.05, |log2FoldChange| > 1) in ADSC-EVs treatment group, whereas the expression of miR-375-3p was significantly downregulated (Fig. 3B and C). Our previous study found that ADSC-EVs can effectively mitigate excitotoxic damage in RGCs via regulating intracellular calcium overload [22]. To investigate the key microRNAs involved in regulating intracellular calcium ion concentration, we used miRanda to predict 648 target genes (score > 150, energy < -30) for differentially expressed microRNAs, and screened 143 genes from the KEGG database that are associated with calcium overload. Then, we identified three candidate genes, *Htr6*, *Ptk2b*, and *Plcd1*, which were common to both datasets (Fig. 3D). All three genes were predicted targets of miR-23a-5p, which showed a 4-fold upregulation (*p* < 0.05, Fig. 3E) following treatment with ADSC-EVs in glutamate-injured R28 cell models. We further confirmed that miR-23a-5p binds to the PLCD1-3’UTR binding region (*p* < 0.001, Fig. 3F).

These findings suggest that ADSC-EVs may mitigate glutamate-induced damage in R28 cells by suppressing PLCD1 expression via miR-23a-5p.

### ADSC-EVs alleviate glutamate-induced calcium overload and death of R28 cells through miR-23a-5p

**Fig. 4:**
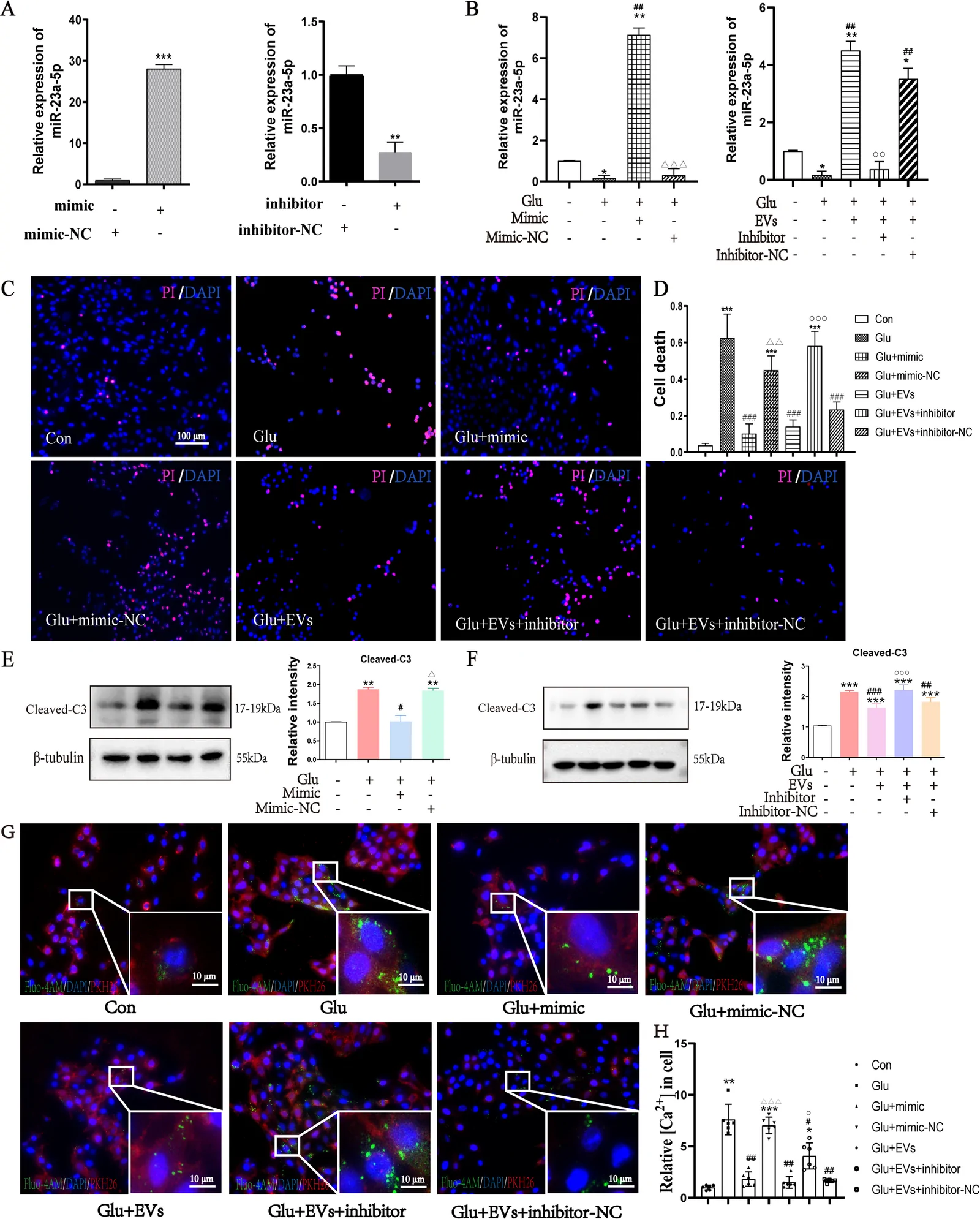
ADSC-EVs alleviate R28 damage by glutamate via miR-23a-5p. **A** Relative expression of miR-23a-5p after transfecting with miR-23a-5p-mimic and miR-23a-5p-inhibitor, sample size = 3. **B** Relative expression of miR-23a-5p under different conditions, sample size = 3. **C** PI staining showing the death of R28 cells under different conditions, scale bar = 100 µm. **(D)** Statistical analysis of PI staining, sample size = 3. **E**, ** F** Western blot showing the expression level of Cleaved-C3 under different conditions, and the statistical analysis, sample size = 3. **G** Fluo-4AM staining of intracellular calcium ion, scale bar = 10 µm. **H** Quantitative analysis of Fluo-4 AM fluorescence signal intensity, sample size = 6. *p < 0.05, **p < 0.01, ***p < 0.001 versus mimic-NC, inhibitor-NC, or Con group; #*p* < 0.05, ##*p* < 0.01, ###*p* < 0.001,versus Glu group; △*p* < 0.05, △△*p* < 0.01, △△△*p* < 0.001, versus mimic group; ○*p* < 0.05, ○○*p* < 0.01, ○○○*p* < 0.001, versus Glu + EVs group by one-way ANOVA followed by Tukey’ s or Games-Howell post hoc test. Values are expressed as mean ± SD

To investigate the anti-excitotoxic effect of miR-23a-5p, we transfected miR-23a-5p mimics (*p* < 0.001) or inhibitors (*p* < 0.01) into R28 cells respectively, with the transfection efficiency validated in Fig. 4A. Compared with the Con group, R28 cells exposed to glutamate exhibited a significant decrease in miR-23a-5p expression (Fig. 4B, *p* < 0.05), while the number of PI-positive cells (Fig. 4C and D, *p* < 0.001), the expression of cleaved caspase-3 (Fig. 4E, *p* < 0.01; Fig. 4F, *p* < 0.001), and intracellular calcium concentration (Fig. 4G and H, *p* < 0.01) were significantly increased. Compared with the Glu group, transfection with a miR-23a-5p mimic increased miR-23a-5p expression (Fig. 4B, *p* < 0.01) and significantly decreased the number of PI-positive cells (Fig. 4C and D, *p* < 0.001), the cleaved-caspase-3 expression (Fig. 4E, *p* < 0.05), and the calcium ion concentration (Fig. 4G and H, *p* < 0.01). Similarly, ADSC-EVs protected R28 cells from glutamate injury, whereas the therapeutic effect was significantly attenuated after transfection with miR-23a-5p inhibitor, as evidenced by a notable increase in PI-positive cells (Fig. 4C and D, *p* < 0.001), cleaved-caspase-3 expression (Fig. 4E, *p* < 0.001), and intracellular calcium ion concentration (Fig. 4G and H, *p* < 0.05). No such changes were observed in the inhibitor-NC group (Fig. 4B-H). These findings suggest that ADSC-EVs mitigate glutamate-induced R28 cell death and calcium overload by miR-23a-5p.

### ADSCs-EVs modulate PLCD1/PKCA/GluA2 axis in glutamate-damaged R28 cells via miR-23a-5p

**Fig. 5:**
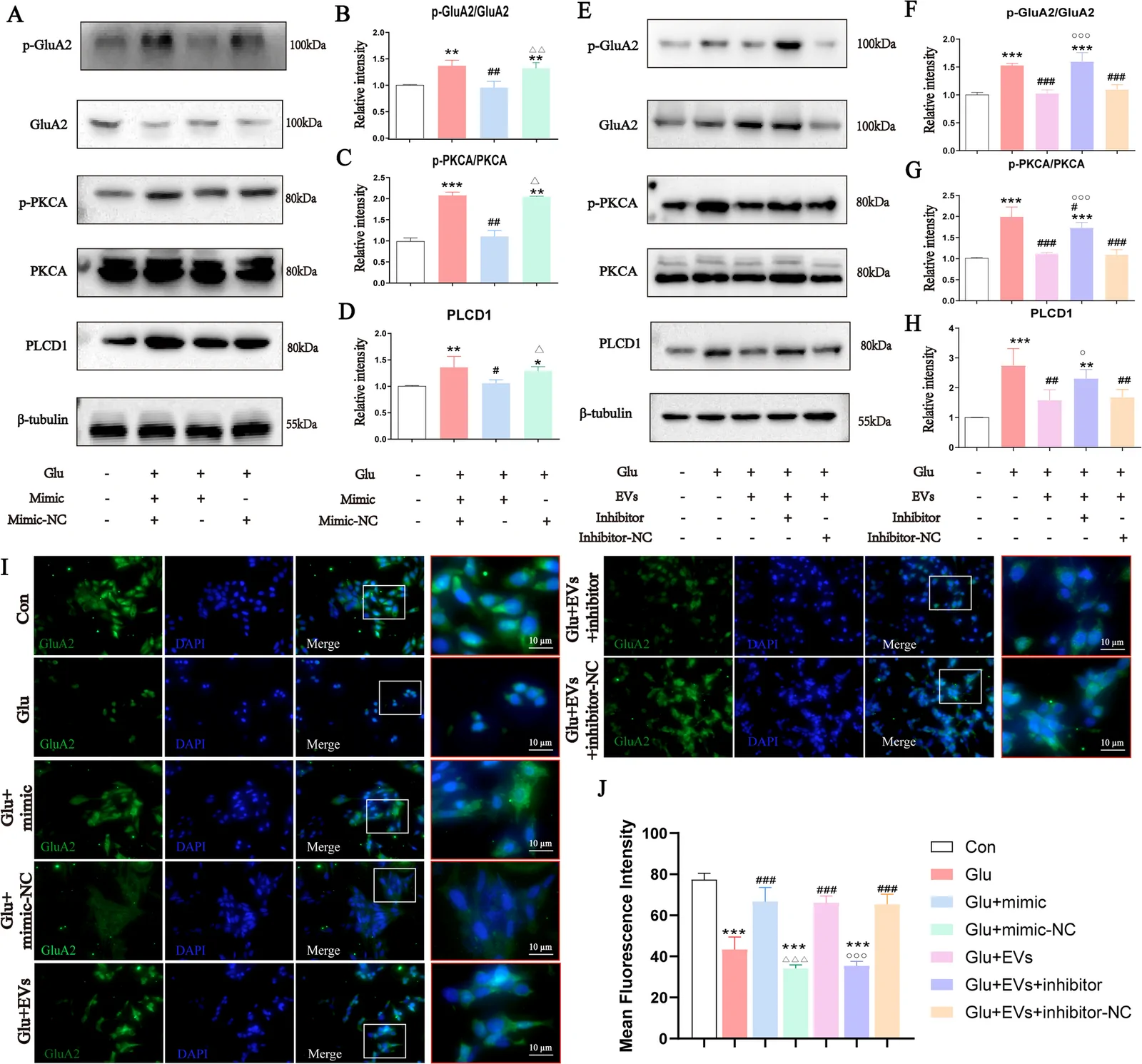
ADSCs-EVs upregulate miR-23a-5p and affect the PLCD1/PKCA/GluA2 axis *in vitro.* **A **–**H** Western blot showing the expression levels of PLCD1, p-GluA2, GluA2, p-PKCA, and PKCA under different conditions, and the statistical analysis, sample size = 3. **I** Representative immunofluorescence images showing the changes in GluA2 expression on cell membranes, from left to right are GluA2 (Alexa Fluor 488, green), DAPI (blue), and merged, Scale bar = 10 µm. **J** Bar charts representing the statistical results of mean fluorescence intensity, sample size = 3. *p < 0.05, **p < 0.01, ***p < 0.001 versus Con group; #*p* < 0.05, ##*p* < 0.01, ###*p* < 0.001,versus Glu group; △*p* < 0.05, △△*p* < 0.01, △△△*p* < 0.001, versus mimic group; ○*p* < 0.05, ○○○*p* < 0.001, versus Glu + EVs group by one-way ANOVA followed by Tukey’ s or Games-Howell post hoc test. Values are expressed as mean ± SD

Previous study identified that the phosphorylation of the PKCA/GluA2 pathway leads to calcium overload [31]. To further explore whether ADSCs-EVs regulate the PKCA/GluA2 pathway via miR-23a-5p mediated PLCD1 low-expression, we evaluated the expression of PLCD1 and the phosphorylation of PKCA and GluA2. Western blotting showed that the Glu group exhibited a significant increase in PLCD1 expression (*p* < 0.01, Fig. 5A and D; *p* < 0.001, Fig. 5E and H), as well as in the phosphorylation of PKCA (*p* < 0.001, Fig. 5A and C; *p* < 0.001, Fig. 5E and G) and GluA2 (*p* < 0.01, Fig. 5A and B; *p* < 0.001, Fig. 5E and F) when compared to the Con group. However, the miR-23a-5p mimic group downregulated the expression of PLCD1 (*p* < 0.05, Fig. 5A and D) and the phosphorylation level of PKCA (*p* < 0.001, Fig. 4A and C) and GluA2 (*p* < 0.001, Fig. 4A and B) relative to those in the Glu group. Similarly, the ADSC-EVs treatment group inhibited the expression of PLCD1 (*p* < 0.01, Fig. 5E and H) and the phosphorylation of PKCA (*p* < 0.001, Fig. 4E and G) and GluA2 (*p* < 0.001, Fig. 5E and F) compared with the Glu group. After transfection with inhibitor-23a-5p, the regulatory effect of ADSC-EVs was significantly attenuated, which is substantiated by the significant upregulation of the expression of PLCD1 (*p* < 0.05, Fig. 5E and H) and the phosphorylation of PKCA (*p* < 0.001, Fig. 5E and G) and GluA2 (*p* < 0.001, Fig. 5E and F). No changes were observed in the mimic-NC and inhibitor-NC groups.

Phosphorylation of GluA2 results in low expression of GluA2 on the cell membrane [31, 32]. Our findings confirmed that GluA2 expression on R28 cell membranes was significantly lower in the Glu group (*p* < 0.001) relative to the Con group. The expression of GluA2 on R28 cell membranes was effectively increased by ADSC-EVs treatment (*p* < 0.001) and miR-23a-5p mimic transfection (*p* < 0.001) compared with the Glu group. However, following transfection with the inhibitor-23a-5p, GluA2 expression on the cell membrane was significantly decreased (*p* < 0.001) compared to ADSC-EVs treatment group (Fig. 5I and J).

These results demonstrate that ADSC-EVs can specifically target and downregulate PLCD1 expression by miR-23a-5p. This process inhibits the phosphorylation of PKCA/GluA2 and decreases the expression of the GluA2 subunit on the cell membrane, hence mitigating the calcium overload.

### MiR-23a-5p regulates the PLCD1/PKCA/GluA2 axis to alleviate glutamate-induced death and visual impairment in RGCs

**Fig. 6:**
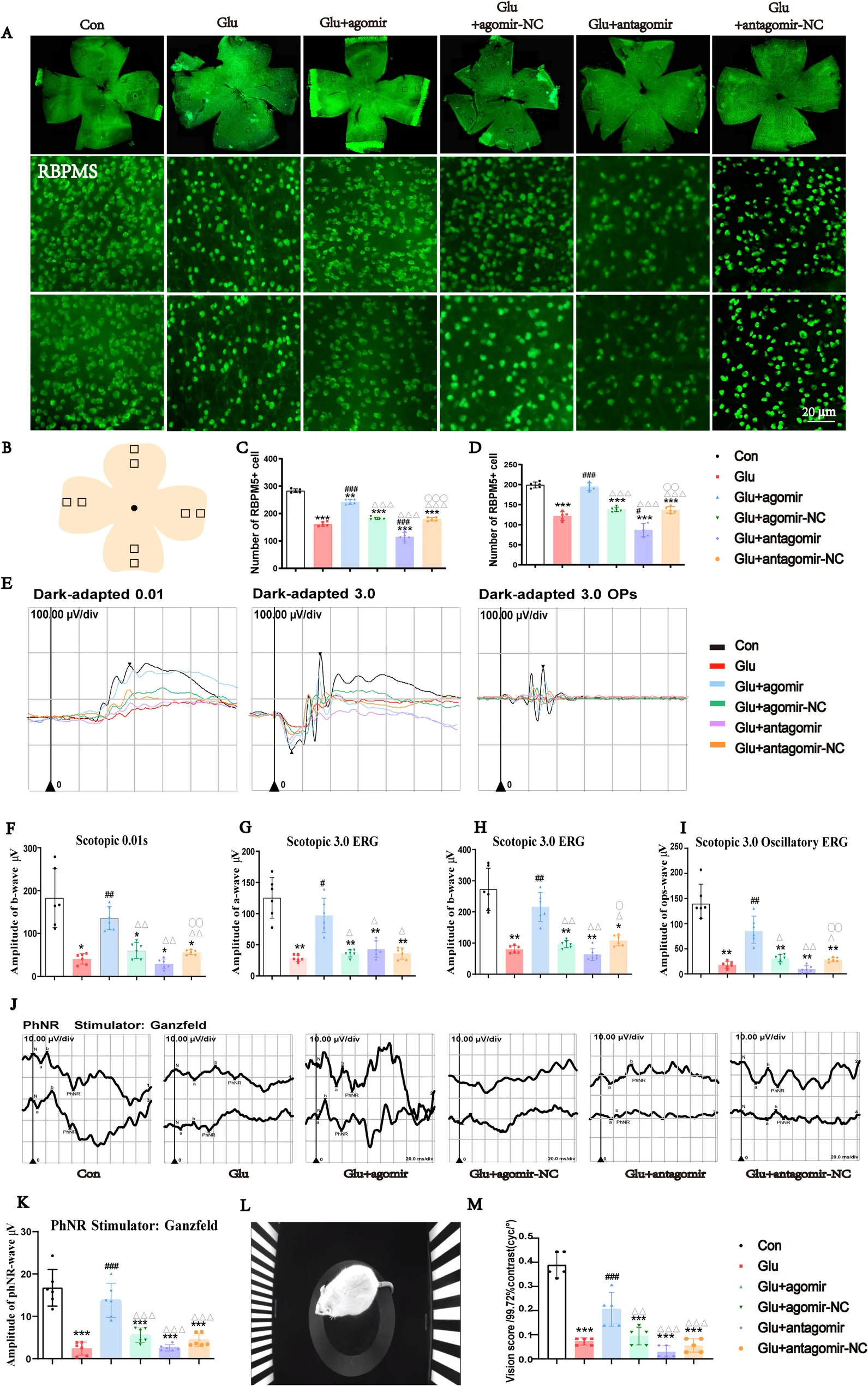
MiR-23a-5p alleviates glutamate-induced RGCs death and vision function impairment *in vivo.* **A** Immunofluorescence was performed to detect the number of RBPMS-positive cells (green) in the different groups. Scale bar = 20 µm. **B** Selection of a 1 mm^2^ area of the mid-peripheral and peripheral retina. **C** Statistical analysis of RBPMS-positive cells in the midperipheral retina, sample size = 5. **D** Statistical analysis of RBPMS-positive cells in the peripheral retina, sample size = 5. **E** Representative waveforms of dark-adapted ERG under various conditions. **F** Representative waveforms of bright-adapted PhNR under different conditions. **G**–**N** Statistical analysis of a-, b-, and OPs-waves in dark-adapted fERG responses, sample size = 6. **M** Statistical analysis of brightly adapted PhNR waves, sample size = 6. **N** Experimental procedure for OptoDrum visual resolution measurement. **O** Statistical analysis of OptoDrum visual resolution, sample size = 6. *p < 0.05, **p < 0.01, ***p < 0.001 versus Con group; #*p* < 0.05, ##*p* < 0.01, ###*p* < 0.001,versus Glu group; △*p* < 0.05, △△*p* < 0.01, △△△*p* < 0.001, versus mimic group; ○*p* < 0.05, ○○*p* < 0.01, ○○○*p* < 0.001, versus Glu + antagomir group by one-way ANOVA followed by Tukey’ s or Games-Howell post hoc test. Values are expressed as mean ± SD

To investigate the role of miR-23a-5p in retinal excitotoxicity, we applied agomir-23a-5p and antagomir-23a-5p as pretreatment measures and assessed the number of RGCs and visual performance in a glutamate-triggered retinal damage model. Our results showed that glutamate significantly decreased the number of RBPMS-positive cells (RGCs) in the mid-peripheral (*p* < 0.001) and peripheral (*p* < 0.001) retinas compared with the Con group. Injection of agomir-23a-5p significantly increased the number of RBPMS-positive cells in the mid-peripheral (*p* < 0.001) and peripheral (*p* < 0.001) retinas, whereas more severe changes occurred in the mid-peripheral (*p* < 0.001) and peripheral (*p* < 0.05) retinas of the Glu+antagomir-23a-5p group compared with the Glu group (Fig. 6A-D). Electroretinographic analysis revealed that glutamate significantly decreased the amplitudes of a- (*p* < 0.01, Fig. 6E and G), b- (*p* < 0.05, Fig. 6E and F; *p* < 0.01, Fig. 5E and H), SOPs- (*p* < 0.01, Fig. 6E and I), and PhNR- (*p* < 0.001, Fig. 6J and K) waves compared with the Con group. Compared to the Glu group, injection of angomir-23a-5p significantly alleviated the decrease in a- (*p* < 0.05, Fig. 6E and G), b- (*p* < 0.01, Fig. 6E and F; *p* < 0.01, Fig. 6E and H), SOPs- (*p* < 0.01, Fig. 6E and I), and PhNR- (*p* < 0.001, Fig. 6J and K) wave amplitudes, however these alterations were not markedly exacerbated in the Glu + antagomir group (Fig. 6E-K). Similarly, the results of the OptoDrum visual resolution assay were consistent with the above results (Fig. 6L and M). These findings imply that miR-23a-5p reduces glutamate-induced RGCs death and inner retina-associated visual function impairment.

**Fig. 7:**
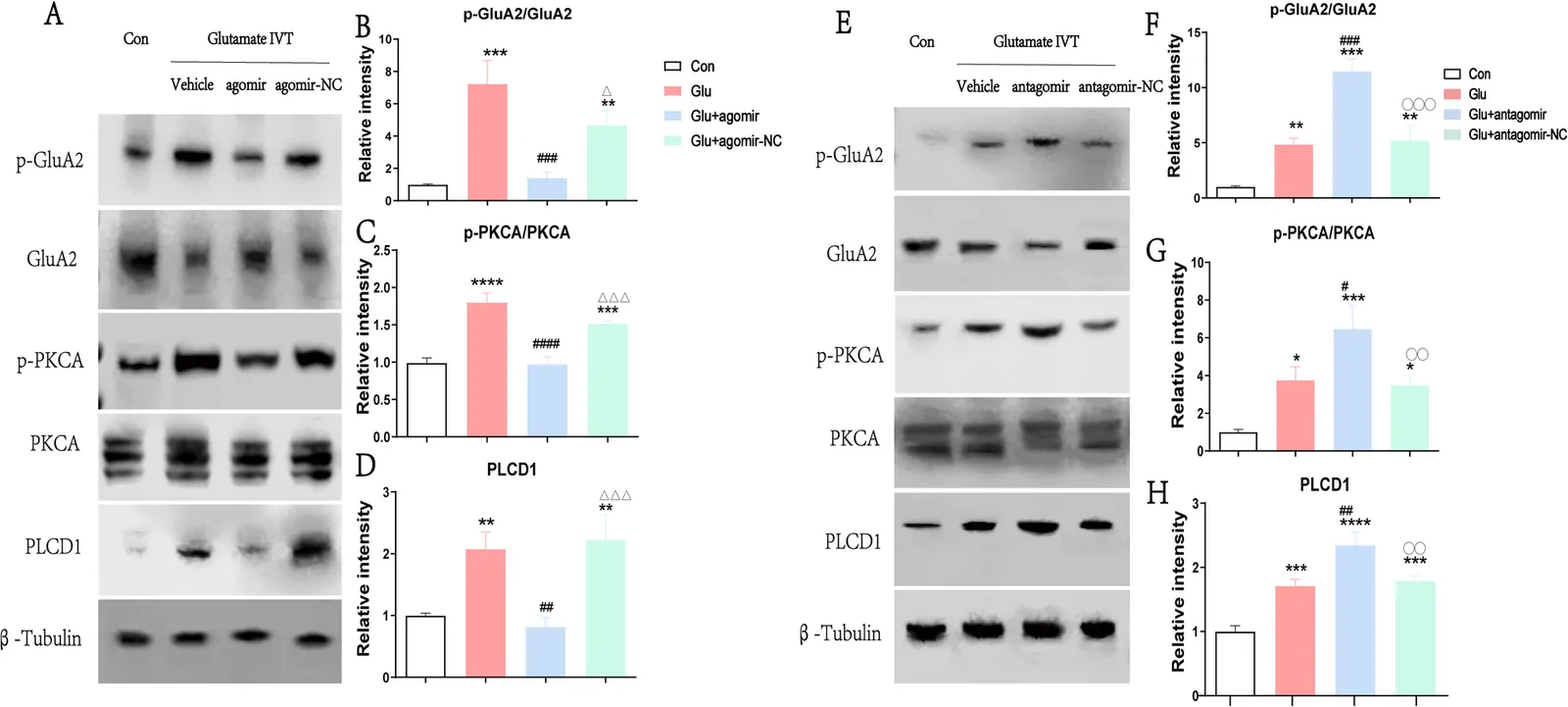
MiR-23a-5p regulates the PLCD1/PKCA/GluA2 axis in vivo. **A** Validation of the protective effect of agomir-23a-5p against glutamate injury in vivo by representative bands of western blotting. **B**–**D** The statistical analysis of p-PKCA/PKCA, p-GluA2/GluA2 and PLCD1, sample size = 3. **E** Representative bands of western blotting involve in the modulatory effect of antagomir-23a-5p on glutamate damage. **F**–**H** The statistical analysis of p-PKCA/PKCA, p-GluA2/GluA2, and PLCD1, sample size = 3. *p < 0.05, **p < 0.01, ***p < 0.001, ****p < 0.0001 versus Con group; #*p* < 0.05, ##*p* < 0.01, ###*p* < 0.001,versus Glu group; △*p* < 0.05, △△△*p* < 0.001, versus mimic group; ○○*p* < 0.01, ○○○*p* < 0.001, versus Glu + antagomir group by one-way ANOVA followed by Tukey’ s or Games-Howell post hoc test. Values are expressed as mean ± SD

To assess the regulation of the PLCD1/PKCA/GluA2 axis by mir-23a-5p in vivo, we examined the expression of PLCD1, p-GluA2, GluA2, p-PKCA, and PKCA in each group. Western blotting analysis showed that intravitreal injection of glutamate significantly upregulated retinal PLCD1 expression (*p* < 0.01, Fig. 7A and D; *p* < 0.001, Fig. 7E and H) and increased the phosphorylation of PKCA (*p* < 0.0001, Fig. 7A and C; *p* < 0.05, Fig. 7E and G) and GluA2 (*p* < 0.001, Fig. 7A and B; *p* < 0.01, Fig. 7E and F). The expression of PLCD1 (*p* < 0.01, Fig. 7A and D) and the phosphorylation of PKCA (*p* < 0.0001, Fig. 7A and C) and GluA2 (*p* < 0.001, Fig. 7A and B) were substantially downregulated by agomir-23a-5p treatment, whereas antagomir-23a-5p conversely exacerbated both PLCD1 upregulation (*p* < 0.01, Fig. 7E and H) and the phosphorylation of PKCA (*p* < 0.05, Fig. 7E and G) and GluA2 (*p* < 0.001, Fig. 7E and F) compared to the Glu group. These results illustrate that miR-23a-5p exerts neuroprotective effects by suppressing PLCD1-mediated PKCA/GluA2 phosphorylation.

## Discussion

Excitotoxicity, a common pathological mechanism following retinal disorders, has been shown to significantly correlate with both structural and functional outcomes in the retina [33, 34]. Previous studies reported that MSC-EVs could perform a neuroprotective effect via functional microRNAs [23, 24]. Our previous studies demonstrated that ADSC-EVs can mitigate glutamate-induced excitotoxic damage in retinal disorders [22]. In the present study, glutamate-induced calcium overload was attenuated by ADSC-EVs, which was associated with increased expression of miR-23a-5p in R28 cells. We further showed that upregulating miR-23a-5p in retina alleviates glutamate-induced RGCs death and visual function impairment. These findings suggest that miR-23a-5p plays a potentially important role in glutamate-induced excitotoxic injury. Additionally, miR-23a-5p was found to be a negative regulator of advanced glycation end product receptor (RAGE) and downstream reactive oxygen species (ROS) signaling activation [35], and negatively regulates peroxiredoxin-2 (PRDX2) and vascular endothelial growth factor (VEGF) expression [36]. Oxidative stress, VEGF pathway activation, and neurodegenerative alteration are typical pathological processes in retinal disorders. Based on the earlier works and our results, we suggest that miR-23a-5p may serve as a promising therapeutic approach for treating retinal conditions.

Early studies implicated miR-23a-5p in the regulation of apoptosis in tumor cells [37, 38]. However, previous studies have also shown that M2 microglia-derived extracellular vesicles promote white matter repair and functional recovery after cerebral ischemia in mice via miR-23a-5p [39]. This research team further revealed that miR-23a-5p effectively attenuated the disruption of the blood-brain barrier after cerebral ischemia by targeting tumor necrosis factor and regulating the expression of matrix metalloproteinase 3 (MMP3), nuclear factor κB, and p65 subunits [40]. With upregulation of TNF and members of the MMP family established as mediators of neuronal apoptosis, and given that MMP9 is upregulated following retinal ganglion cell (RGC) injury while MMP inhibition (including MMP-2/-9 and MMP-3) significantly improves RGC survival and promotes axonal regeneration [41], we propose that miR-23a-5p exerts its protective effects in RGCs by suppressing these pro-inflammatory and pro-apoptotic pathways. This inference aligns with our experimental observations and offers a plausible explanation for the context-dependent functional roles of miR-23a-5p across different cellular settings.

PLCD1, a phospholipase C isoform, hydrolyzes phosphatidylinositol 4,5-bisphosphate (PIP2) into inositol trisphosphate (IP3) and diacylglycerol (DAG), the DAG can activate PKCA signaling. Our previous studies demonstrated that ADSC-EVs can inhibit the calcium permeation of AMPARs in glutamate-related pathological environments by modulating the PKCA signaling pathway [22]. This study further validated that, in a model of glutamate-induced damage, ADSC-EVs-regulated miR-23a-5p inhibited the phosphorylation of PKCA/GluA2 by decreasing the expression of PLCD1. As early as 1995 S. Shimohama reported that PLCD1 antibodies strongly stained neurofibrillary tangles (NFTs) in the brains of Alzheimer’s disease (AD) patients [42]. Matsushima et al. found PLCD1 activity in the peripheral blood of AD patients [43]. In 2013, Kim A. Staats demonstrated elevated expression of PLCD1 in the common neurodegenerative disease - Amyotrophic Lateral Sclerosis (ALS), however knockdown of the *plcd1* gene led to increased survival in ALS while glial cell function was not affected [44]. Therefore, PLCD1 may be a potential therapeutic target for treating neurodegenerative diseases. In addition, PLCD1 has been observed to be involved in the apoptosis and migration of tumor cells [45, 46]. All these works reveal that miR-23a-5p inhibits the PLCD1/PKCA/GluA2 axis in RGCs may be the key mechanism underlying their anti-excitotoxic effects.

Although this study focuses on ADSC-EVs and miR-23a-5p, current findings do not provide direct evidence that the miR-23a-5p is delivered by ADSC-EVs into retinal neurons. Comparison via the miRBase database confirmed that the mature sequence of miR-23a-5p is identical between humans and rats. Previous findings indicated that the miR-23a family is essential for mesenchymal stem cell survival and contributes to osteogenic differentiation capacity. Moreover, sequencing data from ADSC-EVs have revealed that the miR-23a family is the most abundantly expressed miRNA group in these vesicles [47–49]. Therefore, we hypothesize that miR-23a-5p, a microRNA involved in regulating excitotoxicity, is predominantly delivered by ADSC-EVs. Nevertheless, the potential contribution of other molecular mediators or signaling pathways cannot be excluded, a possibility that merits further investigation.

This study utilized intravitreal glutamate injections to mimic excitotoxic damage in the retina, although this model only reflects an acute form of injury. Further validation using models of chronic diseases is necessary to increase the clinical relevance of our results [50]. Additionally, while microRNA-based therapies seem promising, intravitreal delivery of miR-23a-5p still presents challenges, including rapid degradation and off-target effects [51]. In recent years, the construction of nanoparticle-encapsulated microRNA formulations has been proposed to improve the stability and targeted delivery [52, 53]. Recent studies have also been conducted to genetically engineer ADSCs to stably overexpress miR-23a-5p to construct an optimized miR-23a-5p-based small-molecule drug [54, 55]. In addition, enhancing the efficacy of microRNAs through the implantation of scaffolds, especially hydrogels, to achieve the sustained effect of miR-23a-5p, provides a viable solution for prolonging the bioavailability of miR-23a-5p [56, 57].

## Conclusions

In summary, our study indicates that ADSC-EVs protect against glutamate-induced excitotoxicity via miR-23a-5p-mediated suppression of the PLCD1/PKCA/GluA2 axis and reduced calcium permeability in AMPARs. These findings highlight miR-23a-5p as a key mediator and suggest its potential for future exploration as a therapeutic target for retinal disorders (Graphical Abstract).

## Supplementary Information

Below is the link to the electronic supplementary material.

Supplementary Material 1.

## Data Availability

All data generated or analysed during this study are included in this published article. The small RNA sequencing data have been provided in Supplementary file 1.

## Abbreviations

ADSC-EVs: Adipose stem cell-derived extracellular vesicles
MSC-EVs: Mesenchymal stem cell-derived extracellular vesicles
EVs: Extracellular vesicles
MiRNA: MicroRNA
RGCs: Retinal ganglion cells
PKCA: Protein kinase C alpha
GluA2: AMPARs subunit 2
PLCD1: Phospholipase C delta 1
RGCs: Retinal ganglion cells
AMPAR: α-amino-3-hydroxy-5-methyl-4-isoxazoleproprionic acid receptor
SD: Sprague-Dawley
R28 cell: Rat retinal precursor cells
SOPs: Scotopic 3.0 oscillatory potentials
PhNR: Photopic negative response
qRT-PCR: Quantitative real-time polymerase chain reaction
PI: Propidium iodide
NTA: Nanoparticle tracking analysis
PKH-26: Red PKH membrane dye
I.P.: Intraperitoneal injection
