# Supplementary material for: Adipose stem cells derived extracellular vesicles alleviate retinal excitotoxicity via miR-23a-5p/PLCD1/PKCA/GluA2 axis: a potential therapeutic strategy
Source: Stem Cell Res Ther. 2026 Jun 30;17:321. doi: 10.1186/s13287-026-05140-z (PMC13587503; doi:10.1186/s13287-026-05140-z)

Fig1

A

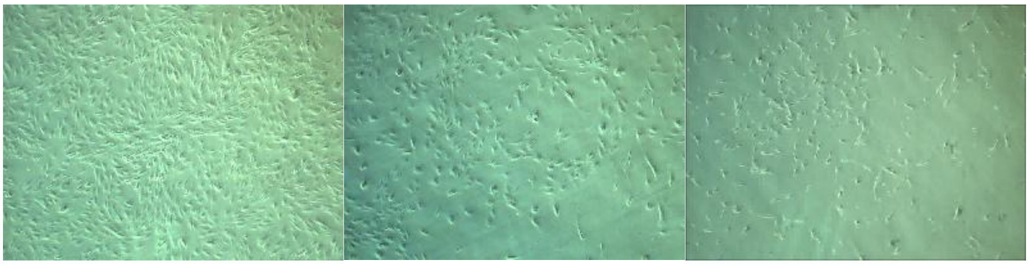

B

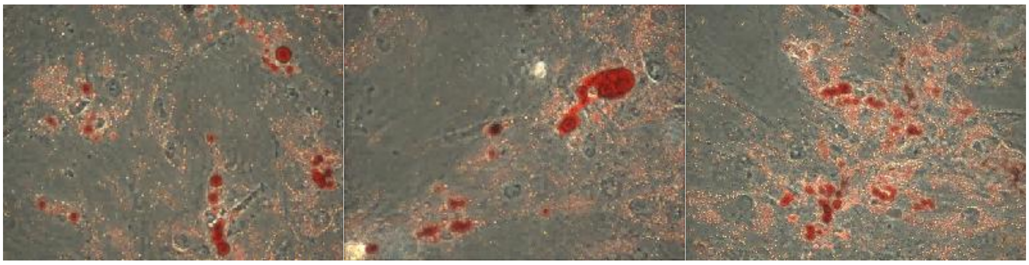

C

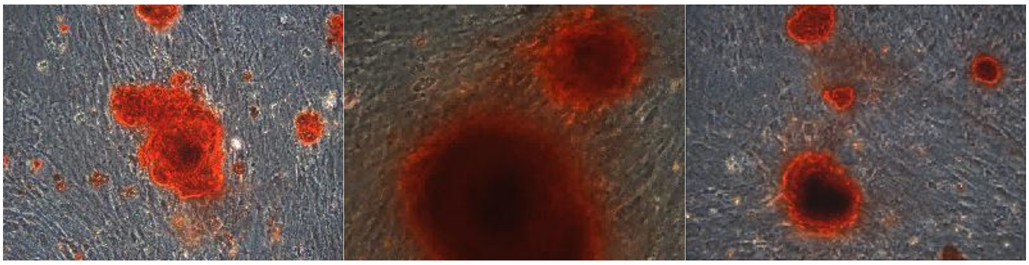

D

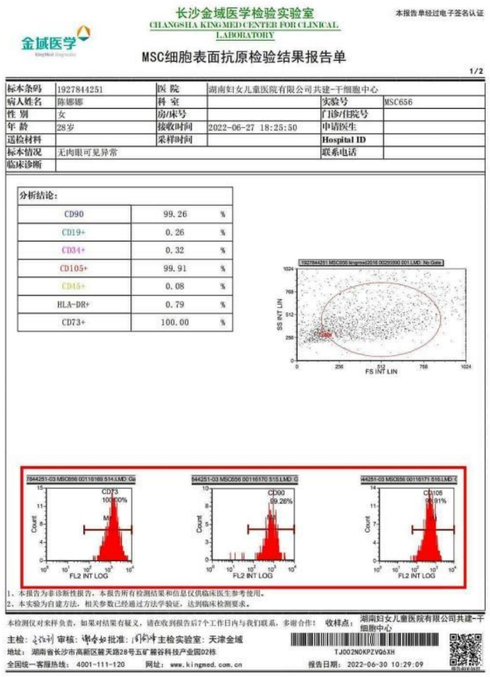

本报告单经过电子签名认证

金域医学

长沙金域医学检验实验室
CHANGSHA KINGMED CENTER FOR CLINICAL LABORATORY

MSC细胞表面抗原检验结果报告单

1/2

标本条码	1927844251	医 院	湖南妇女儿童医院有限公司共建-干细胞中心		
病人姓名	陈娜娜	科 室		实验号	MSC656
性 别	女	房/床号		门诊/住院号	
年 龄	28岁	接收时间	2022-06-27 18:25:50	申请医生	
送检材料		采样时间		Hospital ID	
标本情况	无肉眼可见异常			联系电话	
临床诊断					

分析结论:		
CD90	99.26	%
CD19+	0.26	%
CD34+	0.32	%
CD105+	99.91	%
CD45+	0.08	%
HLA-DR+	0.79	%
CD73+	100.00	%

1、本报告为非诊断性报告，本报告所有检测结果和信息仅供临床医生参考使用。
2、本实验为自建方法，相关参数已经通过方法学验证，达到临床检测要求。

本检测仅对来样负责，如果对结果有疑义，请在收到报告后7个工作日内与我们联系，多谢合作！ 收样点：湖南妇女儿童医院有限公司共建-干细胞中心

主检： 审核： 批准： 主检实验室：天津金域

地址：湖南省长沙市高新区麓天路28号五矿麓谷科技产业园D2栋

全国统一客服热线：4001-111-120 网址：www.kingmed.com.cn

TJ002N0KPZVQ6XH

报告日期：2022-06-30 10:29:09

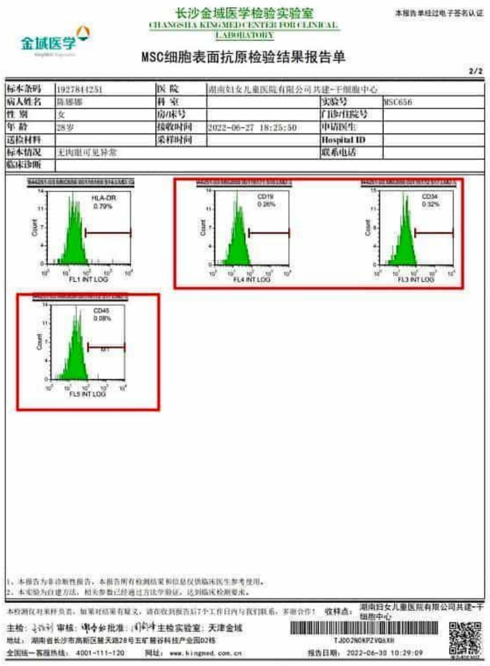

本报告单经过电子签名认证

金域医学

长沙金域医学检验实验室
CHANGSHA KINGMED CENTER FOR CLINICAL LABORATORY

MSC细胞表面抗原检验结果报告单

2/2

标本条码	1927844251	医 院	湖南妇女儿童医院有限公司共建-干细胞中心		
病人姓名	陈娜娜	科 室		实验号	MSC656
性 别	女	房/床号		门诊/住院号	
年 龄	28岁	接收时间	2022-06-27 18:25:50	申请医生	
送检材料		采样时间		Hospital ID	
标本情况	无肉眼可见异常			联系电话	
临床诊断					

1、本报告为非诊断性报告，本报告所有检测结果和信息仅供临床医生参考使用。
2、本实验为自建方法，相关参数已经通过方法学验证，达到临床检测要求。

本检测仅对来样负责，如果对结果有疑义，请在收到报告后7个工作日内与我们联系，多谢合作！ 收样点：湖南妇女儿童医院有限公司共建-干细胞中心

主检： 审核： 批准： 主检实验室：天津金域

地址：湖南省长沙市高新区麓天路28号五矿麓谷科技产业园D2栋

全国统一客服热线：4001-111-120 网址：www.kingmed.com.cn

TJ002N0KPZVQ6XH

报告日期：2022-06-30 10:29:09

E

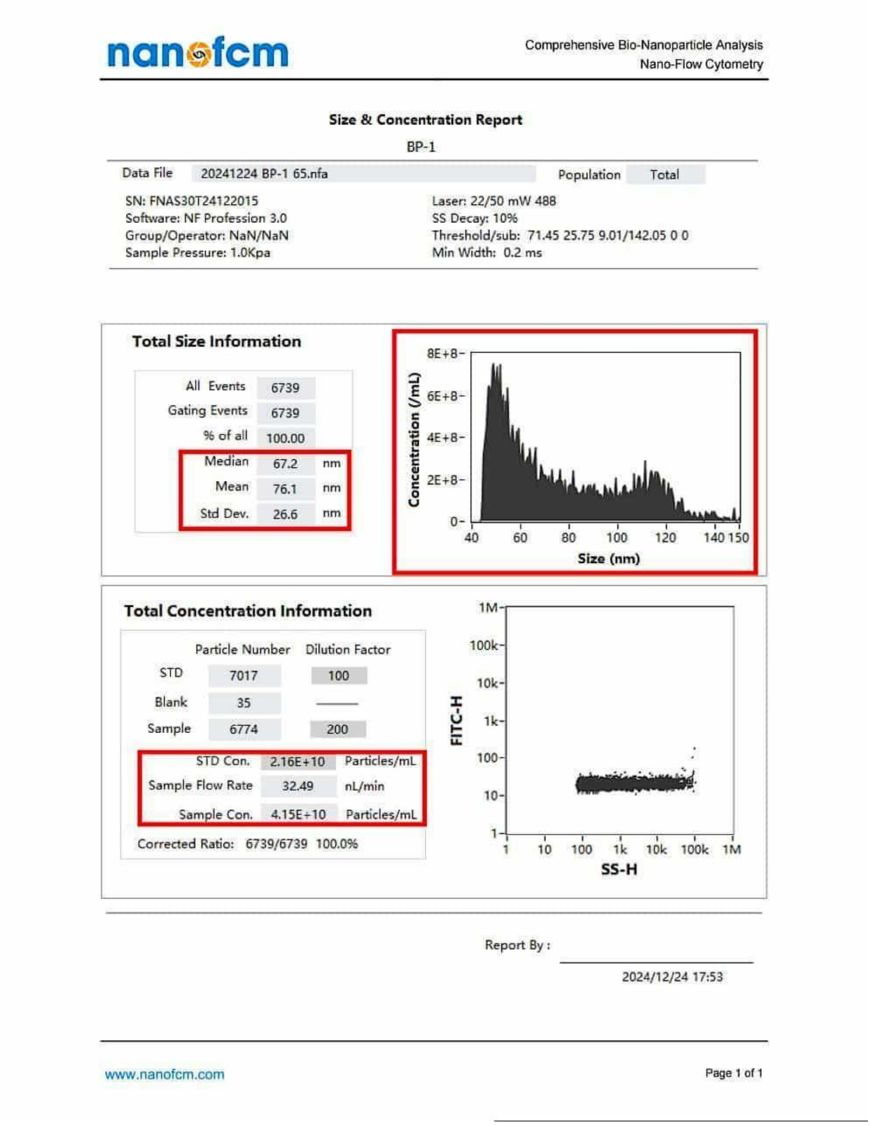

nanofcm

Comprehensive Bio-Nanoparticle Analysis
Nano-Flow Cytometry

Size & Concentration Report

BP-1

Data File	20241224 BP-1 65.nfa	Population	Total

SN: FNAS30T24122015
Software: NF Profession 3.0
Group/Operator: NaN/NaN
Sample Pressure: 1.0Kpa

Laser: 22/50 mW 488
SS Decay: 10%
Threshold/sub: 71.45 25.75 9.01/142.05 0 0
Min Width: 0.2 ms

Total Size Information

All Events	6739	
Gating Events	6739	
% of all	100.00	
Median	67.2	nm
Mean	76.1	nm
Std Dev.	26.6	nm

Total Concentration Information

	Particle Number	Dilution Factor
STD	7017	100
Blank	35	——
Sample	6774	200

STD Con.	2.16E+10	Particles/mL
Sample Flow Rate	32.49	nL/min
Sample Con.	4.15E+10	Particles/mL

Corrected Ratio: 6739/6739 100.0%

Report By :

2024/12/24 17:53

www.nanofcm.com

Page 1 of 1

F

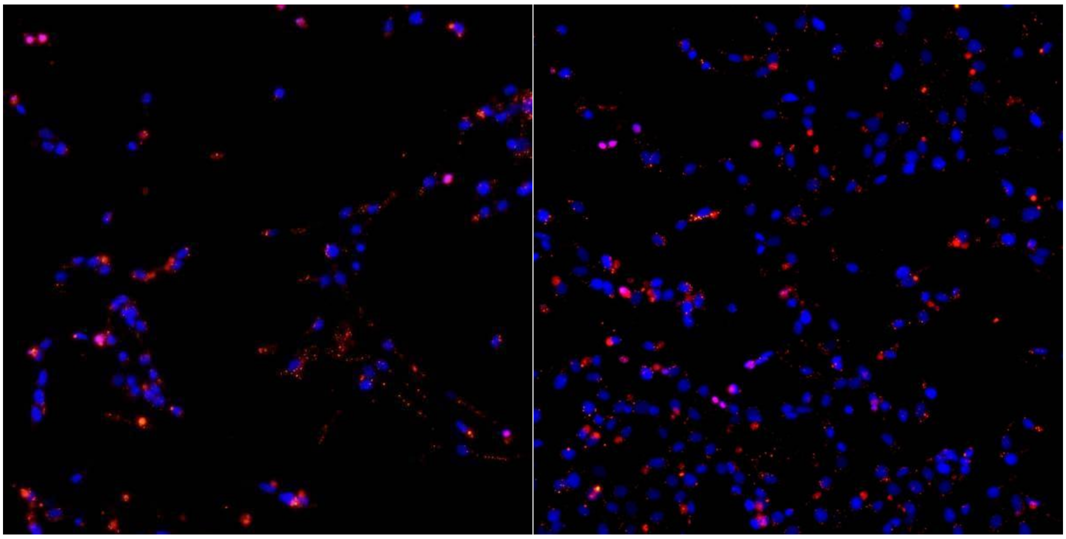

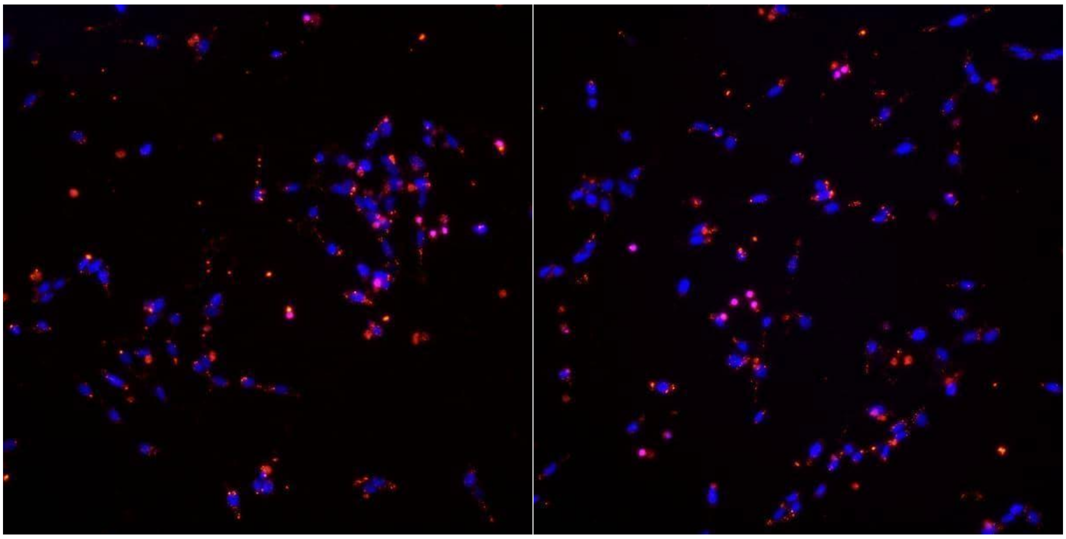

G

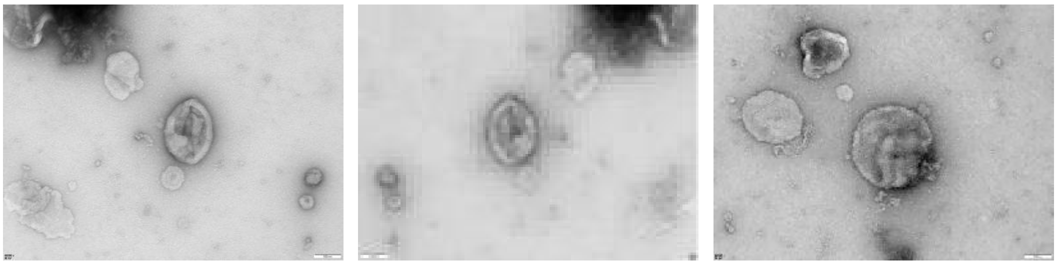

H

NanoFCM Inc. 2024

荧光分析

样品名称	ASC-EXO-1	ASC-EXO-2	ASC-EXO-3
纯度检测	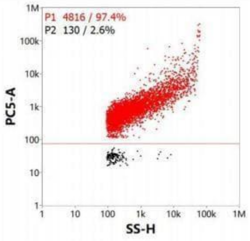	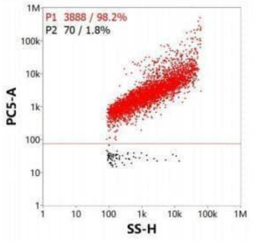	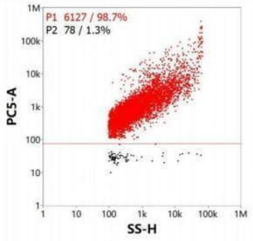
FITC-CD81	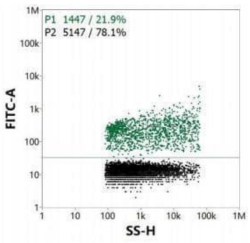	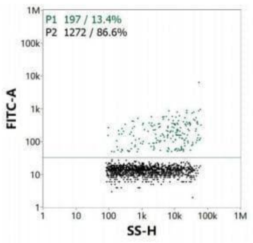	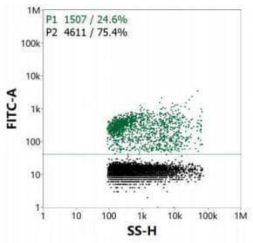
AF488-CD63	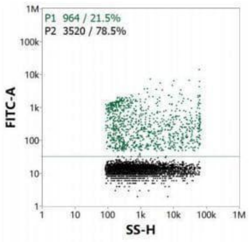	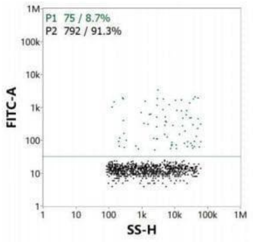	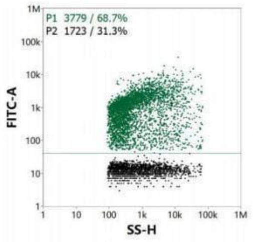
AF647-CD9	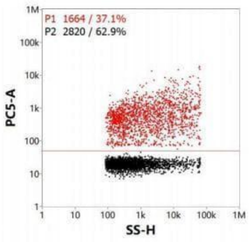	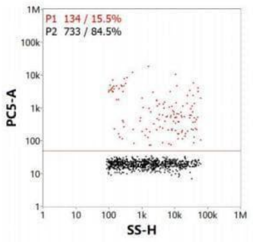	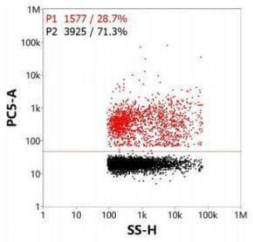

厦门福流生物科技有限公司 www.nanofcm.com 联系电话：+86592-2091013

Copyright ©by NanoFCM Inc. All Rights Reserved.

Fig 4

C, D （The red boxes represent the picture shown in the results）

Con group

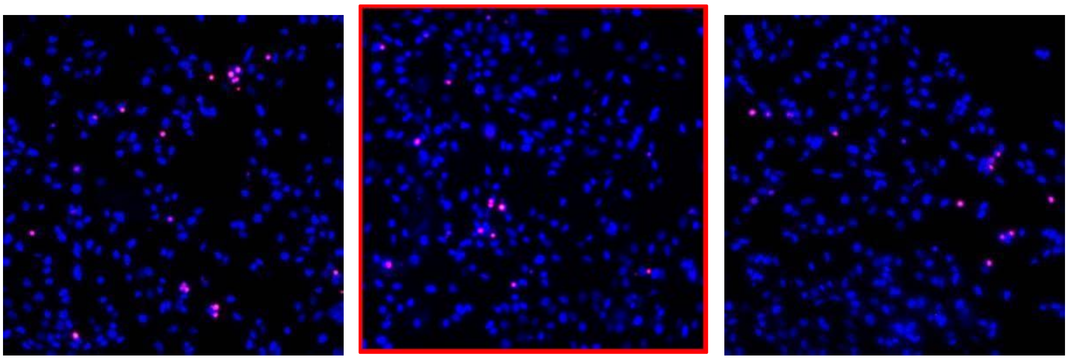

Glu group

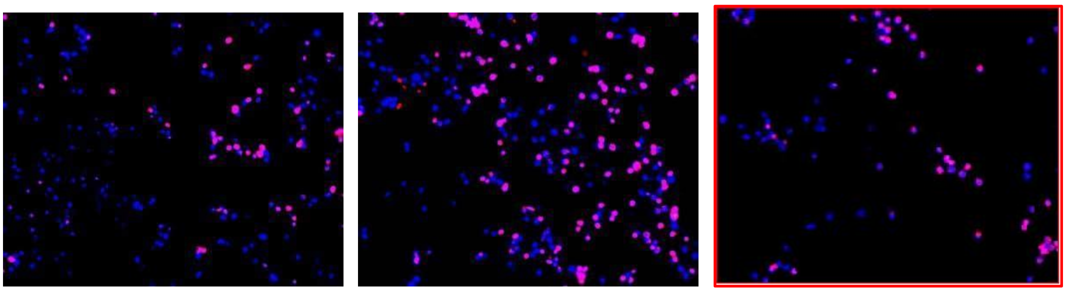

Glu + mimic

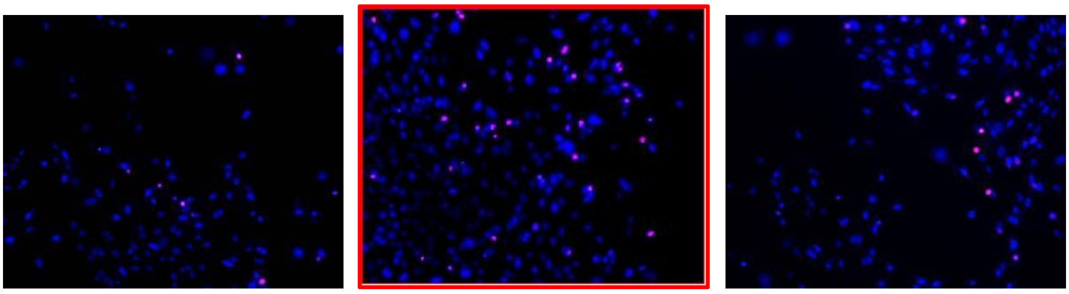

Glu + mimic NC

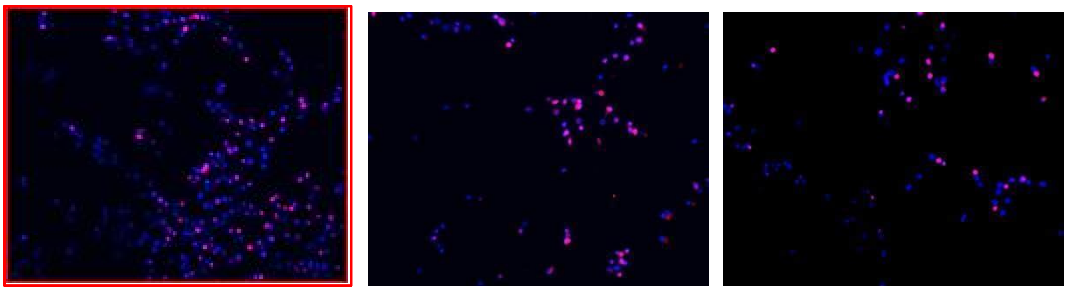

Glu + EVs

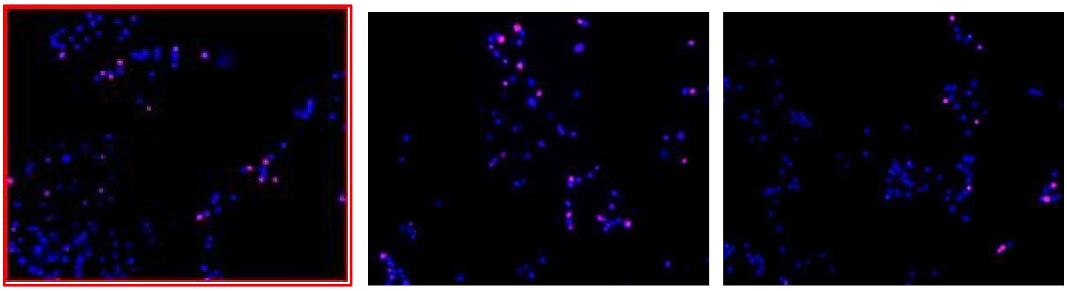

Glu + EVs + inhibitor

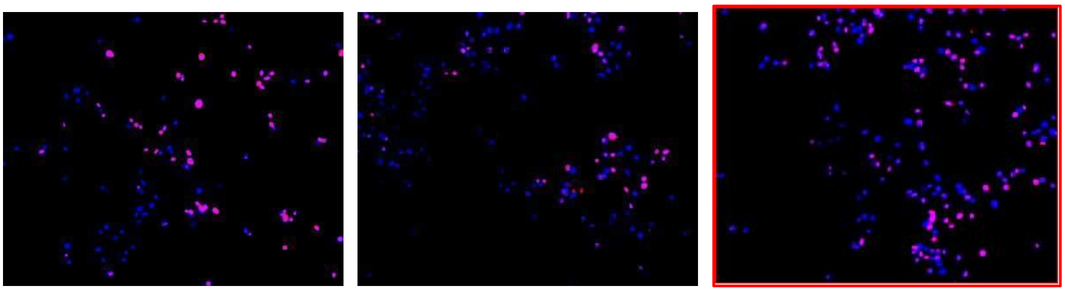

Glu + EVs + inhibitor NC

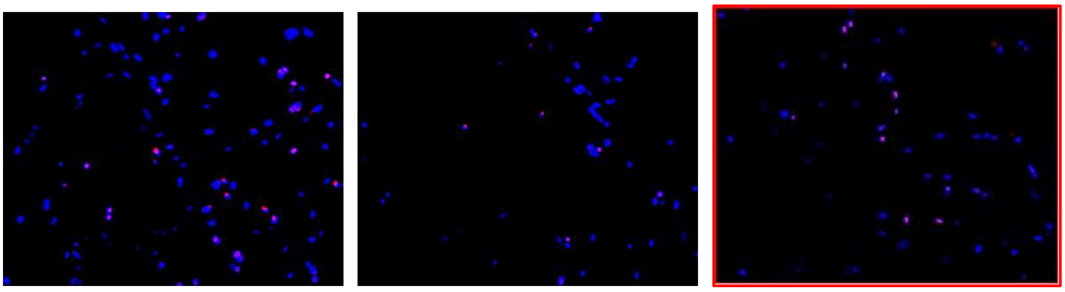

E- Cleaved-C3

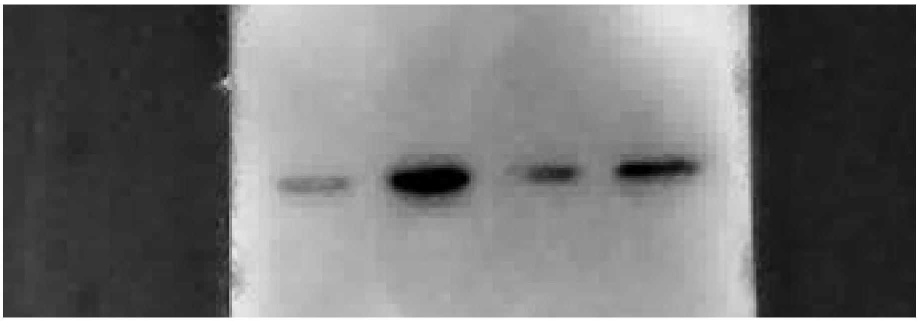

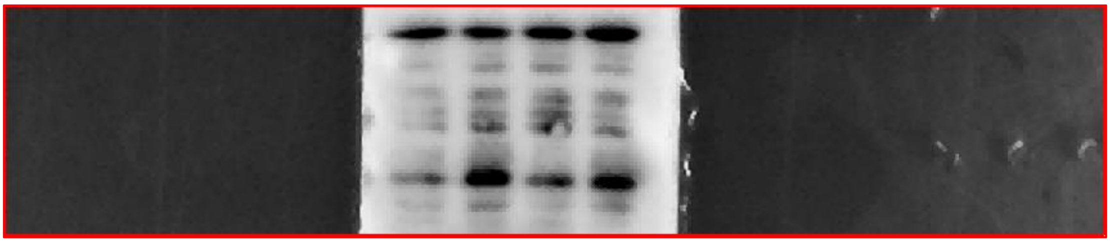

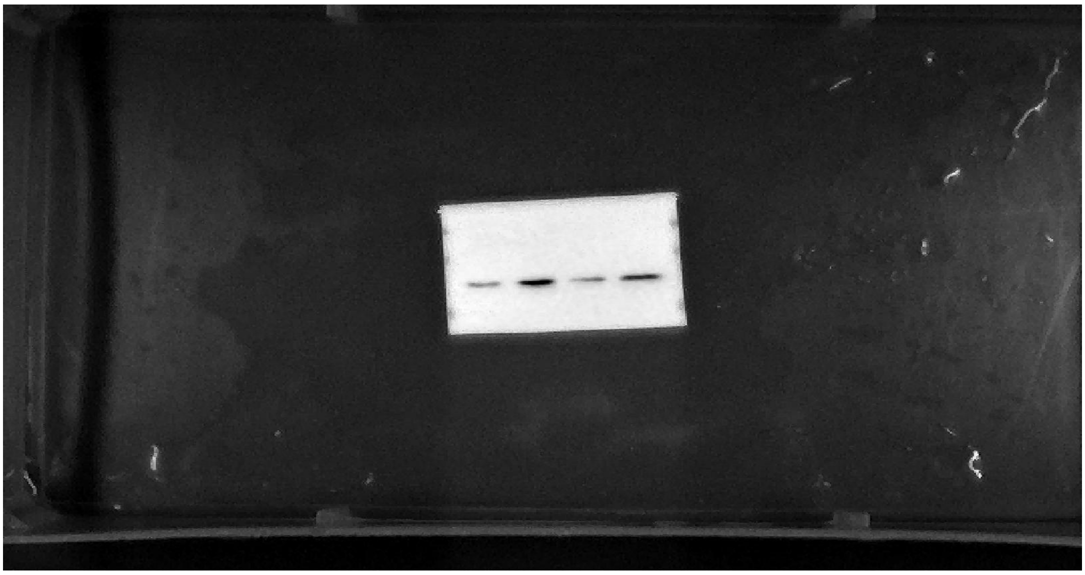

F- Cleaved-C3

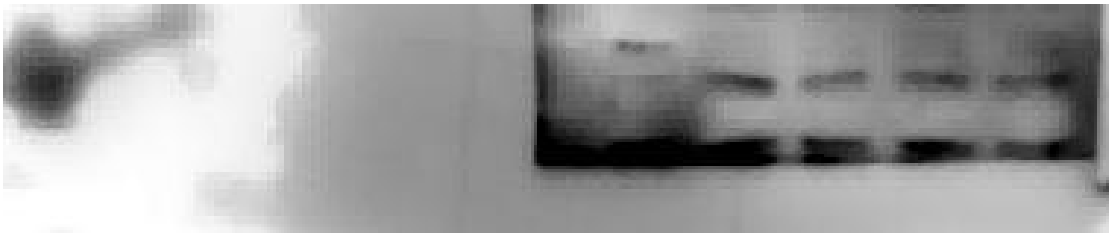

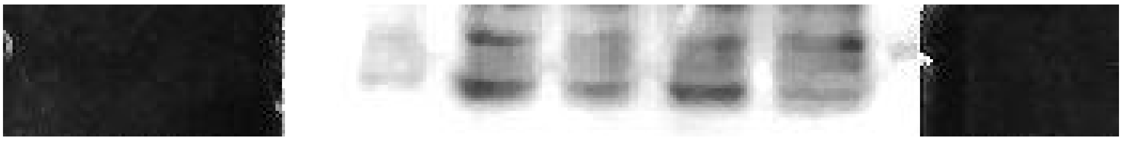

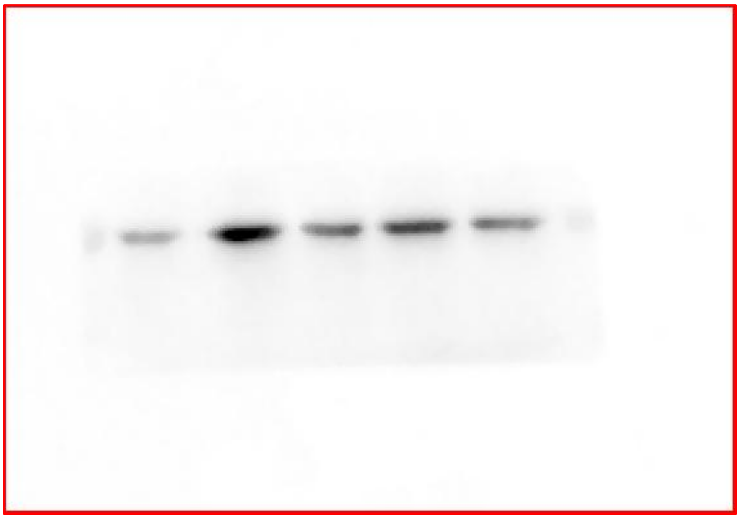

G

Con

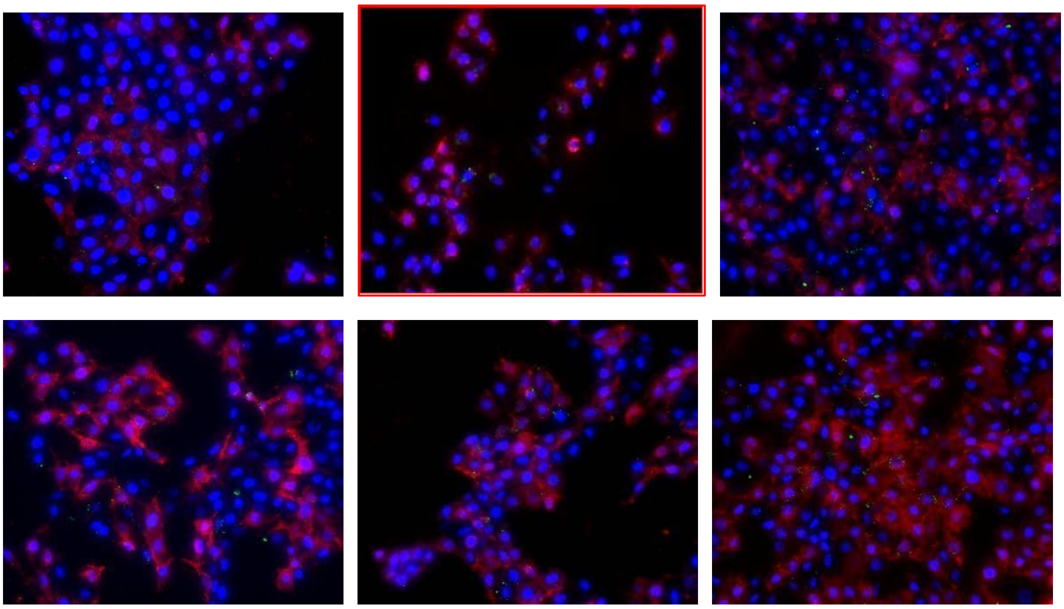

Glu group

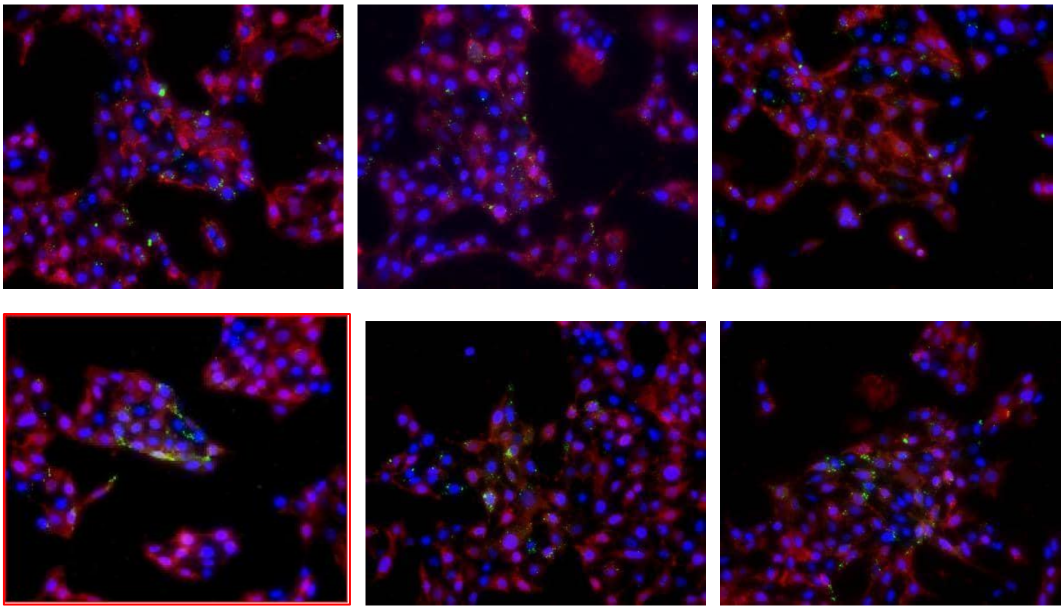

Glu + mimic

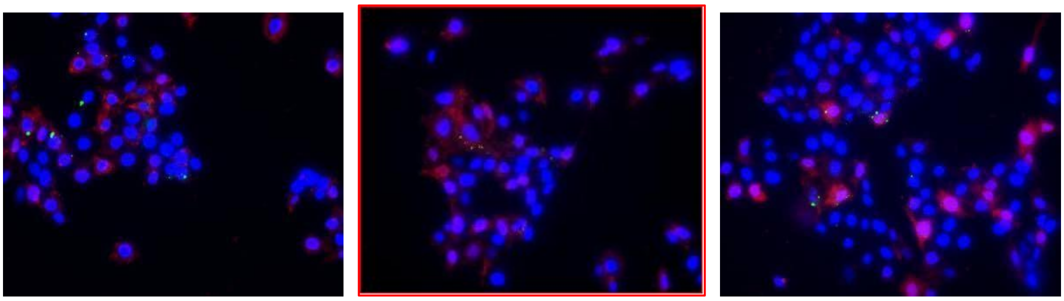

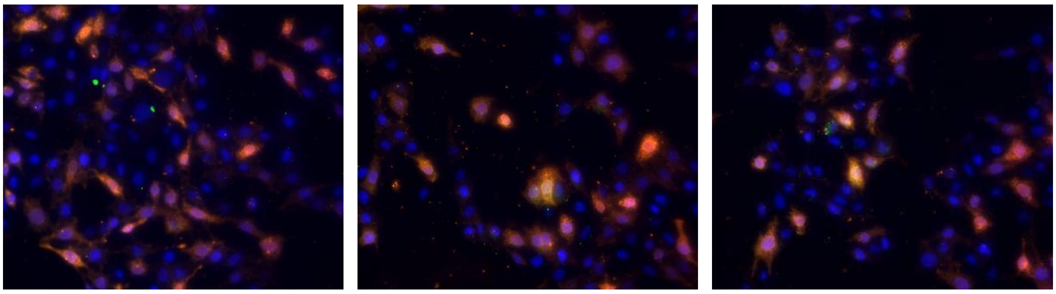

Glu + mimic NC

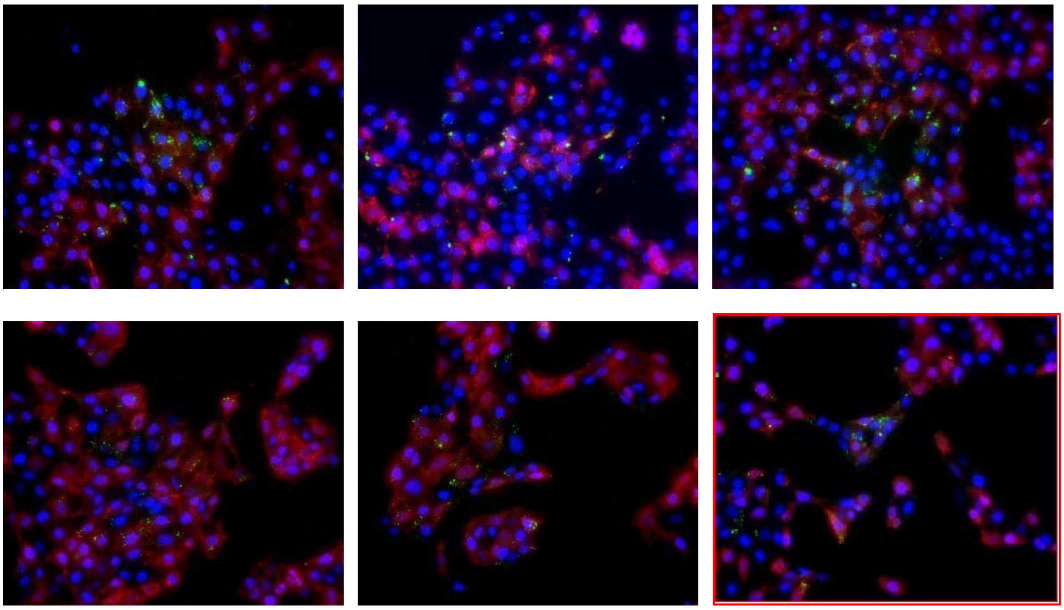

Glu + EVs

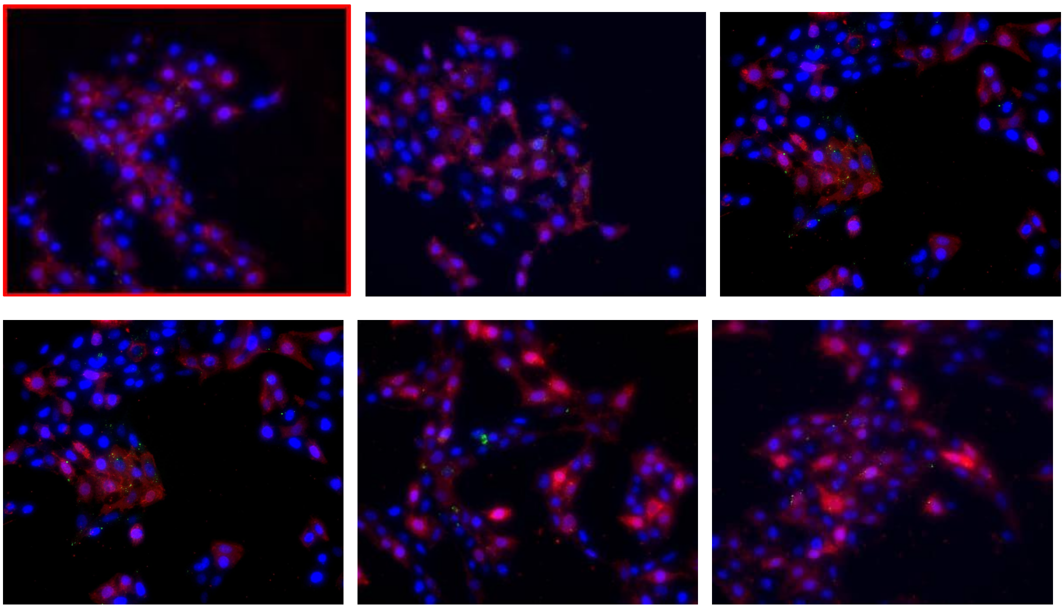

Glu + EVs + inhibitor

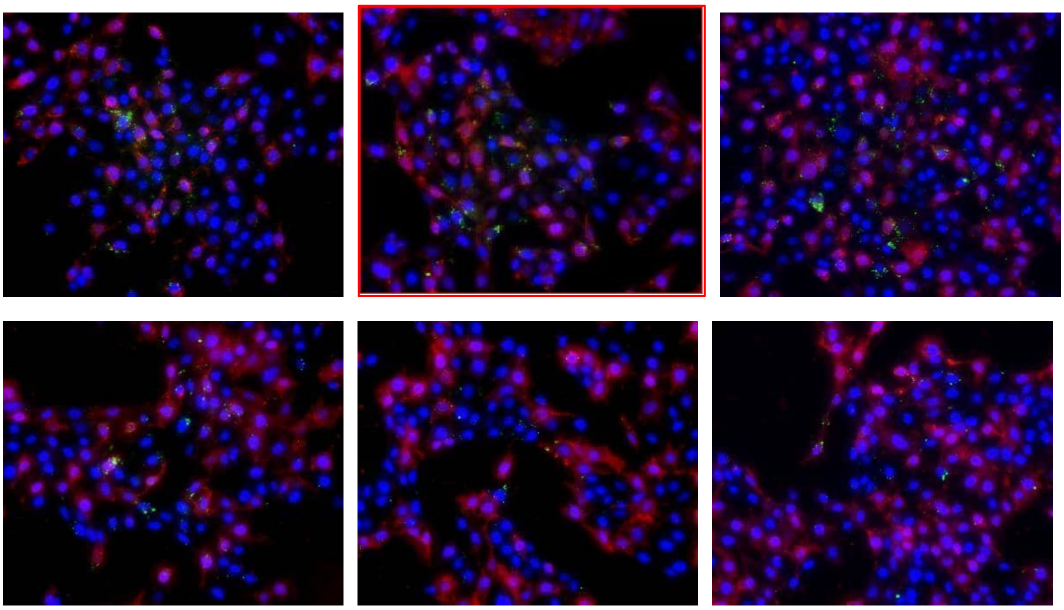

Glu + EVs + inhibitor NC

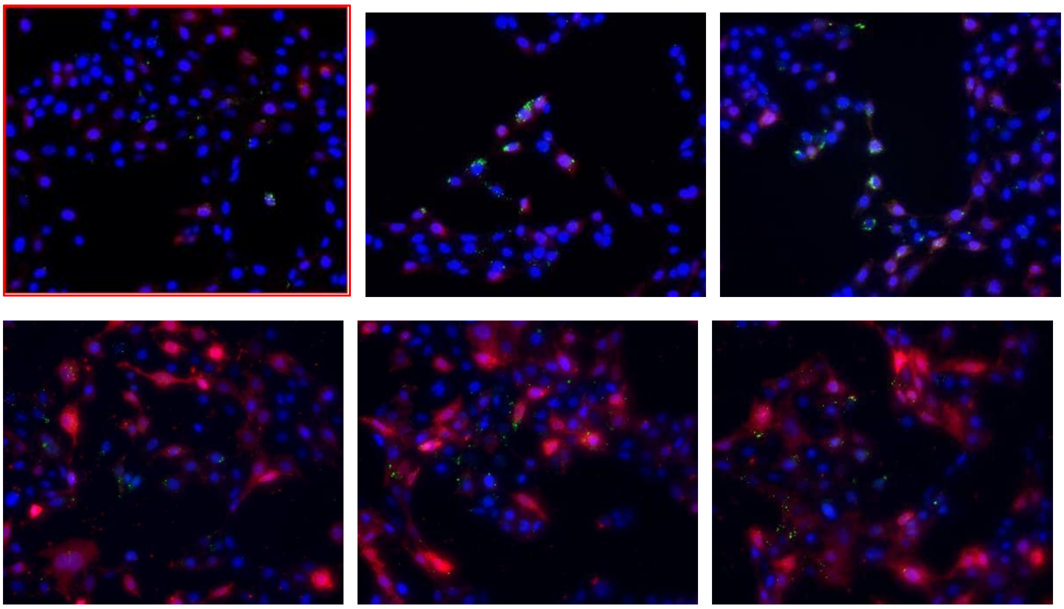

Fig 5

A

P-GluA2

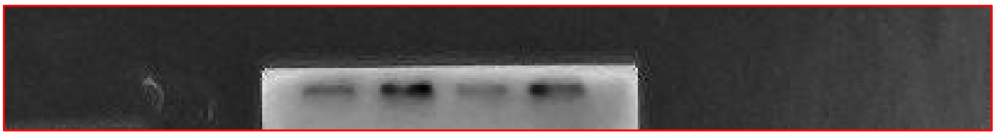

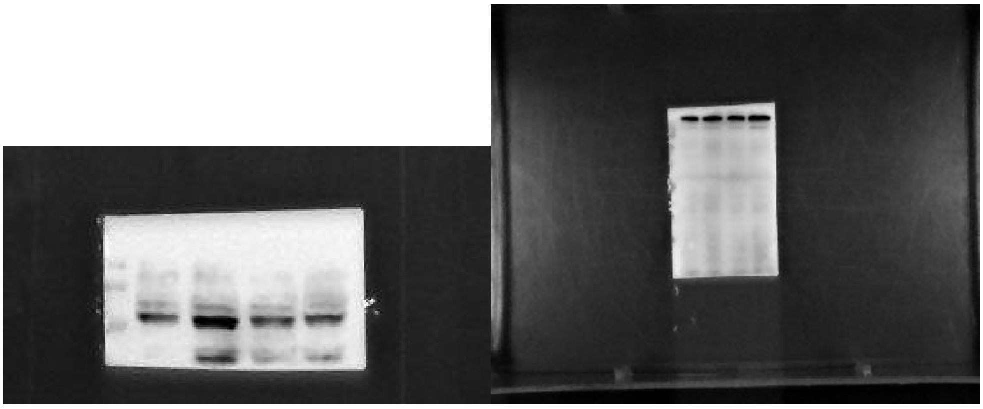

GluA2

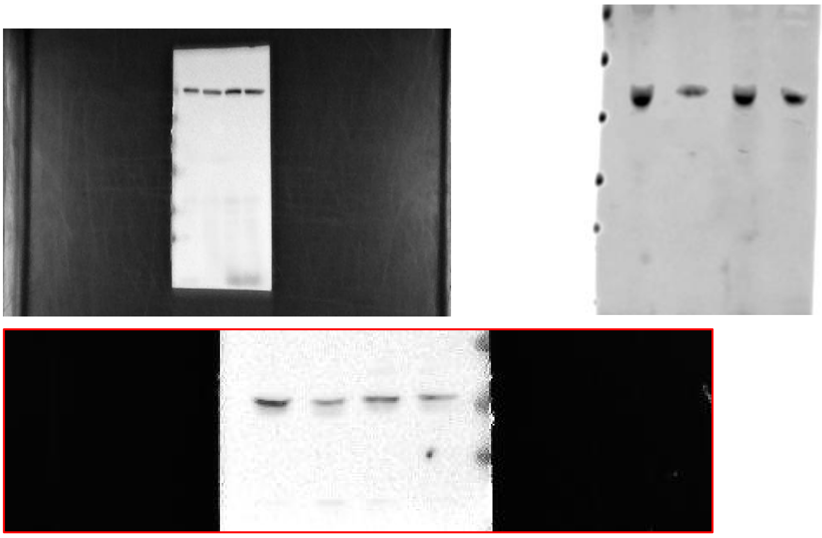

p-PKCA

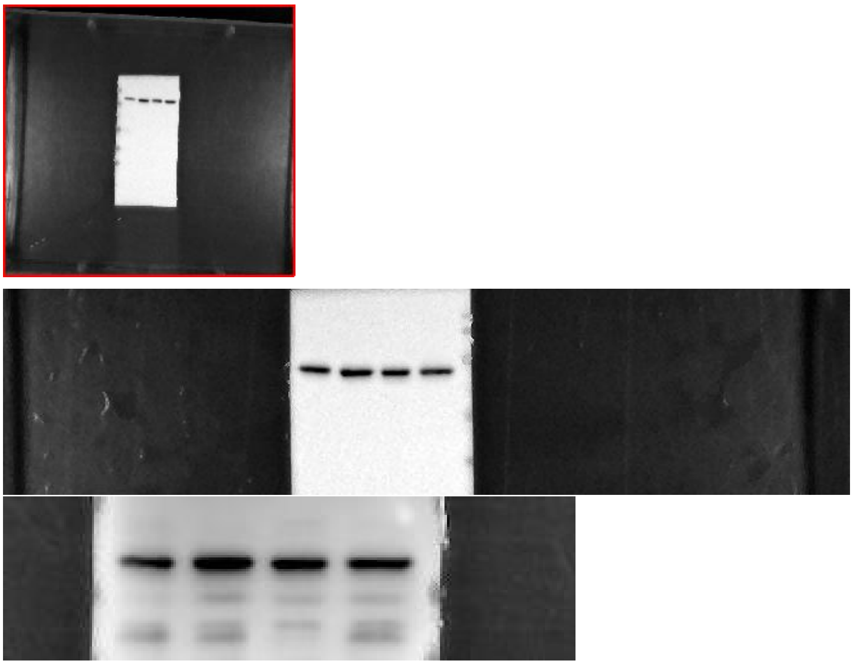

PKCA

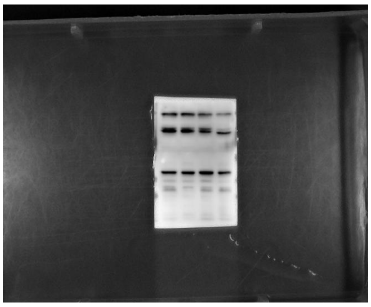

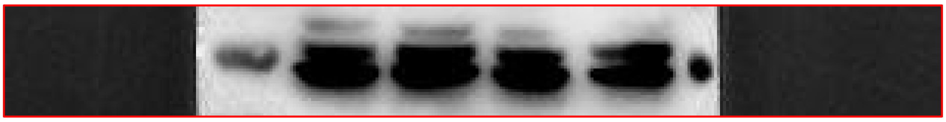

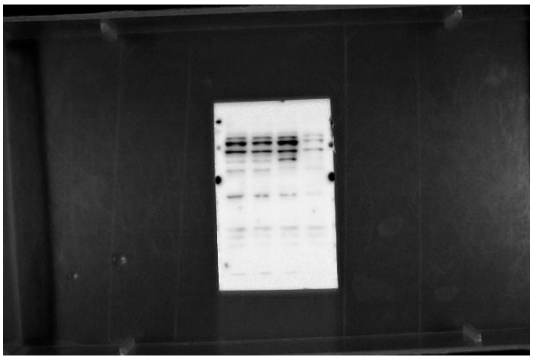

PLCD1

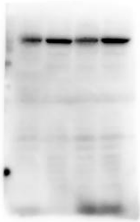

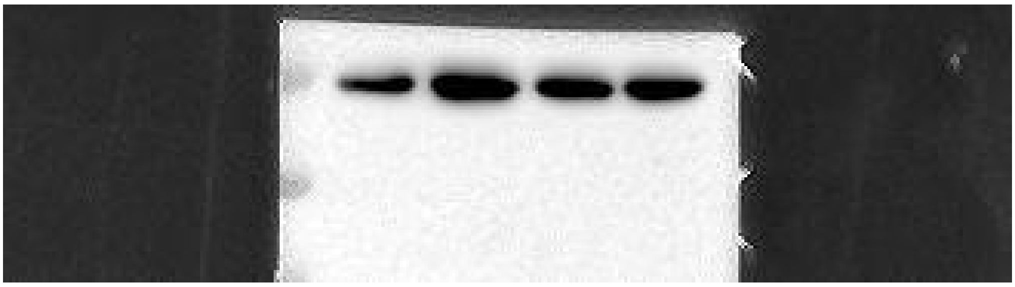

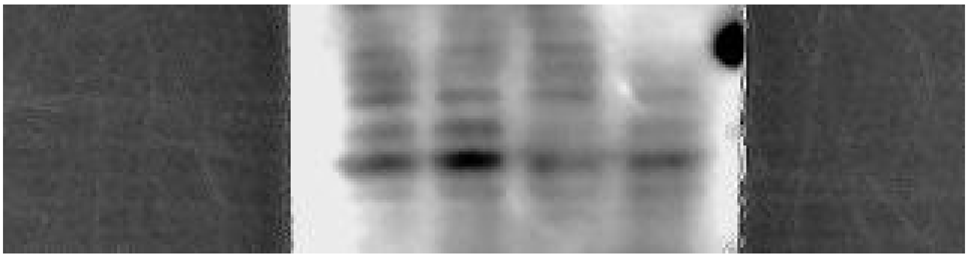

Beta-Tubulin

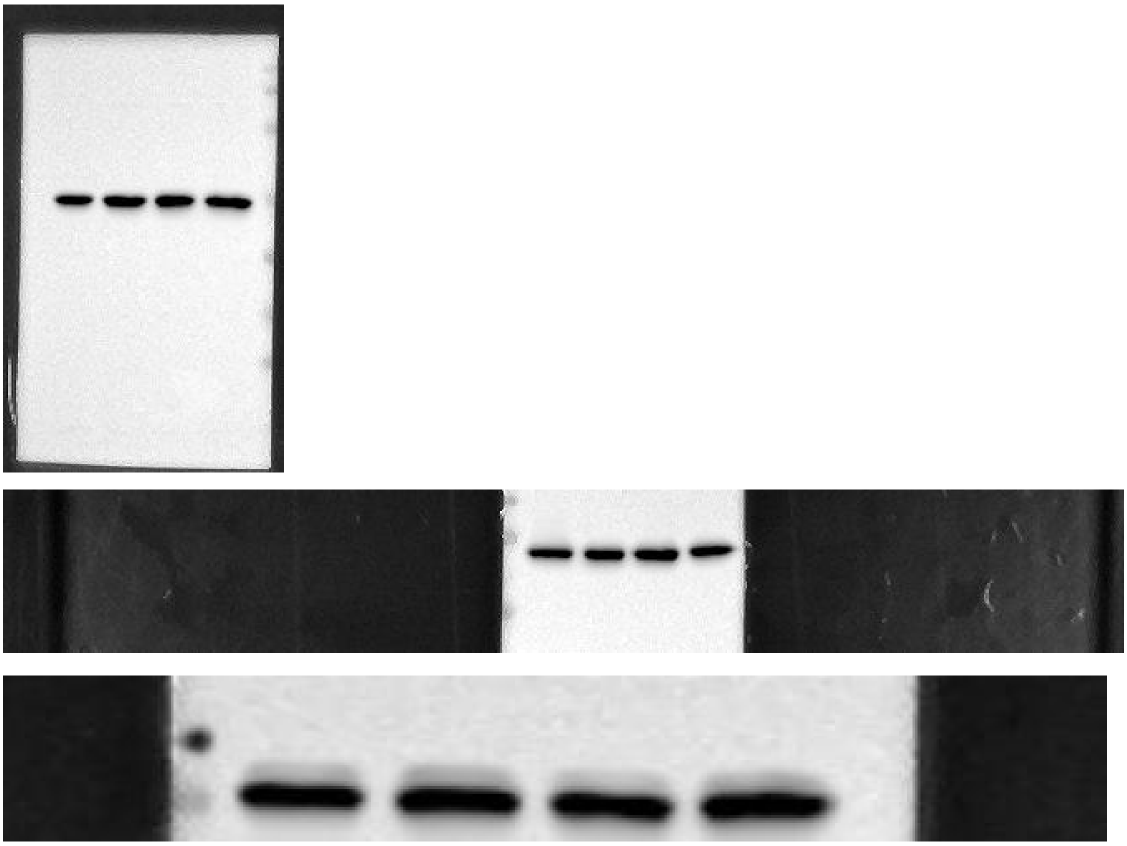

E

P-GluA2

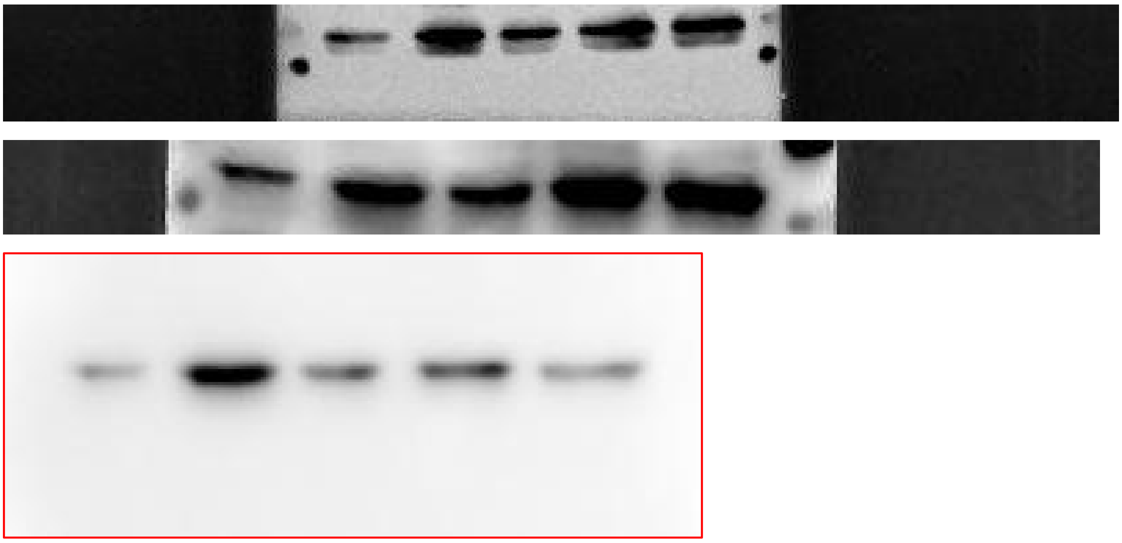

GluA2

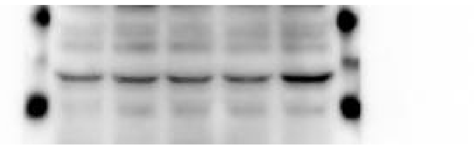

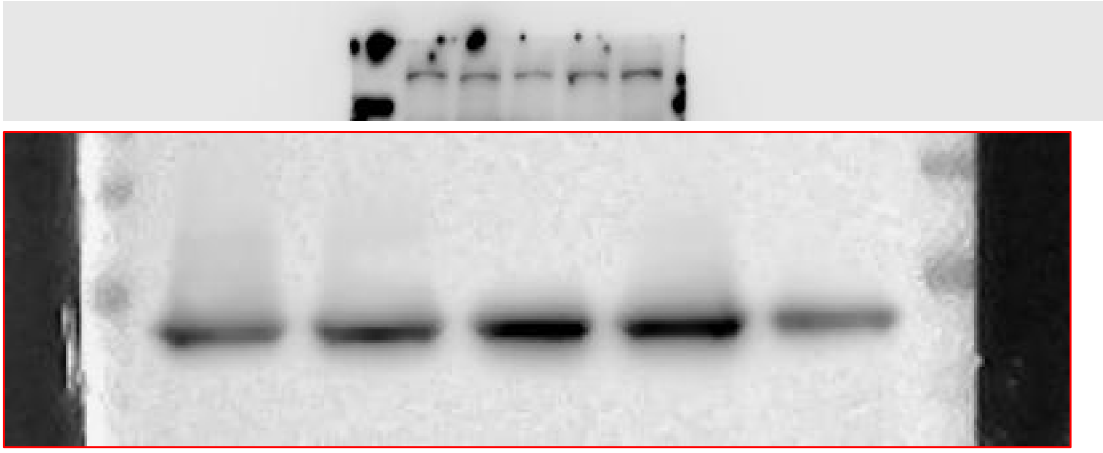

P-PKCA

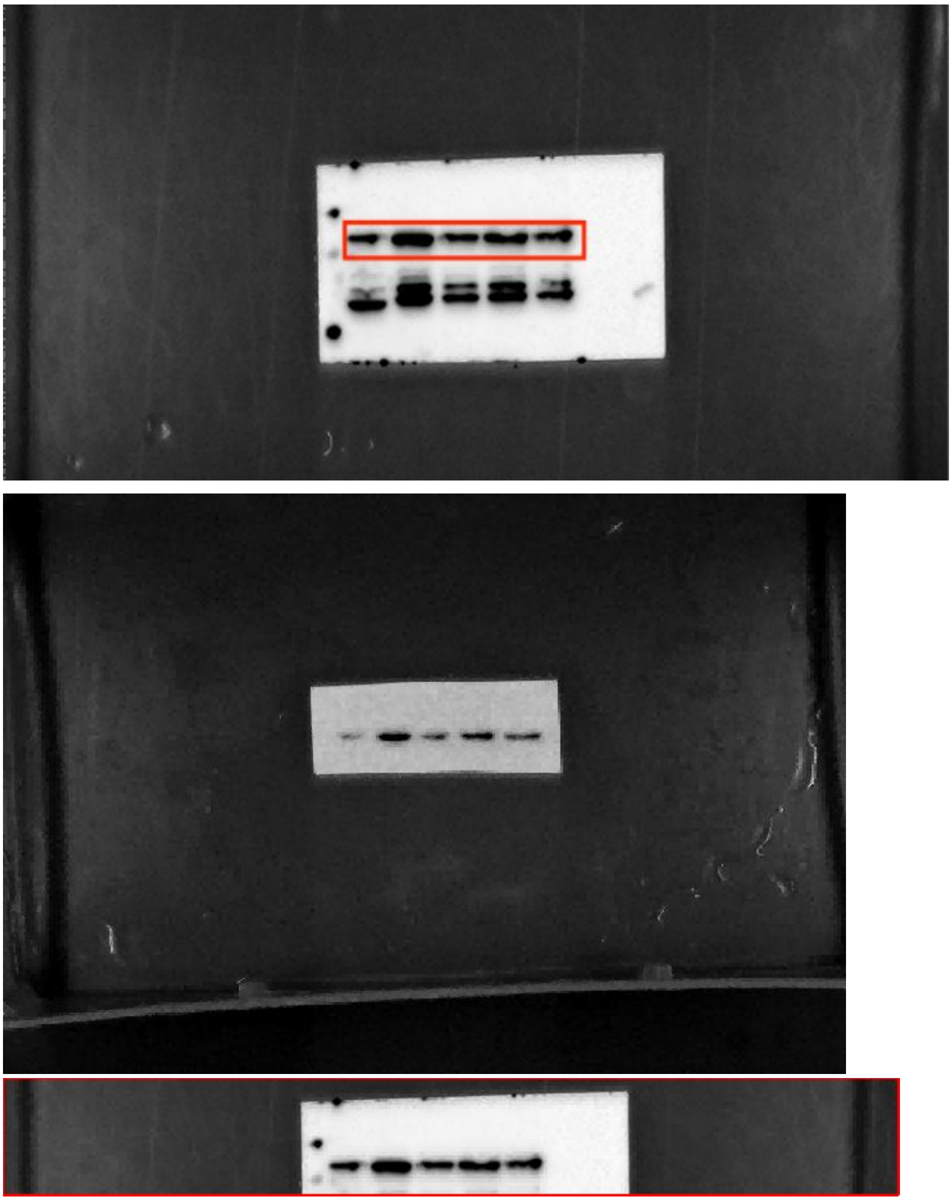

PKCA

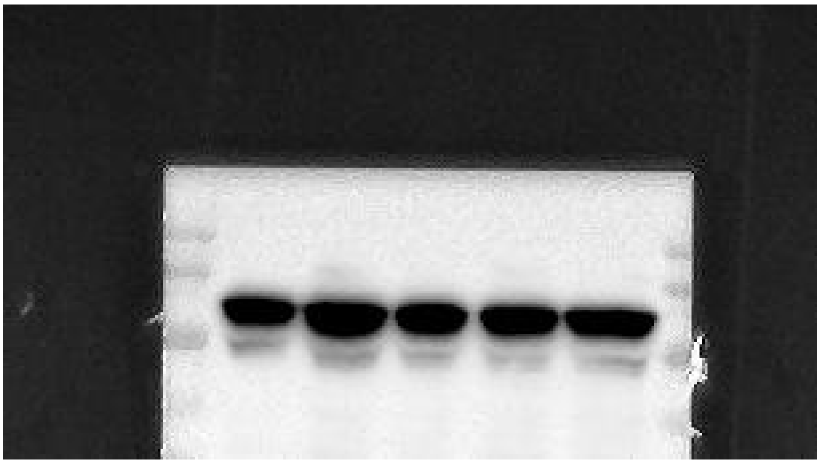

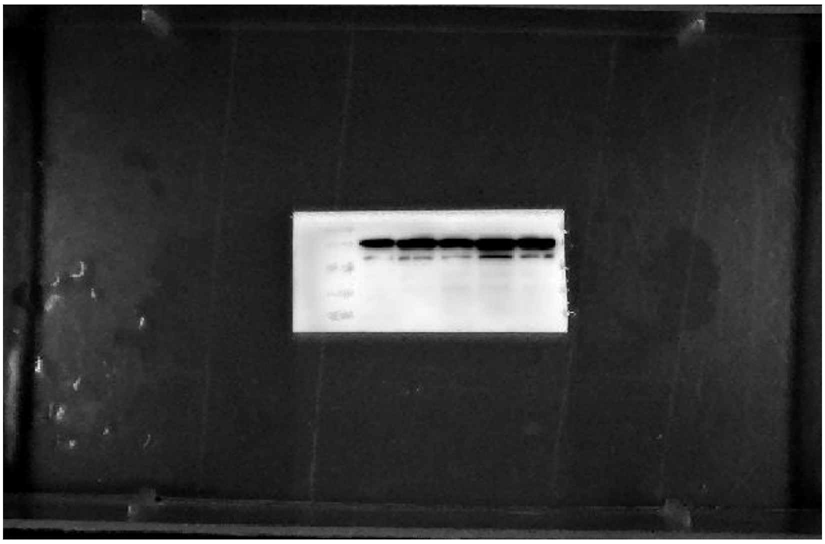

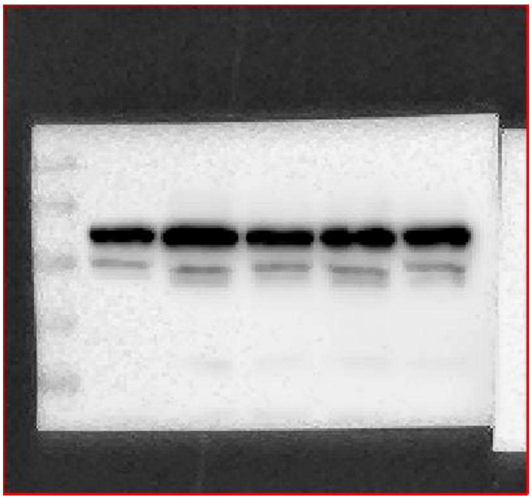

PLCD1

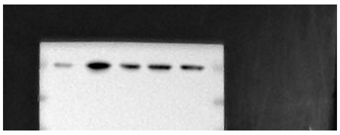

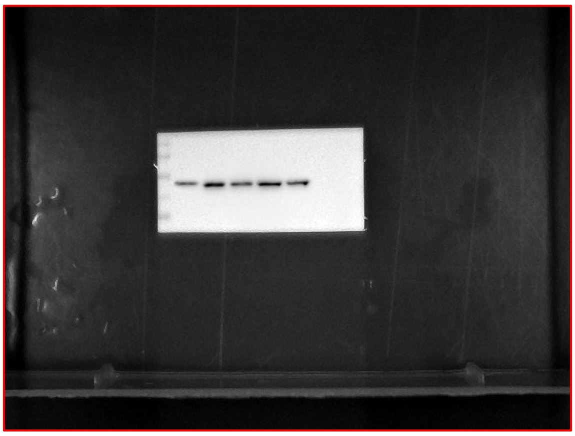

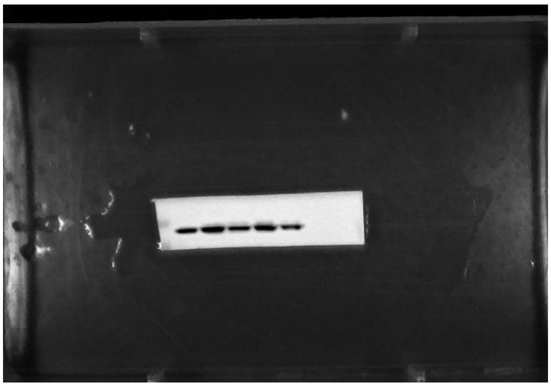

Beta-tubulin

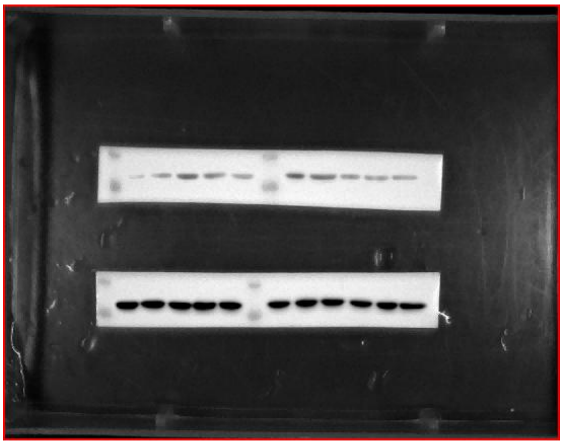

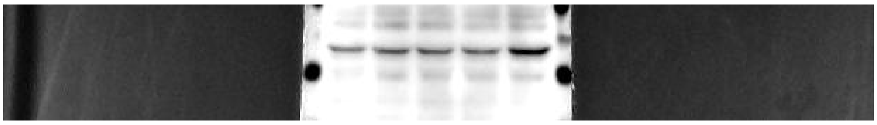

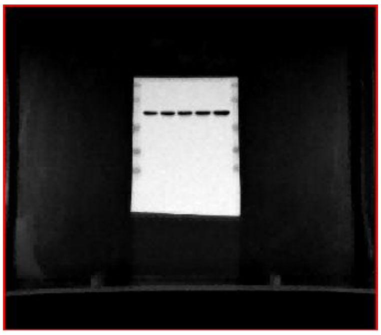

I

Con

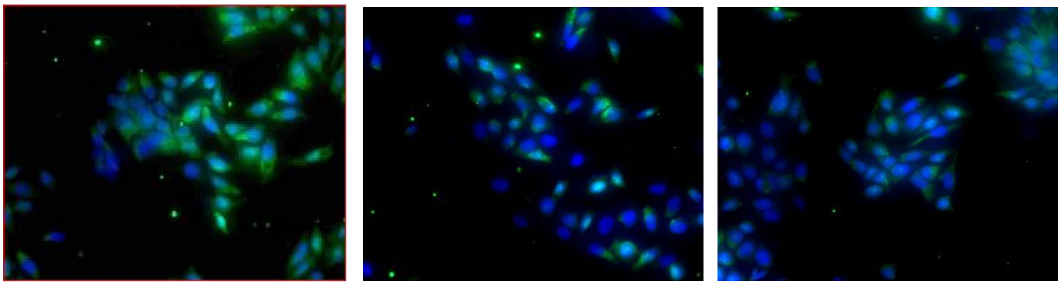

Glu

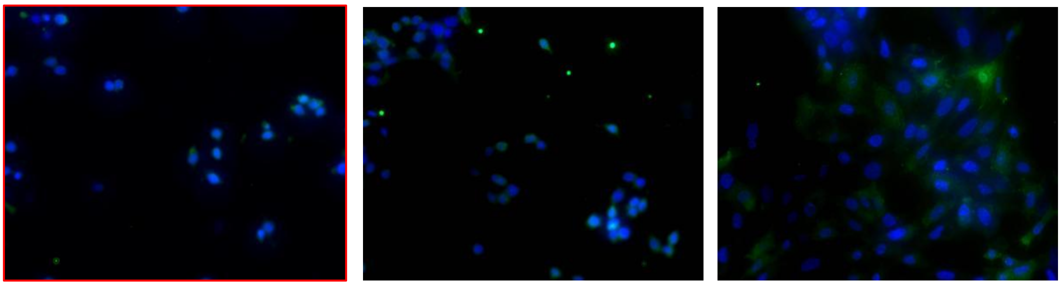

Glu + mimic

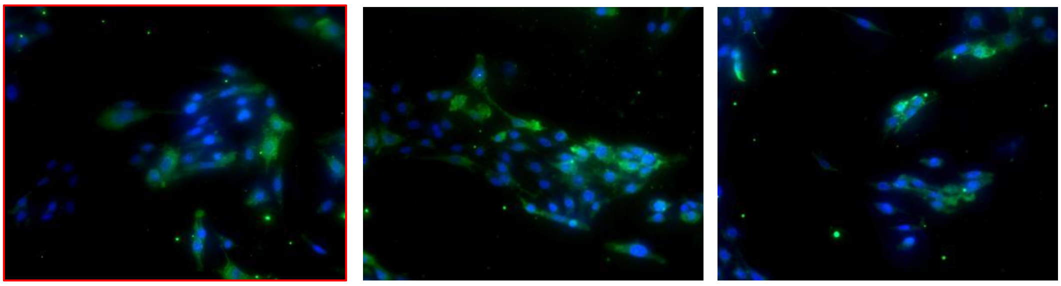

Glu+mimic-NC

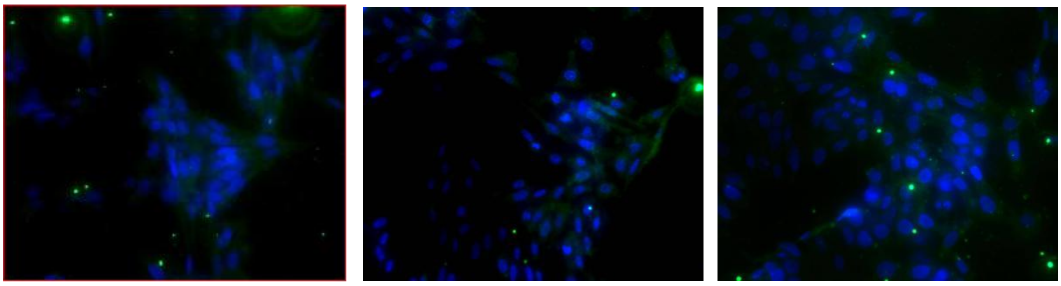

Glu+EVs

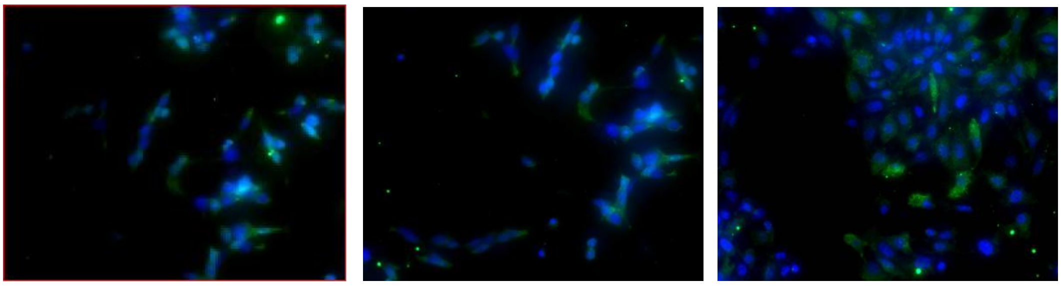

Glu+EVs+Inhibitor

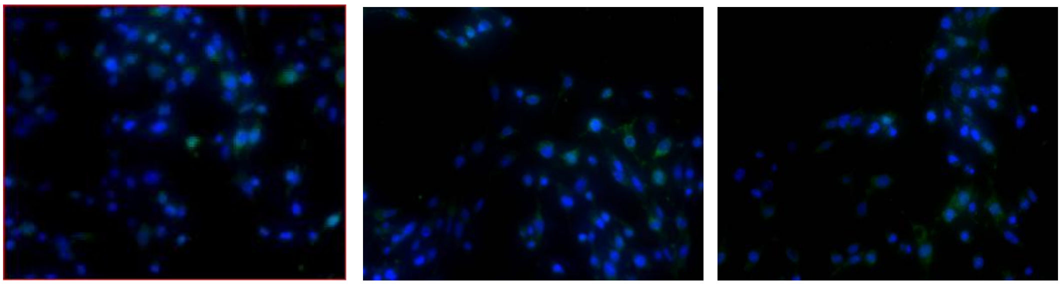

Glu+EVs+Inhibitor-NC

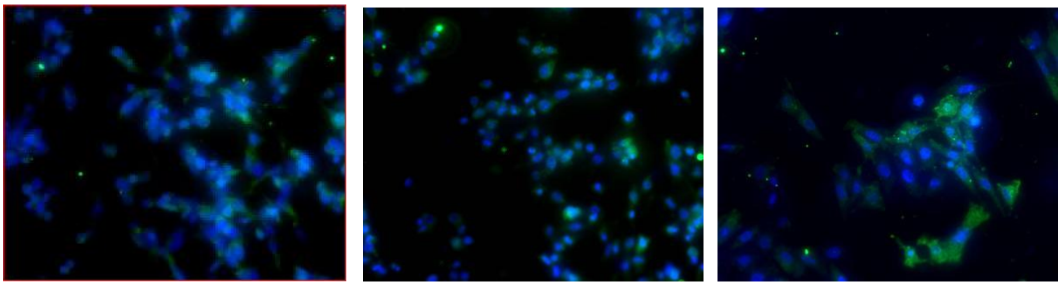

Fig 6

A

Con

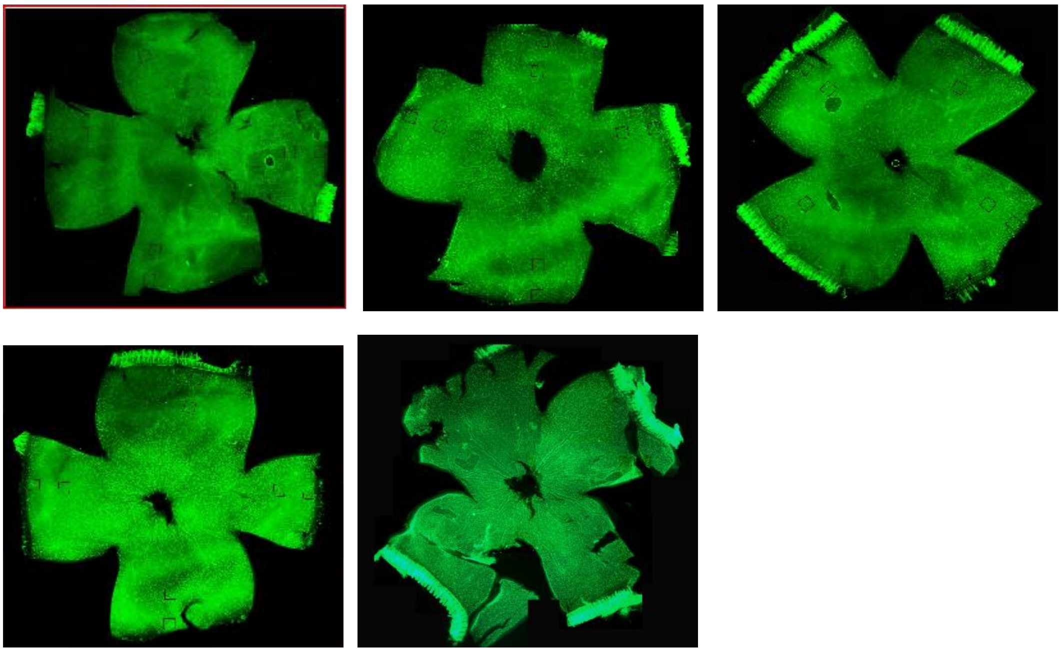

Glu

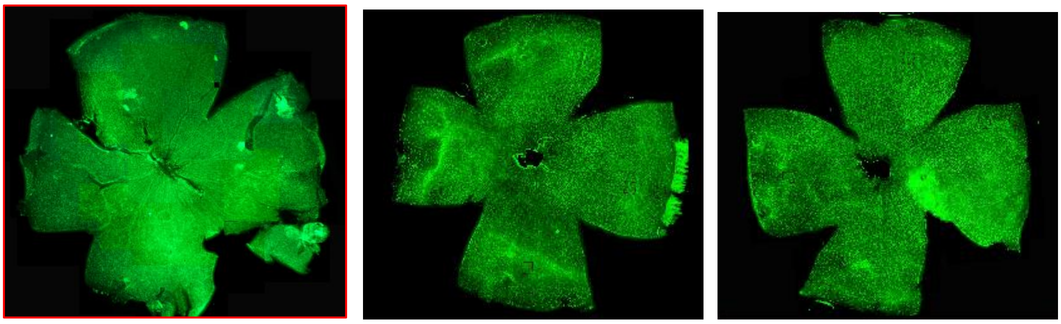

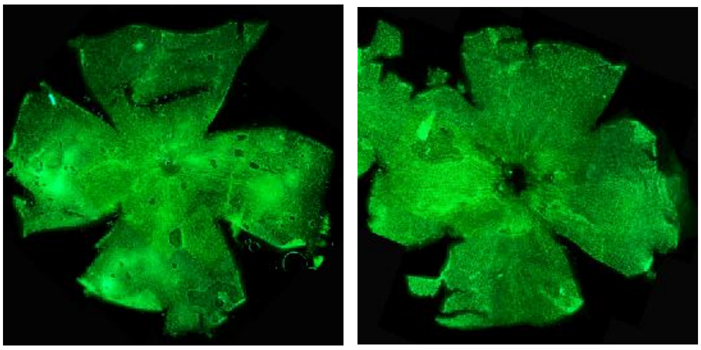

Glu+agomir

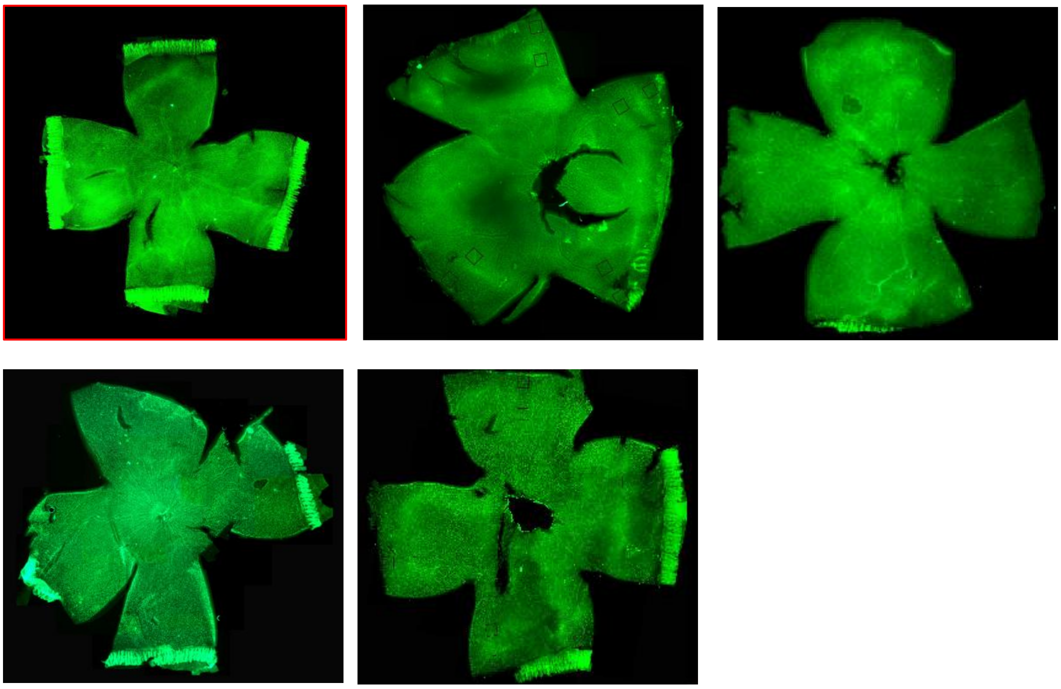

Glu+agomir-NC

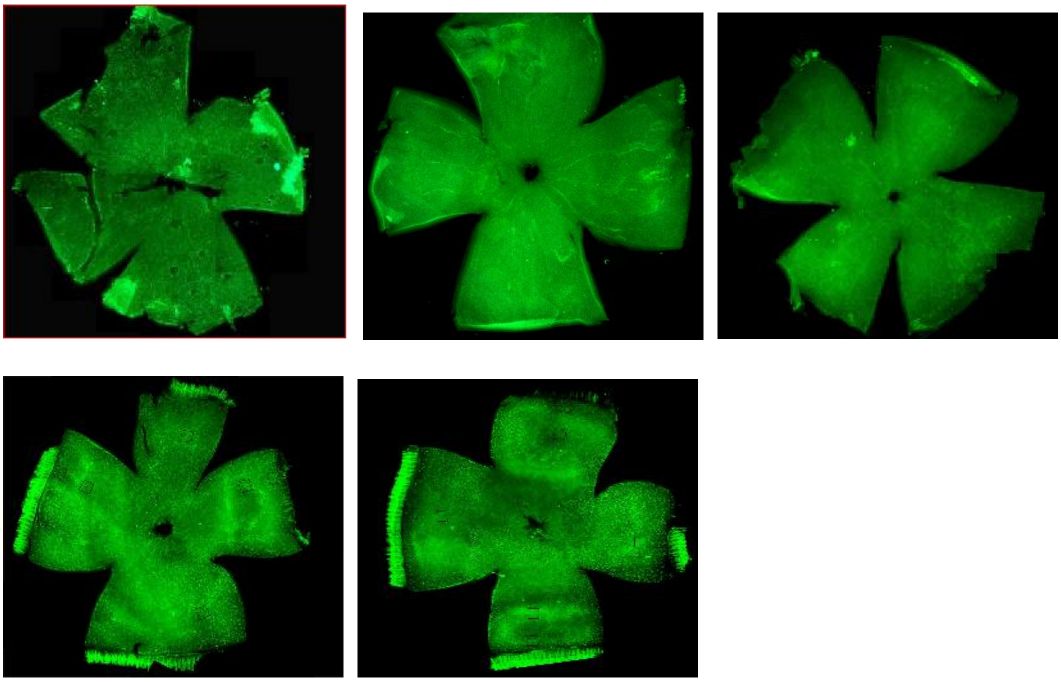

Glu+antagomir

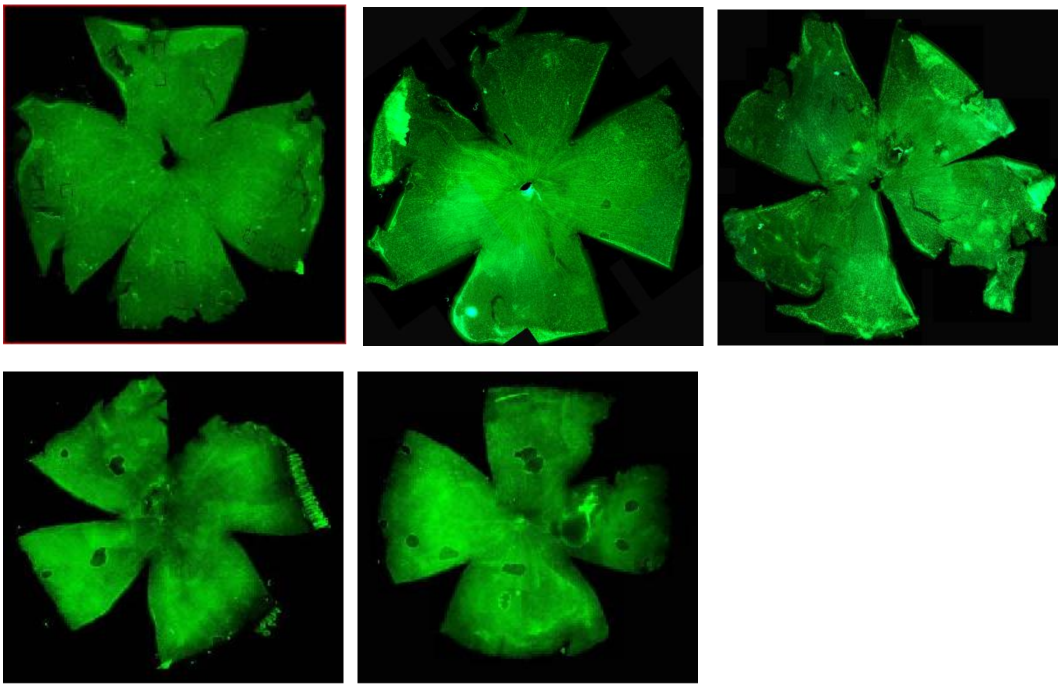

Glu+antagomir-NC

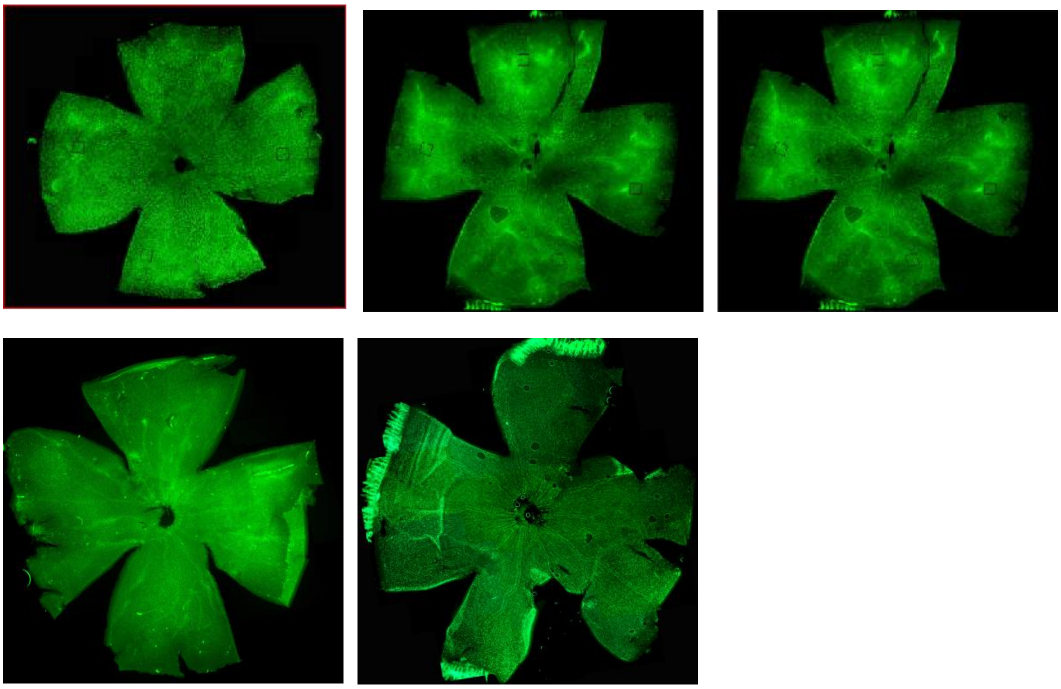

L

CON

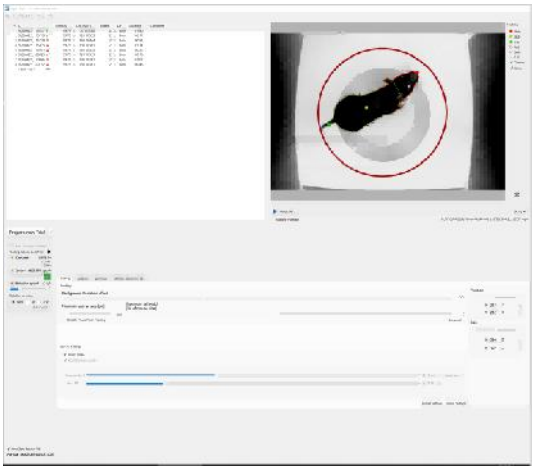

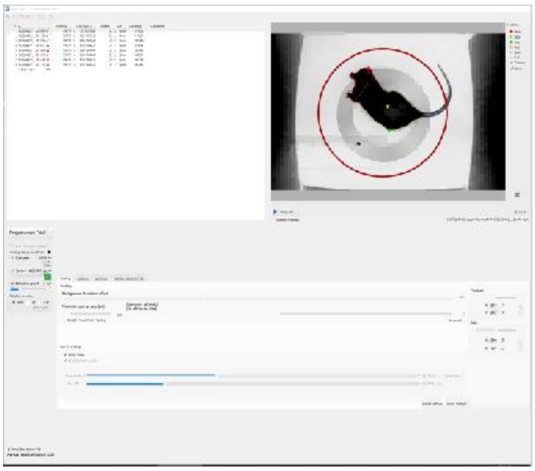

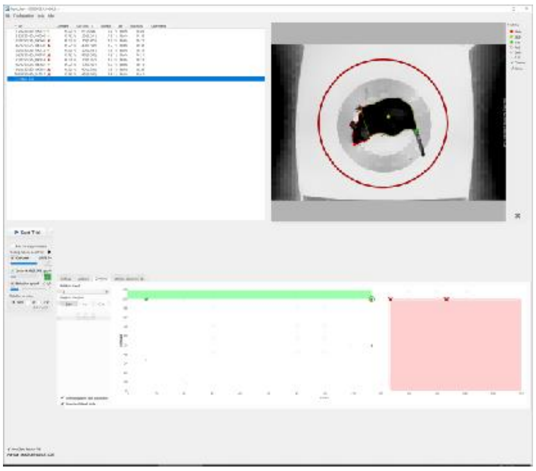

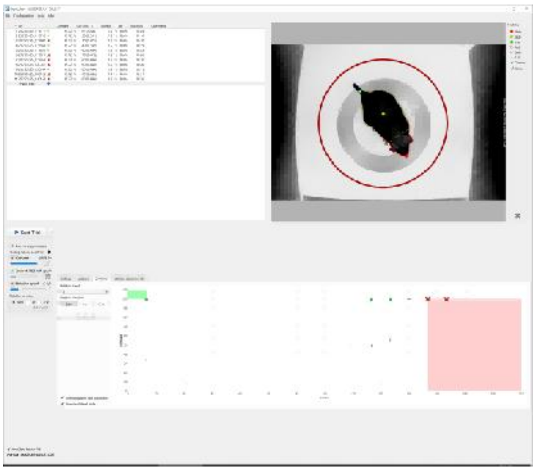

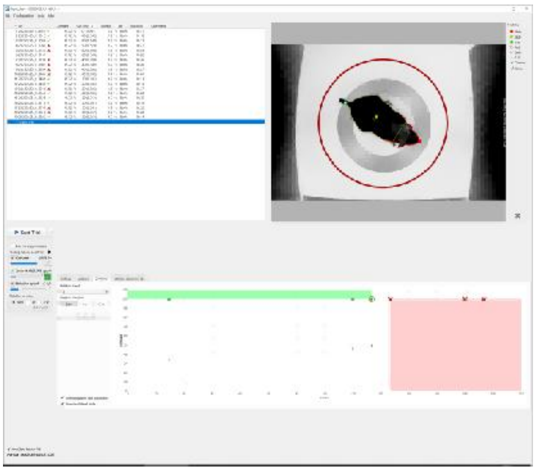

Glu

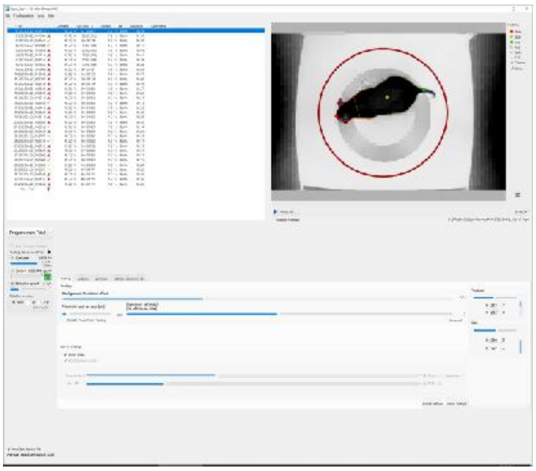

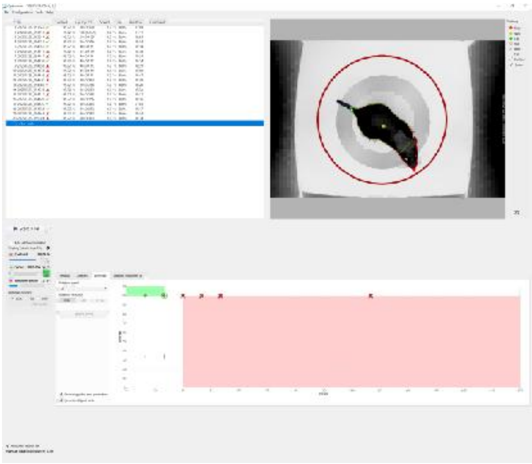

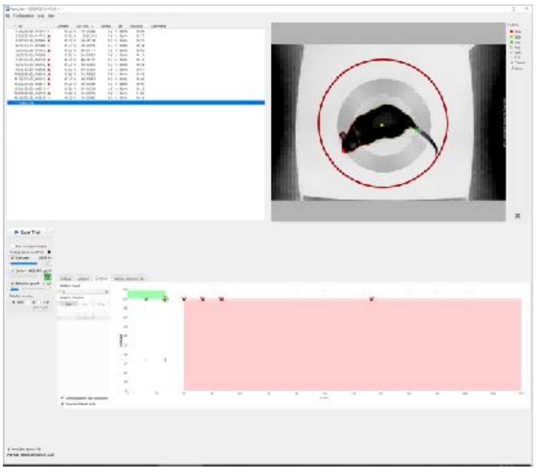

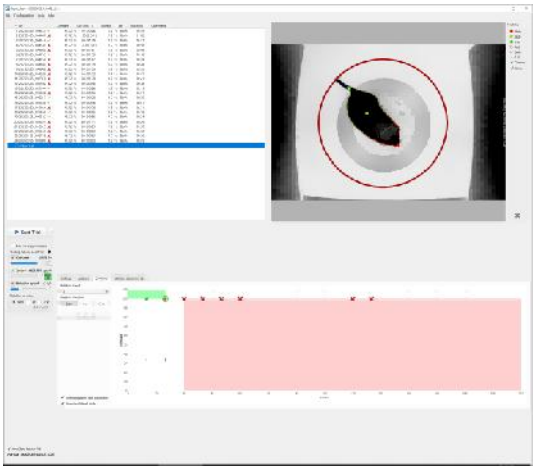

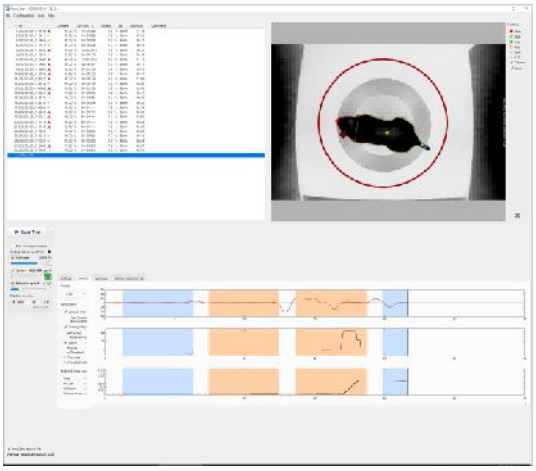

G+agomir

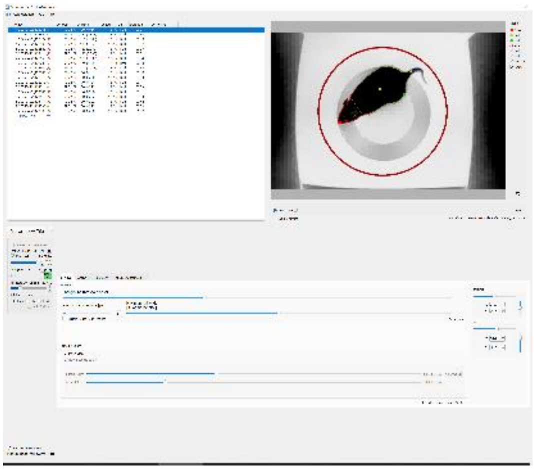

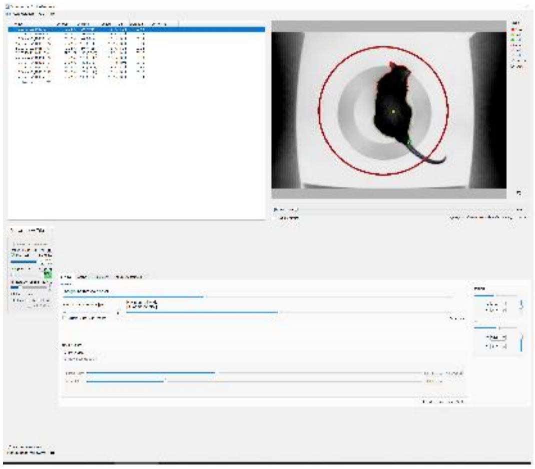

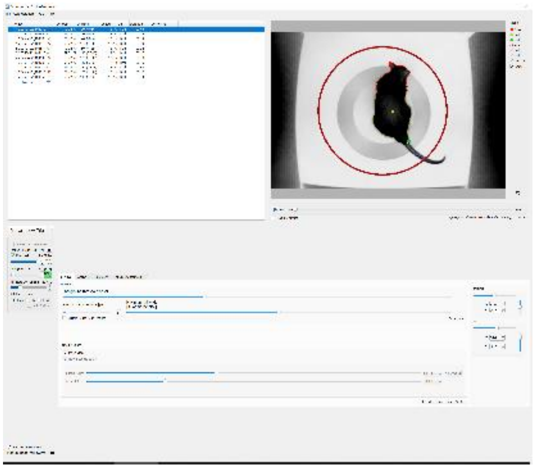

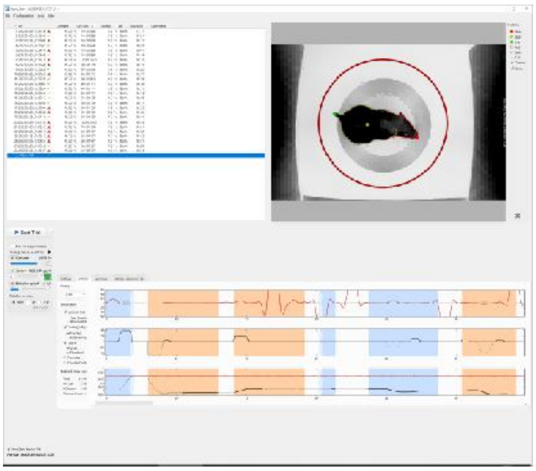

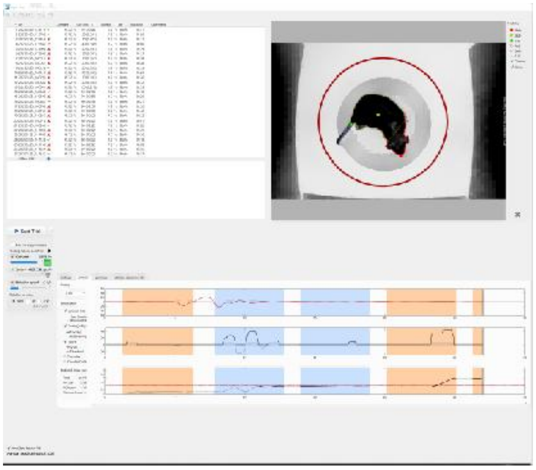

G+agomir-NC

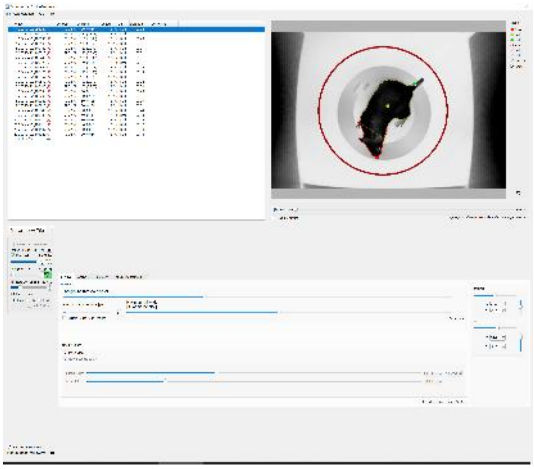

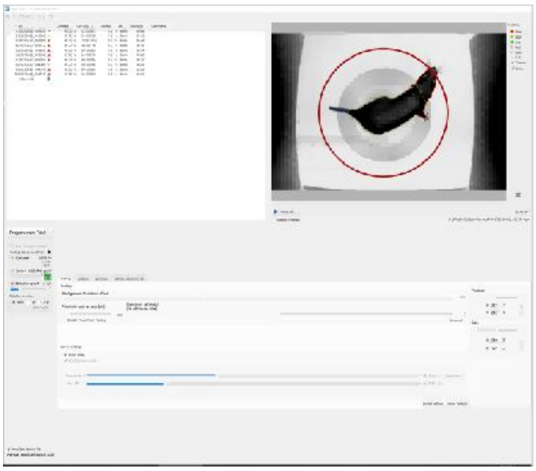

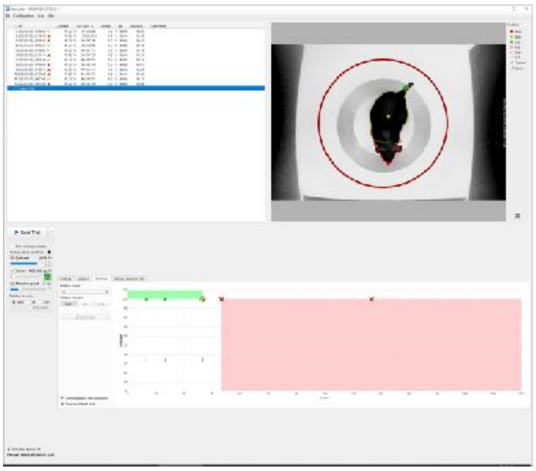

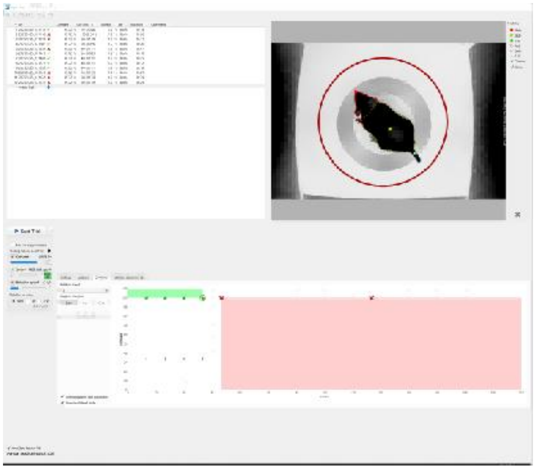

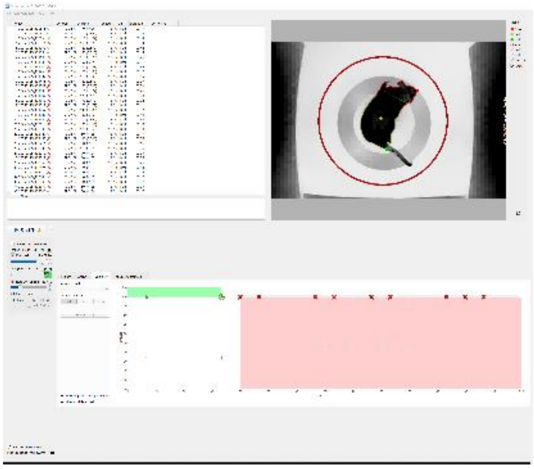

G+antagomir

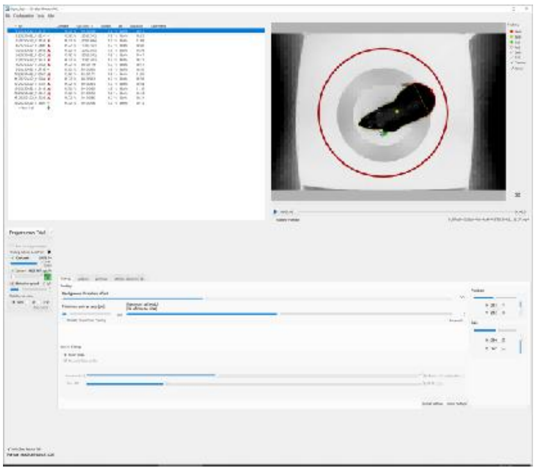

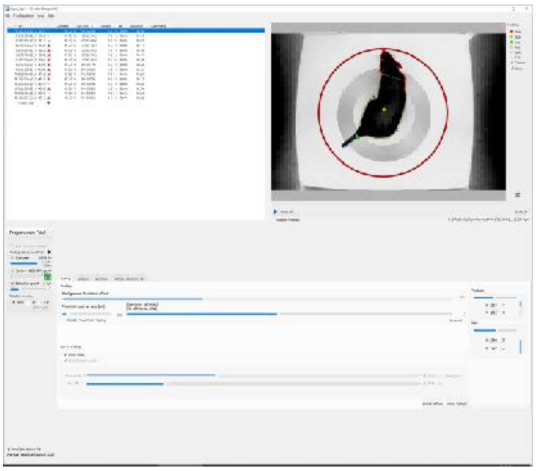

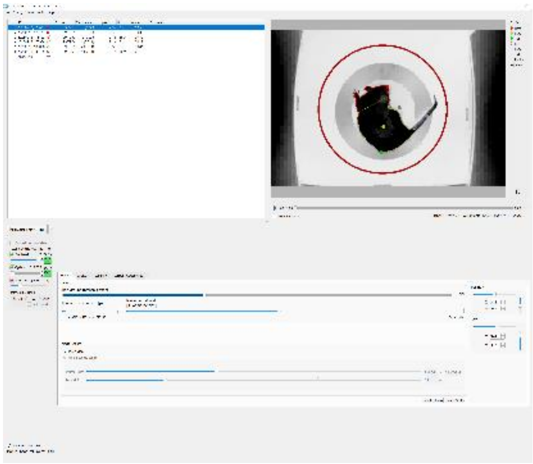

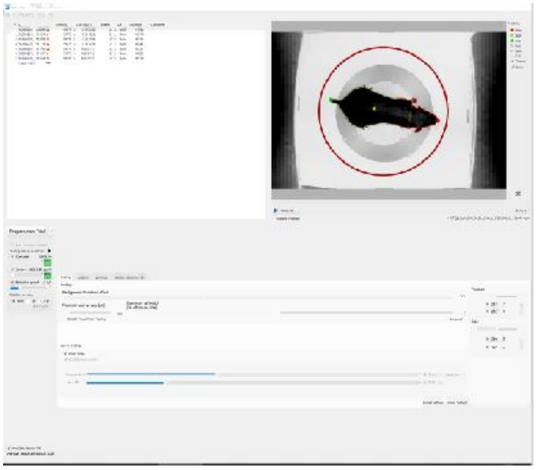

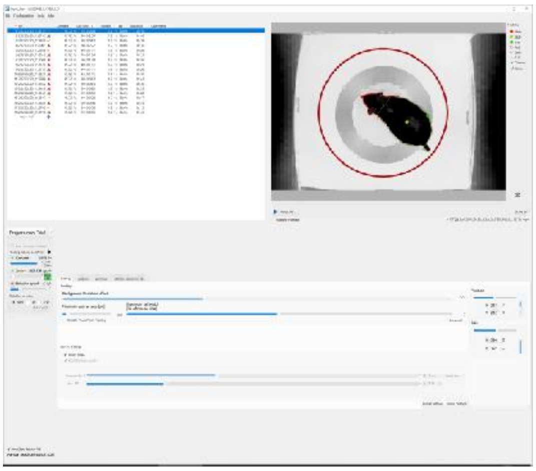

Glu+antagomir-NC

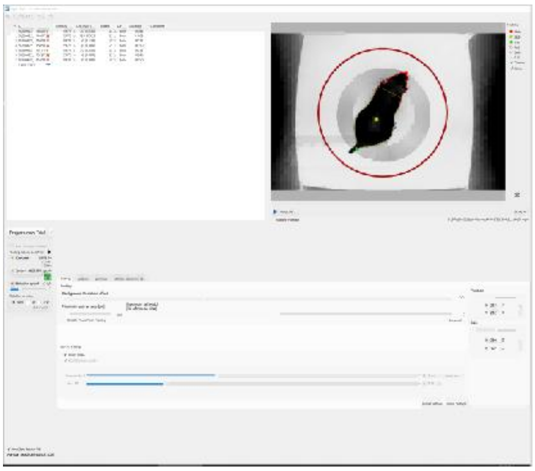

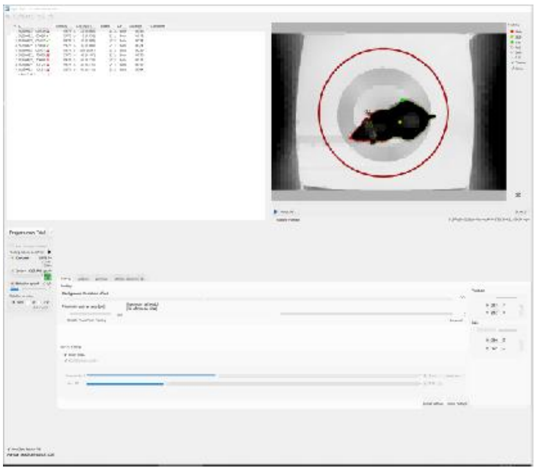

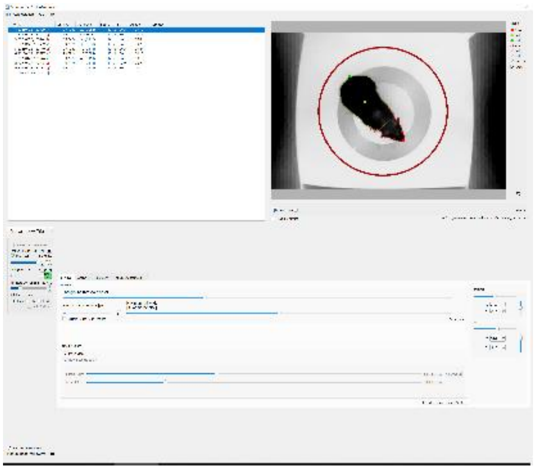

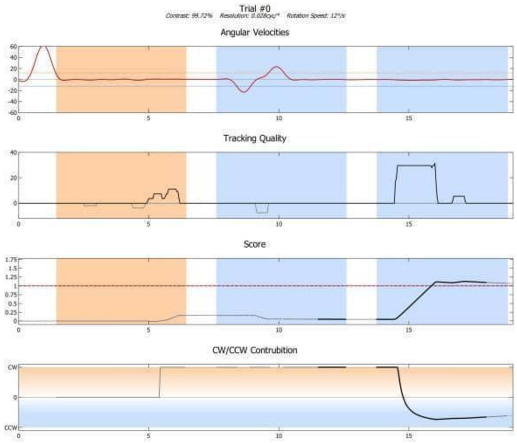

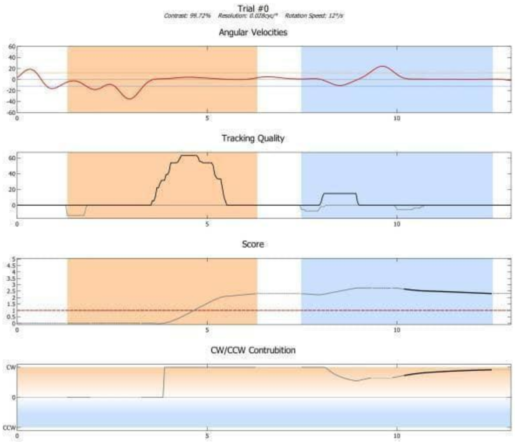

Fig 7

A

P-GluA2

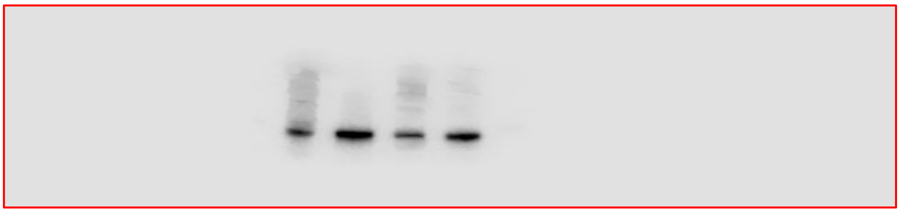

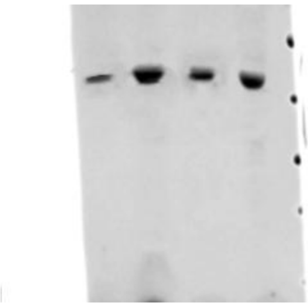

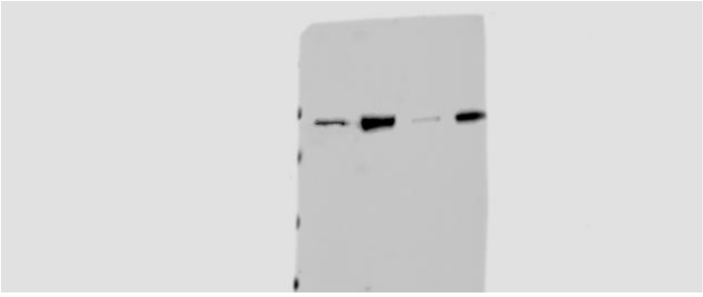

GluA2

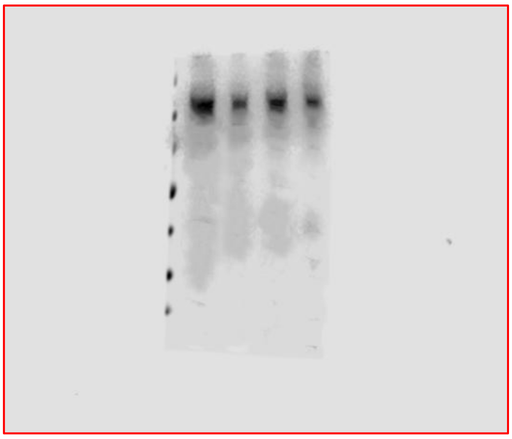

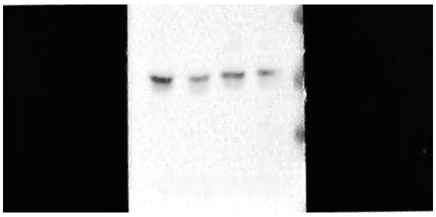

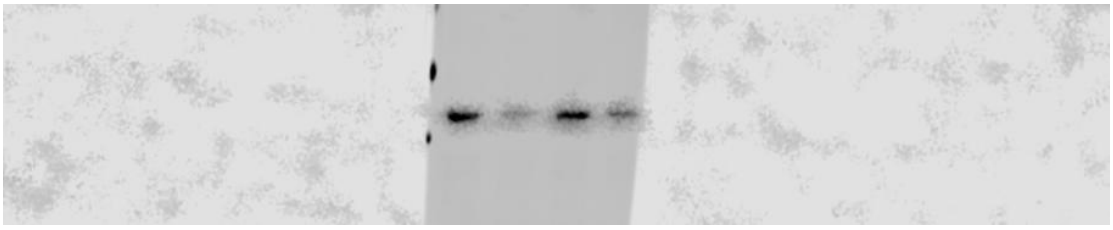

p-PKCA

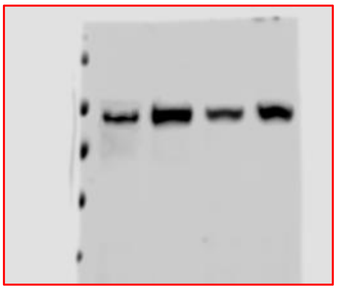

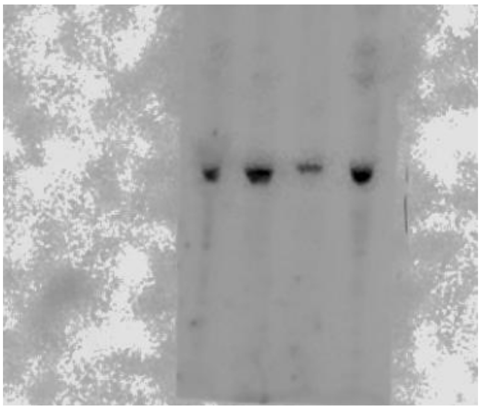

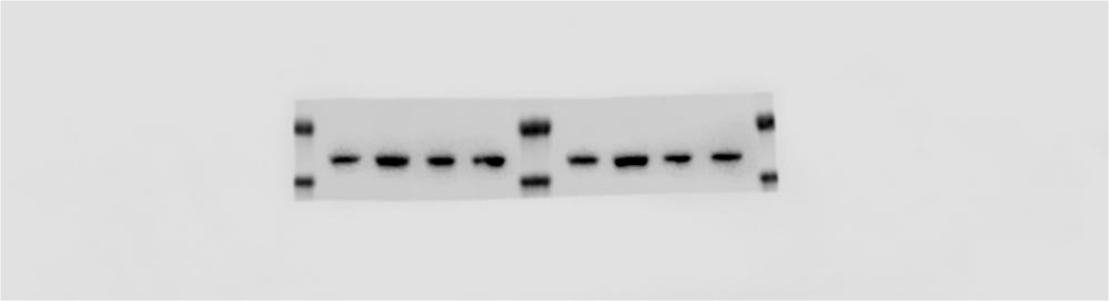

PKCA

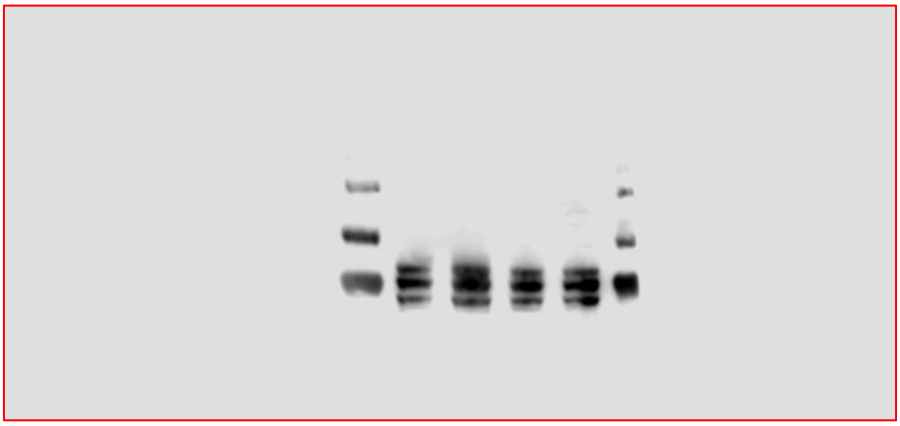

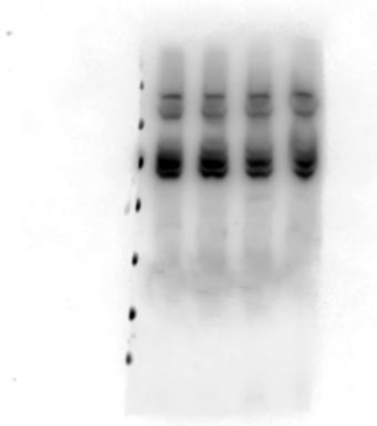

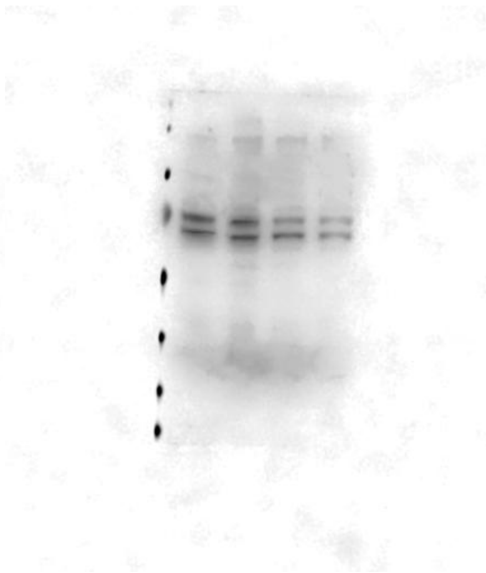

PLCD1

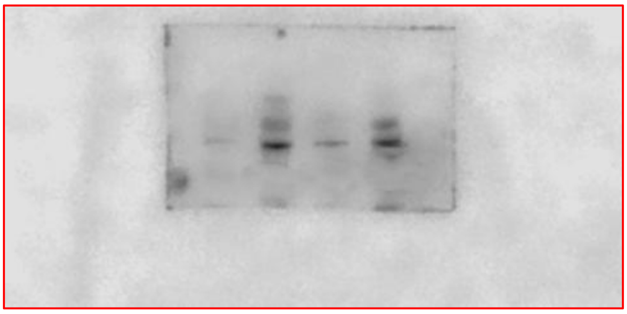

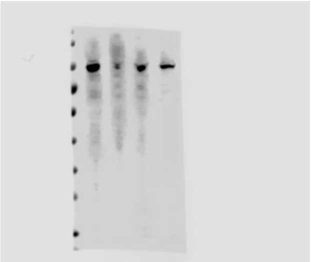

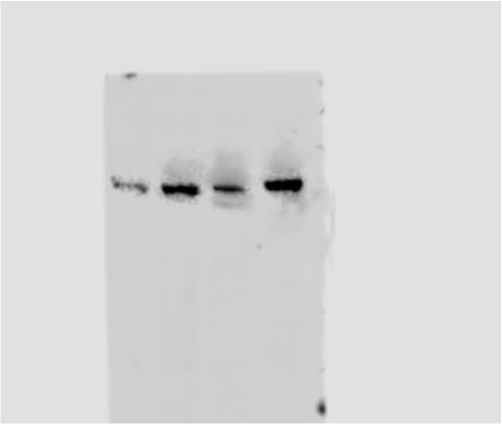

Beta-tubulin

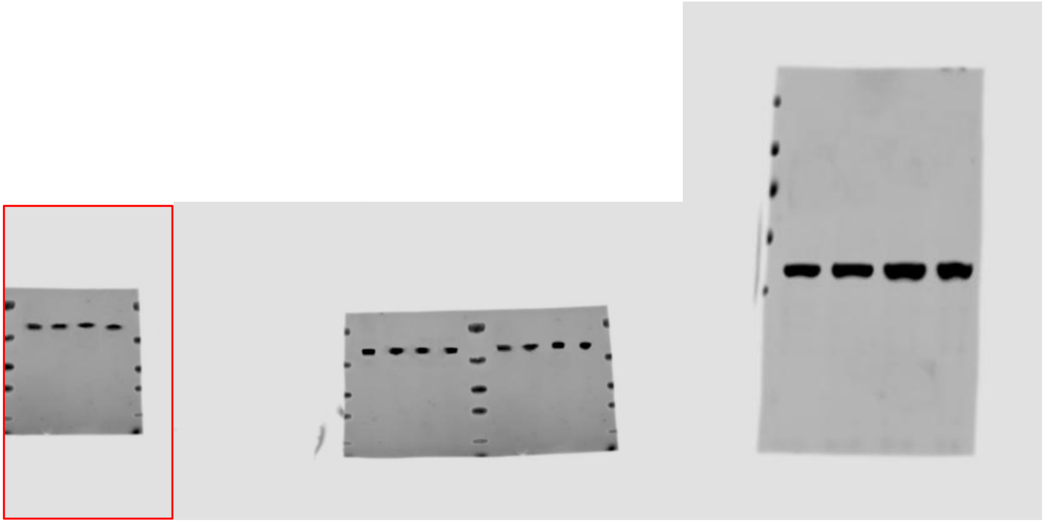

E

P-GluA2

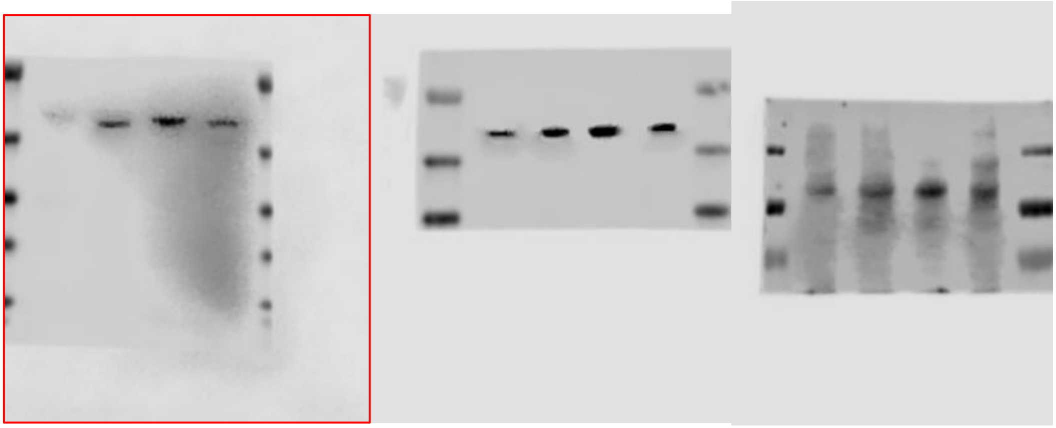

GluA2

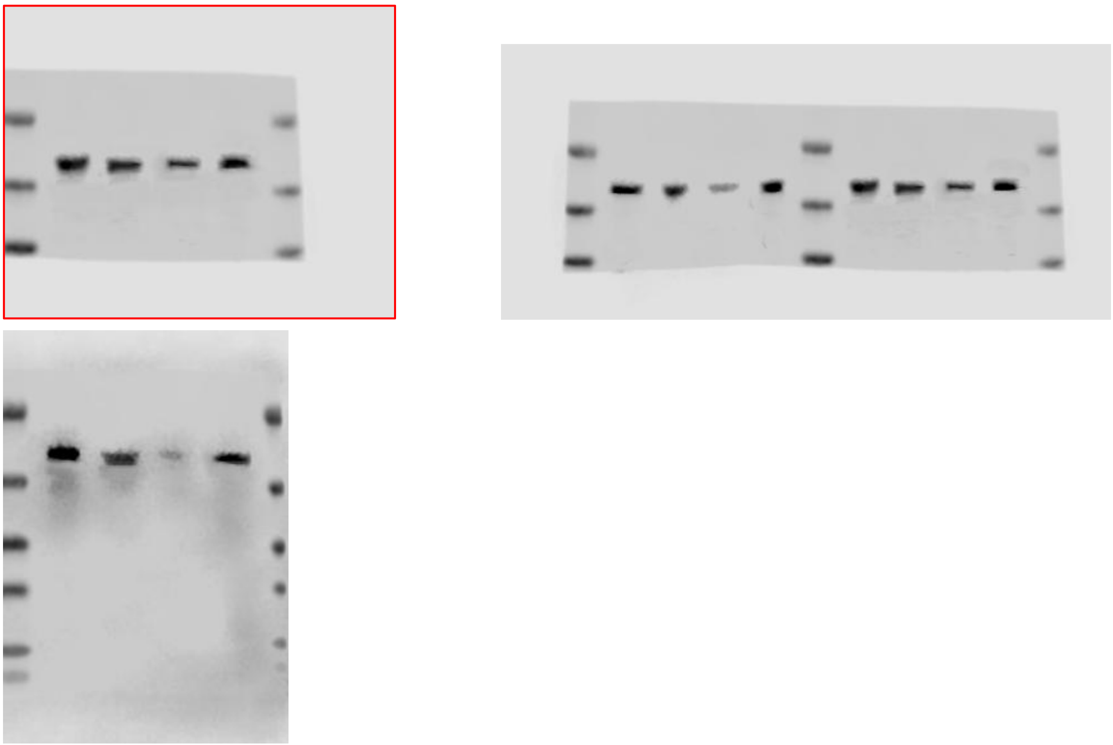

p-PKCA

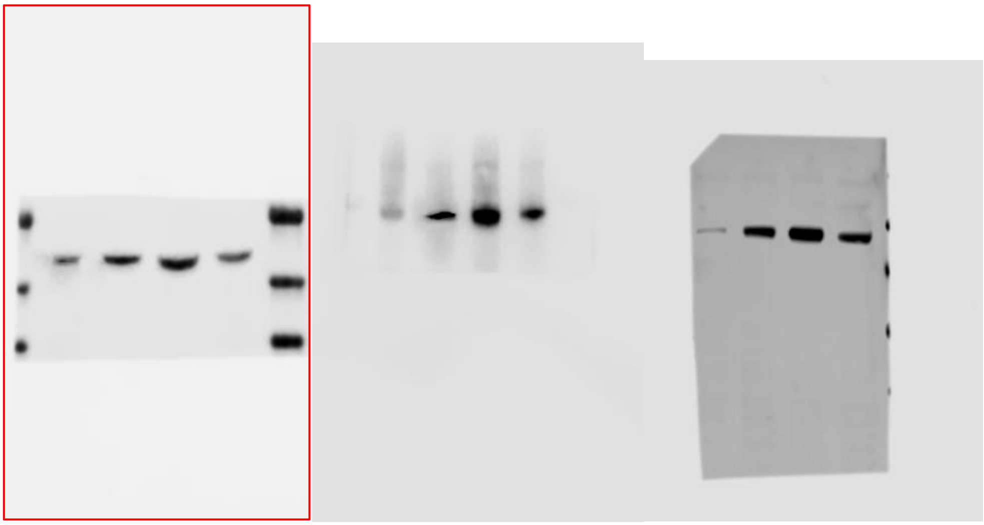

PKCA

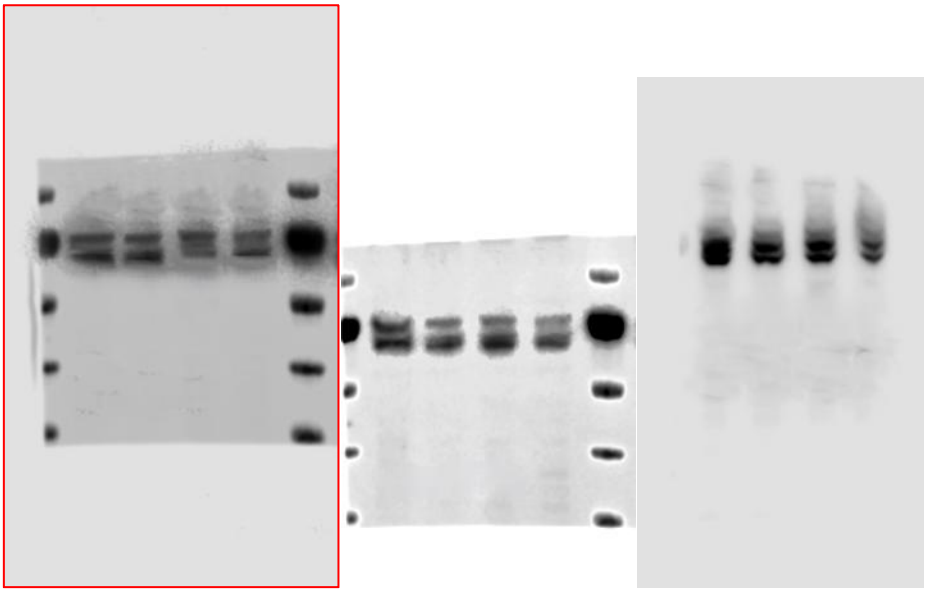

PLCD1

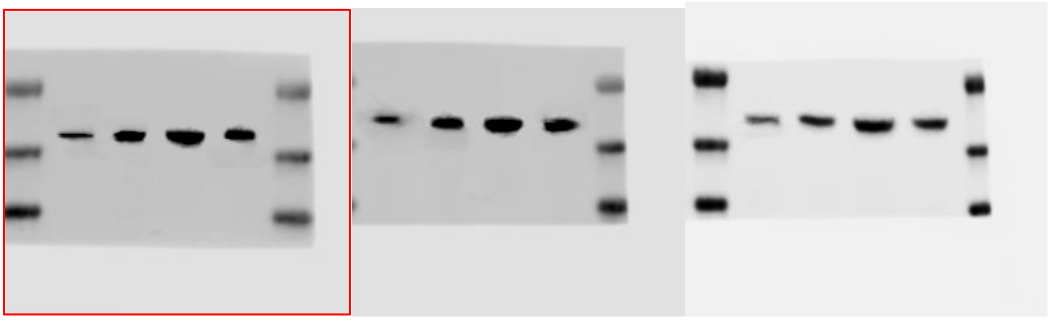

Beta-tubulin

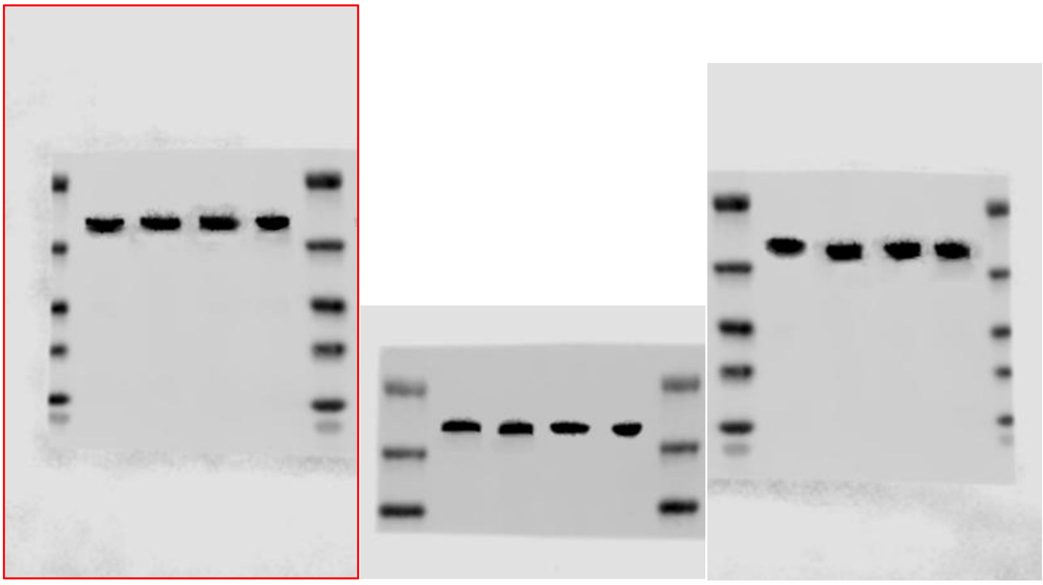

Supplement: Supplementary file 1 [file 13287_2026_5140_MOESM1_ESM.zip › 13287_2026_5140_MOESM1_ESM/13287_2026_5140_MOESM1_ESM.pdf]
