# Supplementary material for: Adipose stem cells derived extracellular vesicles alleviate retinal excitotoxicity via miR-23a-5p/PLCD1/PKCA/GluA2 axis: a potential therapeutic strategy
Source: Stem Cell Res Ther. 2026 Jun 30;17:321. doi: 10.1186/s13287-026-05140-z (PMC13587503; doi:10.1186/s13287-026-05140-z)
Supplement: Supplementary file 1 [file 13287_2026_5140_MOESM1_ESM.zip › 13287_2026_5140_MOESM1_ESM/13287_2026_5140_MOESM2_ESM.pdf]

RETIport	Enter Clinic ID<br>Enter your address!					
	Patient:	**C, DTQ, 1/3/2024**			Electrode:	DTL Thread
	Examined:	1/3/2024 12:50	Sex/Age:	male/0 days	Dilated:	Yes
	ID:		Operator:		Pupil Size:	-

1. Dark-adapted 0.01 ERG Stimulator: Ganzfeld

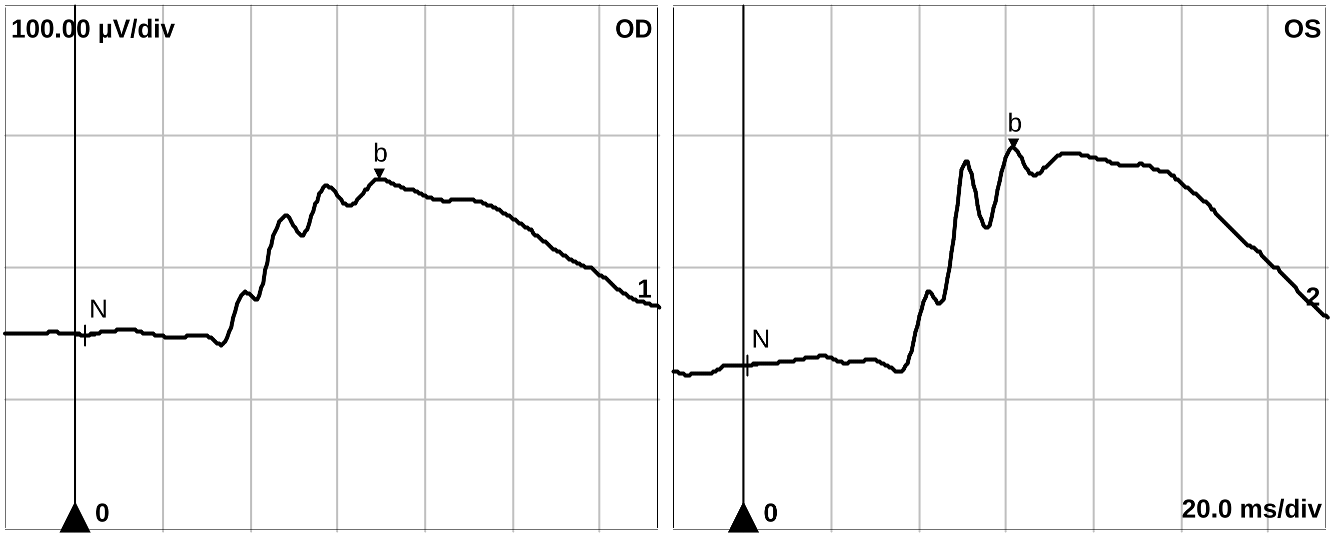

Step	b [ms]	b-wave [µV]	Avgs.
Normal			
1: OD-1	69.7	120.4	5
2: OS-2	61.8	166.8	5

2. Dark-adapted 3.0 ERG Stimulator: Ganzfeld

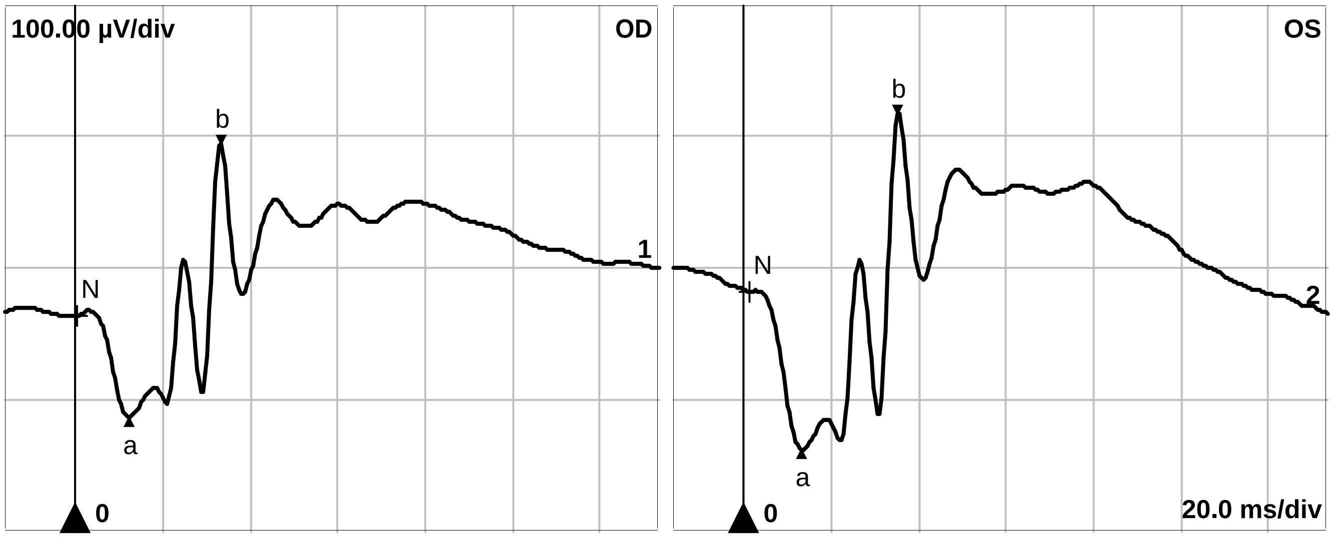

Step	a [ms]	b [ms]	a-wave [µV]	b-wave [µV]	b/a	Avgs.
Normal						
1: OD-1	12.3	33.4	77.5	209.0	2.7	5
2: OS-2	13.5	35.4	120.1	256.9	2.1	5

3. Dark-adapted 3.0 OPs Stimulator: Ganzfeld

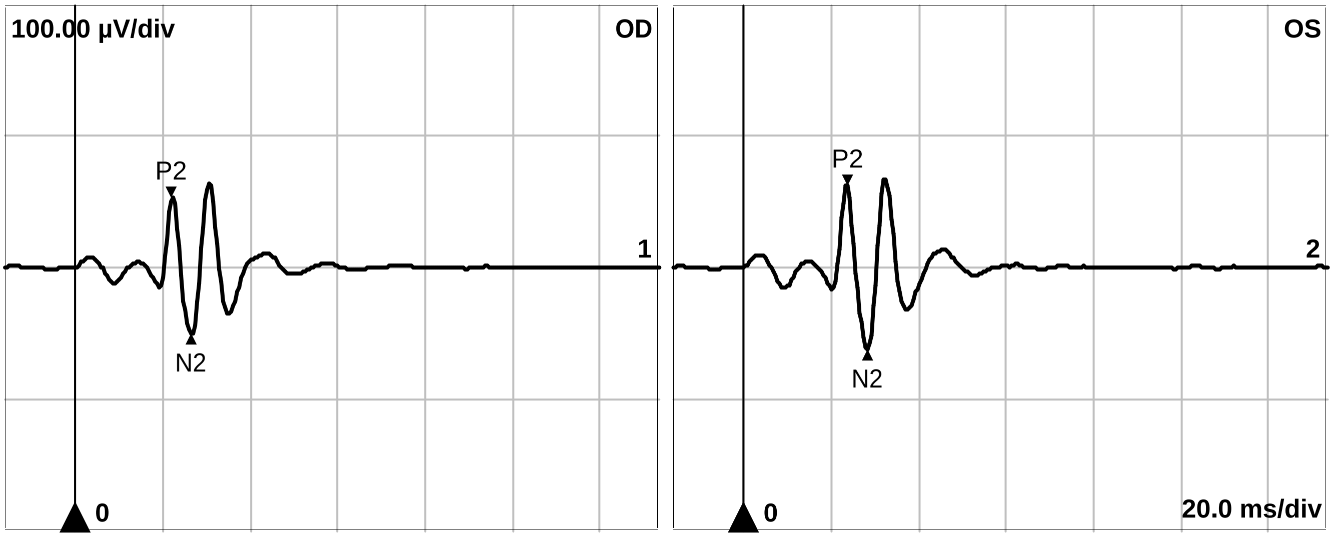

Step	P2 [ms]	OS2 [µV]	Avgs.
Normal			
1: OD-1	22.3	106.8	3
2: OS-2	23.7	126.7	3

4. Dark-adapted 10.0 ERG Stimulator: Ganzfeld

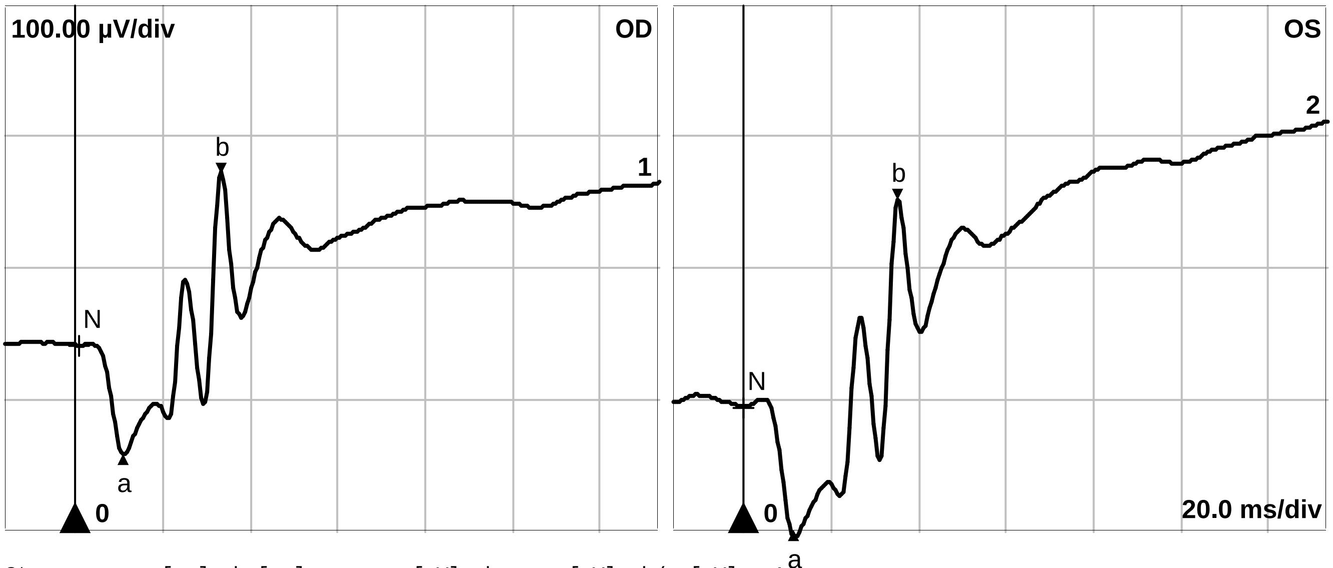

Step	a [ms]	b [ms]	a-wave [µV]	b-wave [µV]	b/a [µV]	Avgs.
Normal						
1: OD-1	11.1	33.4	82.9	216.0	2606715.7	5
2: OS-2	11.7	35.4	99.1	258.6	2609247.0	5

Patient:	CON2, DTQ, 1/3/2024			Electrode:	DTL Thread
Examined:	1/3/2024 13:45	Sex/Age:	male/0 days	Dilated:	Yes
ID:		Operator:		Pupil Size:	-

1. Dark-adapted 0.01 ERG Stimulator: Ganzfeld

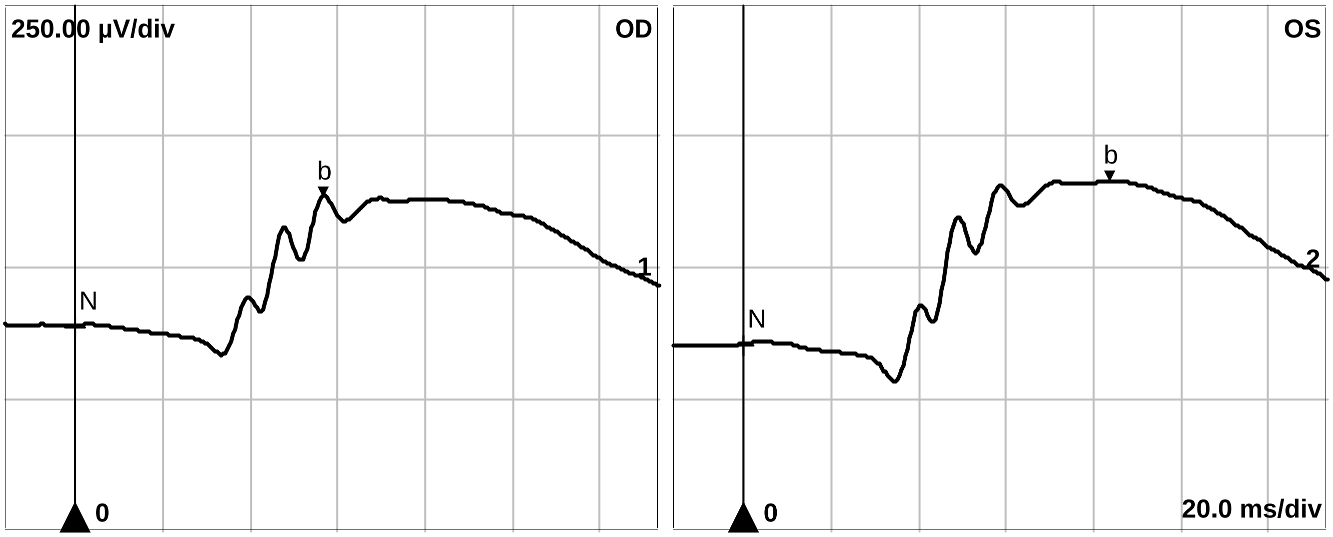

Step	b [ms]	b-wave [µV]	Avgs.
Normal			
1: OD-1	57.1	251.4	5
2: OS-2	84.1	314.8	5

2. Dark-adapted 3.0 ERG Stimulator: Ganzfeld

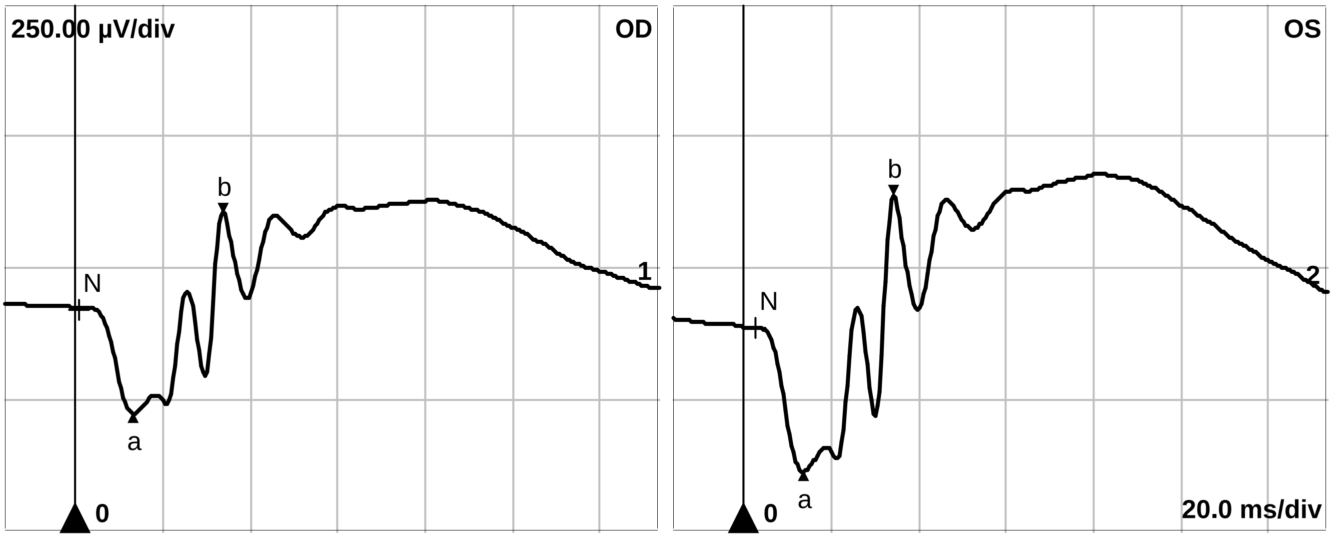

Step	a [ms]	b [ms]	a-wave [µV]	b-wave [µV]	b/a	Avgs.
Normal						
1: OD-1	13.5	34.0	199.4	386.2	1.9	5
2: OS-2	13.8	34.6	273.9	529.3	1.9	5

3. Dark-adapted 3.0 OPs Stimulator: Ganzfeld

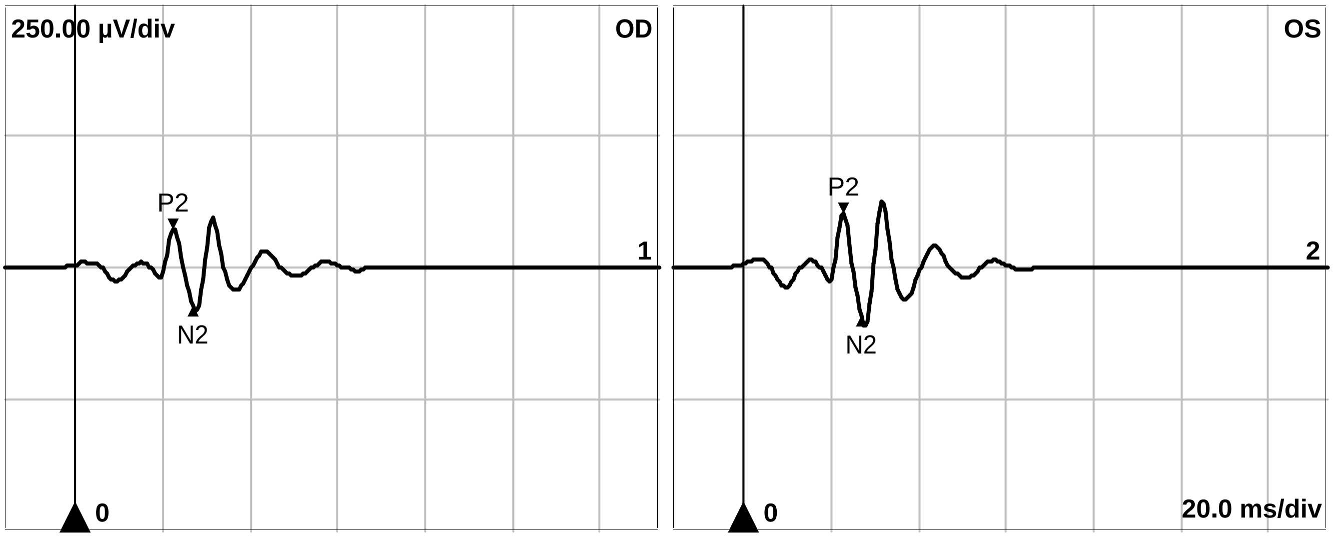

Step	P2 [ms]	OS2 [µV]	Avgs.
Normal			
1: OD-1	22.6	150.1	3
2: OS-2	22.9	200.2	3

4. Dark-adapted 10.0 ERG Stimulator: Ganzfeld

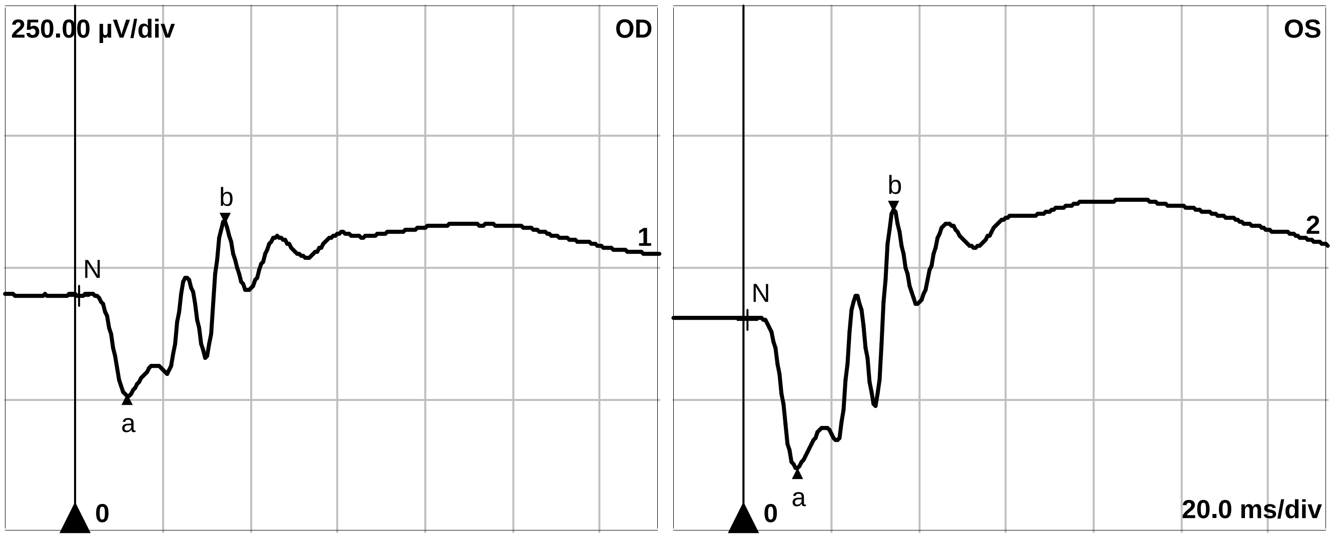

Step	a [ms]	b [ms]	a-wave [µV]	b-wave [µV]	b/a [µV]	Avgs.
Normal						
1: OD-1	12.0	34.3	191.3	334.2	1746995.7	5
2: OS-2	12.3	34.3	284.0	494.7	1742256.9	5

RETIport	Enter Clinic ID<br>Enter your address!			
Patient:	C3, DTQ, 1/3/2024		Electrode:	DTL Thread
Examined:	1/3/2024 11:21	Sex/Age: male/0 days	Dilated:	Yes
ID:		Operator:	Pupil Size:	-

1. Dark-adapted 0.01 ERG Stimulator: Ganzfeld

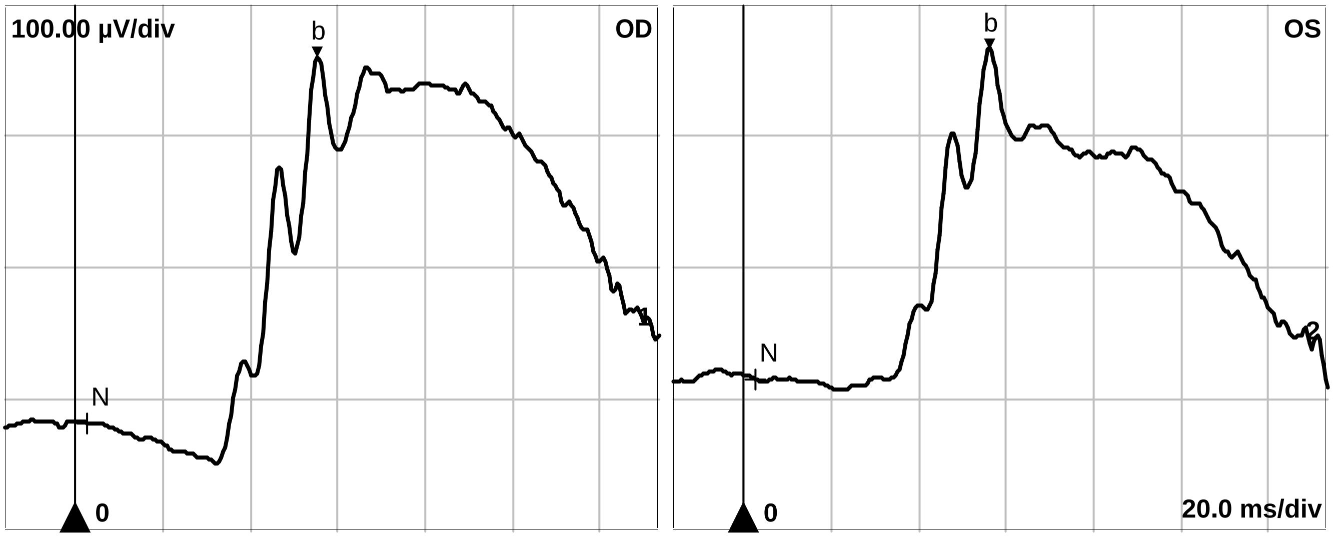

Step	b [ms]	b-wave [µV]	Avgs.
Normal			
1: OD-1	55.7	280.2	5
2: OS-2	56.3	252.9	5

2. Dark-adapted 3.0 ERG Stimulator: Ganzfeld

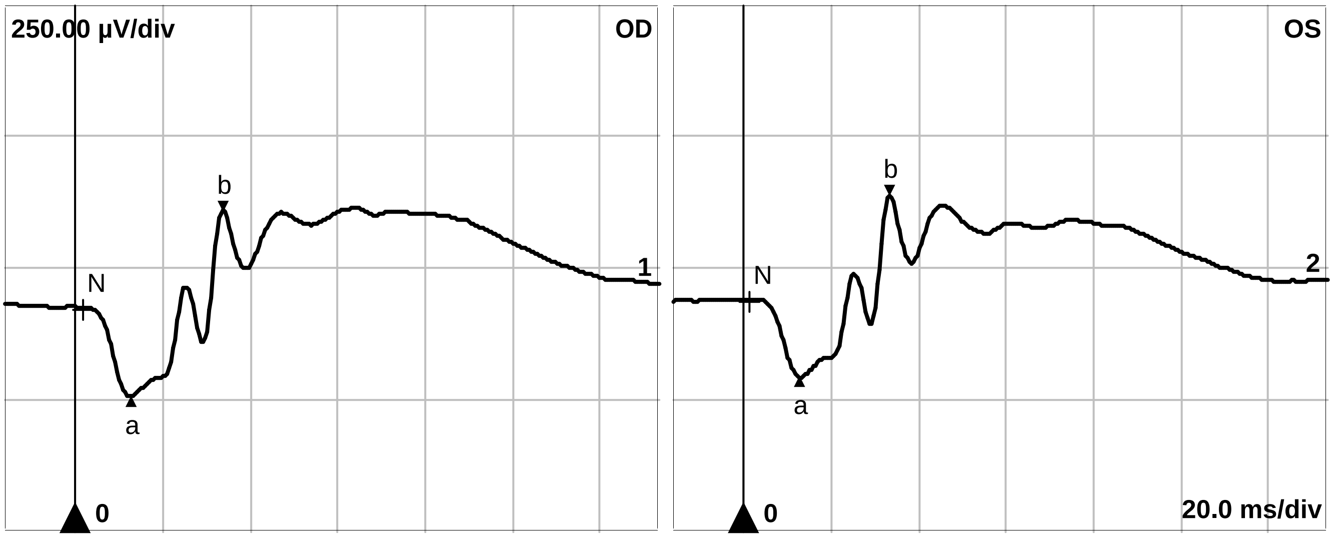

Step	a [ms]	b [ms]	a-wave [µV]	b-wave [µV]	b/a	Avgs.
Normal						
1: OD-1	12.9	34.0	167.5	357.5	2.1	5
2: OS-2	13.2	33.4	146.9	349.1	2.4	5

Patient:	C3, DTQ, 1/3/2024			Electrode:	DTL Thread
Examined:	1/3/2024 11:21	Sex/Age:	male/0 days	Dilated:	Yes
ID:		Operator:		Pupil Size:	-

3. Dark-adapted 3.0 OPs Stimulator: Ganzfeld

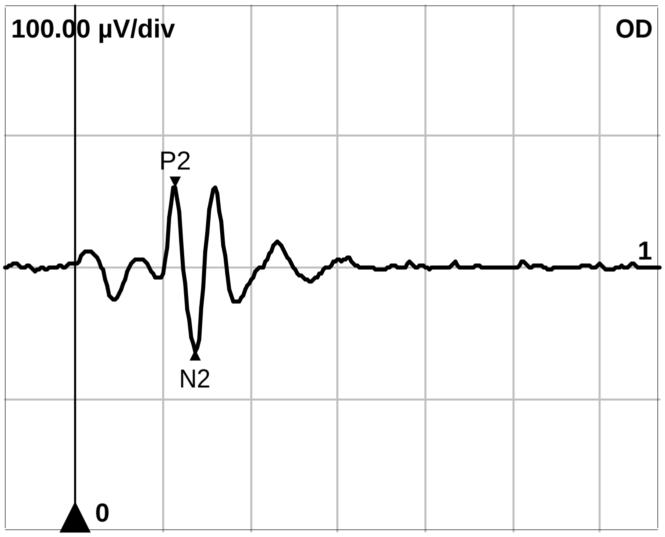

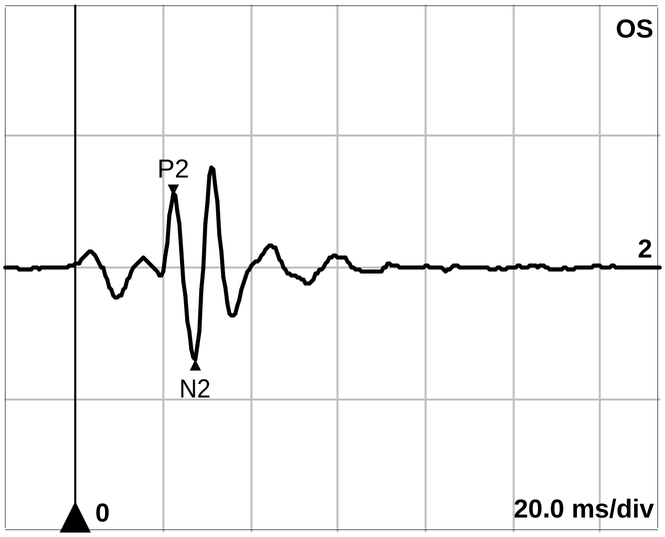

Step	P2 [ms]	OS2 [µV]	Avgs.
Normal			
1: OD-1	22.9	126.4	3
2: OS-2	22.6	127.9	3

4. Dark-adapted 10.0 ERG Stimulator: Ganzfeld

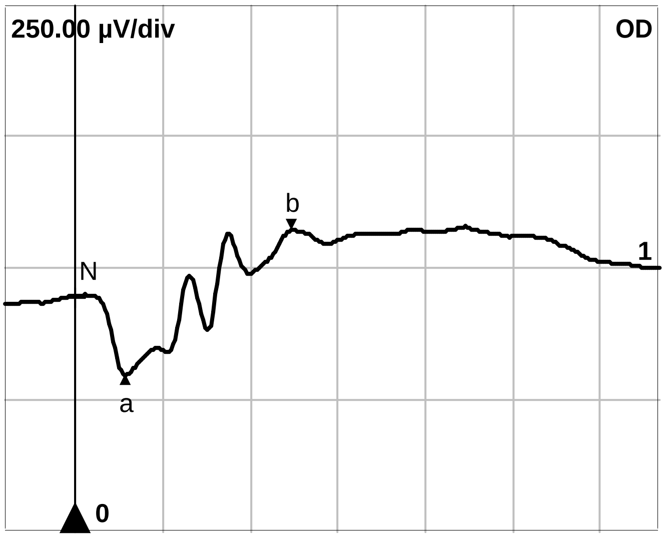

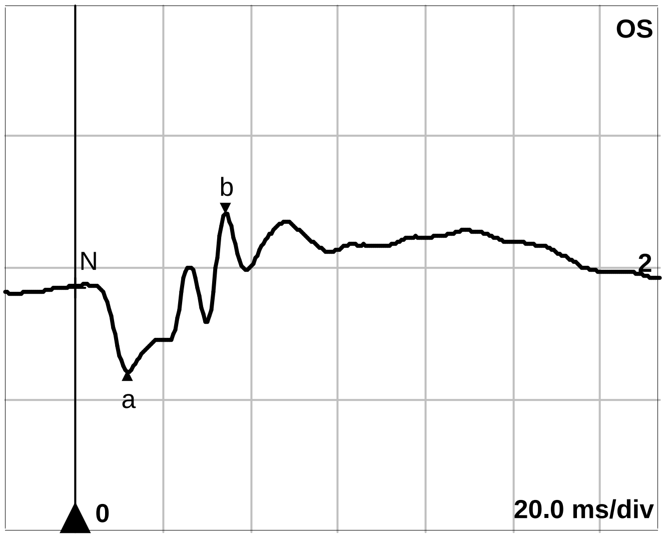

Step	a [ms]	b [ms]	a-wave [µV]	b-wave [µV]	b/a [µV]	Avgs.
Normal						
1: OD-1	11.7	49.8	149.0	280.1	1880251.8	5
2: OS-2	12.0	34.6	161.0	304.1	1889598.7	5

RETIport

Enter Clinic ID
Enter your address!

Patient: G1, DTQ, 5/7/2021 | Electrode: DTL Thread
Examined: 12/6/2023 10:36 | Sex/Age: male/2 | Dilated: Yes
ID: | Operator: | Pupil Size: -

1. Dark-adapted 0.01 ERG Stimulator: Ganzfeld

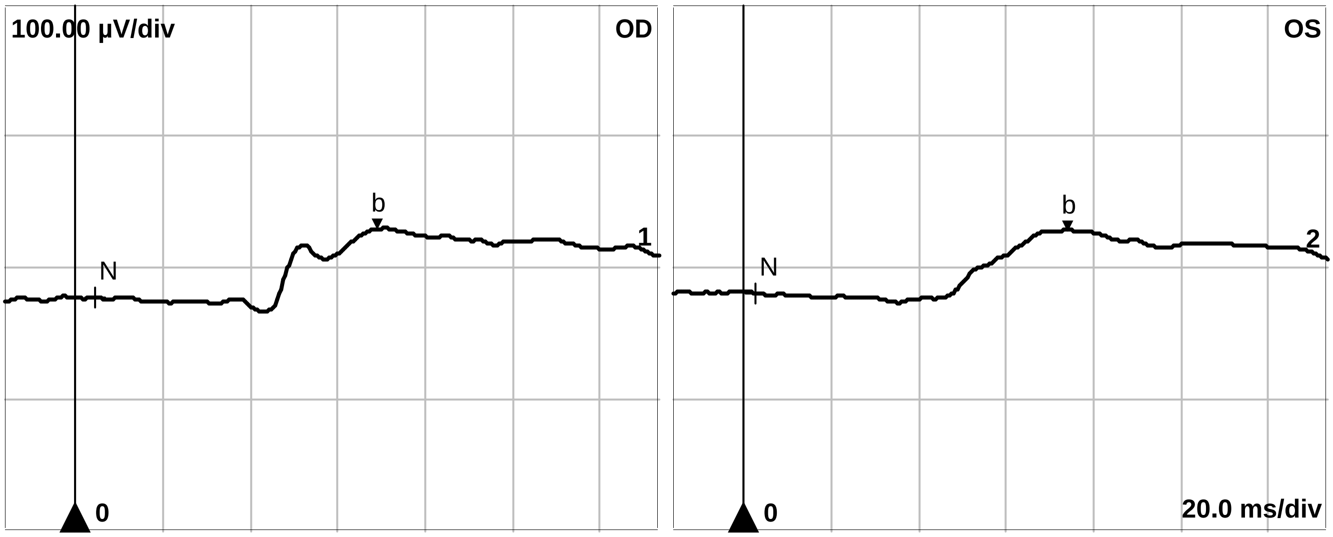

Step	b [ms]	b-wave [µV]	Avgs.
Normal	66.0-93.0	107.0-356.0	
1: OD-1	69.1	52.9 (!)	5
2: OS-2	74.4	49.9 (!)	5

2. Dark-adapted 3.0 ERG Stimulator: Ganzfeld

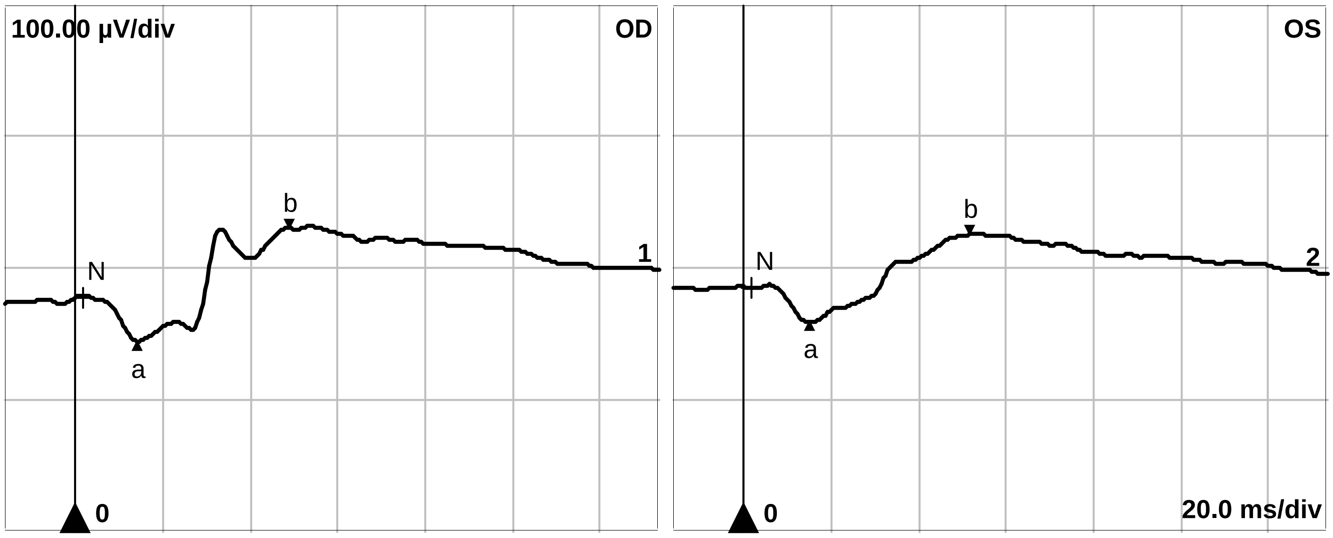

Step	a [ms]	b [ms]	a-wave [µV]	b-wave [µV]	b/a	Avgs.
Normal	15.3-19.4	32.9-58.7	118.0-315.0	168.0-527.0	1.5-2.6	
1: OD-1	14.4 (!)	48.9	34.4 (!)	87.5 (!)	2.5	5
2: OS-2	15.2 (!)	51.9	25.7 (!)	67.7 (!)	2.6 (!)	5

RETIport	Enter Clinic ID<br>Enter your address!					
	Patient:	G1, DTQ, 5/7/2021			Electrode:	DTL Thread
	Examined:	12/6/2023 10:36	Sex/Age:	male/2	Dilated:	Yes
	ID:		Operator:		Pupil Size:	-

3. Dark-adapted 3.0 OPs Stimulator: Ganzfeld

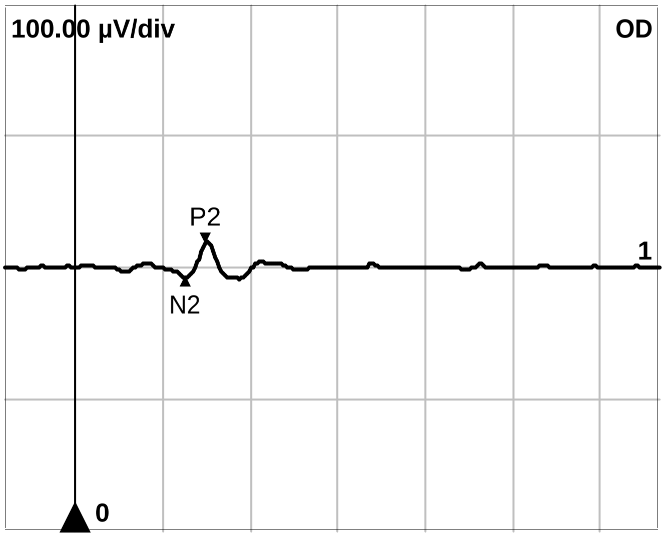

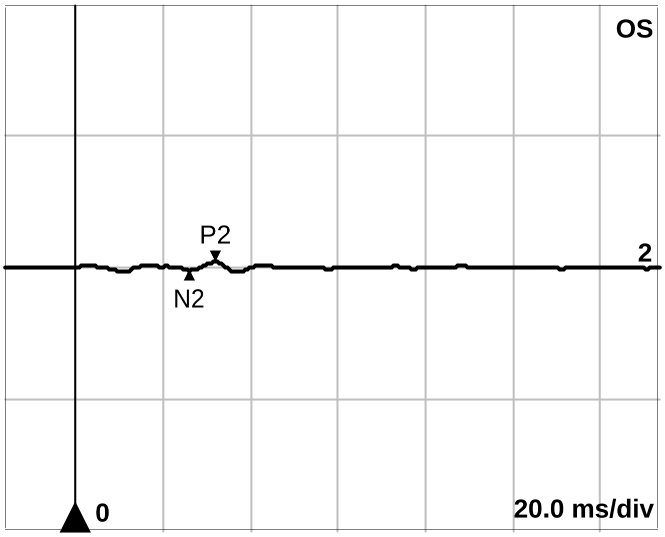

Step	P2 [ms]	OS2 [µV]	Avgs.
Normal	22.9-25.8	14.7-71.1	
1: OD-1	29.9 (!)	27.5	3
2: OS-2	32.2 (!)	8.6 (!)	3

4. Dark-adapted 10.0 ERG Stimulator: Ganzfeld

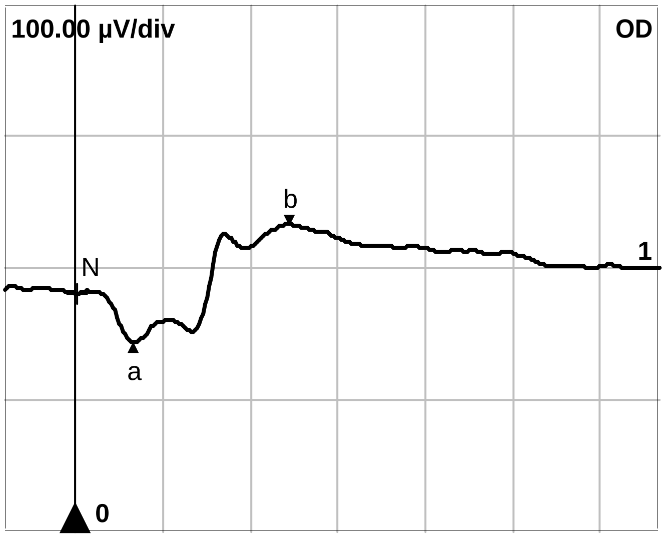

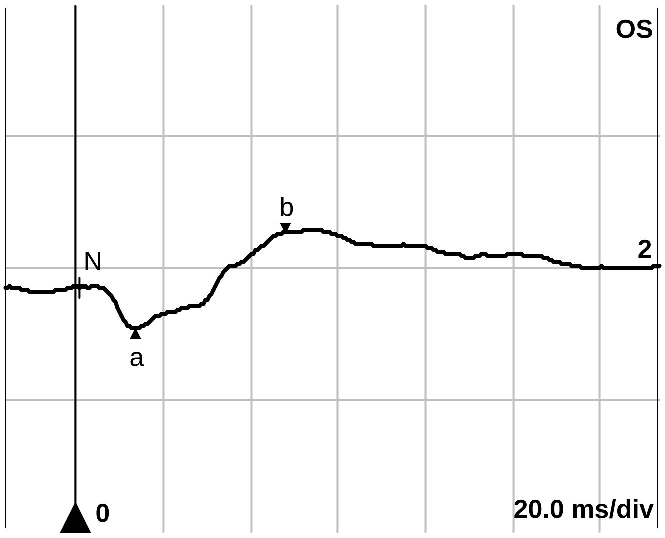

Step	a [ms]	b [ms]	a-wave [µV]	b-wave [µV]	b/a [µV]	Avgs.
Normal	12.9-17.0	32.6-50.8	135.0-322.0	180.0-550.0		
1: OD-1	13.5	48.9	37.3 (!)	91.4 (!)	2453134.8	5
2: OS-2	13.8	48.0	31.8 (!)	75.0 (!)	2360113.1	5

RETIport	Enter Clinic ID<br>Enter your address!					
	Patient:	G2-1, DTQ, 5/7/2021			Electrode:	DTL Thread
	Examined:	12/6/2023 09:56	Sex/Age:	male/2	Dilated:	Yes
	ID:		Operator:		Pupil Size:	-

1. Dark-adapted 0.01 ERG Stimulator: Ganzfeld

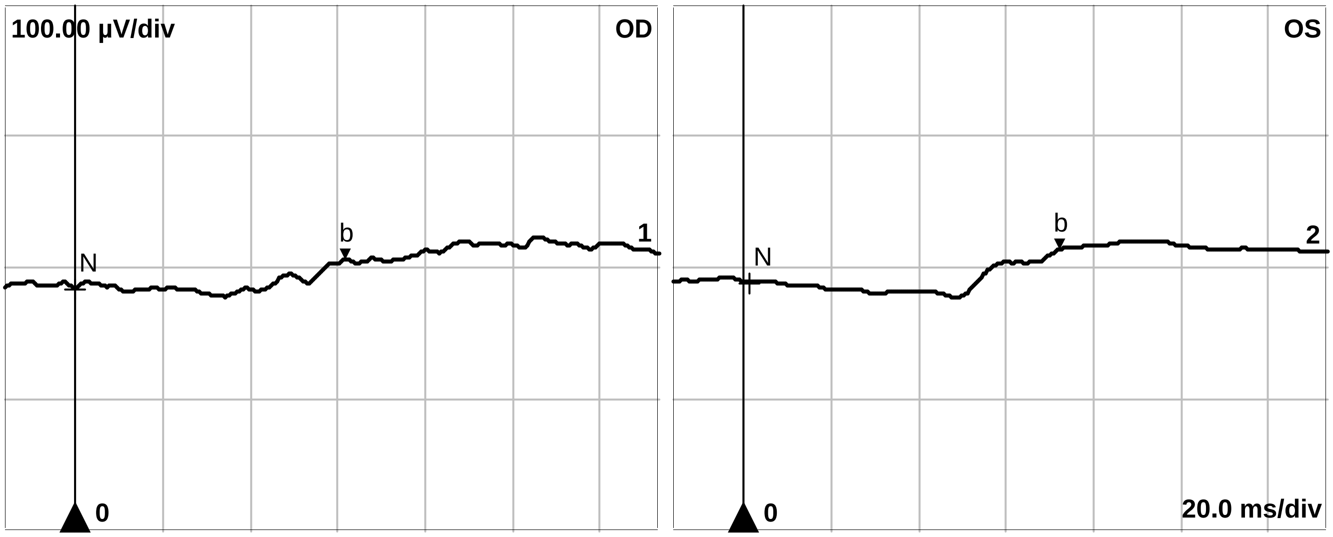

Step	b [ms]	b-wave [µV]	Avgs.
Normal	66.0-93.0	107.0-356.0	
1: OD-1	62.1 (!)	24.2 (!)	5
2: OS-2	72.7	26.5 (!)	5

2. Dark-adapted 3.0 ERG Stimulator: Ganzfeld

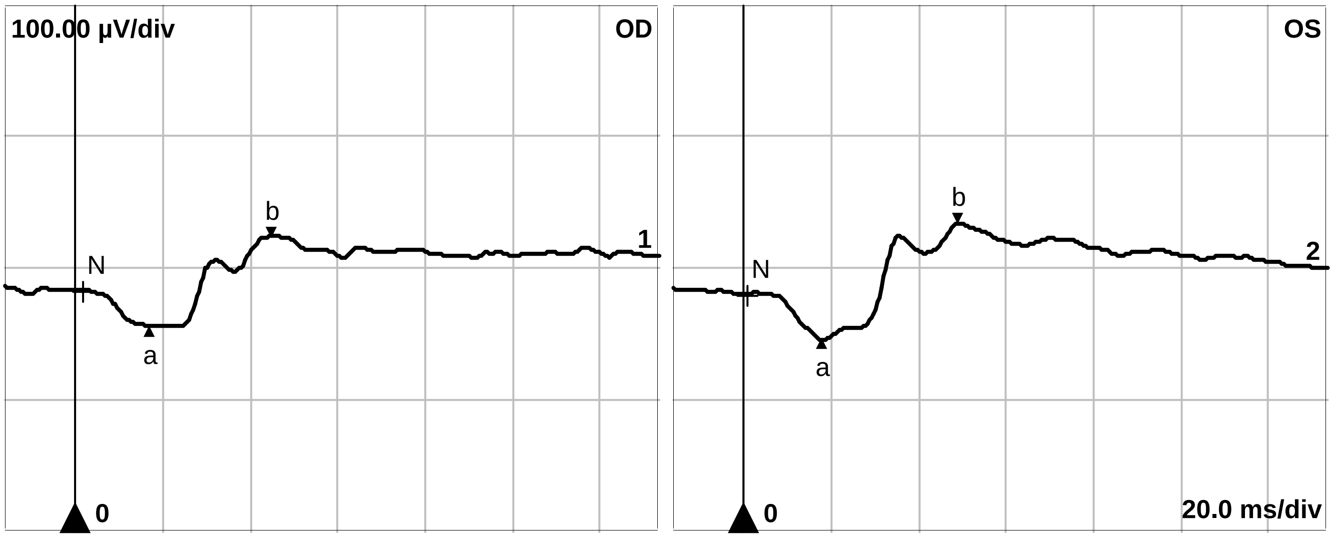

Step	a [ms]	b [ms]	a-wave [µV]	b-wave [µV]	b/a	Avgs.
Normal	15.3-19.4	32.9-58.7	118.0-315.0	168.0-527.0	1.5-2.6	
1: OD-1	17.0	45.1	27.0 (!)	70.1 (!)	2.6 (!)	5
2: OS-2	18.2	49.2	34.4 (!)	89.6 (!)	2.6 (!)	5

RETIport	Enter Clinic ID<br>Enter your address!					
	Patient:	G2, DTQ, 5/7/2021			Electrode:	DTL Thread
	Examined:	12/6/2023 09:56	Sex/Age:	male/2	Dilated:	Yes
	ID:		Operator:		Pupil Size:	-

3. Dark-adapted 3.0 OPs Stimulator: Ganzfeld

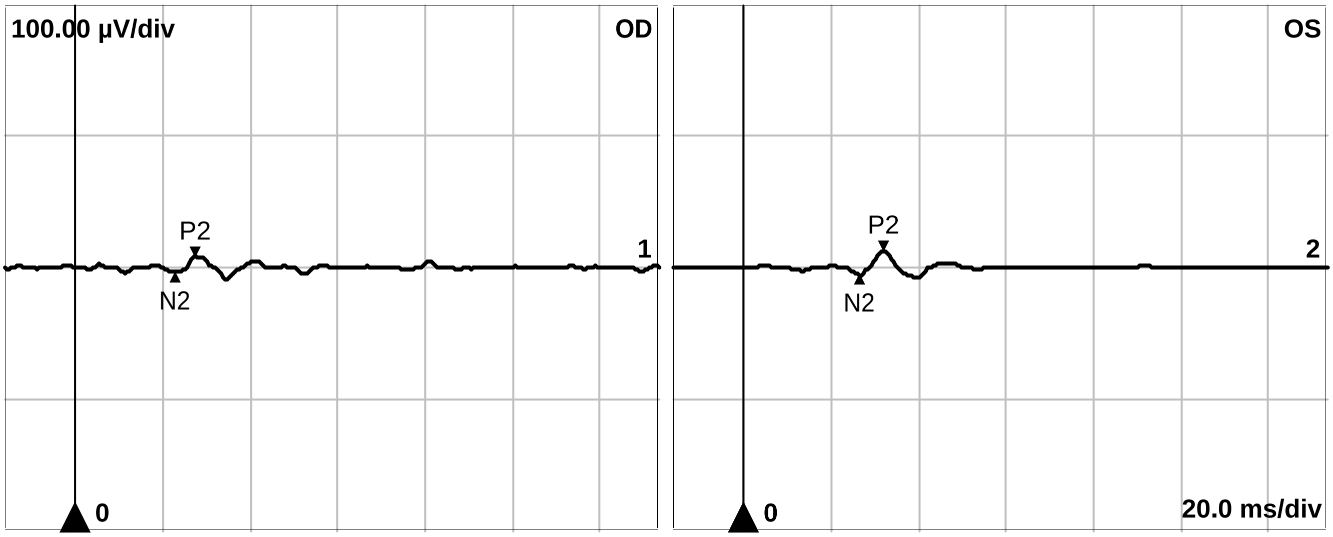

Step	P2 [ms]	OS2 [µV]	Avgs.
Normal	22.9-25.8	14.7-71.1	
1: OD-1	27.5 (!)	14.1 (!)	3
2: OS-2	32.2 (!)	19.8	3

4. Dark-adapted 10.0 ERG Stimulator: Ganzfeld

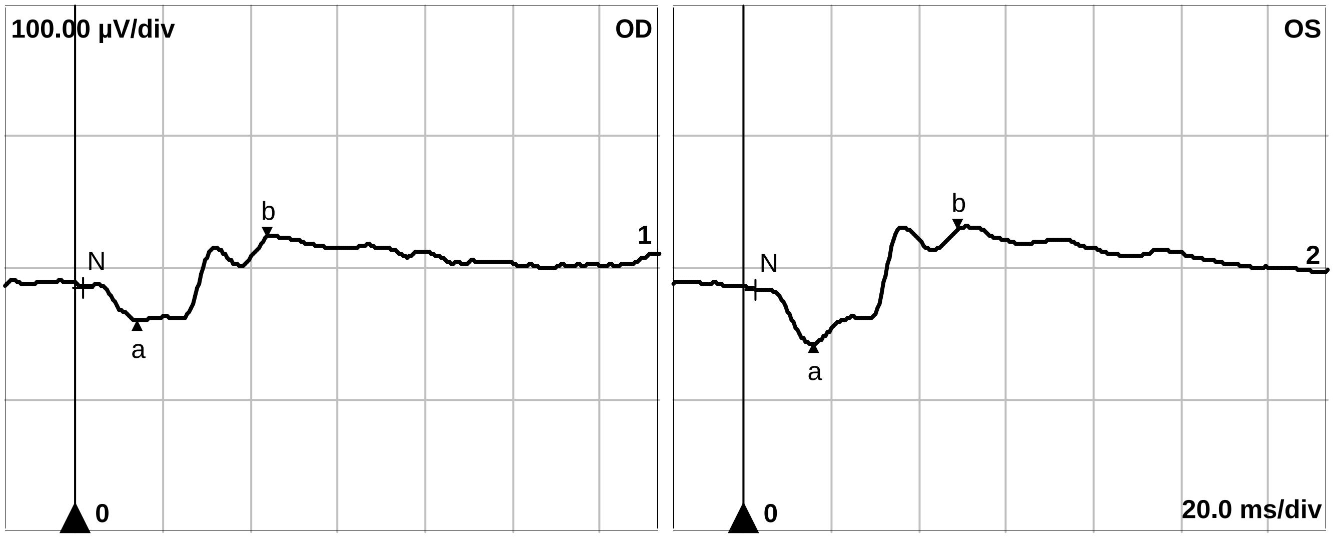

Step	a [ms]	b [ms]	a-wave [µV]	b-wave [µV]	b/a [µV]	Avgs.
Normal	12.9-17.0	32.6-50.8	135.0-322.0	180.0-550.0		
1: OD-1	14.4	44.2	25.7 (!)	65.6 (!)	2555466.2	5
2: OS-2	16.1	49.2	41.3 (!)	88.4 (!)	2139966.5	5

RETIport	Enter Clinic ID<br>Enter your address!				
	Patient: G3, DTQ, 5/7/2021			Electrode:	DTL Thread
	Examined: 12/5/2023 10:55	Sex/Age:	male/2	Dilated:	Yes
	ID:	Operator:		Pupil Size:	-

1. Dark-adapted 0.01 ERG Stimulator: Ganzfeld

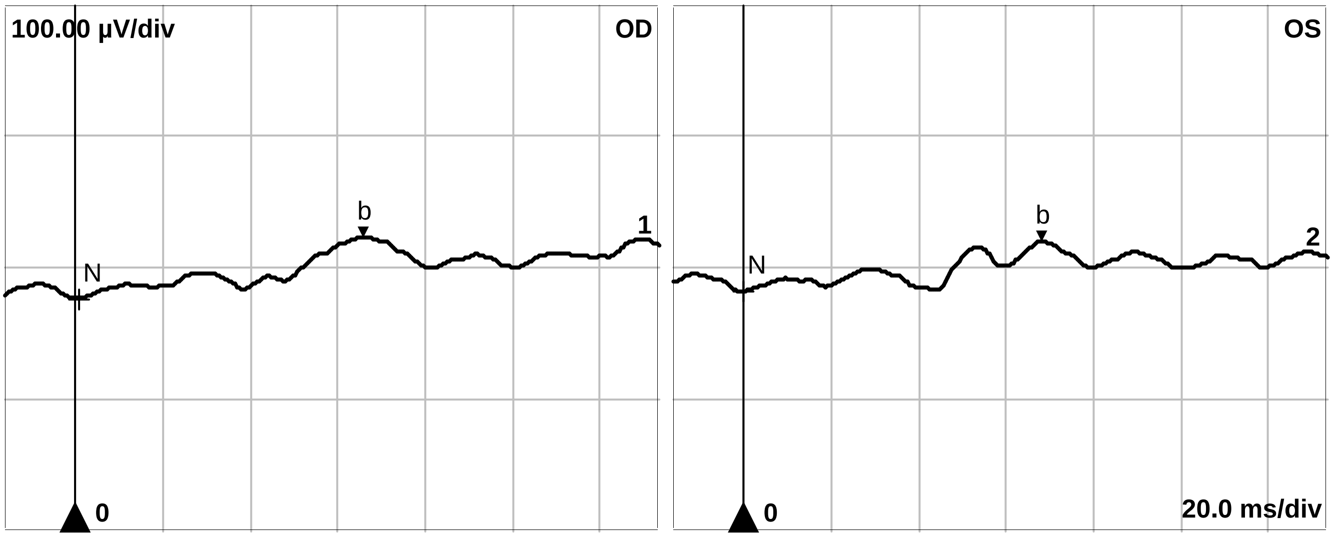

Step	b [ms]	b-wave [µV]	Avgs.
Normal	66.0-93.0	107.0-356.0	
1: OD-1	66.2	48.0 (!)	5
2: OS-2	68.6	39.4 (!)	5

2. Dark-adapted 3.0 ERG Stimulator: Ganzfeld

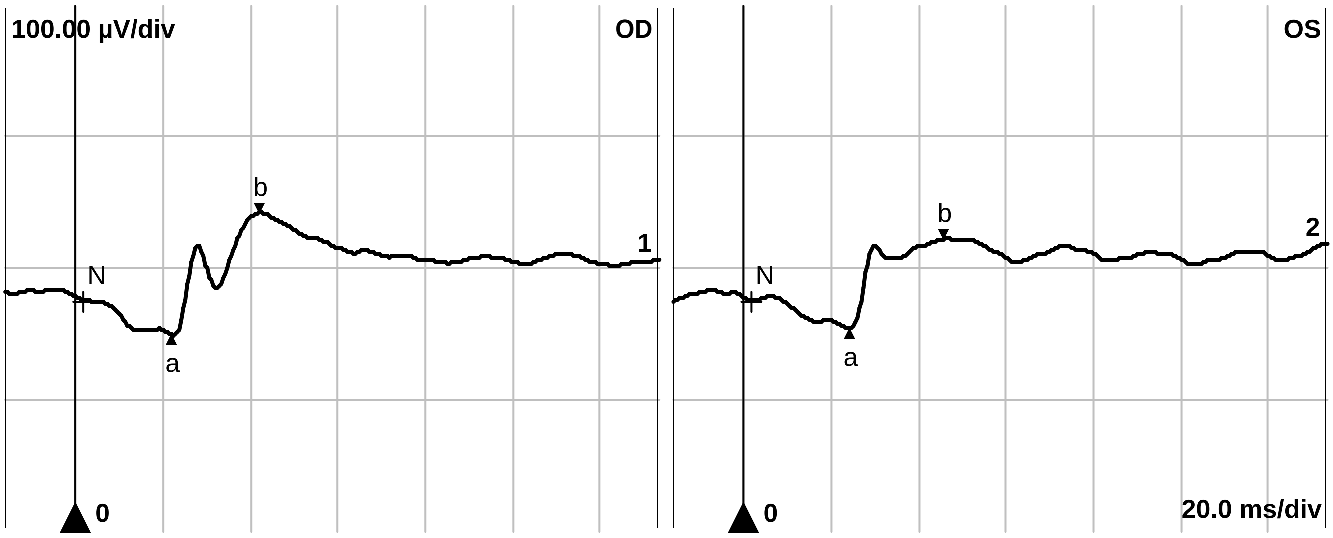

Step	a [ms]	b [ms]	a-wave [µV]	b-wave [µV]	b/a	Avgs.
Normal	15.3-19.4	32.9-58.7	118.0-315.0	168.0-527.0	1.5-2.6	
1: OD-1	22.3 (!)	42.2	26.5 (!)	94.5 (!)	3.6 (!)	5
2: OS-2	24.3 (!)	46.0	21.2 (!)	69.6 (!)	3.3 (!)	5

RETIport	Enter Clinic ID<br>Enter your address!					
	Patient:	G3, DTQ, 5/7/2021			Electrode:	DTL Thread
	Examined:	12/5/2023 10:55	Sex/Age:	male/2	Dilated:	Yes
	ID:		Operator:		Pupil Size:	-

3. Dark-adapted 3.0 OPs Stimulator: Ganzfeld

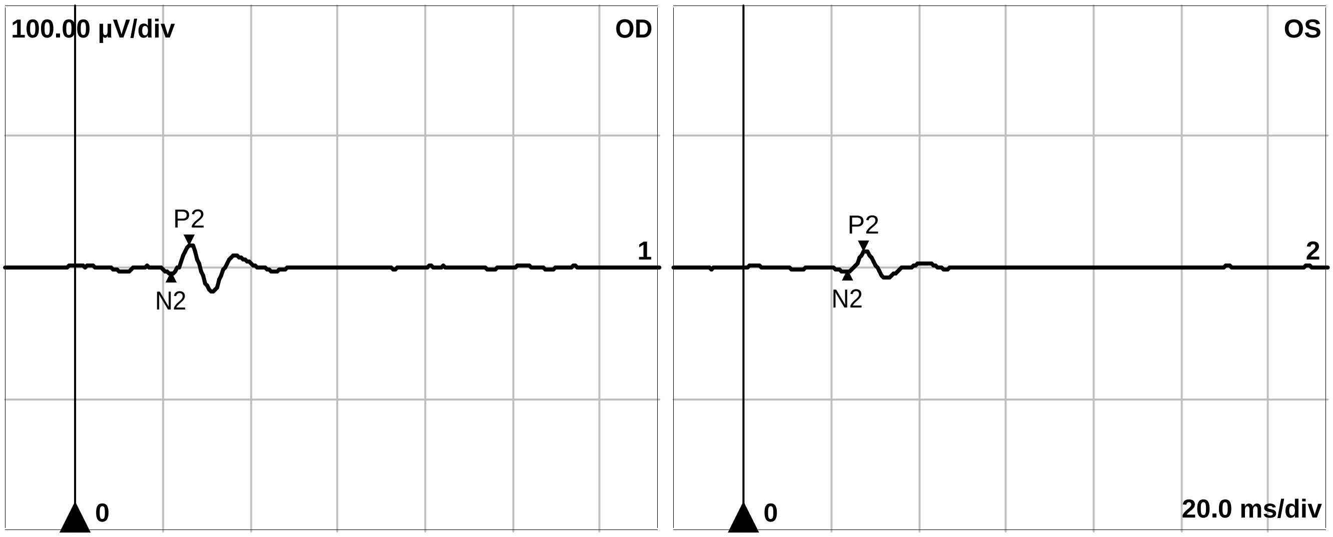

Step	P2 [ms]	OS2 [µV]	Avgs.
Normal	22.9-25.8	14.7-71.1	
1: OD-1	26.4 (!)	22.9	3
2: OS-2	27.8 (!)	16.9	3

4. Dark-adapted 10.0 ERG Stimulator: Ganzfeld

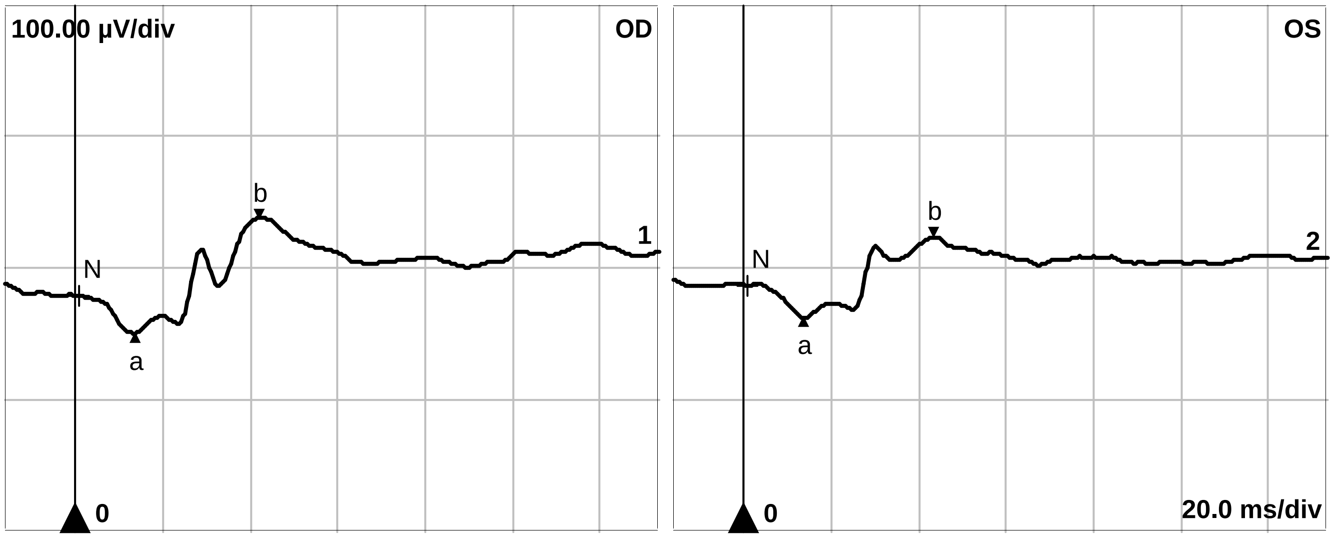

Step	a [ms]	b [ms]	a-wave [µV]	b-wave [µV]	b/a [µV]	Avgs.
Normal	12.9-17.0	32.6-50.8	135.0-322.0	180.0-550.0		
1: OD-1	13.8	42.2	28.6 (!)	89.2 (!)	3120024.0	5
2: OS-2	13.8	43.7	24.4 (!)	62.7 (!)	2574112.9	5

1. Dark-adapted 0.01 ERG Stimulator: Ganzfeld

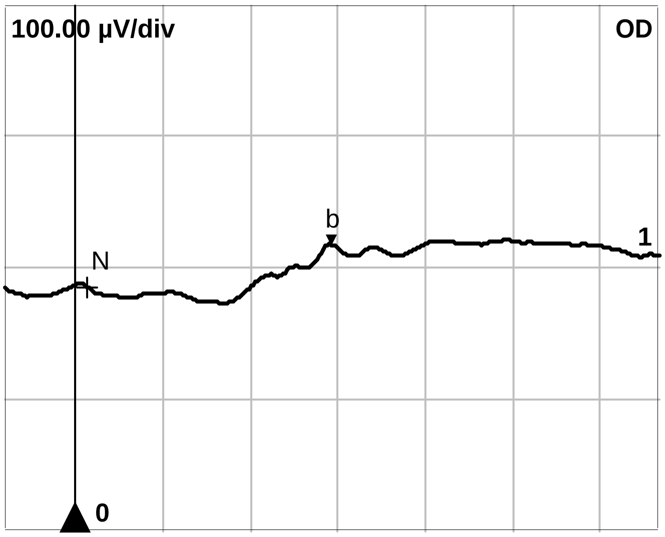

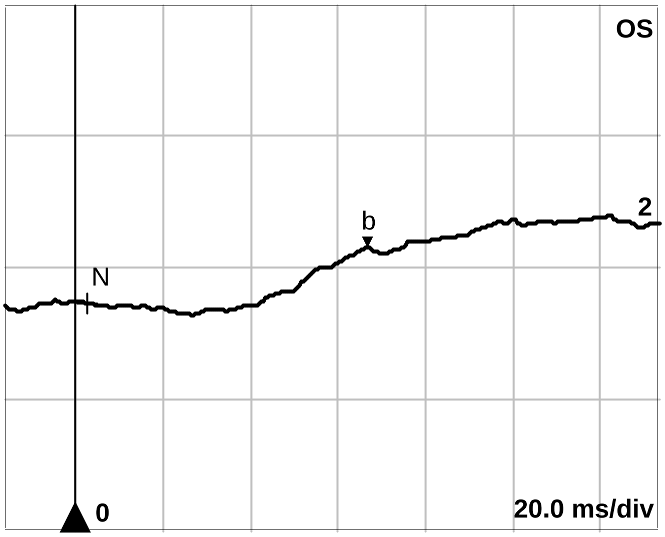

Step	b [ms]	b-wave [µV]	Avgs.
Normal			
1: OD-1	58.6	33.7	5
2: OS-2	66.8	43.5	5

2. Dark-adapted 3.0 ERG Stimulator: Ganzfeld

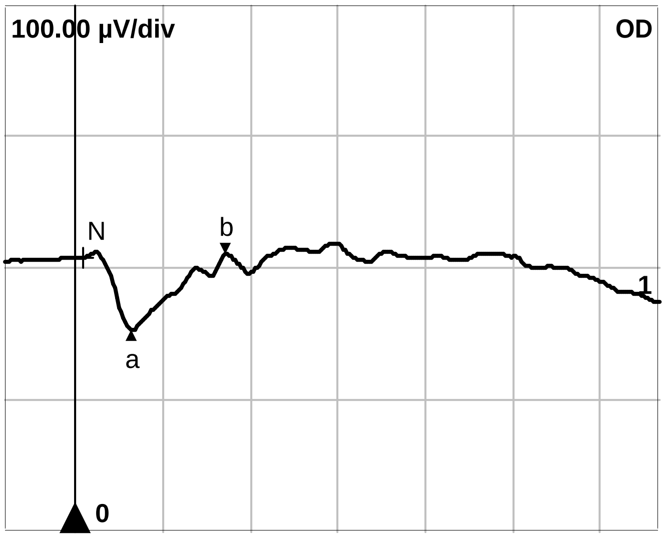

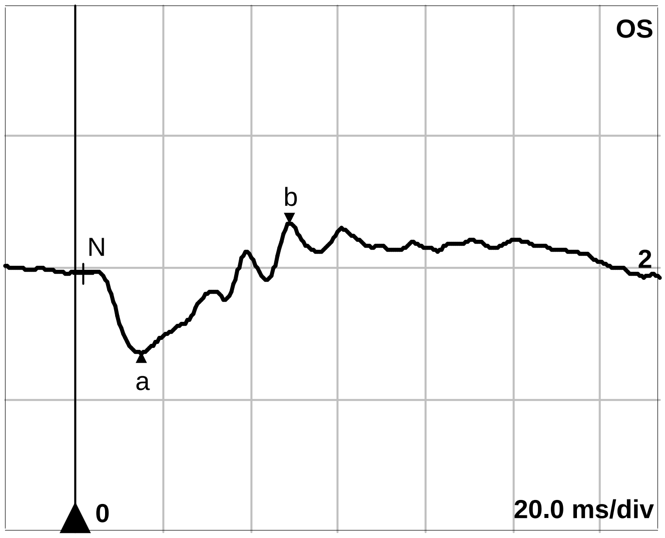

Step	a [ms]	b [ms]	a-wave [µV]	b-wave [µV]	b/a	Avgs.
Normal						
1: OD-1	13.2	34.6	56.2	60.0	1.1	5
2: OS-2	15.2	49.2	61.0	100.5	1.6	5

3. Dark-adapted 3.0 OPs Stimulator: Ganzfeld

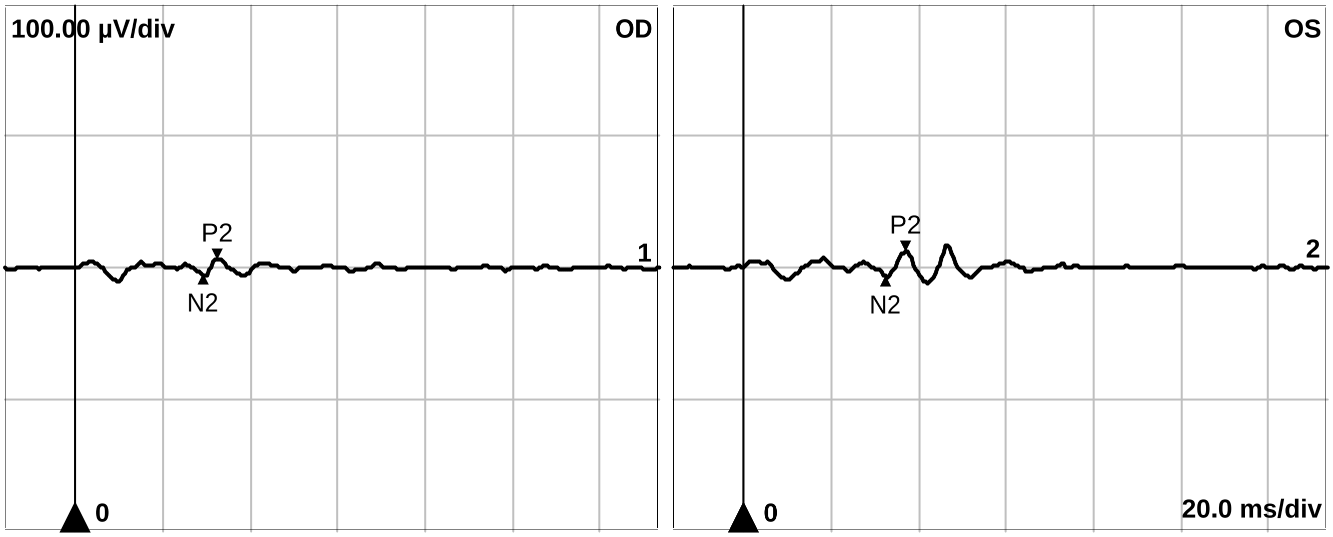

Step	P2 [ms]	OS2 [µV]	Avgs.
Normal			
1: OD-1	32.8	13.9	3
2: OS-2	37.2	21.0	3

4. Dark-adapted 10.0 ERG Stimulator: Ganzfeld

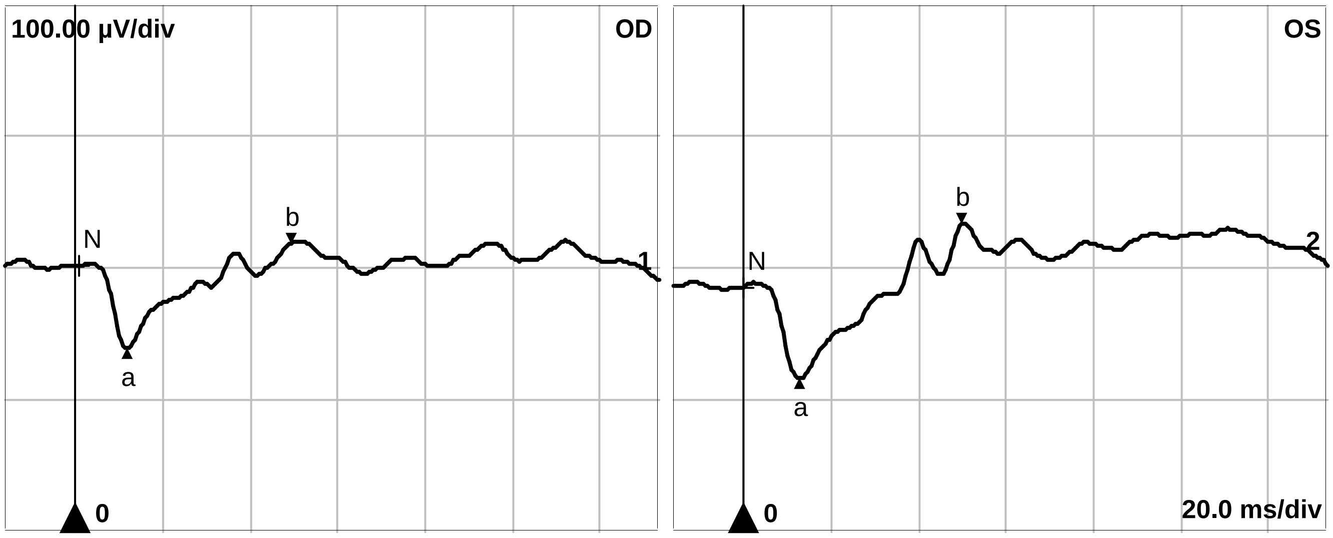

Step	a [ms]	b [ms]	a-wave [µV]	b-wave [µV]	b/a [µV]	Avgs.
Normal						
1: OD-1	12.0	49.8	63.2	81.8	1293236.4	5
2: OS-2	13.2	50.1	69.9	119.7	1713782.8	5

RETIport	Enter Clinic ID<br>Enter your address!				
	Patient:	8-1, DTQ, 5/7/2021		Electrode:	DTL Thread
	Examined:	12/6/2023 10:51	Sex/Age: male/2	Dilated:	Yes
	ID:		Operator:	Pupil Size:	-

1. Dark-adapted 0.01 ERG Stimulator: Ganzfeld

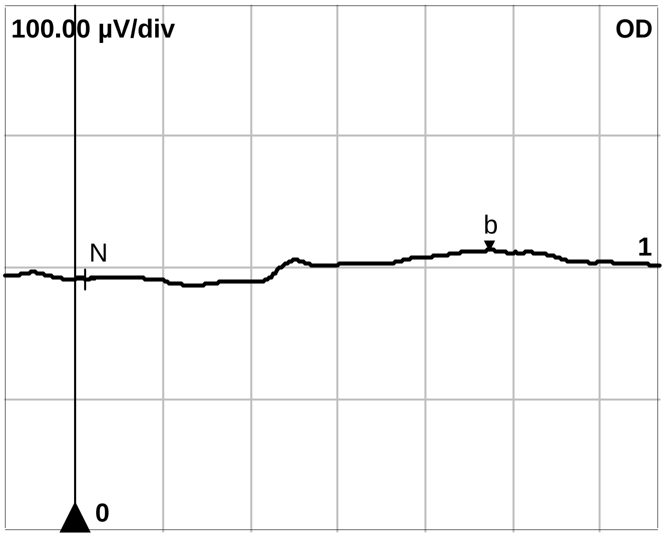

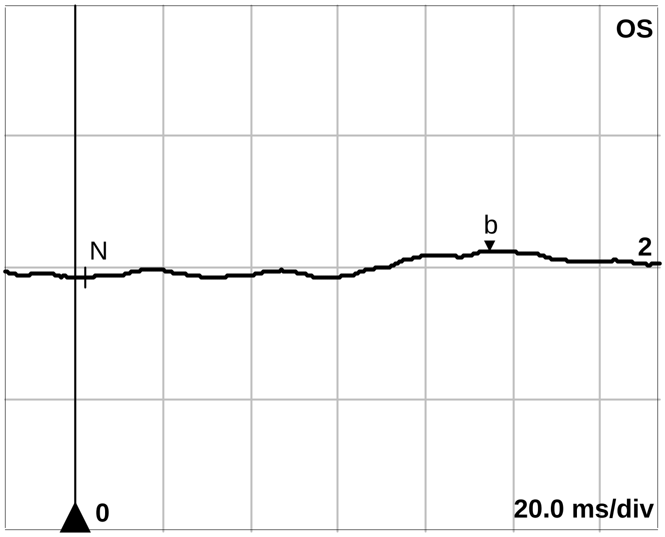

Step	b [ms]	b-wave [µV]	Avgs.
Normal	66.0-93.0	107.0-356.0	
1: OD-1	94.9 (!)	23.2 (!)	5
2: OS-2	94.9 (!)	21.8 (!)	5

2. Dark-adapted 3.0 ERG Stimulator: Ganzfeld

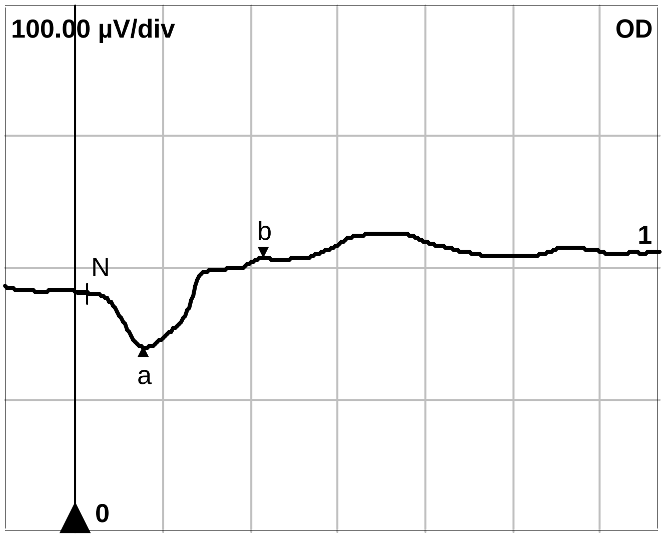

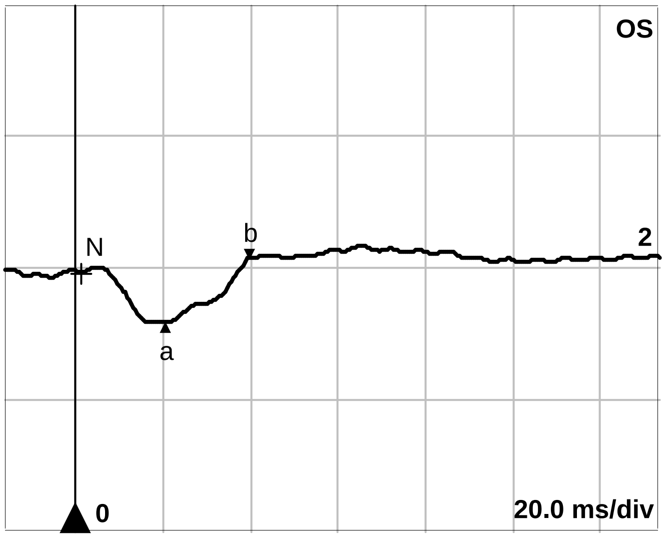

Step	a [ms]	b [ms]	a-wave [µV]	b-wave [µV]	b/a	Avgs.
Normal	15.3-19.4	32.9-58.7	118.0-315.0	168.0-527.0	1.5-2.6	
1: OD-1	15.8	43.1	41.3 (!)	69.7 (!)	1.7	5
2: OS-2	20.8 (!)	39.8	38.6 (!)	50.8 (!)	1.3 (!)	5

RETIport	Enter Clinic ID<br>Enter your address!					
	Patient:	**8-1, DTQ, 5/7/2021**			Electrode:	DTL Thread
	Examined:	12/6/2023 10:51	Sex/Age:	male/2	Dilated:	Yes
	ID:		Operator:		Pupil Size:	-

3. Dark-adapted 3.0 OPs Stimulator: Ganzfeld

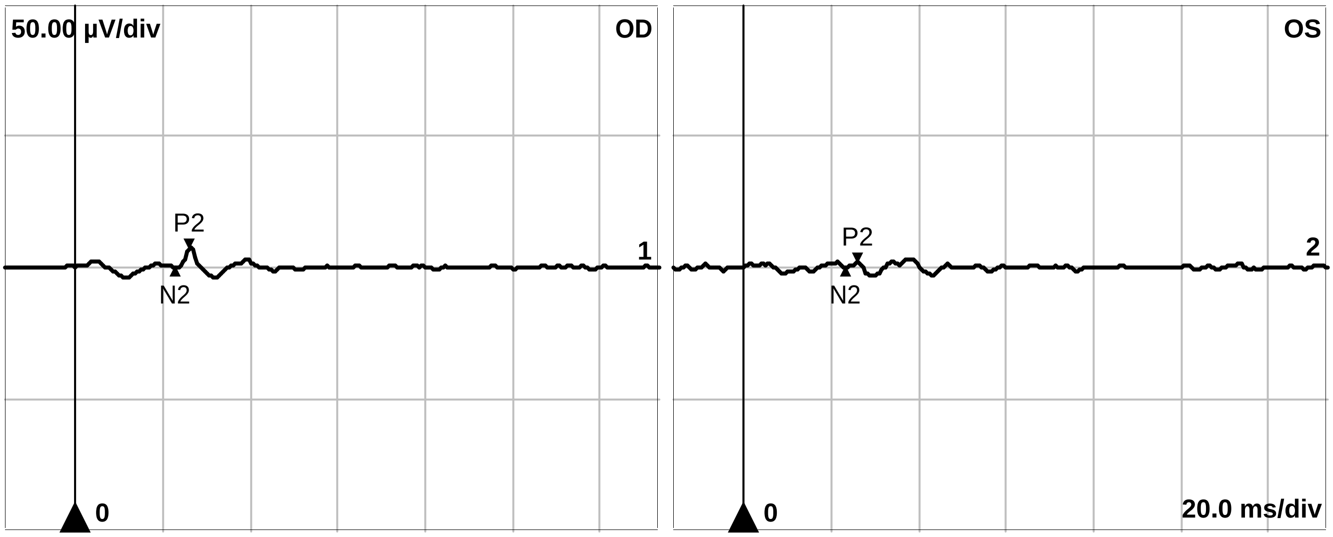

Step	P2 [ms]	OS2 [µV]	Avgs.
Normal	22.9-25.8	14.7-71.1	
1: OD-1	26.4 (!)	8.2 (!)	3
2: OS-2	26.4 (!)	2.0 (!)	3

4. Dark-adapted 10.0 ERG Stimulator: Ganzfeld

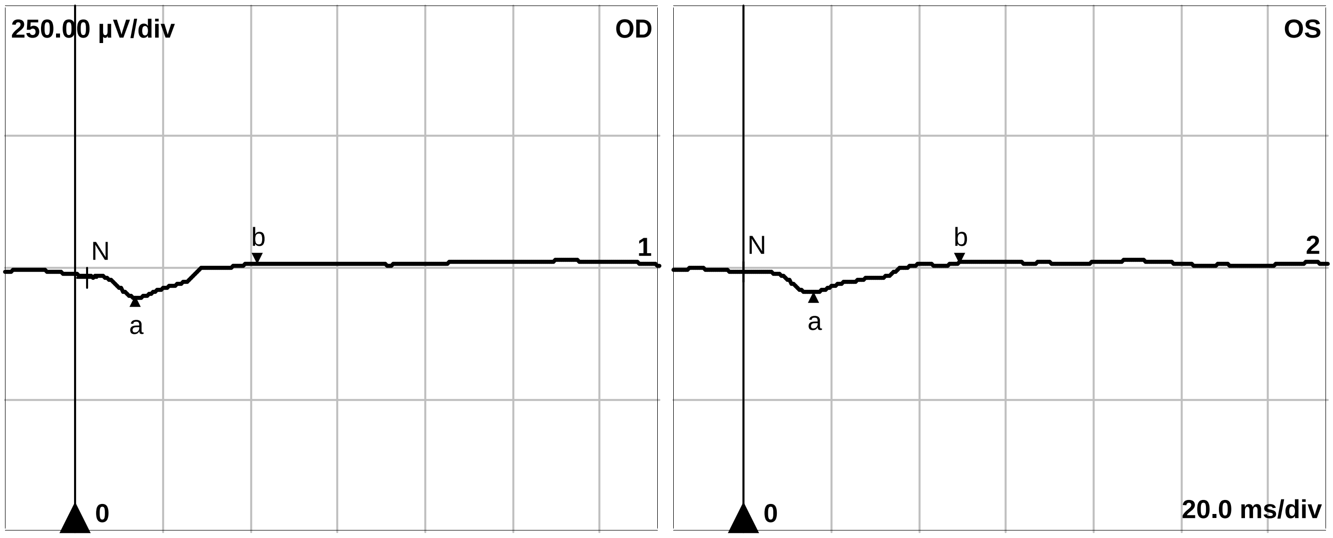

Step	a [ms]	b [ms]	a-wave [µV]	b-wave [µV]	b/a [µV]	Avgs.
Normal	12.9-17.0	32.6-50.8	135.0-322.0	180.0-550.0		
1: OD-1	14.1	41.6	40.7 (!)	69.0 (!)	1698119.5	5
2: OS-2	16.1	49.8	39.0 (!)	60.6 (!)	1552988.3	5

Patient:	I3, DTQ, 5/7/2021			Electrode:	DTL Thread
Examined:	12/6/2023 10:57	Sex/Age:	male/2	Dilated:	Yes
ID:		Operator:		Pupil Size:	-

1. Dark-adapted 0.01 ERG Stimulator: Ganzfeld

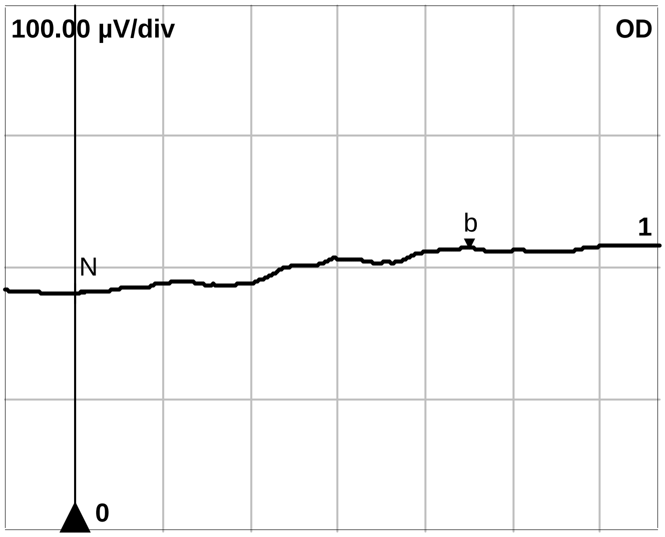

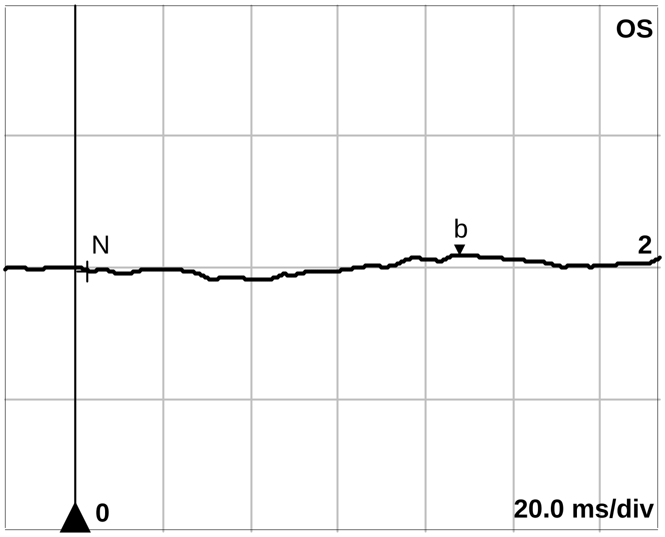

Step	b [ms]	b-wave [µV]	Avgs.
Normal	66.0-93.0	107.0-356.0	
1: OD-1	90.2	36.3 (!)	5
2: OS-2	88.2	13.0 (!)	5

2. Dark-adapted 3.0 ERG Stimulator: Ganzfeld

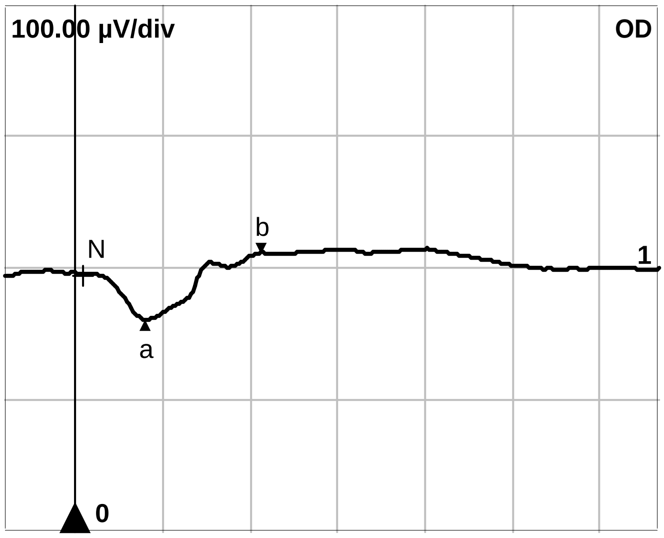

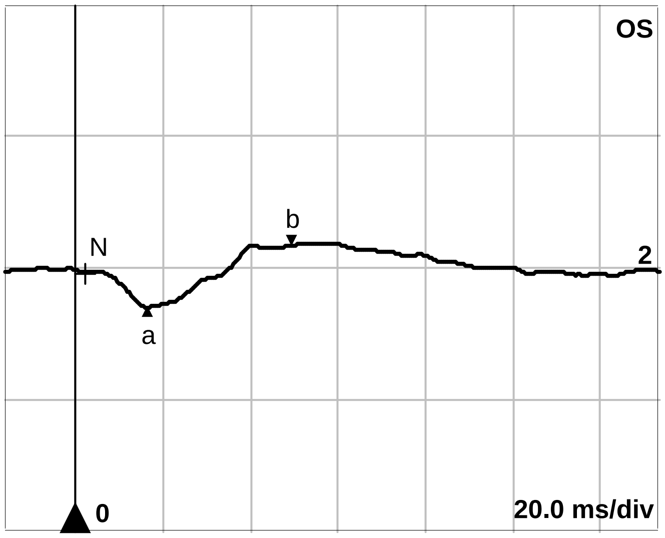

Step	a [ms]	b [ms]	a-wave [µV]	b-wave [µV]	b/a	Avgs.
Normal	15.3-19.4	32.9-58.7	118.0-315.0	168.0-527.0	1.5-2.6	
1: OD-1	16.1	42.8	34.9 (!)	52.7 (!)	1.5	5
2: OS-2	16.4	49.8	25.9 (!)	48.1 (!)	1.9	5

3. Dark-adapted 3.0 OPs Stimulator: Ganzfeld

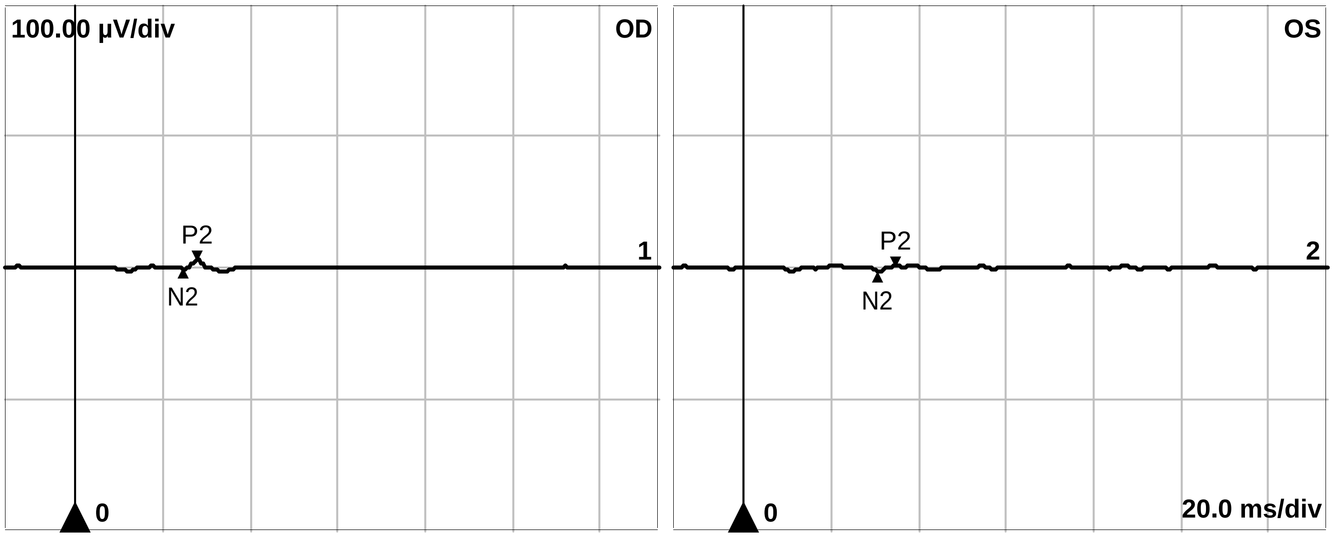


```
Step         P2 [ms]   OS2 [µV]   Avgs.
Normal     22.9-25.8  14.7-71.1
1: OD-1     28.1 (!)    8.3 (!)    3
2: OS-2     34.9 (!)    6.1 (!)    3
```

4. Dark-adapted 10.0 ERG Stimulator: Ganzfeld

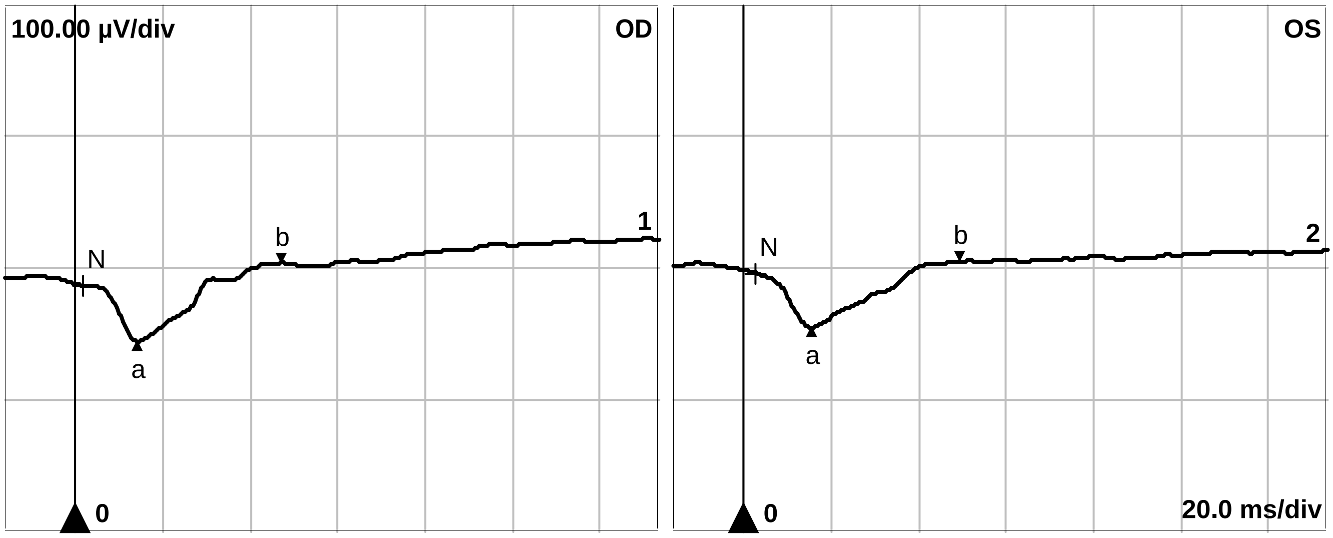


```
Step         a [ms]     b [ms]     a-wave [µV]  b-wave [µV]  b/a [µV]   Avgs.
Normal     12.9-17.0  32.6-50.8  135.0-322.0  180.0-550.0
1: OD-1     14.4       47.5        42.3 (!)     61.2 (!)    1444585.0    5
2: OS-2     15.5       49.8        41.6 (!)     51.8 (!)    1243732.9    5
```

Patient:	I-NC1, **DTQ, 5/7/2021**			Electrode:	DTL Thread
Examined:	12/5/2023 12:15	Sex/Age:	male/2	Dilated:	Yes
ID:		Operator:		Pupil Size:	-

1. Dark-adapted 0.01 ERG Stimulator: Ganzfeld

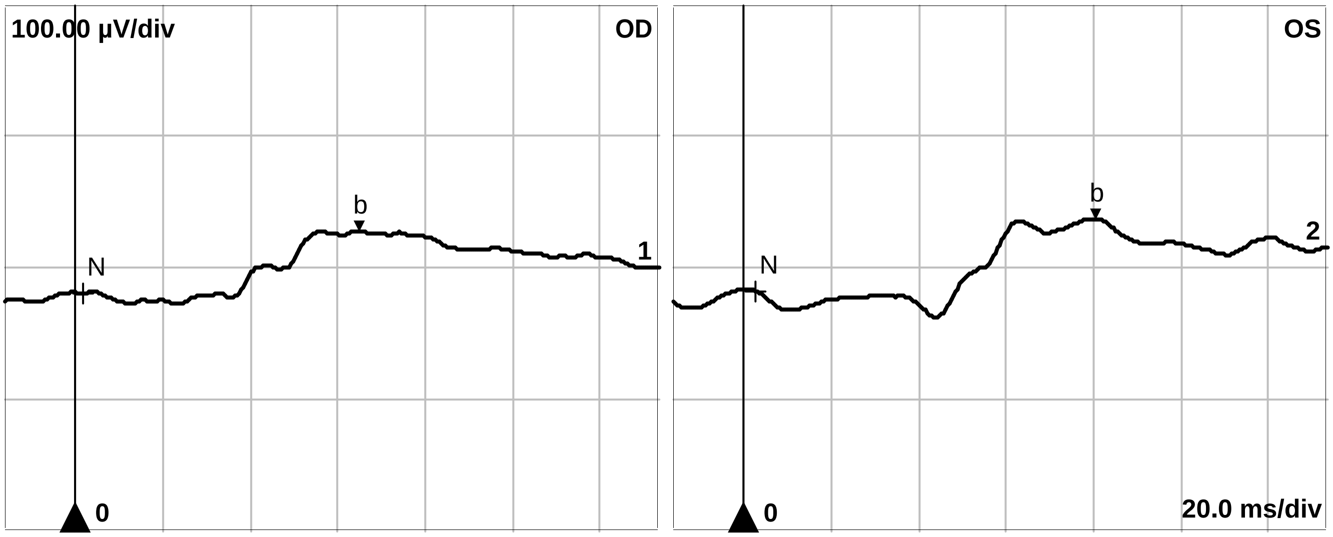

Step	b [ms]	b-wave [µV]	Avgs.
Normal	66.0-93.0	107.0-356.0	
1: OD-1	65.0 (!)	49.1 (!)	5
2: OS-2	80.6	55.9 (!)	5

2. Dark-adapted 3.0 ERG Stimulator: Ganzfeld

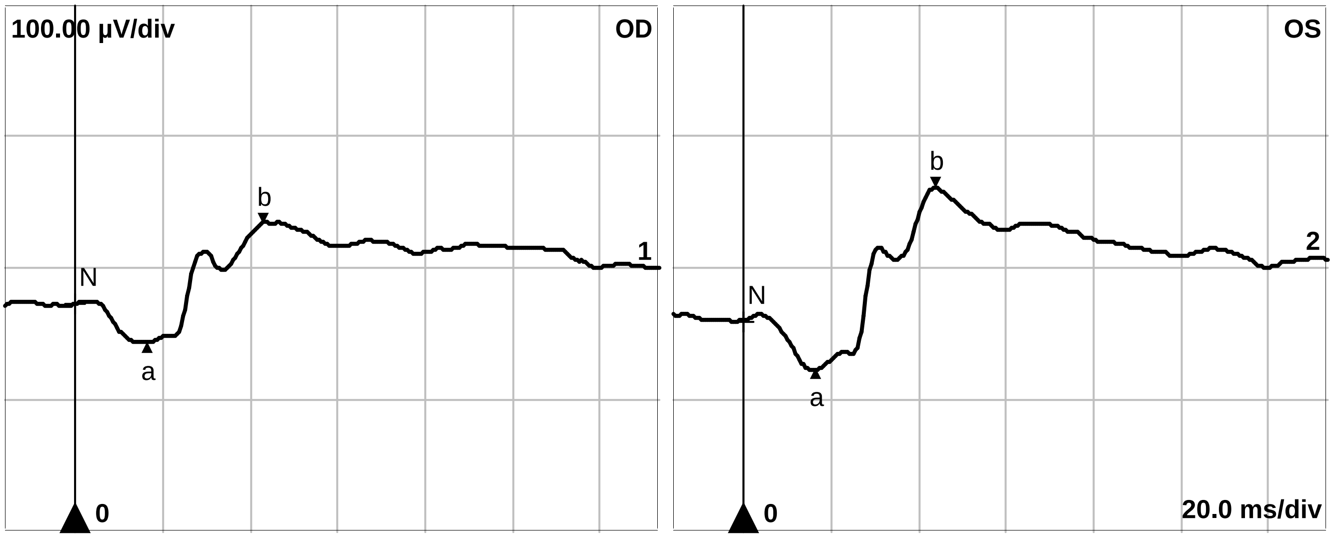

Step	a [ms]	b [ms]	a-wave [µV]	b-wave [µV]	b/a	Avgs.
Normal	15.3-19.4	32.9-58.7	118.0-315.0	168.0-527.0	1.5-2.6	
1: OD-1	16.7	43.4	29.2 (!)	93.0 (!)	3.2 (!)	5
2: OS-2	16.4	43.9	37.1 (!)	139.6 (!)	3.8 (!)	5

Patient:	I-NC1, DTQ, 5/7/2021			Electrode:	DTL Thread
Examined:	12/5/2023 12:15	Sex/Age:	male/2	Dilated:	Yes
ID:		Operator:		Pupil Size:	-

3. Dark-adapted 3.0 OPs Stimulator: Ganzfeld

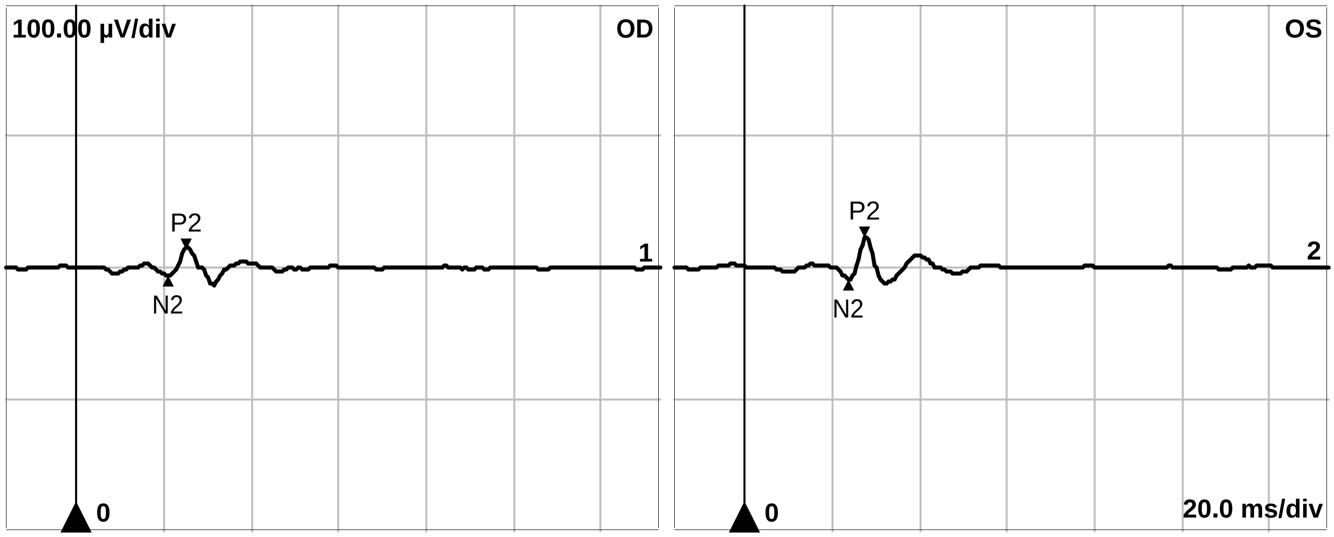

Step	P2 [ms]	OS2 [µV]	Avgs.
Normal	22.9-25.8	14.7-71.1	
1: OD-1	25.5	22.9	3
2: OS-2	27.8 (!)	33.8	3

4. Dark-adapted 10.0 ERG Stimulator: Ganzfeld

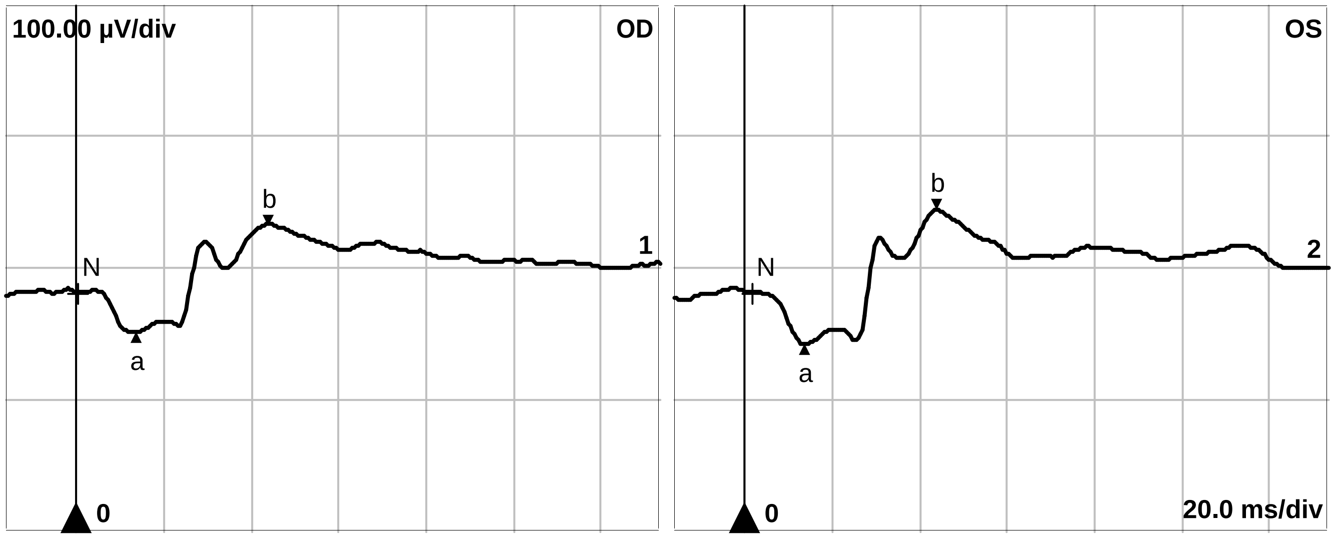

Step	a [ms]	b [ms]	a-wave [µV]	b-wave [µV]	b/a [µV]	Avgs.
Normal	12.9-17.0	32.6-50.8	135.0-322.0	180.0-550.0		
1: OD-1	13.8	44.2	30.0 (!)	83.9 (!)	2795449.5	5
2: OS-2	14.1	43.9	39.7 (!)	104.1 (!)	2620270.3	5

RETIport	Enter Clinic ID<br>Enter your address!					
	Patient:	I-N-2**, DTQ, 5/7/2021**			Electrode:	DTL Thread
	Examined:	12/6/2023 11:09	Sex/Age:	male/2	Dilated:	Yes
	ID:		Operator:		Pupil Size:	-

1. Dark-adapted 0.01 ERG Stimulator: Ganzfeld

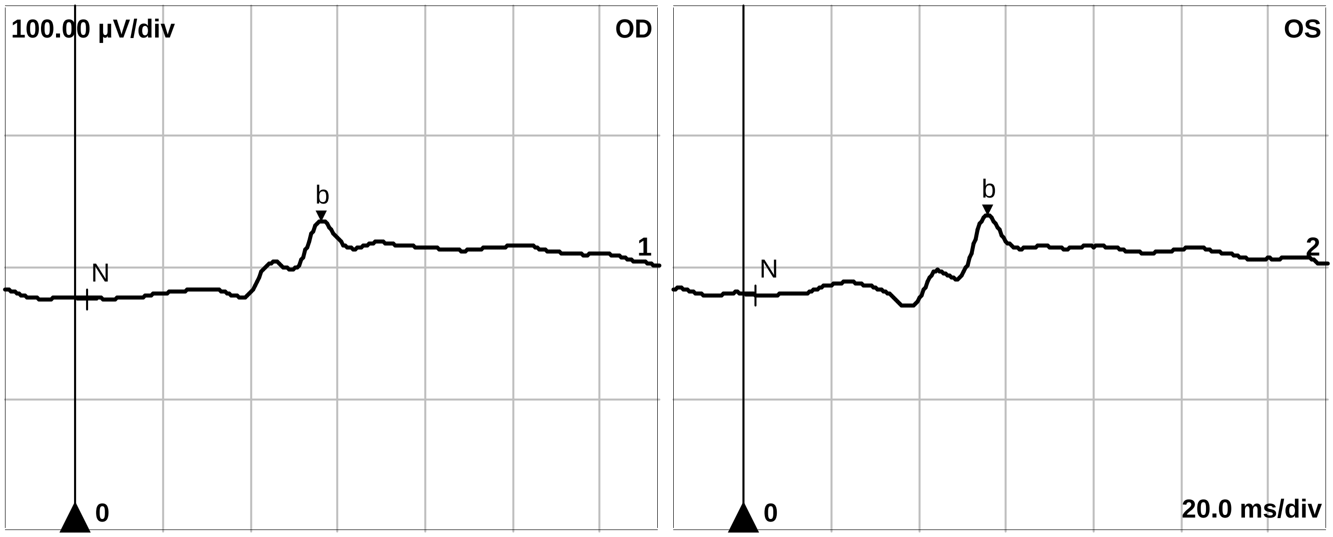

Step	b [ms]	b-wave [µV]	Avgs.
Normal	66.0-93.0	107.0-356.0	
1: OD-1	56.5 (!)	60.0 (!)	5
2: OS-2	56.0 (!)	62.4 (!)	5

2. Dark-adapted 3.0 ERG Stimulator: Ganzfeld

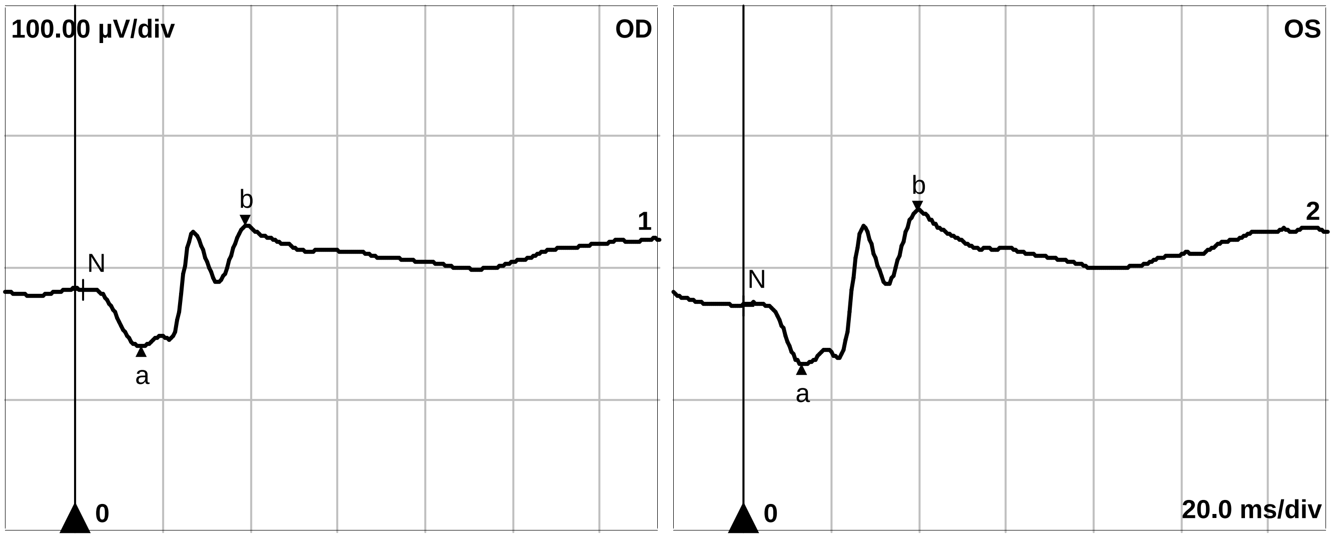

Step	a [ms]	b [ms]	a-wave [µV]	b-wave [µV]	b/a	Avgs.
Normal	15.3-19.4	32.9-58.7	118.0-315.0	168.0-527.0	1.5-2.6	
1: OD-1	15.2 (!)	39.3	43.1 (!)	92.9 (!)	2.2	5
2: OS-2	13.5 (!)	40.1	45.3 (!)	118.3 (!)	2.6 (!)	5

Patient:	I-N-2, DTQ, 5/7/2021			Electrode:	DTL Thread
Examined:	12/6/2023 11:09	Sex/Age:	male/2	Dilated:	Yes
ID:		Operator:		Pupil Size:	-

3. Dark-adapted 3.0 OPs Stimulator: Ganzfeld

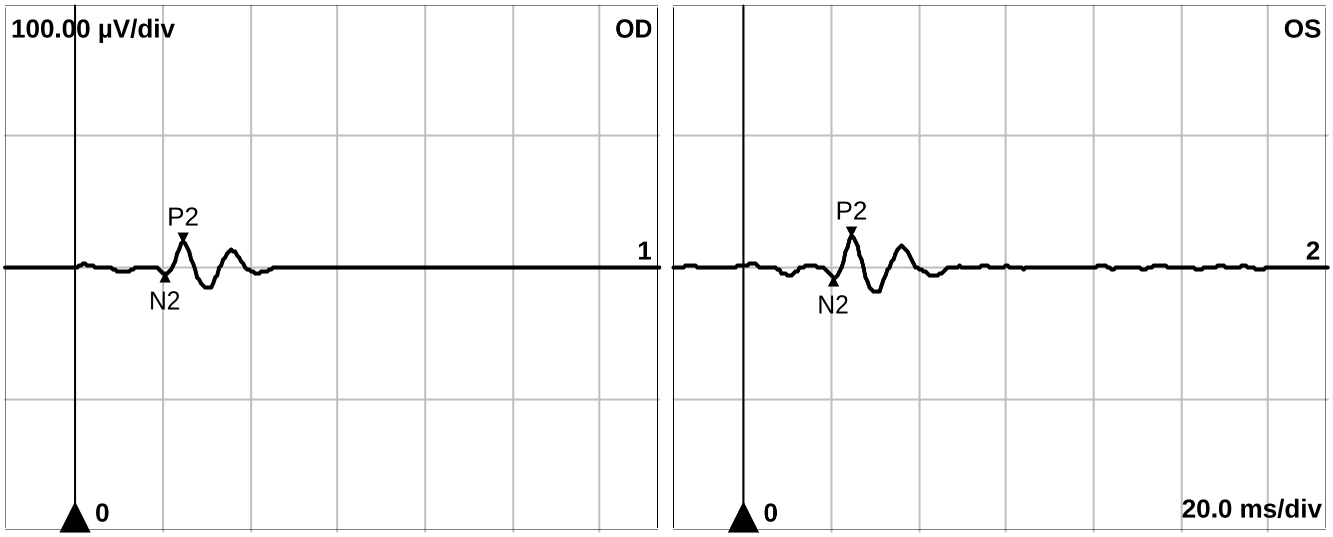

Step	P2 [ms]	OS2 [µV]	Avgs.
Normal	22.9-25.8	14.7-71.1	
1: OD-1	24.9	25.5	3
2: OS-2	24.9	32.6	3

4. Dark-adapted 10.0 ERG Stimulator: Ganzfeld

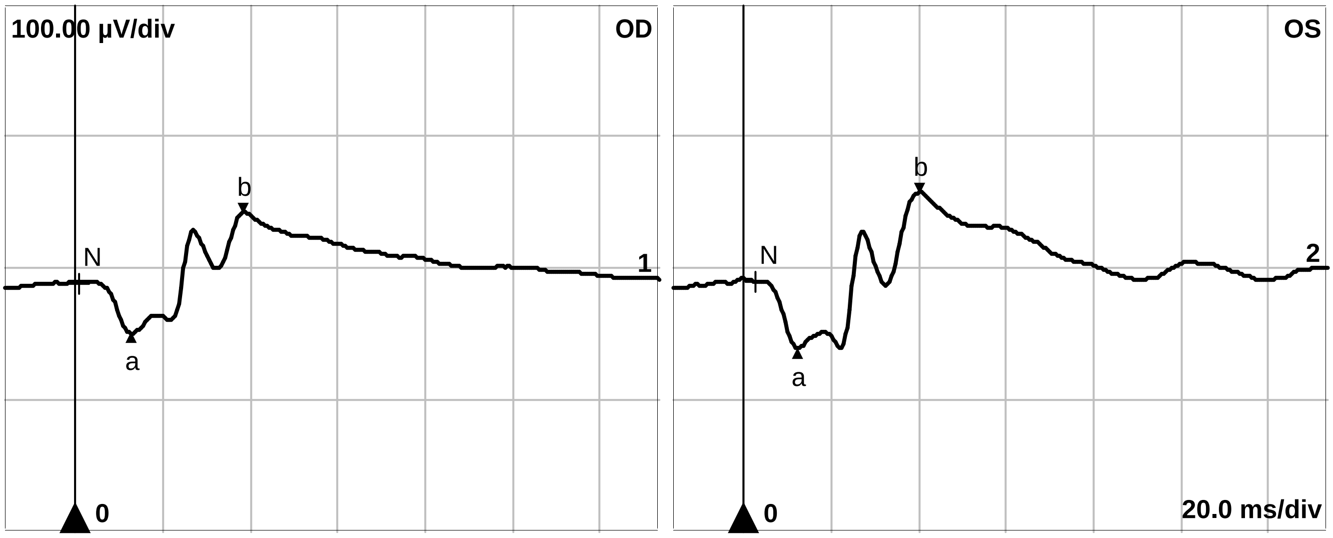

Step	a [ms]	b [ms]	a-wave [µV]	b-wave [µV]	b/a [µV]	Avgs.
Normal	12.9-17.0	32.6-50.8	135.0-322.0	180.0-550.0		
1: OD-1	12.9 (!)	38.7	38.5 (!)	93.4 (!)	2423389.9	5
2: OS-2	12.6 (!)	40.4	50.7 (!)	120.0 (!)	2365772.2	5

Patient:	I-N-3, **DTQ, 5/7/2021**			Electrode:	DTL Thread
Examined:	12/6/2023 11:23	Sex/Age:	male/2	Dilated:	Yes
ID:		Operator:		Pupil Size:	-

1. Dark-adapted 0.01 ERG Stimulator: Ganzfeld

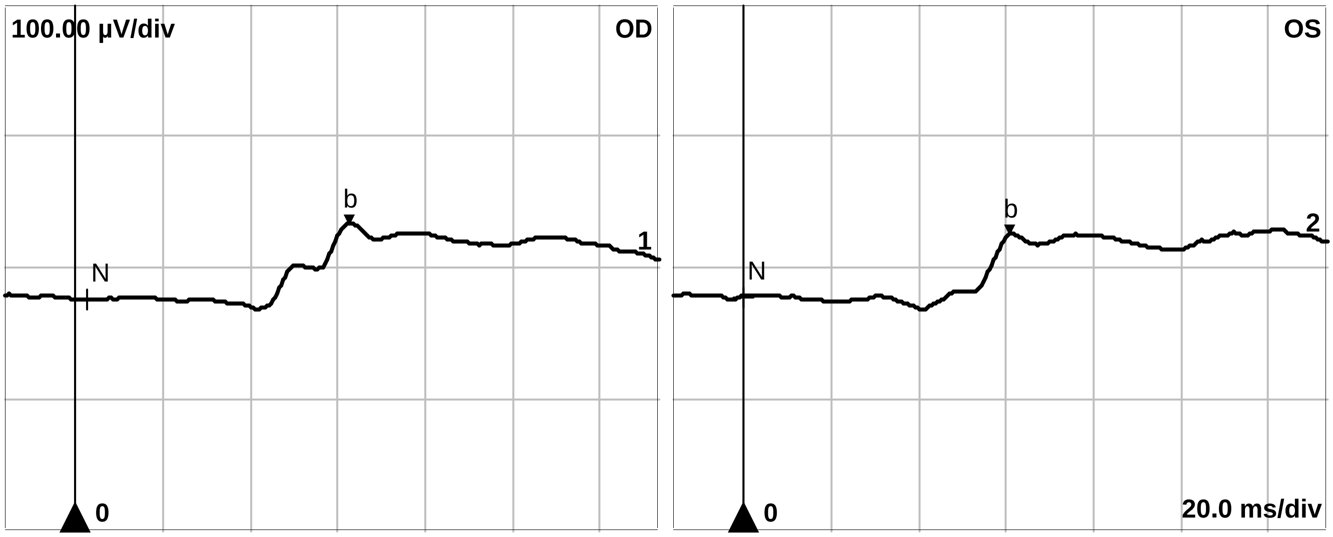

Step	b [ms]	b-wave [µV]	Avgs.
Normal	66.0-93.0	107.0-356.0	
1: OD-1	63.0 (!)	58.9 (!)	5
2: OS-2	61.2 (!)	48.5 (!)	5

2. Dark-adapted 3.0 ERG Stimulator: Ganzfeld

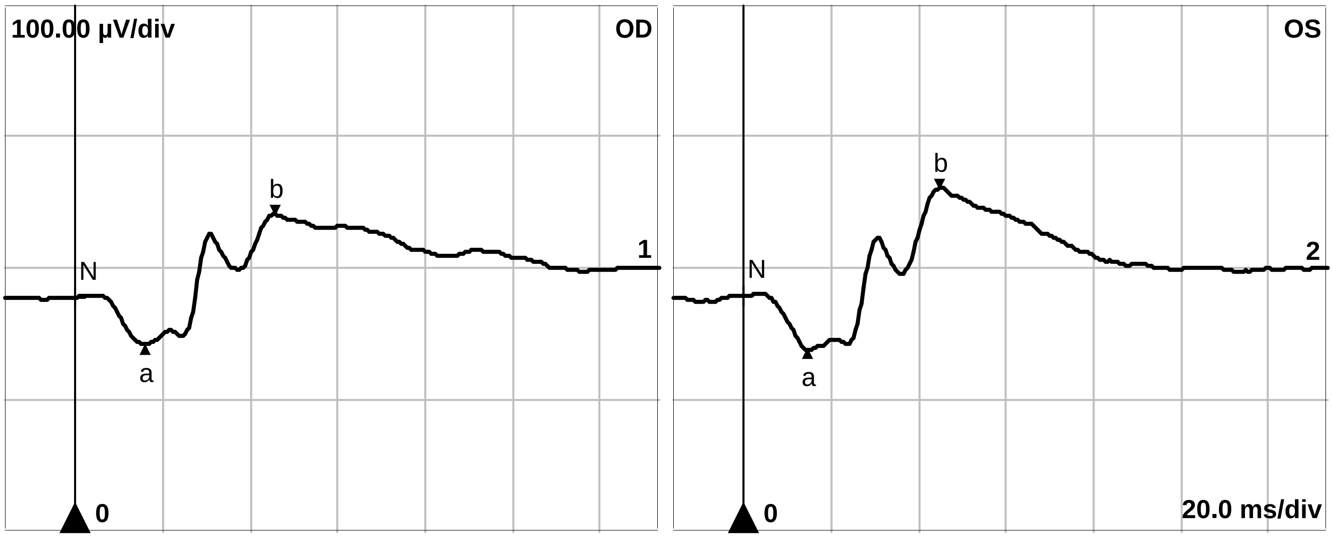

Step	a [ms]	b [ms]	a-wave [µV]	b-wave [µV]	b/a	Avgs.
Normal	15.3-19.4	32.9-58.7	118.0-315.0	168.0-527.0	1.5-2.6	
1: OD-1	16.1	45.7	35.9 (!)	100.2 (!)	2.8 (!)	5
2: OS-2	14.9 (!)	45.1	40.8 (!)	124.0 (!)	3.0 (!)	5

3. Dark-adapted 3.0 OPs Stimulator: Ganzfeld

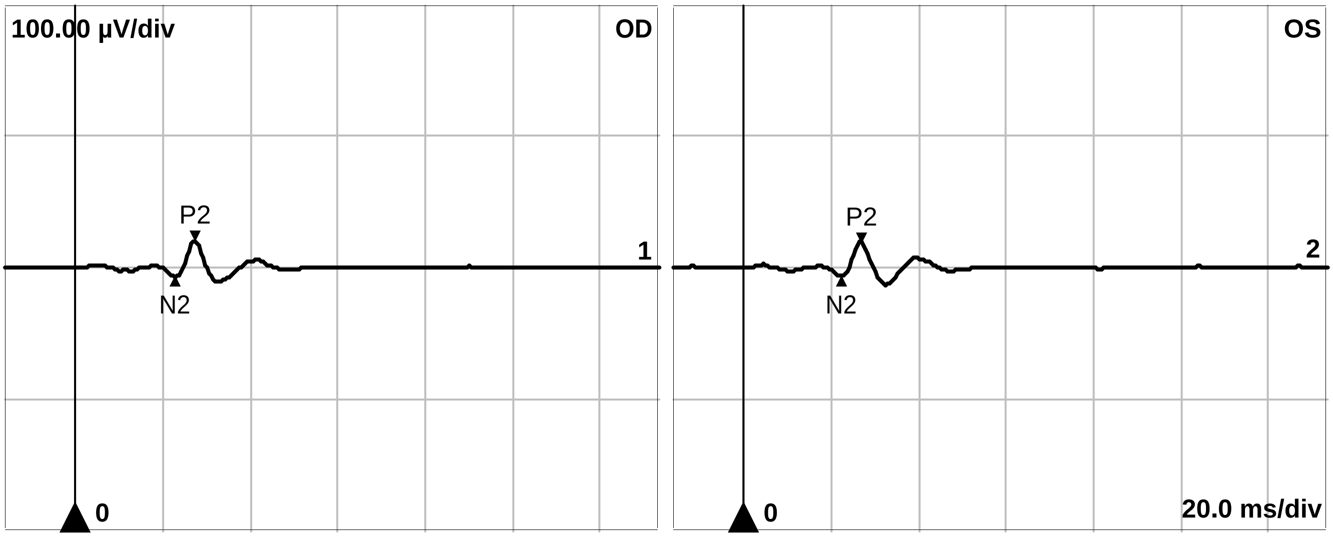

Step	P2 [ms]	OS2 [µV]	Avgs.
Normal	22.9-25.8	14.7-71.1	
1: OD-1	27.5 (!)	29.0	6
2: OS-2	27.0 (!)	27.3	6

4. Dark-adapted 10.0 ERG Stimulator: Ganzfeld

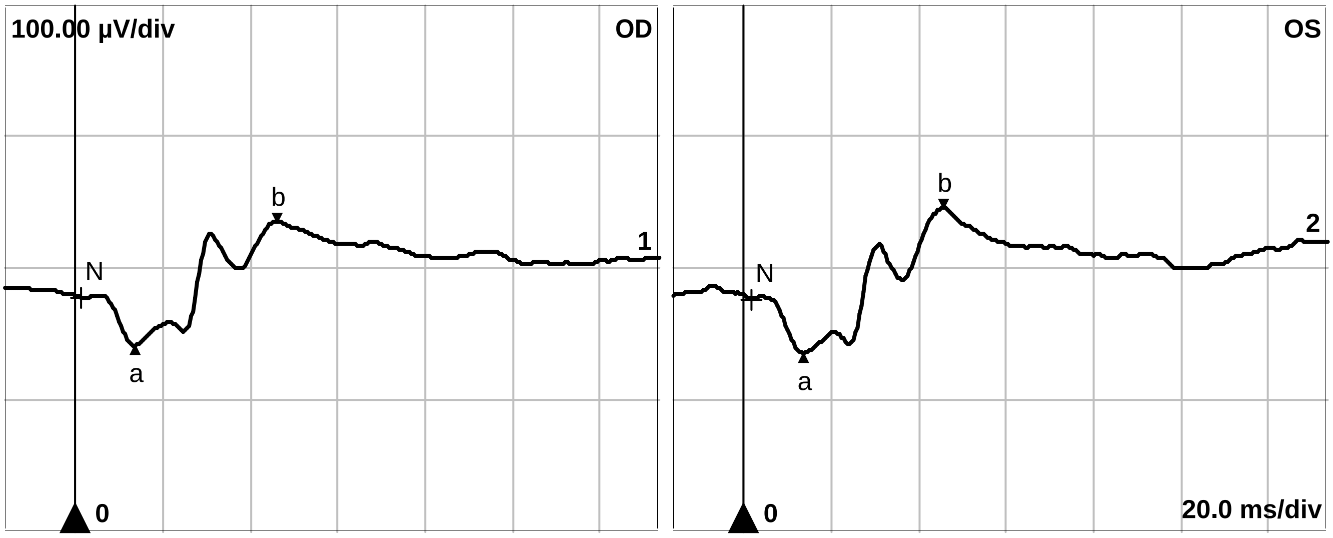

Step	a [ms]	b [ms]	a-wave [µV]	b-wave [µV]	b/a [µV]	Avgs.
Normal	12.9-17.0	32.6-50.8	135.0-322.0	180.0-550.0		
1: OD-1	13.8	46.6	36.8 (!)	95.1 (!)	2580839.9	5
2: OS-2	13.8	45.7	41.3 (!)	111.7 (!)	2703569.4	5

RETIport	Enter Clinic ID<br>Enter your address!					
	Patient:	M1, DTQ, 1/3/2024			Electrode:	DTL Thread
	Examined:	1/3/2024 13:55	Sex/Age:	male/0 days	Dilated:	Yes
	ID:		Operator:		Pupil Size:	-

1. Dark-adapted 0.01 ERG Stimulator: Ganzfeld

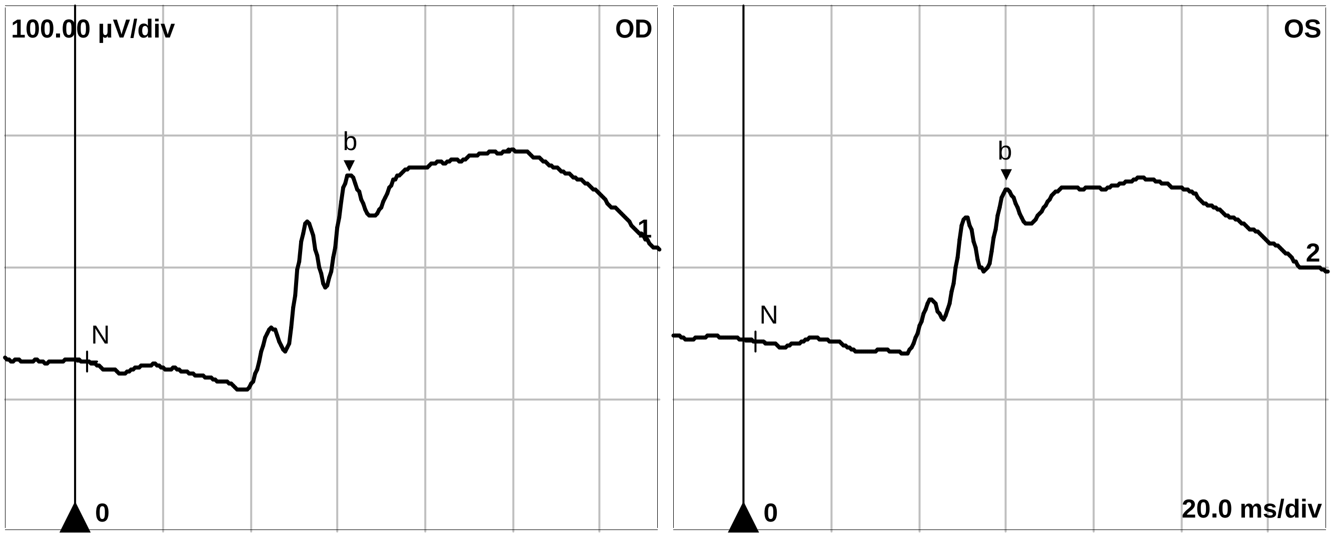

Step	b [ms]	b-wave [µV]	Avgs.
Normal			
1: OD-1	69.9	161.8	5
2: OS-2	60.8	126.4	5

2. Dark-adapted 3.0 ERG Stimulator: Ganzfeld

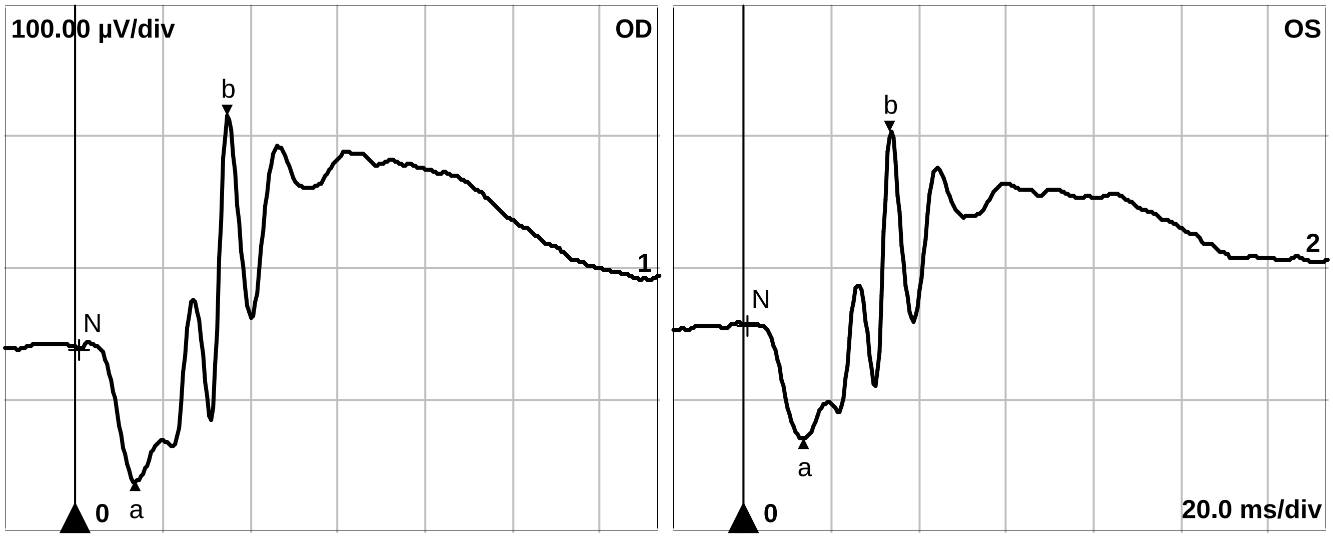

Step	a [ms]	b [ms]	a-wave [µV]	b-wave [µV]	b/a	Avgs.
Normal						
1: OD-1	13.8	35.2	101.7	280.2	2.8	5
2: OS-2	13.8	33.7	86.5	234.6	2.7	5

Patient:	M1, DTQ, 1/3/2024			Electrode:	DTL Thread
Examined:	1/3/2024 13:55	Sex/Age:	male/0 days	Dilated:	Yes
ID:		Operator:		Pupil Size:	-

3. Dark-adapted 3.0 OPs Stimulator: Ganzfeld

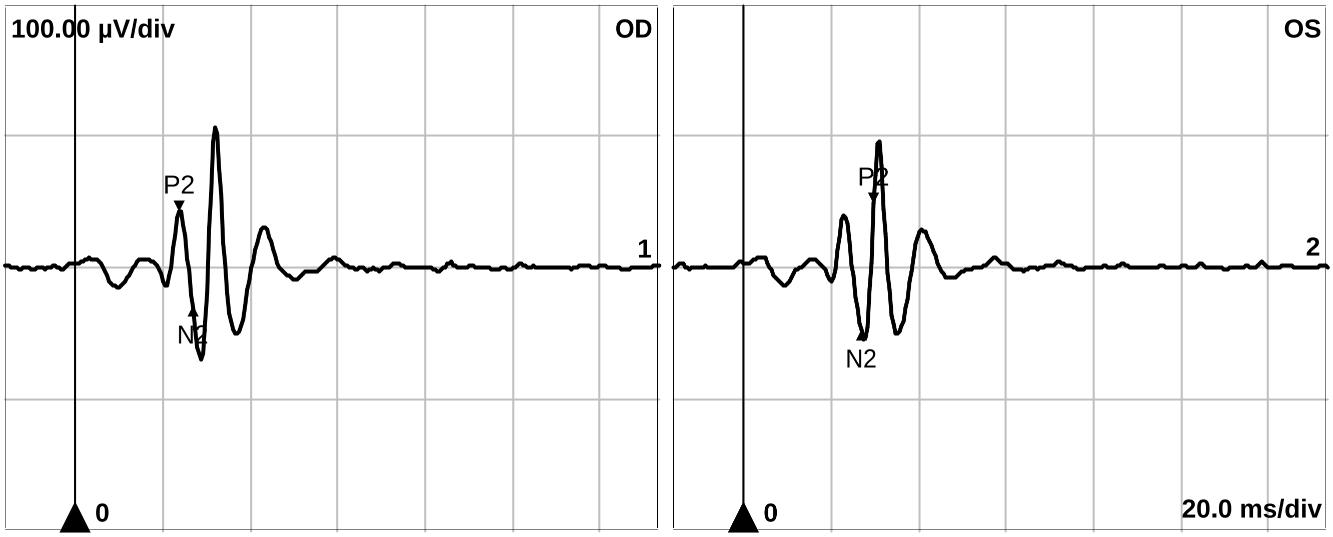

Step	P2 [ms]	OS2 [µV]	Avgs.
Normal			
1: OD-1	24.0	75.3	3
2: OS-2	29.9	98.7	3

4. Dark-adapted 10.0 ERG Stimulator: Ganzfeld

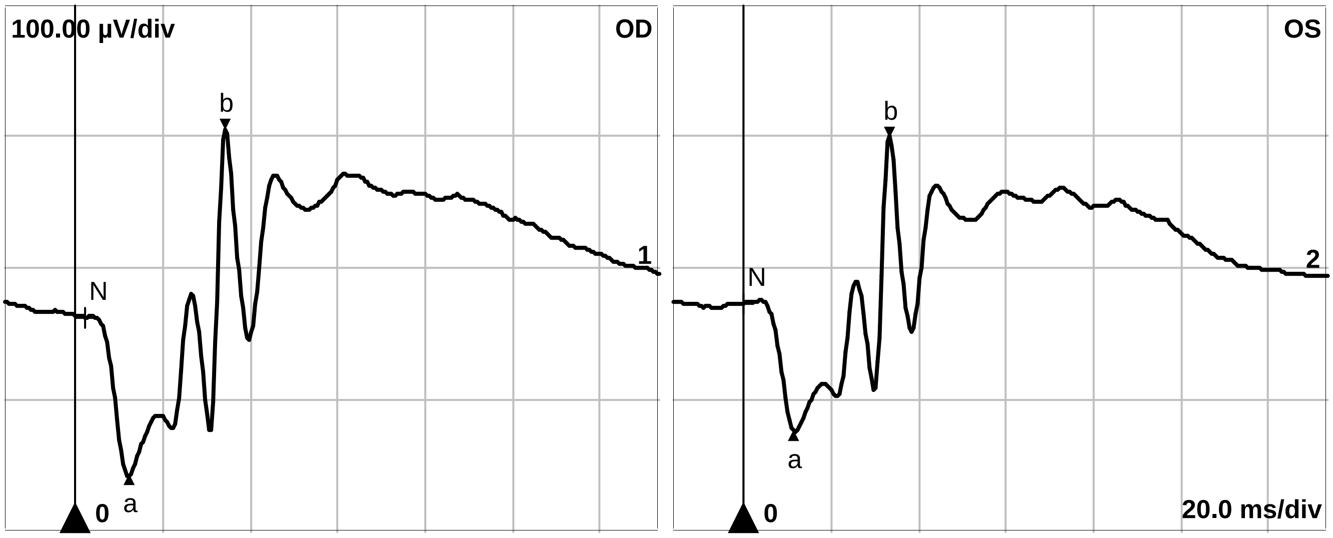

Step	a [ms]	b [ms]	a-wave [µV]	b-wave [µV]	b/a [µV]	Avgs.
Normal						
1: OD-1	12.3	34.6	120.8	265.6	2199394.0	5
2: OS-2	11.7	33.4	97.7	225.8	2310969.6	5

RETIport	Enter Clinic ID<br>Enter your address!			
	Patient: **M2, DTQ, 1/3/2024**		Electrode:	DTL Thread
	Examined: 1/3/2024 13:34	Sex/Age: male/0 days	Dilated:	Yes
	ID:	Operator:	Pupil Size:	-

1. Dark-adapted 0.01 ERG Stimulator: Ganzfeld

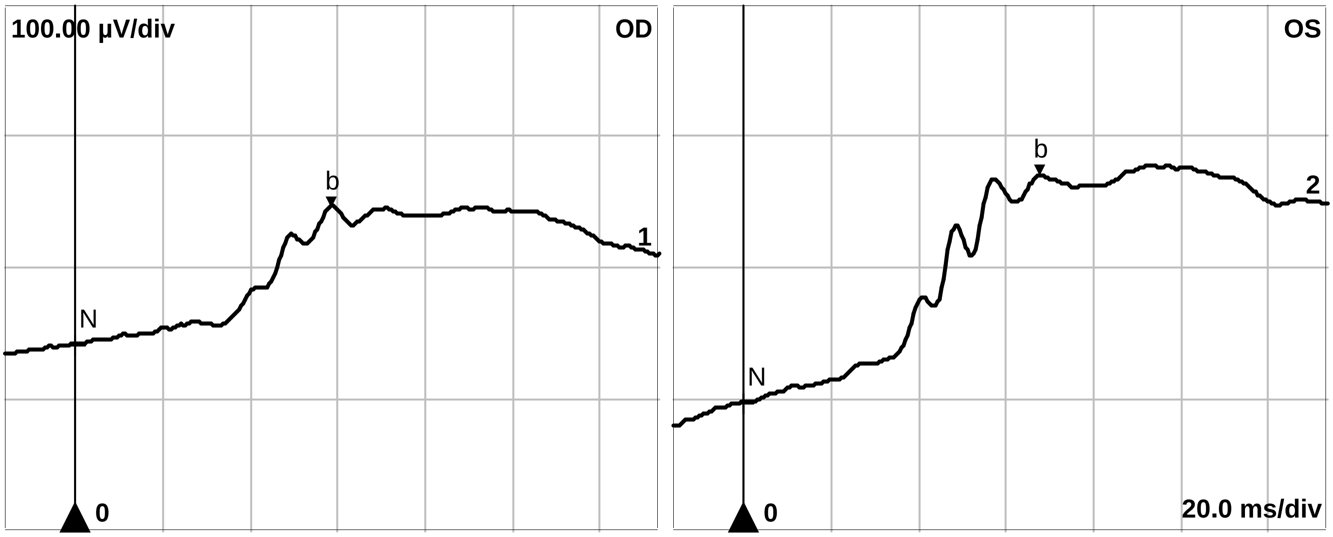

Step	b [ms]	b-wave [µV]	Avgs.
Normal			
1: OD-1	58.9	106.5	5
2: OS-2	67.7	173.9	5

2. Dark-adapted 3.0 ERG Stimulator: Ganzfeld

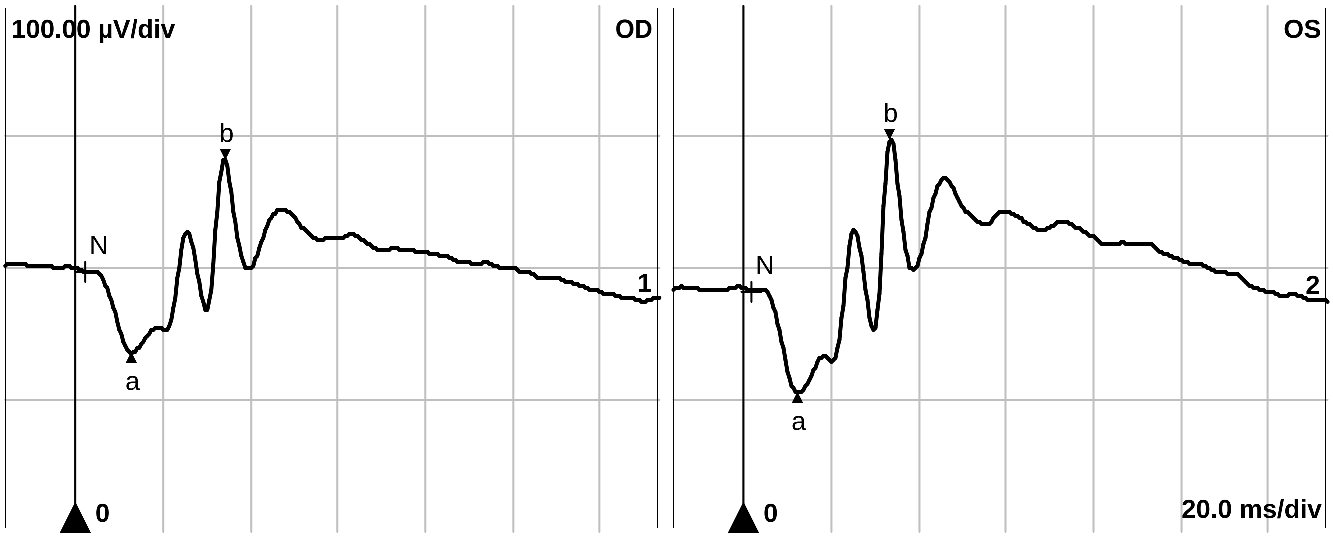

Step	a [ms]	b [ms]	a-wave [µV]	b-wave [µV]	b/a	Avgs.
Normal						
1: OD-1	12.9	34.3	61.9	148.6	2.4	5
2: OS-2	12.6	33.7	77.7	194.7	2.5	5

3. Dark-adapted 3.0 OPs Stimulator: Ganzfeld

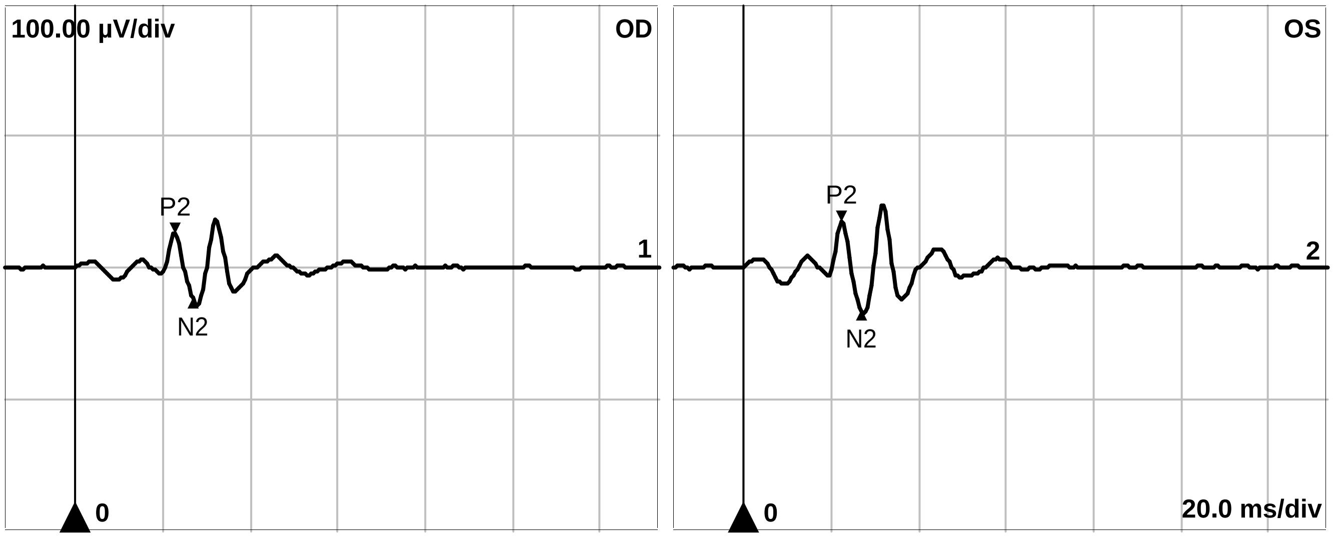

Step	P2 [ms]	OS2 [µV]	Avgs.
Normal			
1: OD-1	22.9	51.0	3
2: OS-2	22.6	69.3	3

4. Dark-adapted 10.0 ERG Stimulator: Ganzfeld

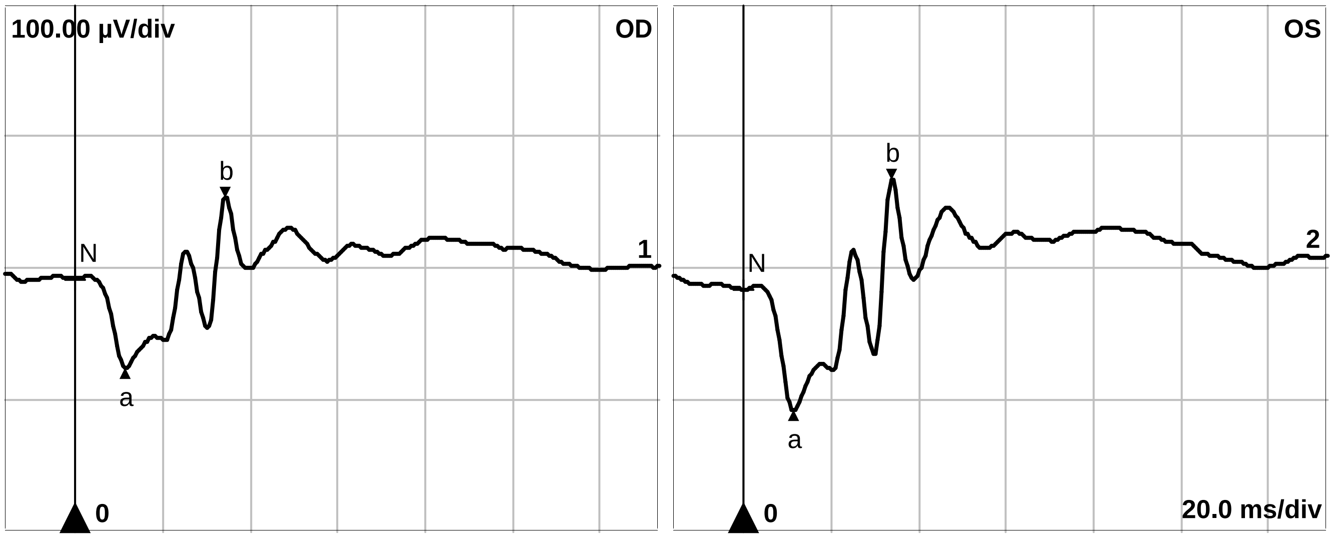

Step	a [ms]	b [ms]	a-wave [µV]	b-wave [µV]	b/a [µV]	Avgs.
Normal						
1: OD-1	11.7	34.6	68.5	132.4	1934072.7	5
2: OS-2	11.4	34.0	91.8	177.1	1929705.0	5

RETIport	Enter Clinic ID<br>Enter your address!			
	Patient: M3, DTQ, 1/3/2024		Electrode:	DTL Thread
	Examined: 1/3/2024 12:35	Sex/Age: male/0 days	Dilated:	Yes
	ID:	Operator:	Pupil Size:	-

1. Dark-adapted 0.01 ERG Stimulator: Ganzfeld

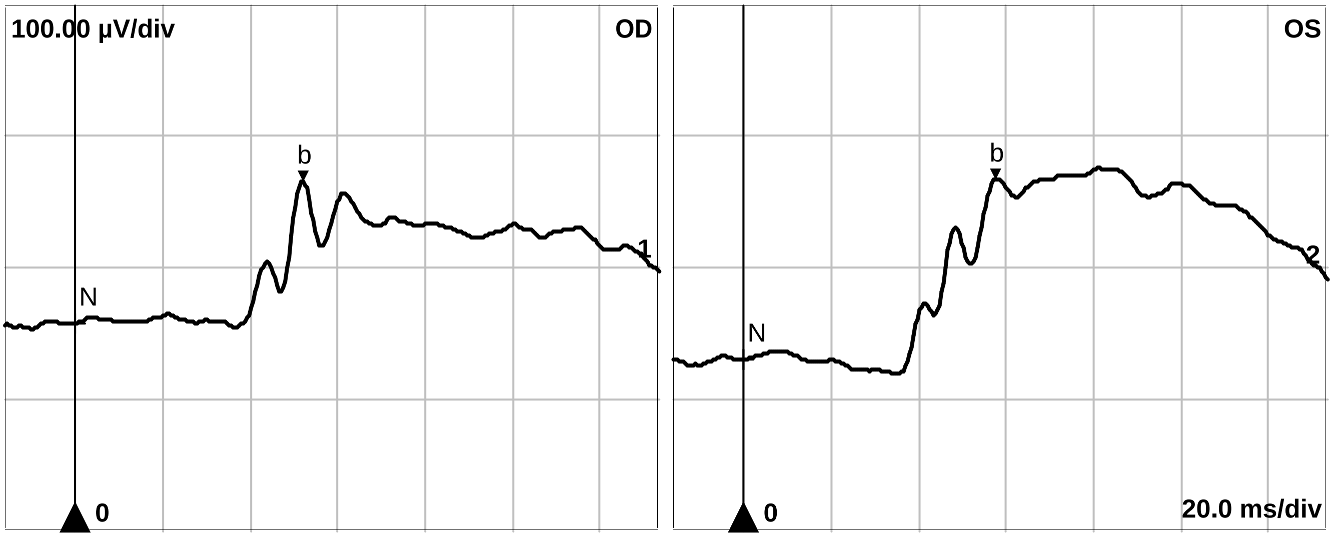

Step	b [ms]	b-wave [µV]	Avgs.
Normal			
1: OD-1	52.1	109.4	5
2: OS-2	57.7	138.7	5

2. Dark-adapted 3.0 ERG Stimulator: Ganzfeld

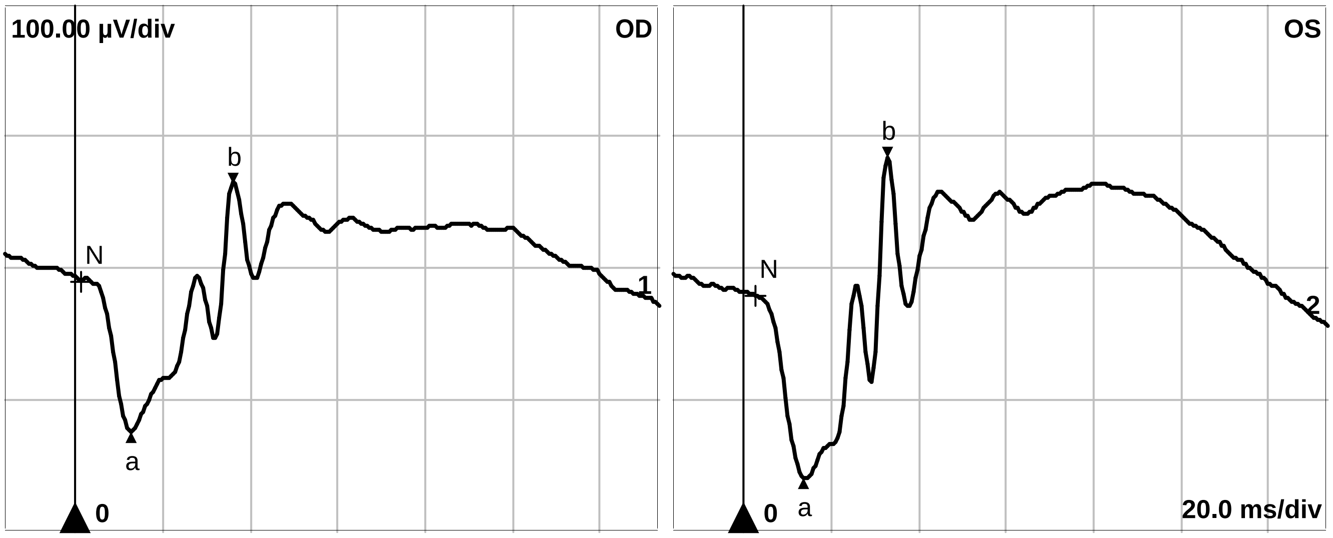

Step	a [ms]	b [ms]	a-wave [µV]	b-wave [µV]	b/a	Avgs.
Normal						
1: OD-1	12.9	36.3	115.5	191.1	1.7	5
2: OS-2	14.1	33.1	139.1	245.7	1.8	5

RETIport	Enter Clinic ID<br>Enter your address!			
	Patient: M3, DTQ, 1/3/2024		Electrode:	DTL Thread
	Examined: 1/3/2024 12:35	Sex/Age: male/0 days	Dilated:	Yes
	ID:	Operator:	Pupil Size:	-

3. Dark-adapted 3.0 OPs Stimulator: Ganzfeld

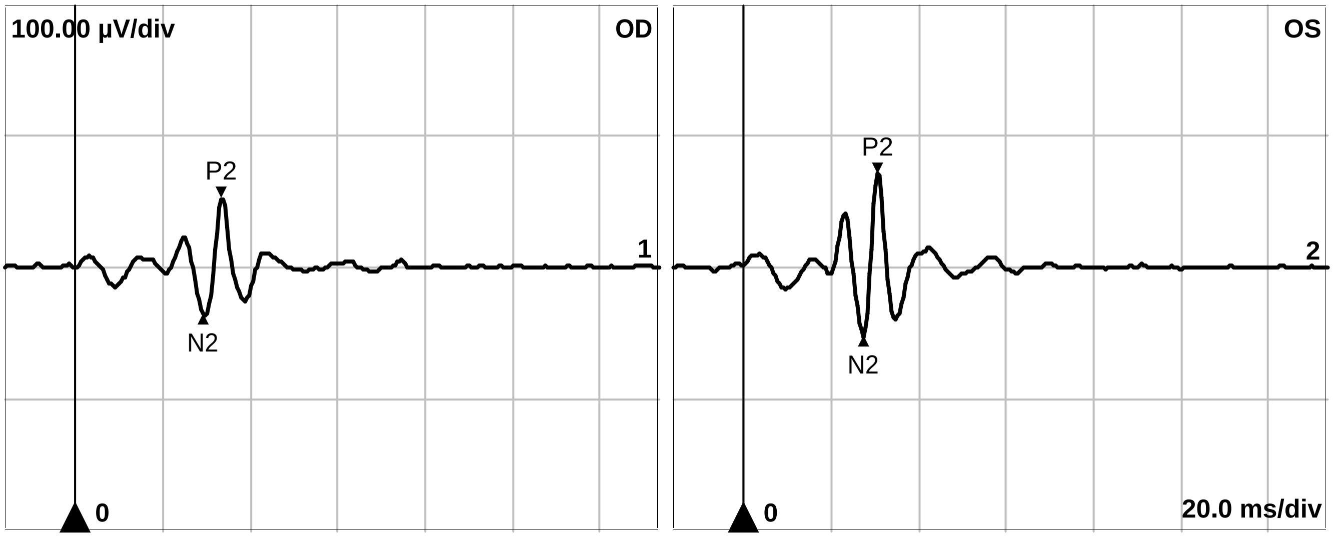

Step	P2 [ms]	OS2 [µV]	Avgs.
Normal			
1: OD-1	33.7	91.0	3
2: OS-2	30.8	126.2	3

4. Dark-adapted 10.0 ERG Stimulator: Ganzfeld

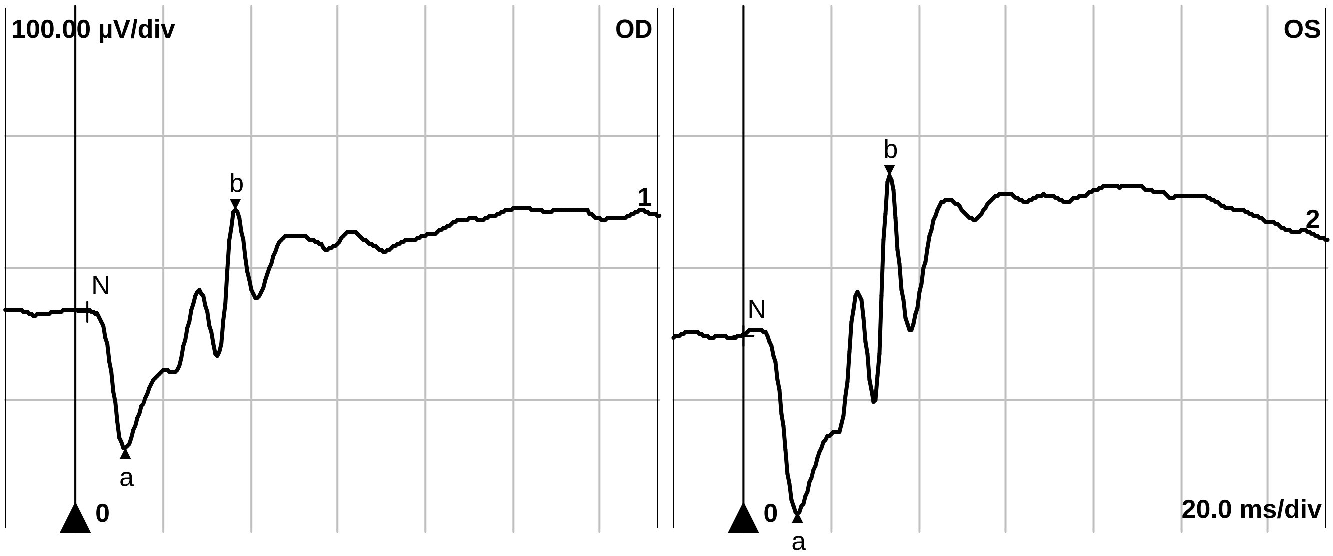

Step	a [ms]	b [ms]	a-wave [µV]	b-wave [µV]	b/a [µV]	Avgs.
Normal						
1: OD-1	11.4	36.6	105.1	183.4	1746064.1	5
2: OS-2	12.3	33.7	135.8	258.5	1903358.7	5

RETIport	Enter Clinic ID<br>Enter your address!				
	Patient: M-N-1, **DTQ, 5/7/2021**			Electrode:	DTL Thread
	Examined: 12/5/2023 11:21	Sex/Age:	male/2	Dilated:	Yes
	ID:	Operator:		Pupil Size:	-

1. Dark-adapted 0.01 ERG Stimulator: Ganzfeld

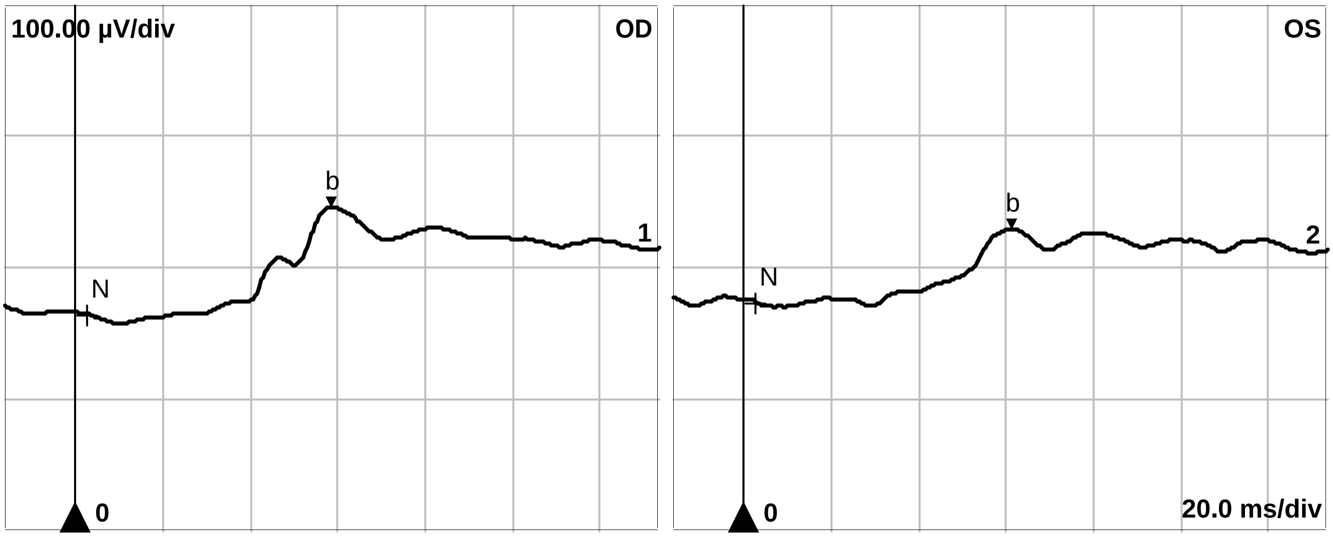

Step	b [ms]	b-wave [µV]	Avgs.
Normal	66.0-93.0	107.0-356.0	
1: OD-1	58.6 (!)	82.9 (!)	5
2: OS-2	61.5 (!)	57.3 (!)	5

2. Dark-adapted 3.0 ERG Stimulator: Ganzfeld

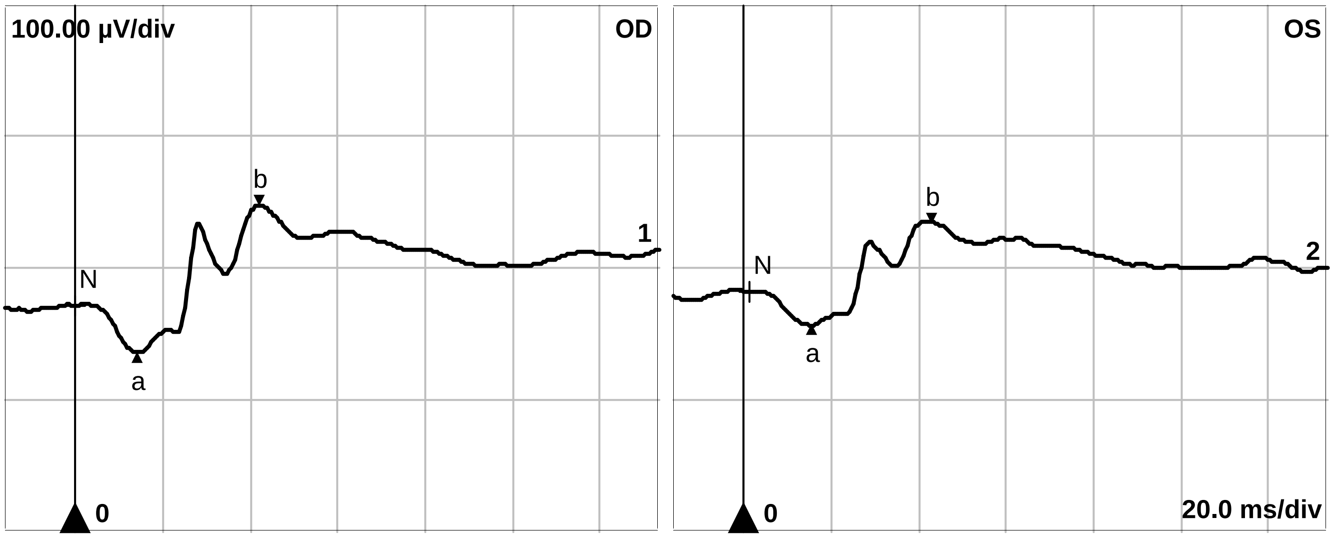

Step	a [ms]	b [ms]	a-wave [µV]	b-wave [µV]	b/a	Avgs.
Normal	15.3-19.4	32.9-58.7	118.0-315.0	168.0-527.0	1.5-2.6	
1: OD-1	14.4 (!)	42.5	35.9 (!)	113.5 (!)	3.2 (!)	5
2: OS-2	15.5	43.1	25.7 (!)	80.0 (!)	3.1 (!)	5

Patient:	M-N-1, DTQ, 5/7/2021			Electrode:	DTL Thread
Examined:	12/5/2023 11:21	Sex/Age:	male/2	Dilated:	Yes
ID:		Operator:		Pupil Size:	-

3. Dark-adapted 3.0 OPs Stimulator: Ganzfeld

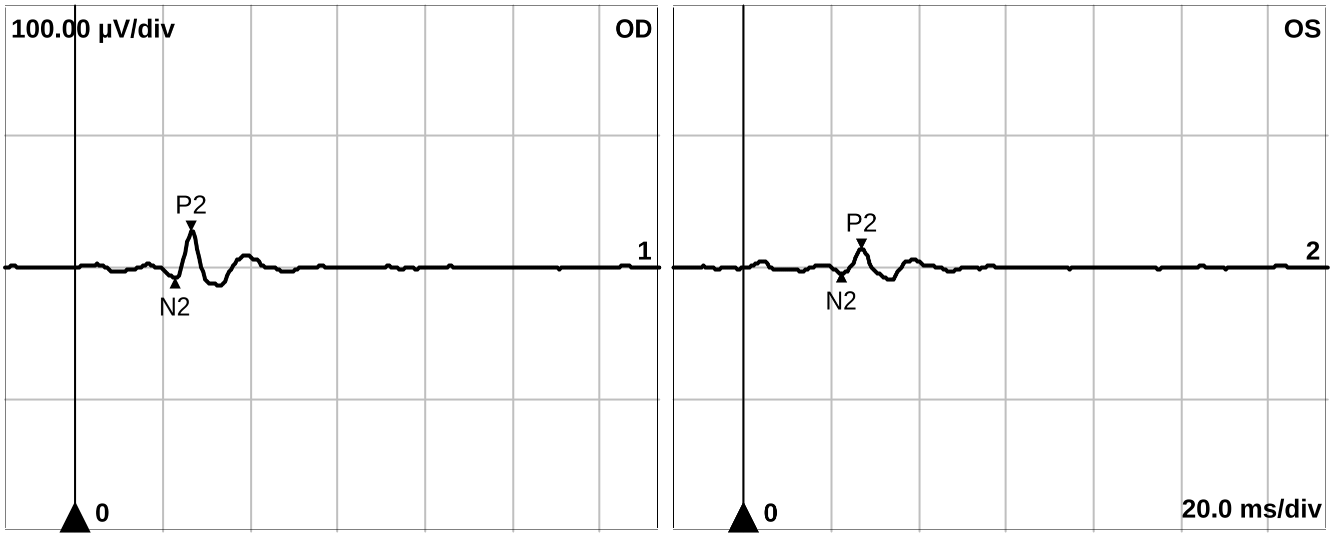

Step	P2 [ms]	OS2 [µV]	Avgs.
Normal	22.9-25.8	14.7-71.1	
1: OD-1	26.7 (!)	37.6	3
2: OS-2	27.2 (!)	20.2	3

4. Dark-adapted 10.0 ERG Stimulator: Ganzfeld

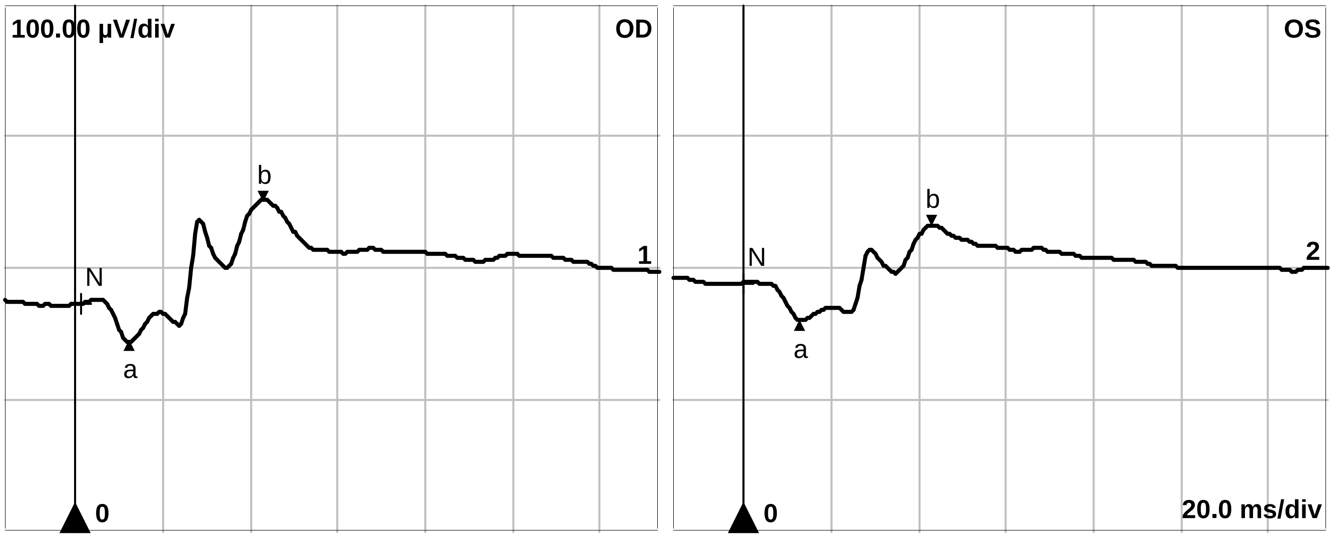

Step	a [ms]	b [ms]	a-wave [µV]	b-wave [µV]	b/a [µV]	Avgs.
Normal	12.9-17.0	32.6-50.8	135.0-322.0	180.0-550.0		
1: OD-1	12.6 (!)	43.4	29.2 (!)	109.1 (!)	3742802.1	5
2: OS-2	13.2	43.4	28.5 (!)	73.7 (!)	2582350.7	5

Patient:	M-N-2**, DTQ, 5/7/2021**			Electrode:	DTL Thread
Examined:	12/6/2023 10:17	Sex/Age:	male/2	Dilated:	Yes
ID:		Operator:		Pupil Size:	-

1. Dark-adapted 0.01 ERG Stimulator: Ganzfeld

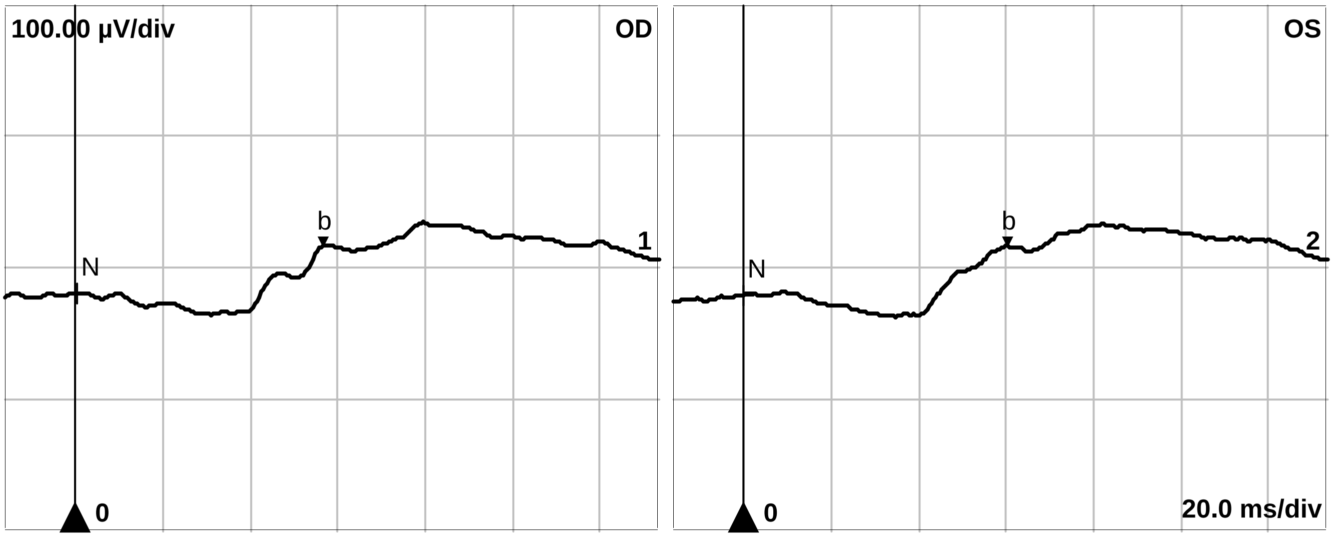

Step	b [ms]	b-wave [µV]	Avgs.
Normal	66.0-93.0	107.0-356.0	
1: OD-1	56.8 (!)	37.6 (!)	5
2: OS-2	60.6 (!)	38.2 (!)	5

2. Dark-adapted 3.0 ERG Stimulator: Ganzfeld

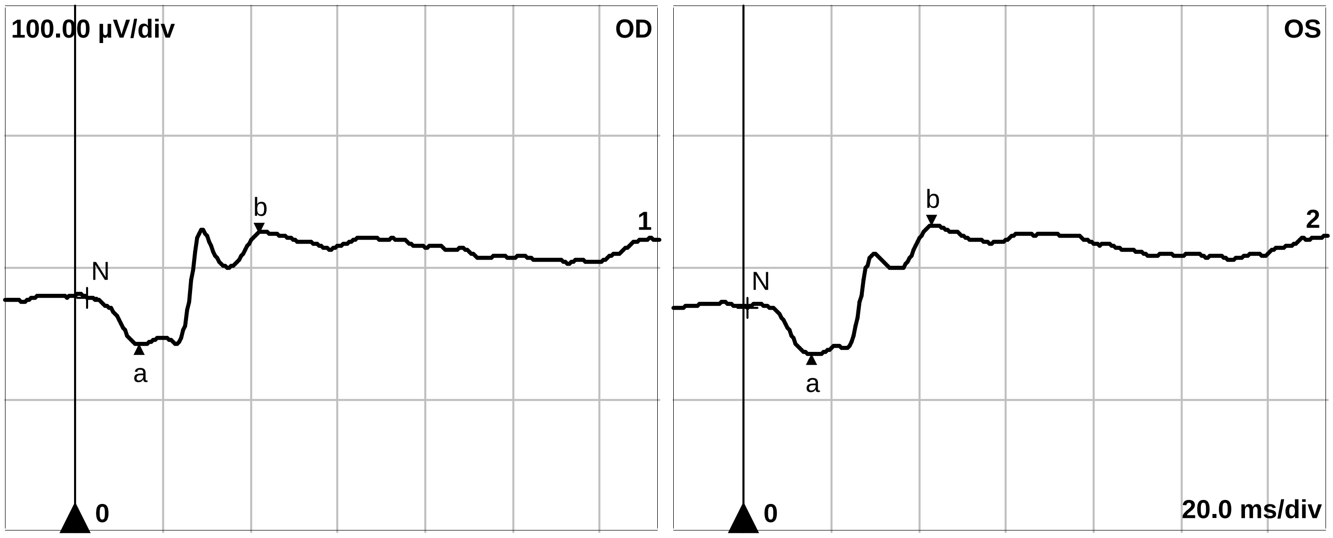

Step	a [ms]	b [ms]	a-wave [µV]	b-wave [µV]	b/a	Avgs.
Normal	15.3-19.4	32.9-58.7	118.0-315.0	168.0-527.0	1.5-2.6	
1: OD-1	14.9 (!)	42.5	36.2 (!)	87.3 (!)	2.4	5
2: OS-2	15.5	43.4	35.6 (!)	99.0 (!)	2.8 (!)	5

Patient:	M-N-2**, DTQ, 5/7/2021**			Electrode:	DTL Thread
Examined:	12/6/2023 10:17	Sex/Age:	male/2	Dilated:	Yes
ID:		Operator:		Pupil Size:	-

3. Dark-adapted 3.0 OPs Stimulator: Ganzfeld

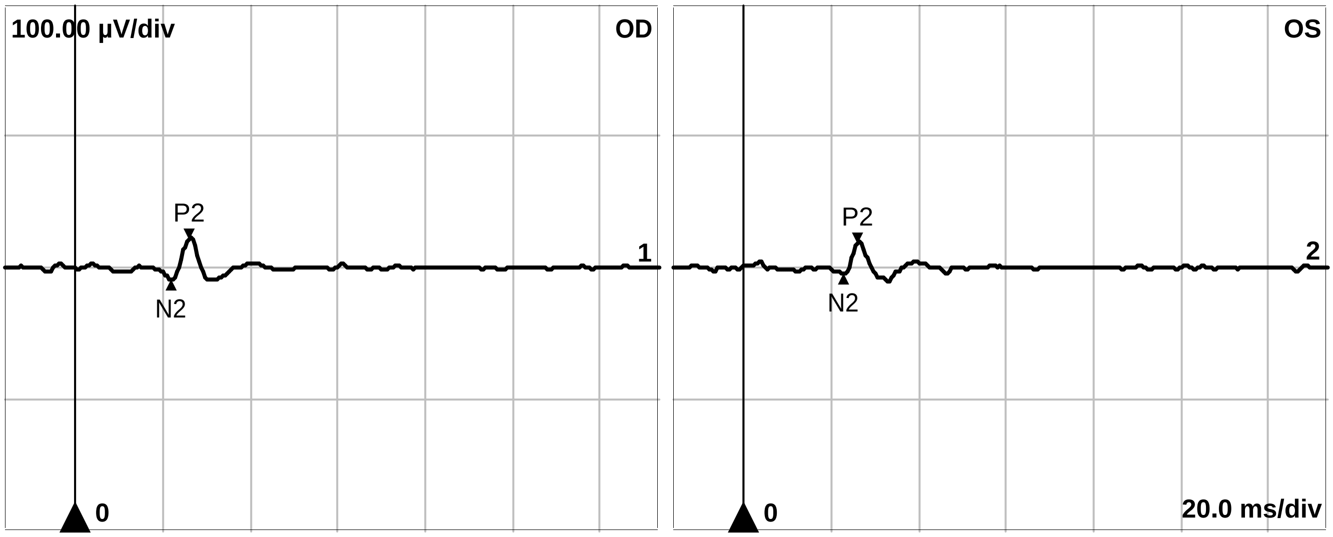

Step	P2 [ms]	OS2 [µV]	Avgs.
Normal	22.9-25.8	14.7-71.1	
1: OD-1	26.4 (!)	33.4	3
2: OS-2	26.4 (!)	25.9	3

4. Dark-adapted 10.0 ERG Stimulator: Ganzfeld

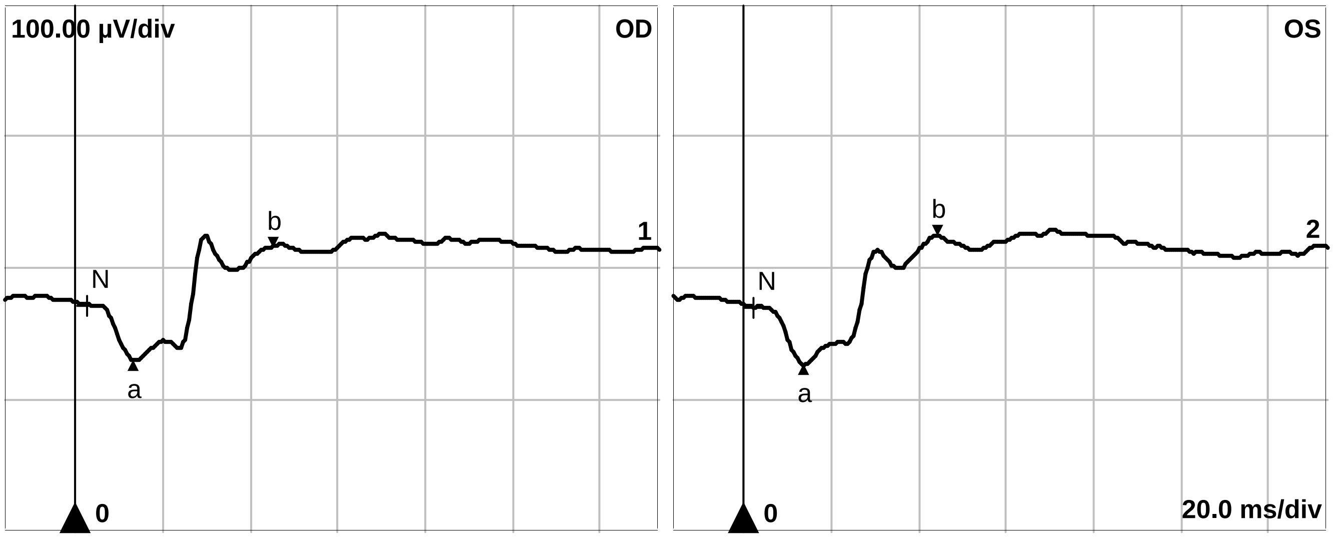

Step	a [ms]	b [ms]	a-wave [µV]	b-wave [µV]	b/a [µV]	Avgs.
Normal	12.9-17.0	32.6-50.8	135.0-322.0	180.0-550.0		
1: OD-1	13.5	45.4	42.9 (!)	87.9 (!)	2046478.7	5
2: OS-2	13.8	44.5	44.1 (!)	100.0 (!)	2267970.3	5

RETIport	Enter Clinic ID<br>Enter your address!					
	Patient:	M-N-3, **DTQ, 5/7/2021**			Electrode:	DTL Thread
	Examined:	12/5/2023 10:40	Sex/Age:	male/2	Dilated:	Yes
	ID:		Operator:		Pupil Size:	-

1. Dark-adapted 0.01 ERG Stimulator: Ganzfeld

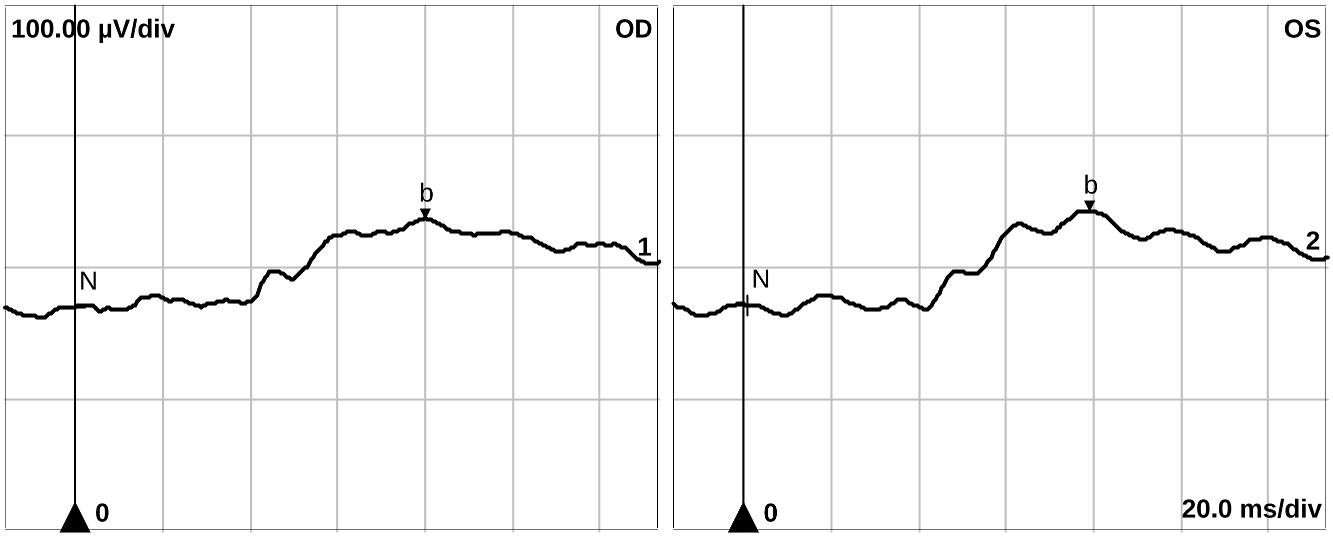

Step	b [ms]	b-wave [µV]	Avgs.
Normal	66.0-93.0	107.0-356.0	
1: OD-1	80.3	69.0 (!)	5
2: OS-2	79.4	73.1 (!)	5

2. Dark-adapted 3.0 ERG Stimulator: Ganzfeld

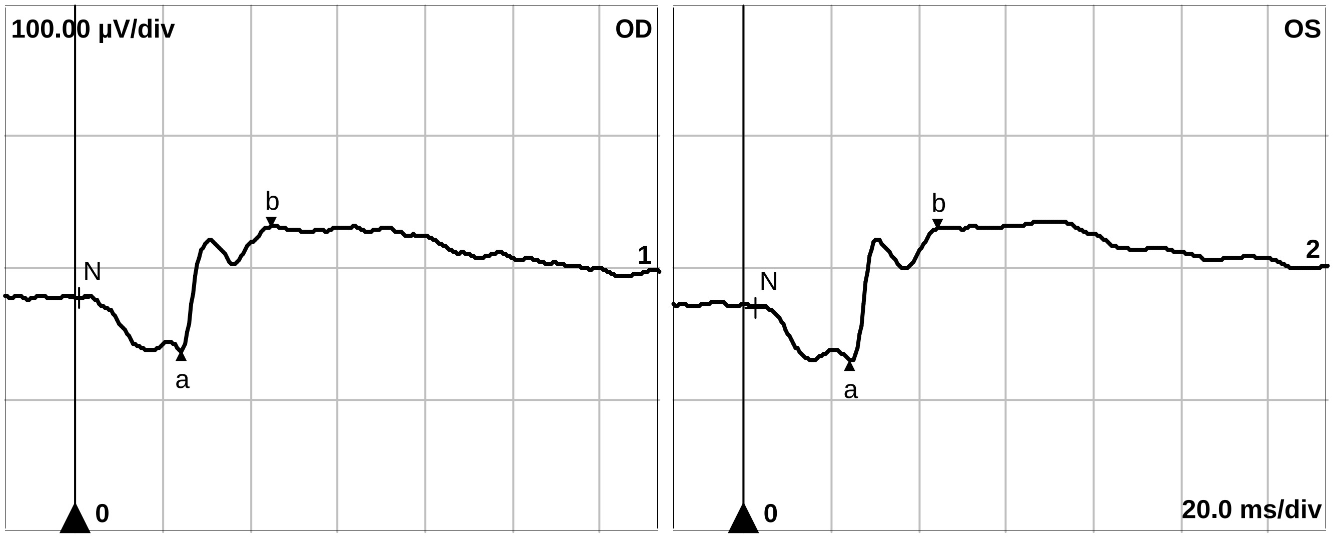

Step	a [ms]	b [ms]	a-wave [µV]	b-wave [µV]	b/a	Avgs.
Normal	15.3-19.4	32.9-58.7	118.0-315.0	168.0-527.0	1.5-2.6	
1: OD-1	24.3 (!)	44.8	41.1 (!)	96.3 (!)	2.3	5
2: OS-2	24.6 (!)	44.5	41.6 (!)	102.0 (!)	2.5	5

3. Dark-adapted 3.0 OPs Stimulator: Ganzfeld

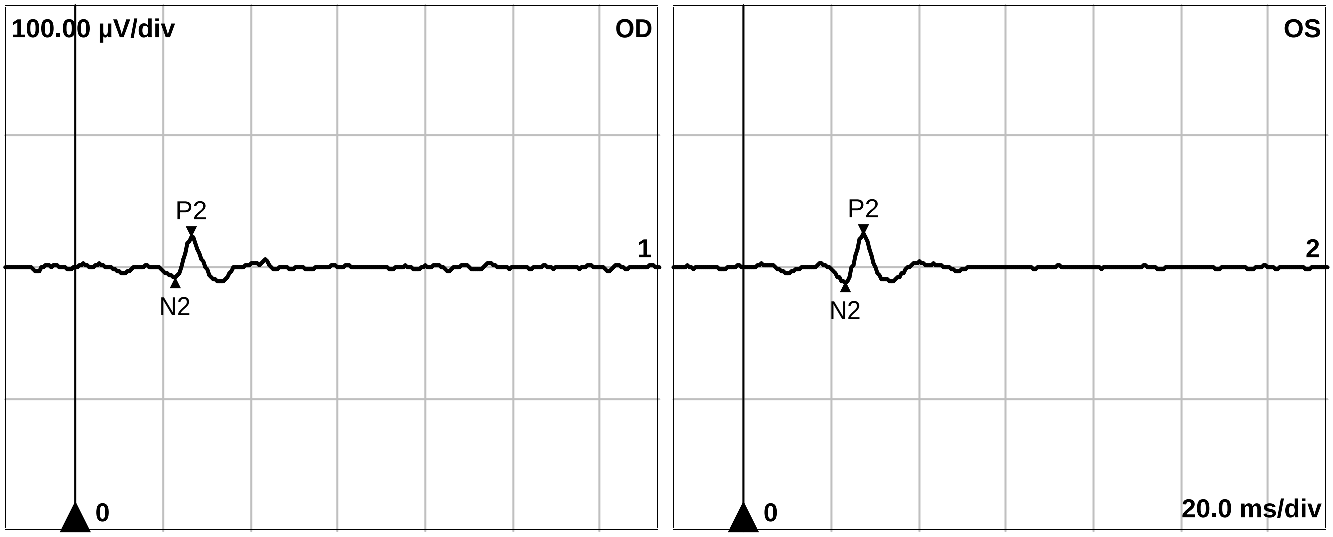

Step	P2 [ms]	OS2 [µV]	Avgs.
Normal	22.9-25.8	14.7-71.1	
1: OD-1	26.7 (!)	32.1	3
2: OS-2	27.5 (!)	39.1	3

4. Dark-adapted 10.0 ERG Stimulator: Ganzfeld

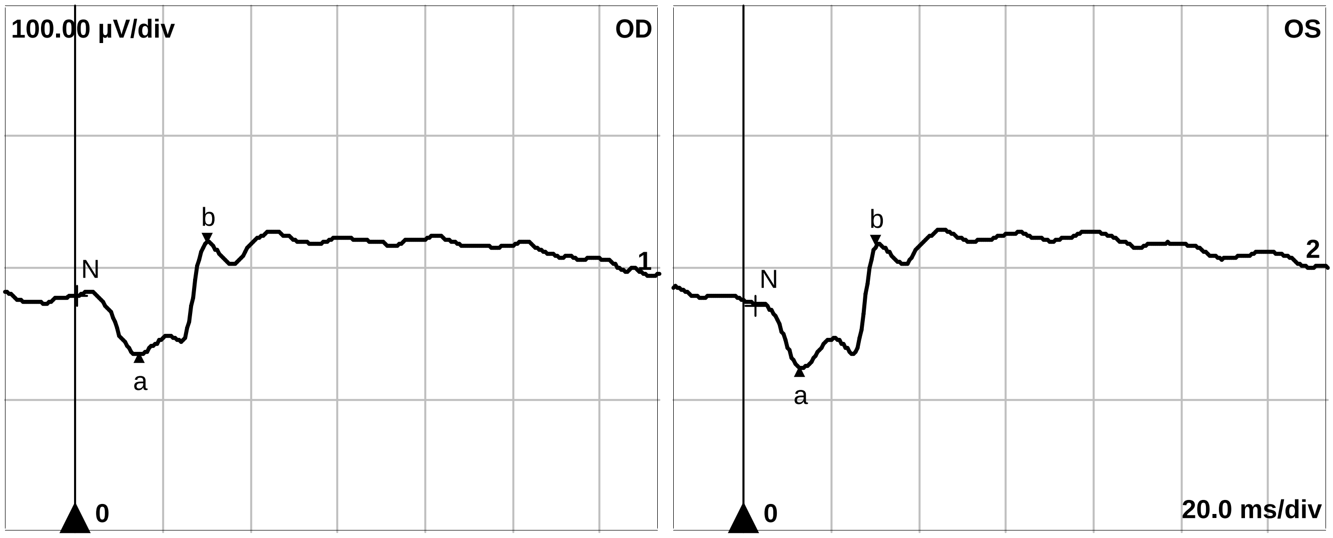

Step	a [ms]	b [ms]	a-wave [µV]	b-wave [µV]	b/a [µV]	Avgs.
Normal	12.9-17.0	32.6-50.8	135.0-322.0	180.0-550.0		
1: OD-1	14.6	30.2 (!)	44.2 (!)	85.7 (!)	1940701.7	5
2: OS-2	13.2	30.5 (!)	48.4 (!)	94.5 (!)	1952789.8	5

RETIport	Enter Clinic ID<br>Enter your address!

Patient: CON, 2, 4/22/2025
Examined: 4/22/2025 17:43
ID: 2
Sex/Age: male/0 days
Operator:
Electrode: DTL Thread
Dilated: Yes
Pupil Size: -

PhNR Stimulator: Ganzfeld

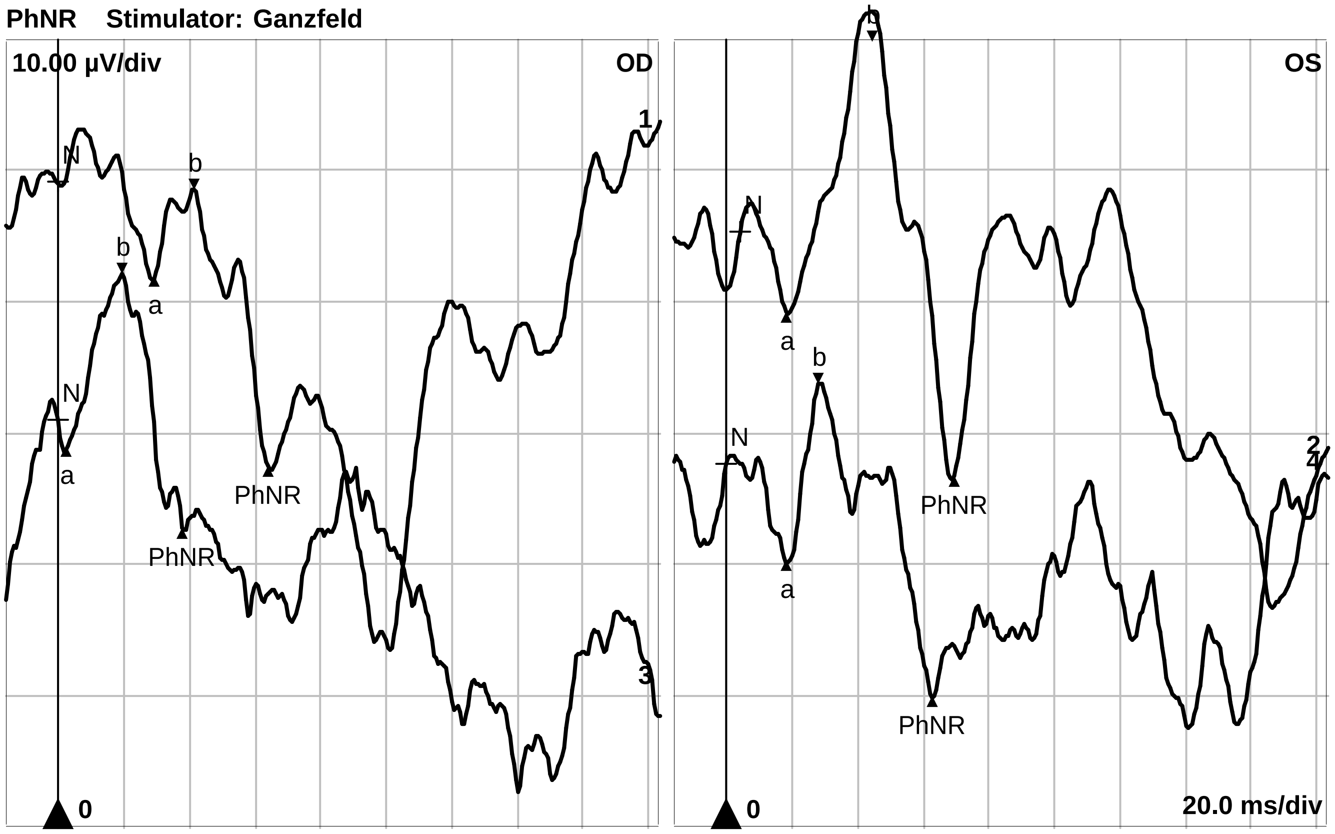

Step	a [ms]	b [ms]	a-wave [µV]	b-wave [µV]	PhNR [µV]	Avgs.
1: OD 1.	29.3	41.4	7.3	6.9	21.8	4
3: OD 2.	2.34	19.9	2.2	13.5	8.4	7
2: OS 1.	18.8	44.5	6.2	23.1	18.7	5
4: OS 2.	18.4	28.5	7.5	13.8	17.9	9

RETIport	Enter Clinic ID<br>Enter your address!					
	Patient:	CON, 4/22/2025			Electrode:	DTL Thread
	Examined:	4/22/2025 18:31	Sex/Age:	male/0 days	Dilated:	Yes
	ID:	2	Operator:		Pupil Size:	-

PhNR Stimulator: Ganzfeld

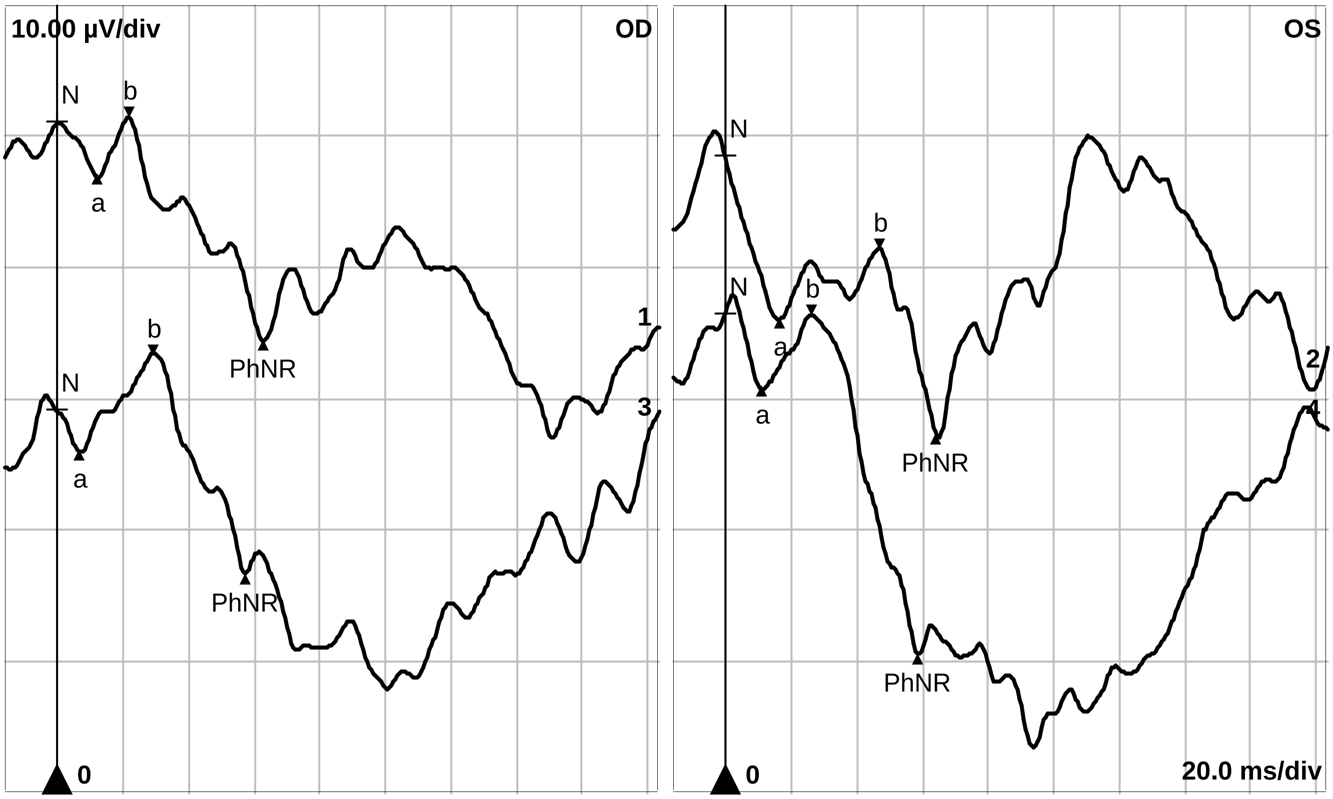

Step	a [ms]	b [ms]	a-wave [µV]	b-wave [µV]	PhNR [µV]	Avgs.
1: OD 1.	12.5	22.3	4.1	4.6	16.7	13
3: OD 2.	7.03	29.7	3.1	7.5	12.6	9
2: OS 1.	16.4	47.3	12.4	5.5	21.3	9
4: OS 2.	11.3	26.2	5.8	5.6	26.1	9

RETIport	Enter Clinic ID<br>Enter your address!

Patient:	CON, **4/22/2025**			Electrode:	DTL Thread
Examined:	4/22/2025 18:51	Sex/Age:	male/0 days	Dilated:	Yes
ID:	2	Operator:		Pupil Size:	-

PhNR Stimulator: Ganzfeld

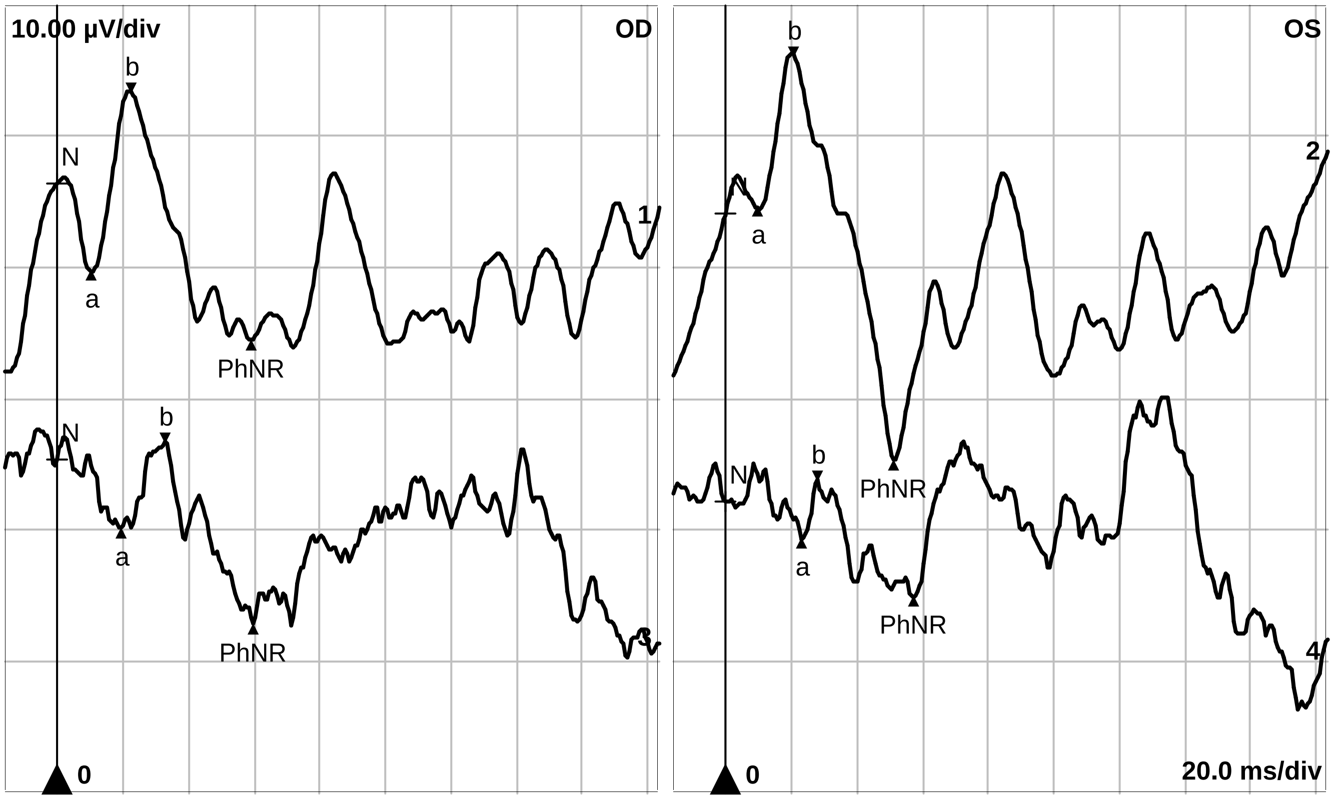

Step	a [ms]	b [ms]	a-wave [µV]	b-wave [µV]	PhNR [µV]	Avgs.
1: OD 1.	10.5	23.0	6.8	13.7	12.0	3
3: OD 2.	19.9	33.2	5.4	6.7	12.6	4
2: OS 1.	10.2	21.1	0.4	11.6	18.9	3
4: OS 2.	23.4	28.1	3.0	4.7	7.4	4

RETIport

Enter Clinic ID
Enter your address!

Patient:	CON, 4/22/2025			Electrode:	DTL Thread
Examined:	4/22/2025 18:57	Sex/Age:	male/0 days	Dilated:	Yes
ID:	2	Operator:		Pupil Size:	-

PhNR Stimulator: Ganzfeld

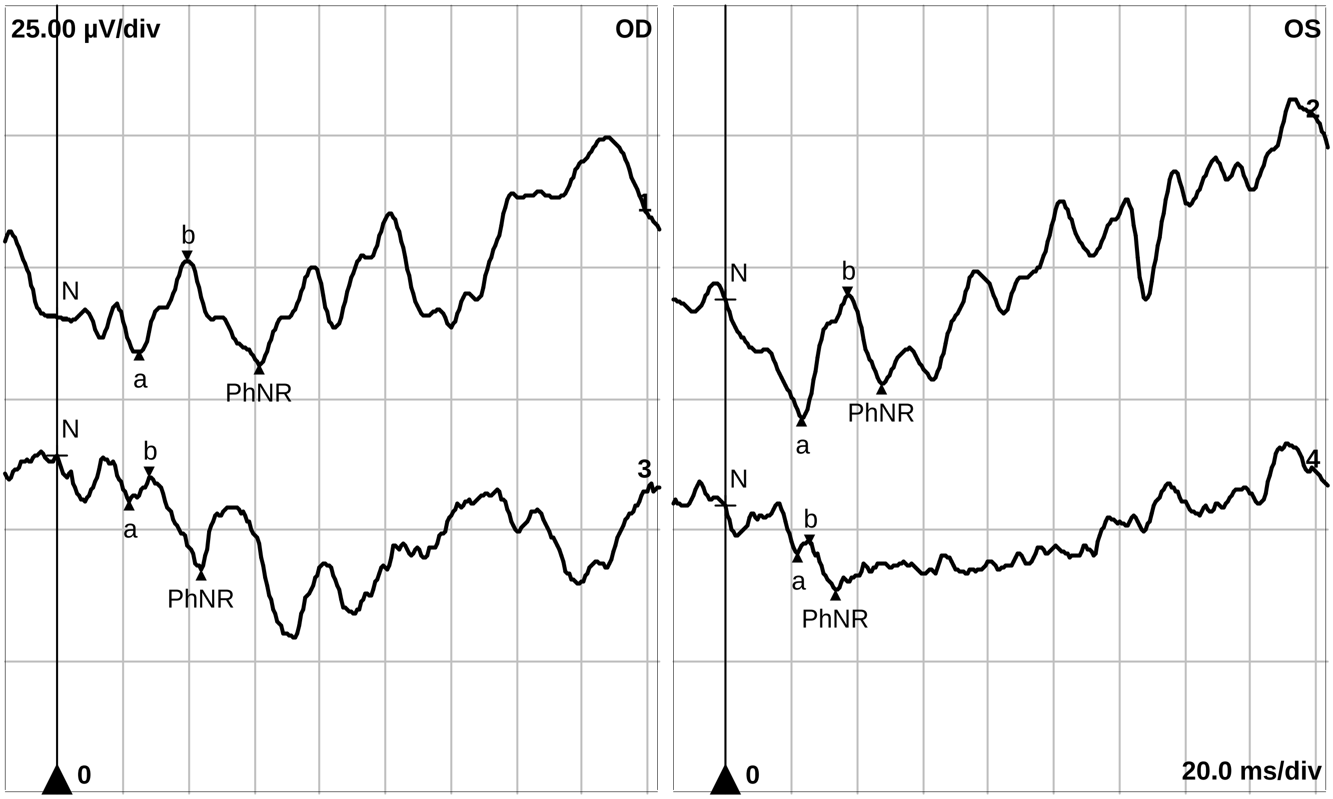

Step	a [ms]	b [ms]	a-wave [µV]	b-wave [µV]	PhNR [µV]	Avgs.
1: OD 1.	25.0	39.8	6.6	17.6	9.2	1
3: OD 2.	22.3	28.5	8.5	4.9	22.0	3
2: OS 1.	23.4	37.5	22.6	23.5	16.2	1
4: OS 2.	21.9	25.8	9.3	2.0	16.5	6

RETIport	Enter Clinic ID<br>Enter your address!

Patient:	CON, **4/22/2025**			Electrode:	DTL Thread
Examined:	4/22/2025 19:00	Sex/Age:	male/0 days	Dilated:	Yes
ID:	2	Operator:		Pupil Size:	-

PhNR Stimulator: Ganzfeld

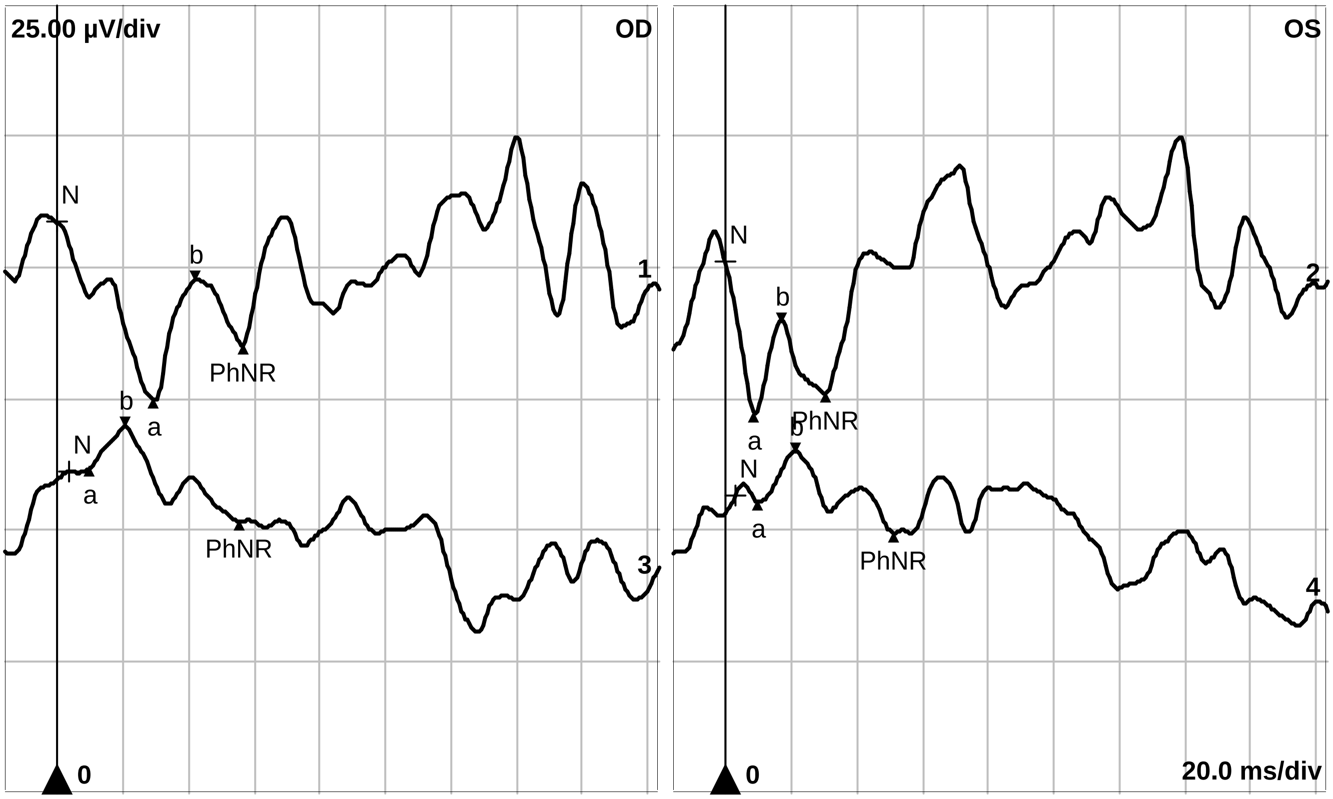

Step	a [ms]	b [ms]	a-wave [µV]	b-wave [µV]	PhNR [µV]	Avgs.
1: OD 1.	29.3	42.6	34.1	23.0	23.7	1
3: OD 2.	10.2	20.7	0.6	8.0	9.6	3
2: OS 1.	8.98	17.2	29.0	17.8	25.1	1
4: OS 2.	10.2	21.5	1.1	9.4	7.5	3

RETIport	Enter Clinic ID<br>Enter your address!					
	Patient:	CON, **4/22/2025**			Electrode:	DTL Thread
	Examined:	4/22/2025 19:09	Sex/Age:	male/0 days	Dilated:	Yes
	ID:	2	Operator:		Pupil Size:	-

PhNR Stimulator: Ganzfeld

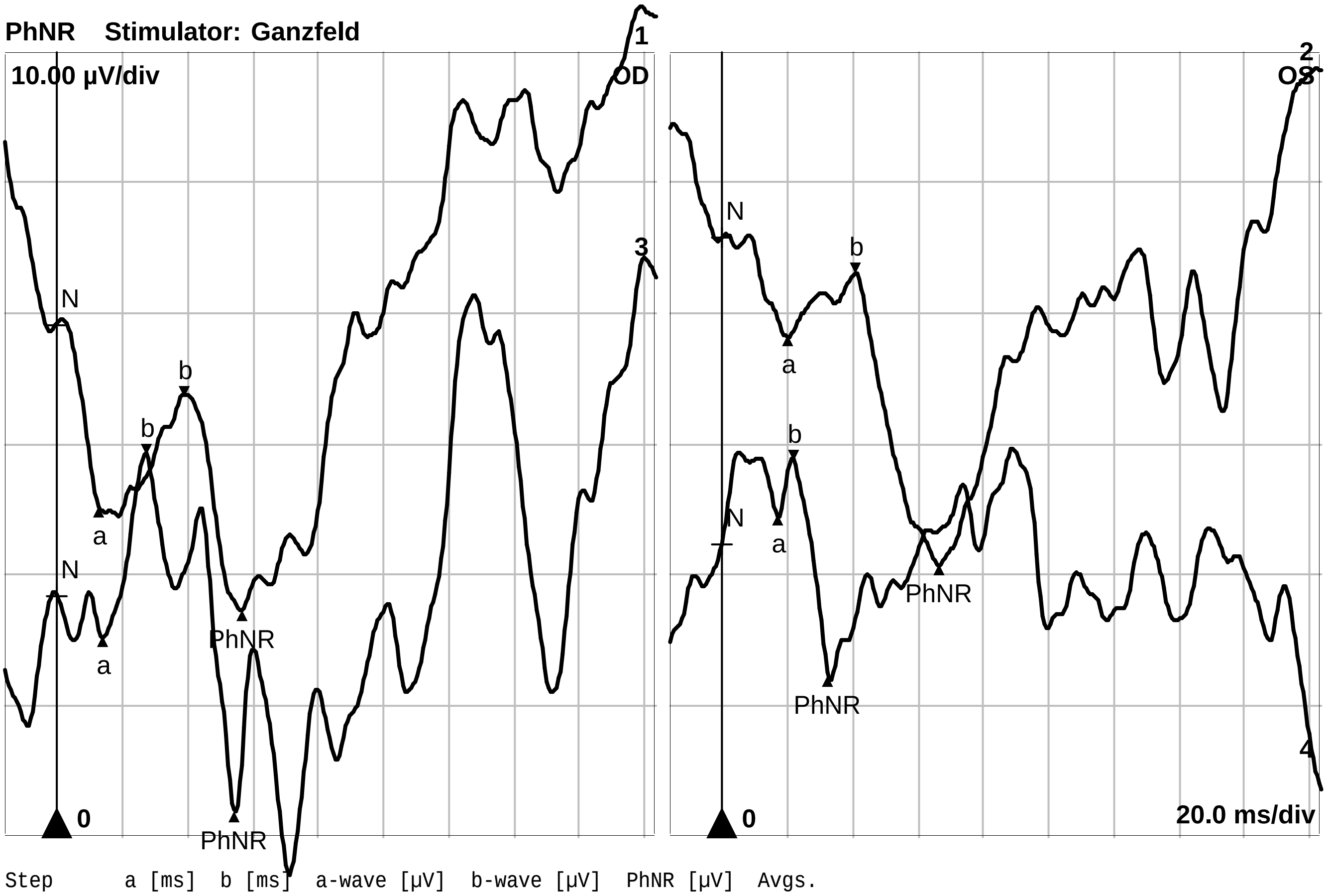

Step	a [ms]	b [ms]	a-wave [µV]	b-wave [µV]	PhNR [µV]	Avgs.
1: OD 1.	13.3	39.5	14.2	8.7	22.0	2
3: OD 2.	14.5	27.3	3.3	14.2	16.7	1
2: OS 1.	20.3	41.0	7.7	5.0	25.1	2
4: OS 2.	17.2	21.9	2.2	4.3	10.2	2

RETIport	Enter Clinic ID<br>Enter your address!

Patient:	GLU, 5/7/2021			Electrode:	DTL Thread
Examined:	3/26/2025 10:30	Sex/Age:	male/3	Dilated:	Yes
ID:	2	Operator:		Pupil Size:	-

PhNR Stimulator: Ganzfeld

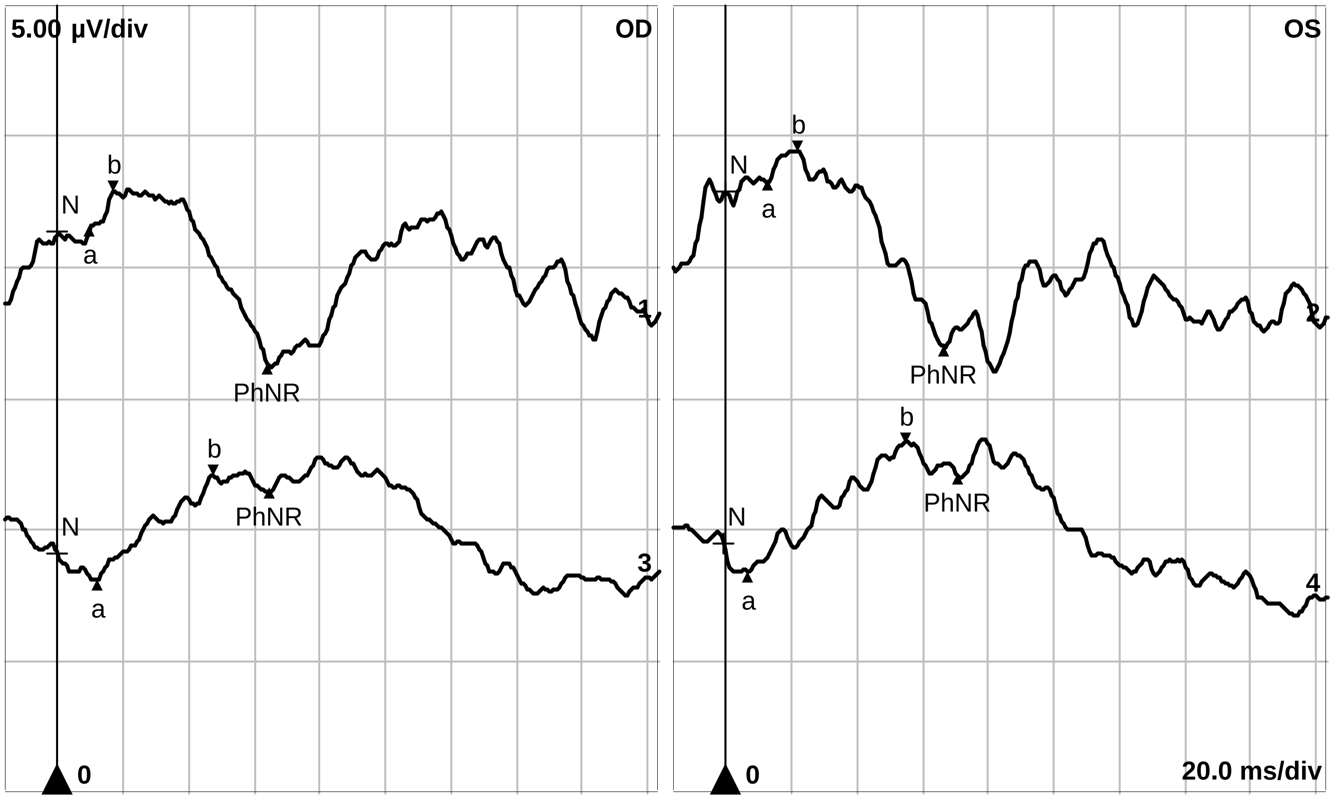

Step	a [ms]	b [ms]	a-wave [µV]	b-wave [µV]	PhNR [µV]	Avgs.
1: OD 1.	10.2	17.2	0.2	1.4	5.1	60
3: OD 2.	12.1	47.7	1.1	4.1	2.4	200
2: OS 1.	12.9	21.9	0.4	1.2	5.9	60
4: OS 2.	6.64	55.1	1.1	5.0	2.6	200

RETIport	Enter Clinic ID<br>Enter your address!					
	Patient:	1, 11, 5/7/2021			Electrode:	DTL Thread
	Examined:	3/26/2025 10:57	Sex/Age:	male/3	Dilated:	Yes
	ID:	3	Operator:		Pupil Size:	-

PhNR Stimulator: Ganzfeld

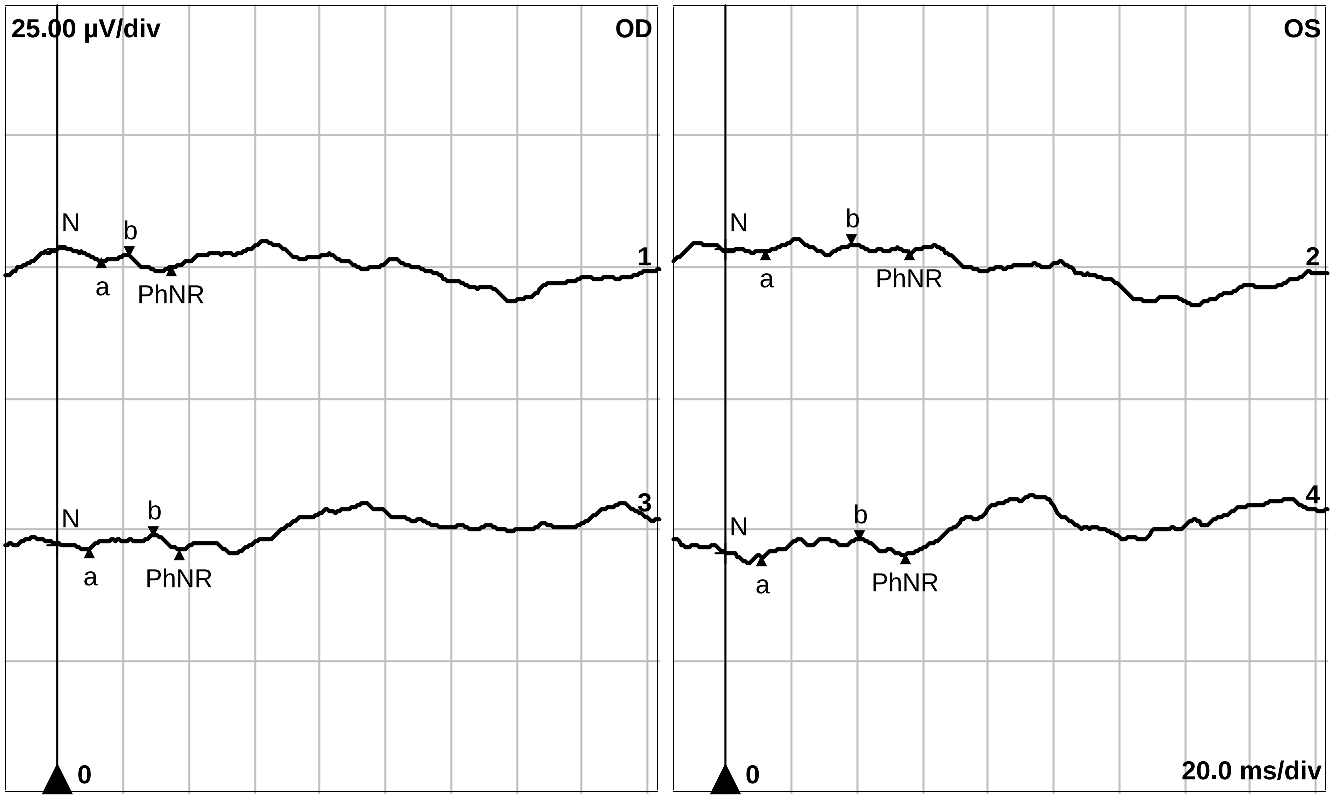

Step	a [ms]	b [ms]	a-wave [µV]	b-wave [µV]	PhNR [µV]	Avgs.
1: OD 1.	13.7	21.9	2.1	1.0	3.5	80
3: OD 2.	10.2	29.3	0.9	2.6	1.2	60
2: OS 1.	12.5	38.7	0.3	1.4	0.5	80
4: OS 2.	11.3	41.0	0.7	3.4	0.3	60

RETIport	Enter Clinic ID<br>Enter your address!					
	Patient:	GLU, 5/7/2021			Electrode:	DTL Thread
	Examined:	3/26/2025 11:10	Sex/Age:	male/3	Dilated:	Yes
	ID:	3	Operator:		Pupil Size:	-

PhNR Stimulator: Ganzfeld

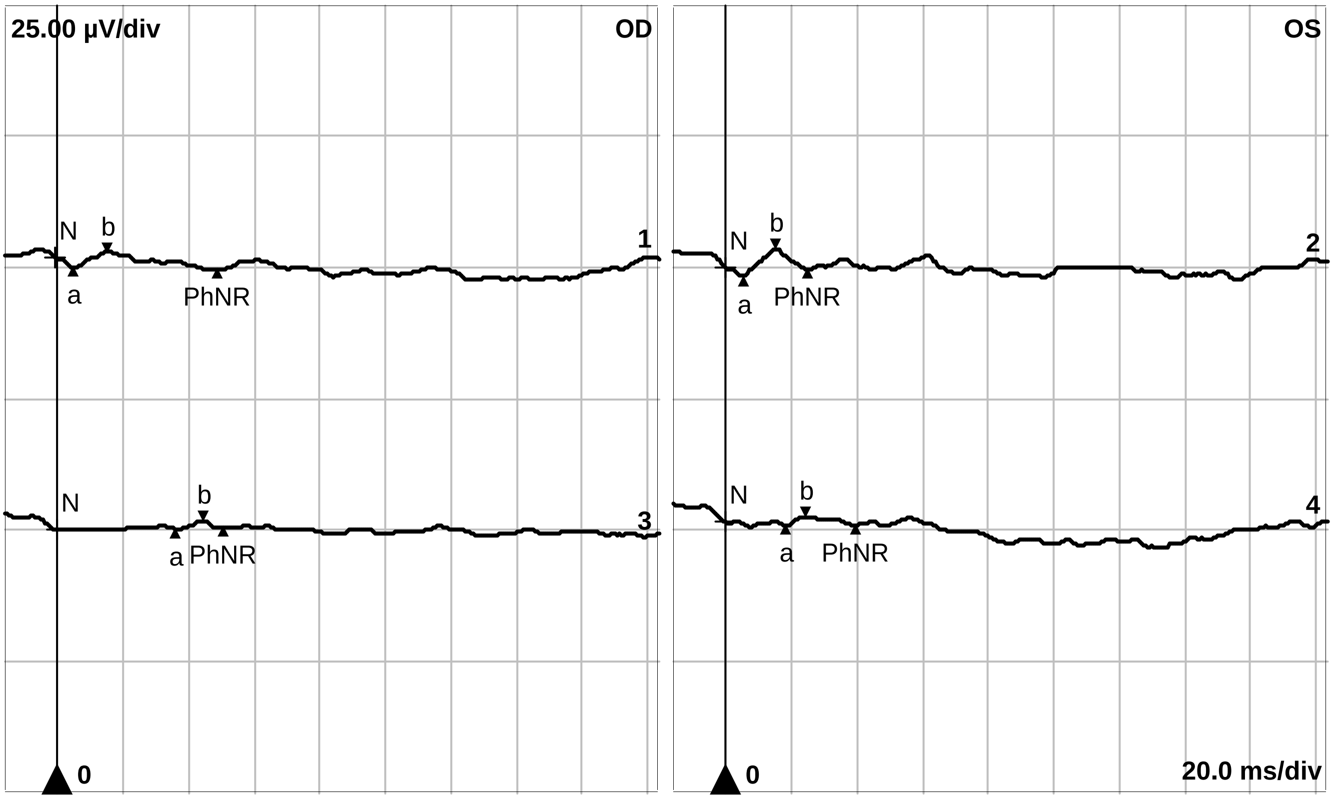

Step	a [ms]	b [ms]	a-wave [µV]	b-wave [µV]	PhNR [µV]	Avgs.
1: OD 1.	5.08	15.2	1.9	3.0	2.6	80
3: OD 2.	36.3	44.5	0.2	1.7	0.5	140
2: OS 1.	5.86	15.6	2.0	5.4	0.6	80
4: OS 2.	18.8	24.6	0.7	1.6	0.8	140

RETIport	Enter Clinic ID<br>Enter your address!			
Patient:	d, dd, 5/7/2021		Electrode:	DTL Thread
Examined:	3/26/2025 12:17	Sex/Age: male/3	Dilated:	Yes
ID:	4	Operator:	Pupil Size:	-

PhNR Stimulator: Ganzfeld

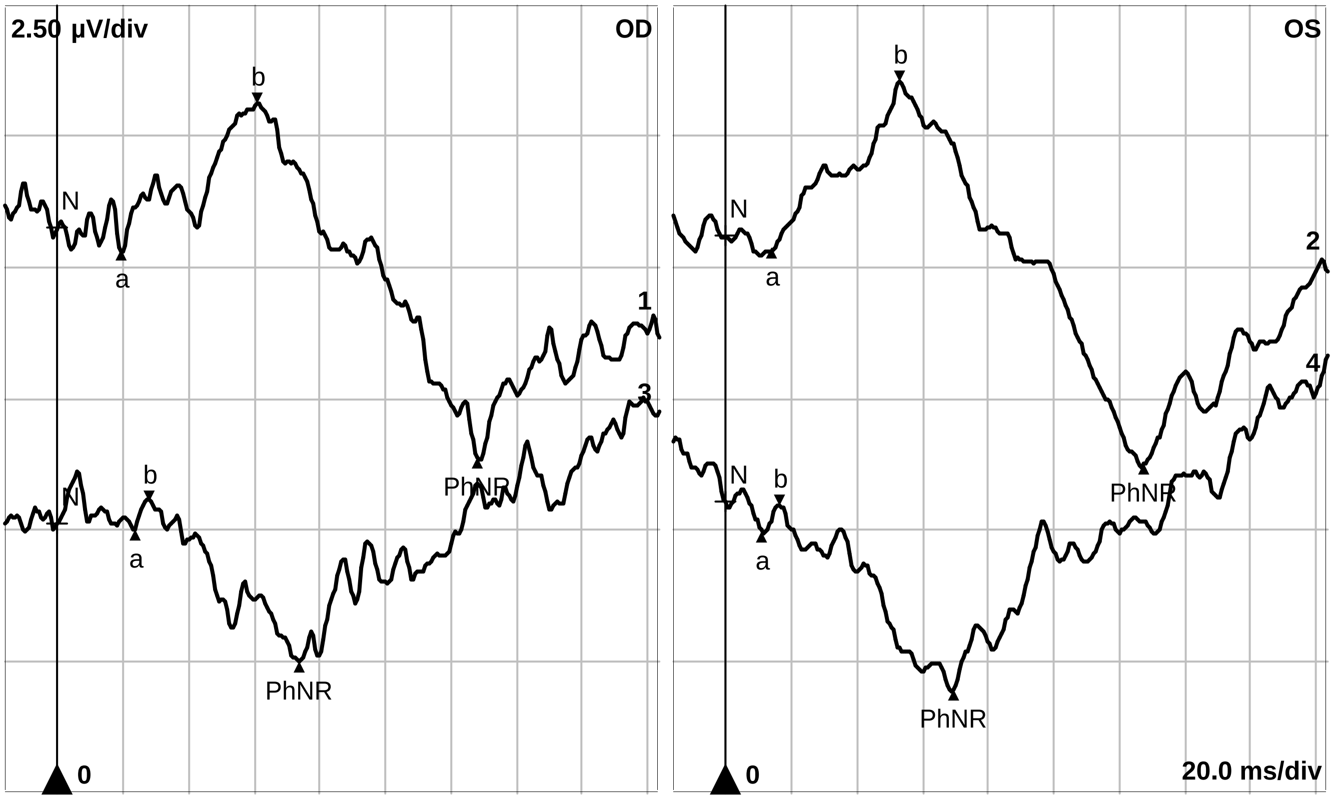

Step	a [ms]	b [ms]	a-wave [µV]	b-wave [µV]	PhNR [µV]	Avgs.
1: OD 1.	19.9	61.3	0.4	2.9	4.4	240
3: OD 2.	23.8	28.1	0.2	0.6	2.7	240
2: OS 1.	14.1	53.1	0.3	3.2	4.4	240
4: OS 2.	11.3	16.4	0.6	0.6	3.6	240

RETIport	Enter Clinic ID<br>Enter your address!

Patient:	GLU, 5/7/2021			Electrode:	DTL Thread
Examined:	3/26/2025 11:08	Sex/Age:	male/3	Dilated:	Yes
ID:	3	Operator:		Pupil Size:	-

PhNR Stimulator: Ganzfeld

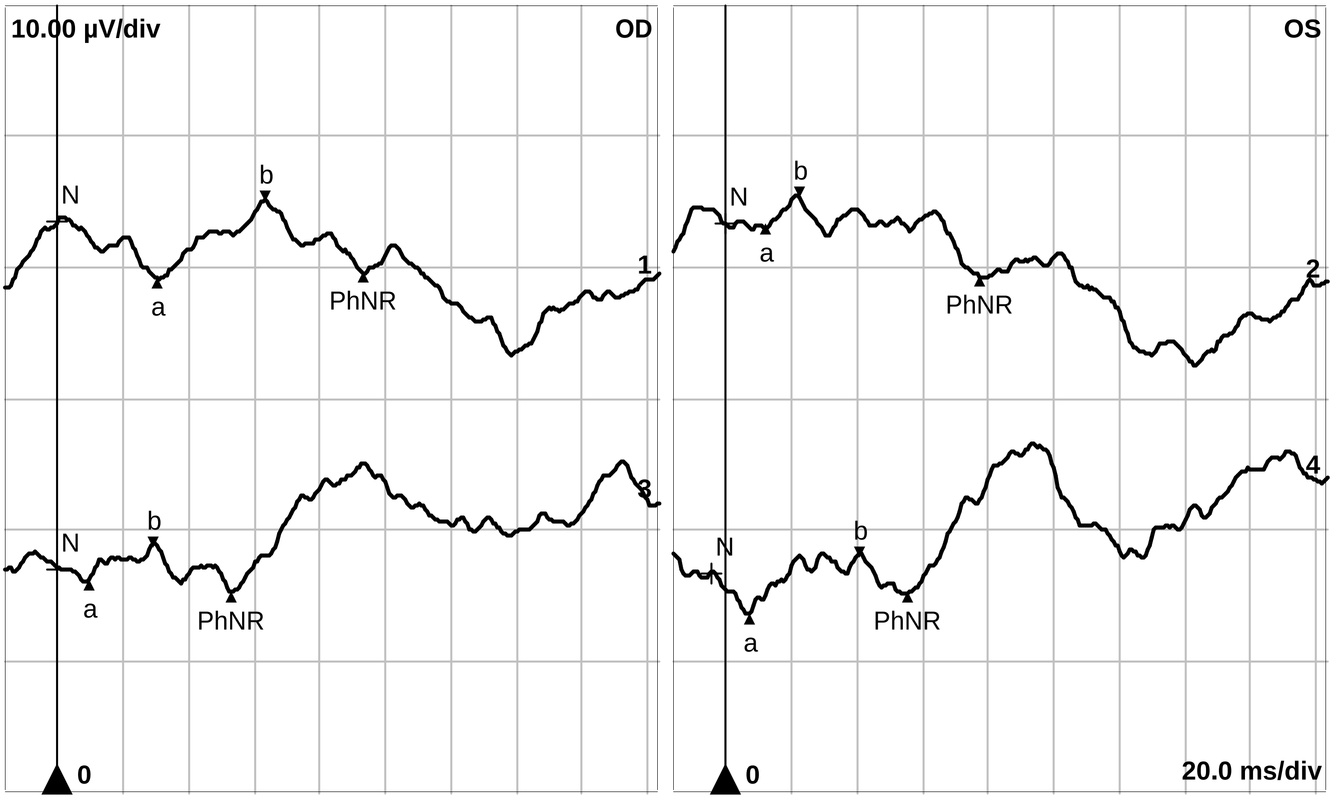

Step	a [ms]	b [ms]	a-wave [µV]	b-wave [µV]	PhNR [µV]	Avgs.
1: OD 1.	30.9	63.7	4.4	6.1	4.0	80
3: OD 2.	10.2	29.3	0.9	2.6	1.8	60
2: OS 1.	12.5	22.7	0.3	2.4	4.1	80
4: OS 2.	7.42	41.0	3.2	4.6	1.6	60

RETIport	Enter Clinic ID<br>Enter your address!

Patient:	**D, TQ, 5/7/2021**			Electrode:	DTL Thread
Examined:	3/26/2025 10:30	Sex/Age:	male/3	Dilated:	Yes
ID:	2	Operator:		Pupil Size:	-

PhNR Stimulator: Ganzfeld

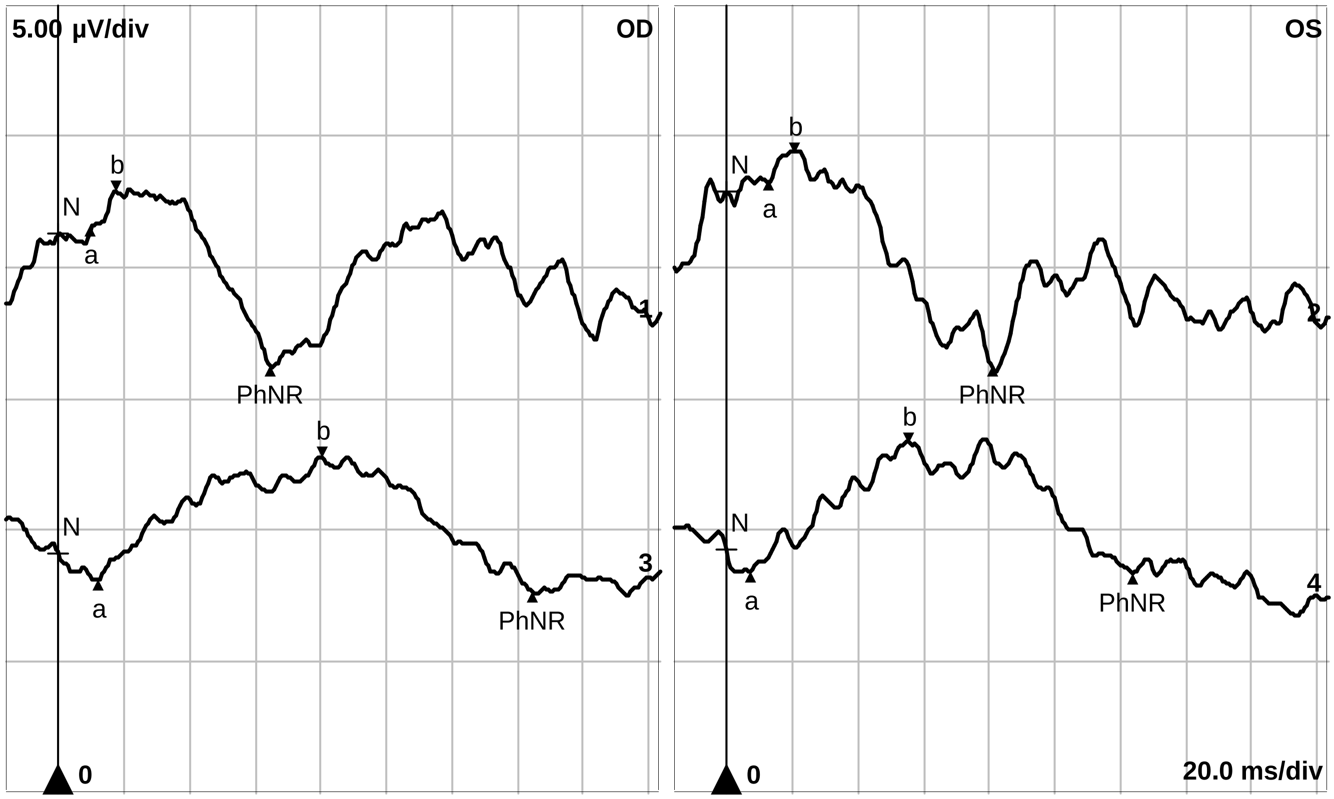

Step	a [ms]	b [ms]	a-wave [µV]	b-wave [µV]	PhNR [µV]	Avgs.
1: OD 1.	10.2	17.6	0.3	1.4	5.0	60
3: OD 2.	12.1	80.5	1.1	4.8	1.5	200
2: OS 1.	12.9	21.1	0.4	1.2	6.7	60
4: OS 2.	7.42	55.9	0.9	5.0	0.9	200

RETIport	Enter Clinic ID<br>Enter your address!				
	Patient:	**2, -1, 4/22/2025**		Electrode:	DTL Thread
	Examined:	4/22/2025 16:34	Sex/Age: male/0 days	Dilated:	Yes
	ID:	2	Operator:	Pupil Size:	-

PhNR Stimulator: Ganzfeld

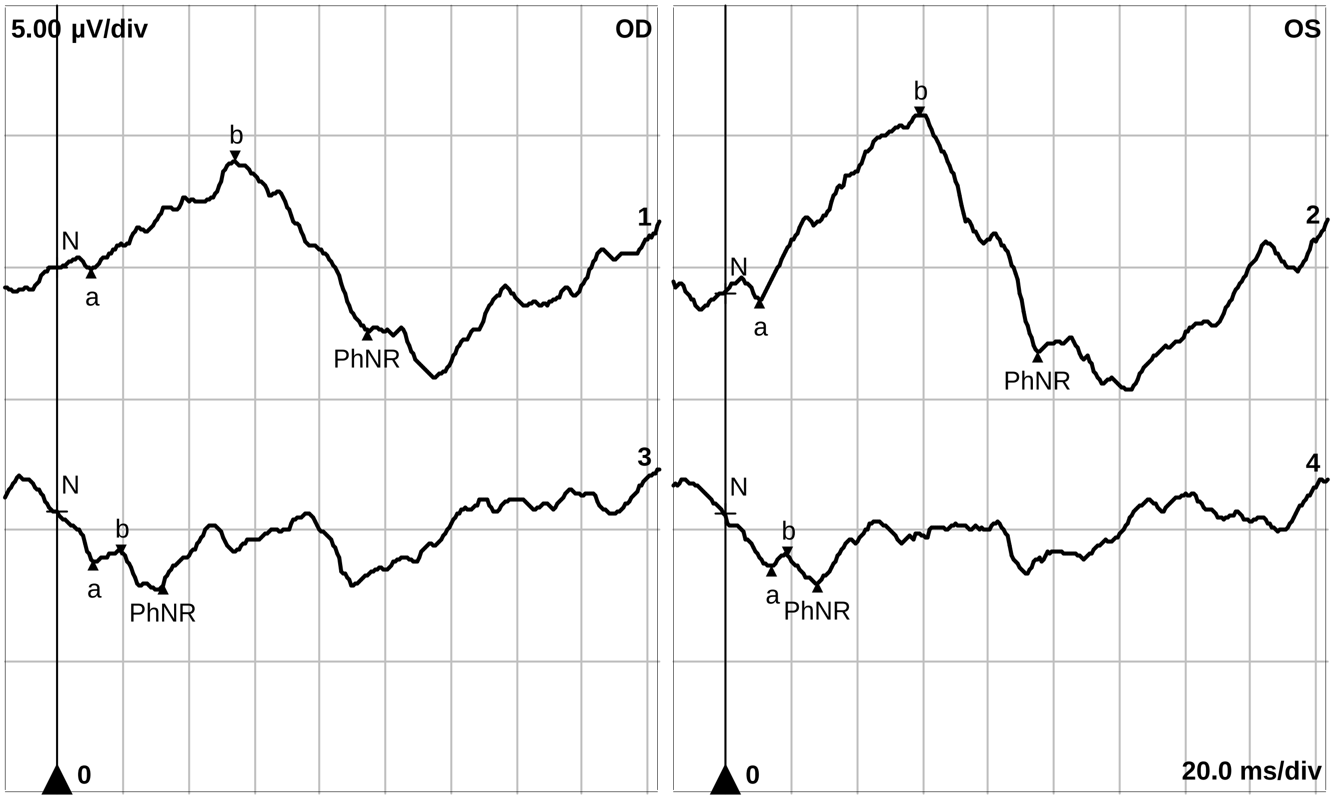

Step	a [ms]	b [ms]	a-wave [µV]	b-wave [µV]	PhNR [µV]	Avgs.
1: OD 1.	10.5	54.3	0.1	4.2	2.4	100
3: OD 2.	10.9	19.9	1.9	0.3	2.8	160
2: OS 1.	10.5	59.4	0.3	7.0	2.3	100
4: OS 2.	14.1	19.1	2.0	0.4	2.6	160

RETIport	Enter Clinic ID<br>Enter your address!					
	Patient:	I2, 4/22/2025			Electrode:	DTL Thread
	Examined:	4/22/2025 17:08	Sex/Age:	male/0 days	Dilated:	Yes
	ID:	2	Operator:		Pupil Size:	-

PhNR Stimulator: Ganzfeld

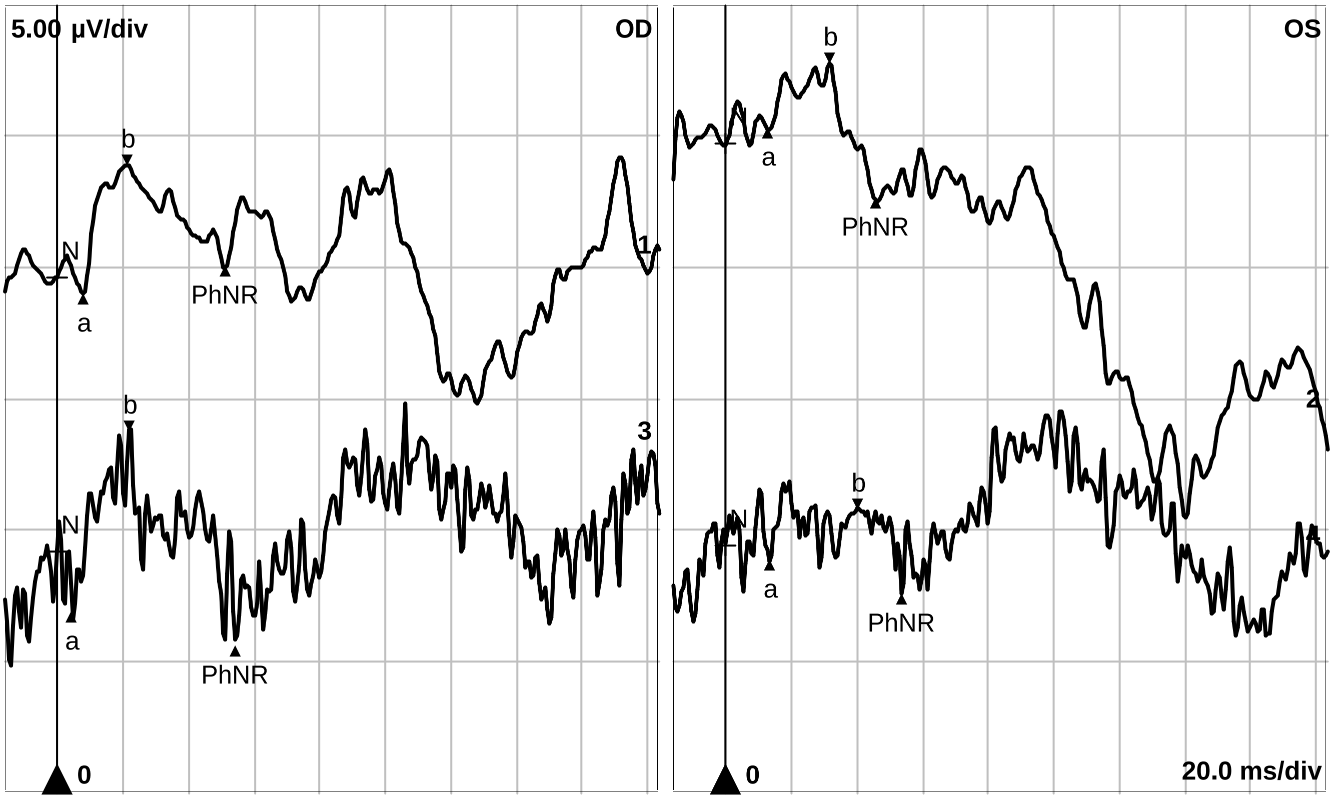

Step	a [ms]	b [ms]	a-wave [µV]	b-wave [µV]	PhNR [µV]	Avgs.
1: OD 1.	8.20	21.5	0.6	5.0	0.4	80
3: OD 2.	4.69	21.9	2.3	7.0	3.7	100
2: OS 1.	13.3	32.0	0.6	2.6	2.1	80
4: OS 2.	13.7	40.6	0.7	2.1	1.9	100

RETIport	Enter Clinic ID<br>Enter your address!				
Patient:	I6, **5/7/2021**			Electrode:	DTL Thread
Examined:	3/26/2025 12:04	Sex/Age:	male/3	Dilated:	Yes
ID:	4	Operator:		Pupil Size:	-

PhNR Stimulator: Ganzfeld

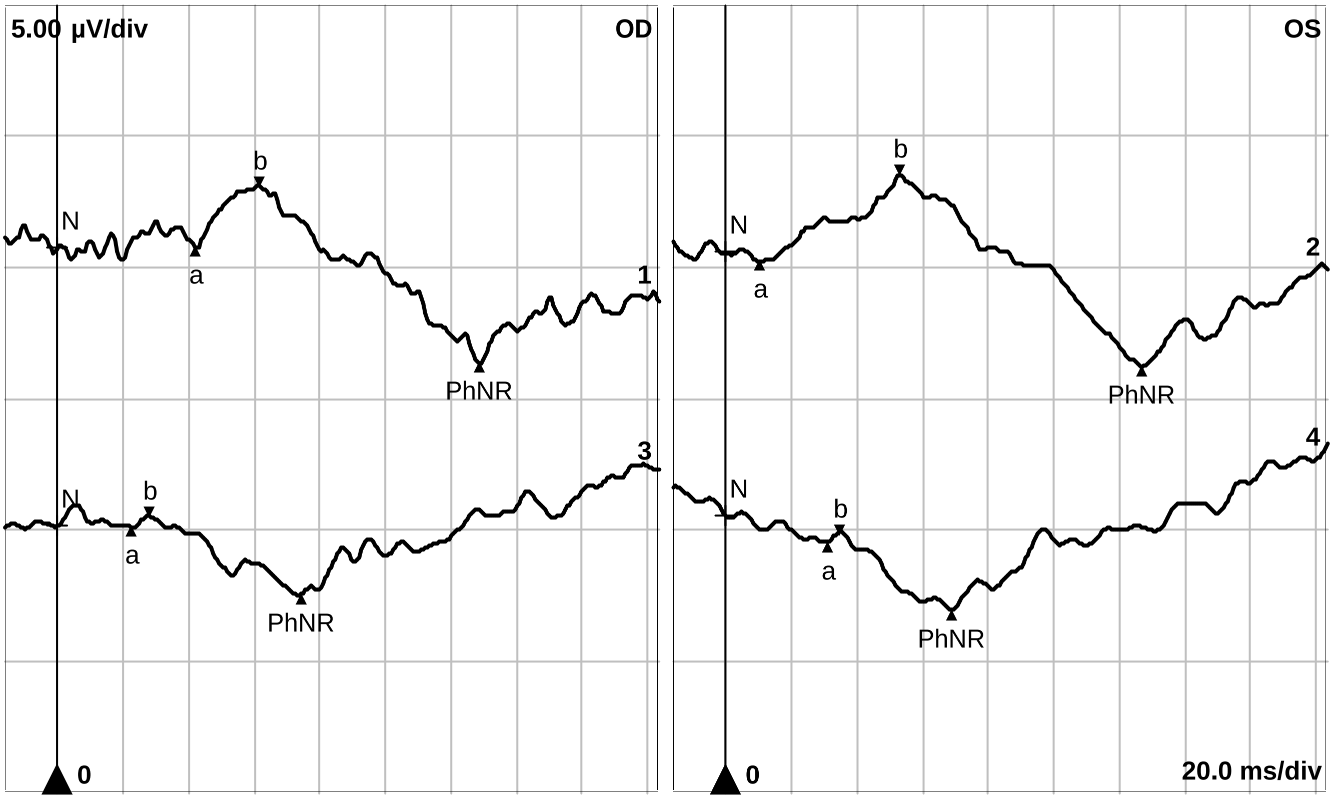

Step	a [ms]	b [ms]	a-wave [µV]	b-wave [µV]	PhNR [µV]	Avgs.
1: OD 1.	42.6	62.1	0.0	2.3	4.4	240
3: OD 2.	23.0	28.1	0.1	0.4	2.7	240
2: OS 1.	10.5	53.5	0.4	3.3	4.4	240
4: OS 2.	31.3	35.2	1.1	0.4	3.6	240

RETIport	Enter Clinic ID<br>Enter your address!					
	Patient:	**d, dd, 5/7/2021**			Electrode:	DTL Thread
	Examined:	3/26/2025 12:39	Sex/Age:	male/3	Dilated:	Yes
	ID:	4	Operator:		Pupil Size:	-

PhNR Stimulator: Ganzfeld

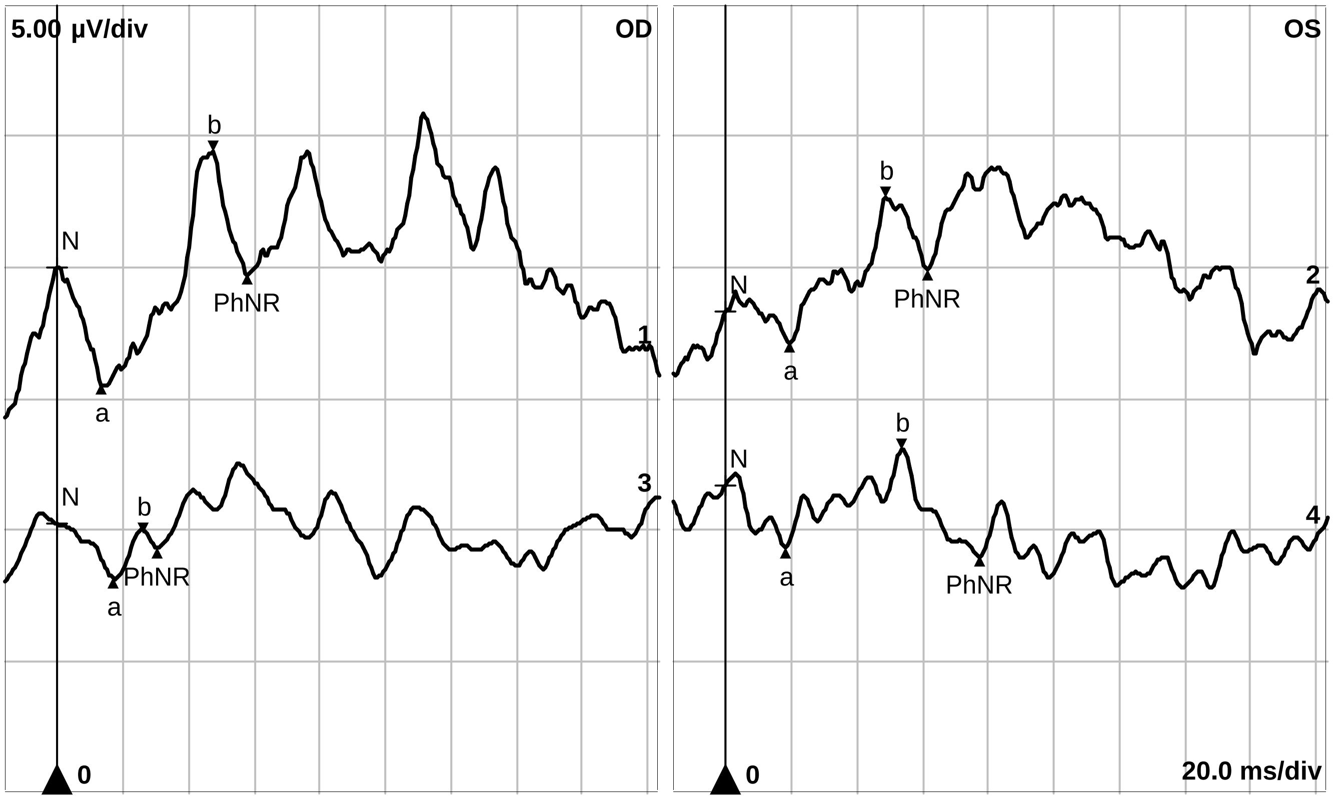

Step	a [ms]	b [ms]	a-wave [µV]	b-wave [µV]	PhNR [µV]	Avgs.
1: OD 1.	13.7	47.7	4.5	8.9	0.3	40
3: OD 2.	17.2	26.2	2.1	1.9	1.0	100
2: OS 1.	19.5	48.8	1.2	5.6	1.5	40
4: OS 2.	18.4	53.9	2.4	3.8	2.8	100

RETIport	Enter Clinic ID<br>Enter your address!

Patient:	I4, 5/7/2021			Electrode:	DTL Thread
Examined:	3/26/2025 10:41	Sex/Age:	male/3	Dilated:	Yes
ID:	2	Operator:		Pupil Size:	-

PhNR Stimulator: Ganzfeld

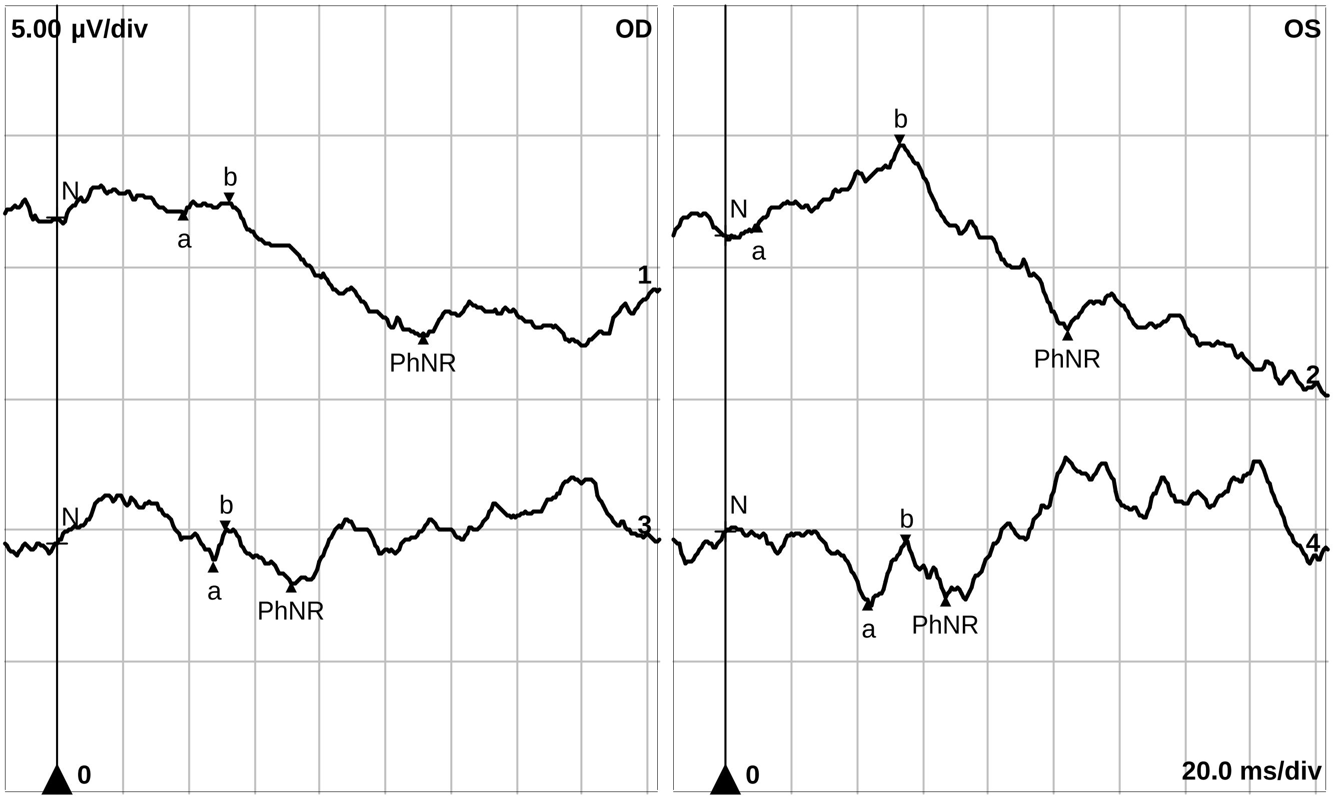

Step	a [ms]	b [ms]	a-wave [µV]	b-wave [µV]	PhNR [µV]	Avgs.
1: OD 1.	38.7	52.7	0.2	0.4	4.5	120
3: OD 2.	48.0	51.6	0.7	1.3	1.5	80
2: OS 1.	10.2	53.5	0.4	3.1	3.6	120
4: OS 2.	43.4	55.1	2.7	2.2	2.5	80

RETIport	Enter Clinic ID<br>Enter your address!					
	Patient:	d, dd, 5/7/2021			Electrode:	DTL Thread
	Examined:	3/26/2025 12:42	Sex/Age:	male/3	Dilated:	Yes
	ID:	4	Operator:		Pupil Size:	-

PhNR Stimulator: Ganzfeld

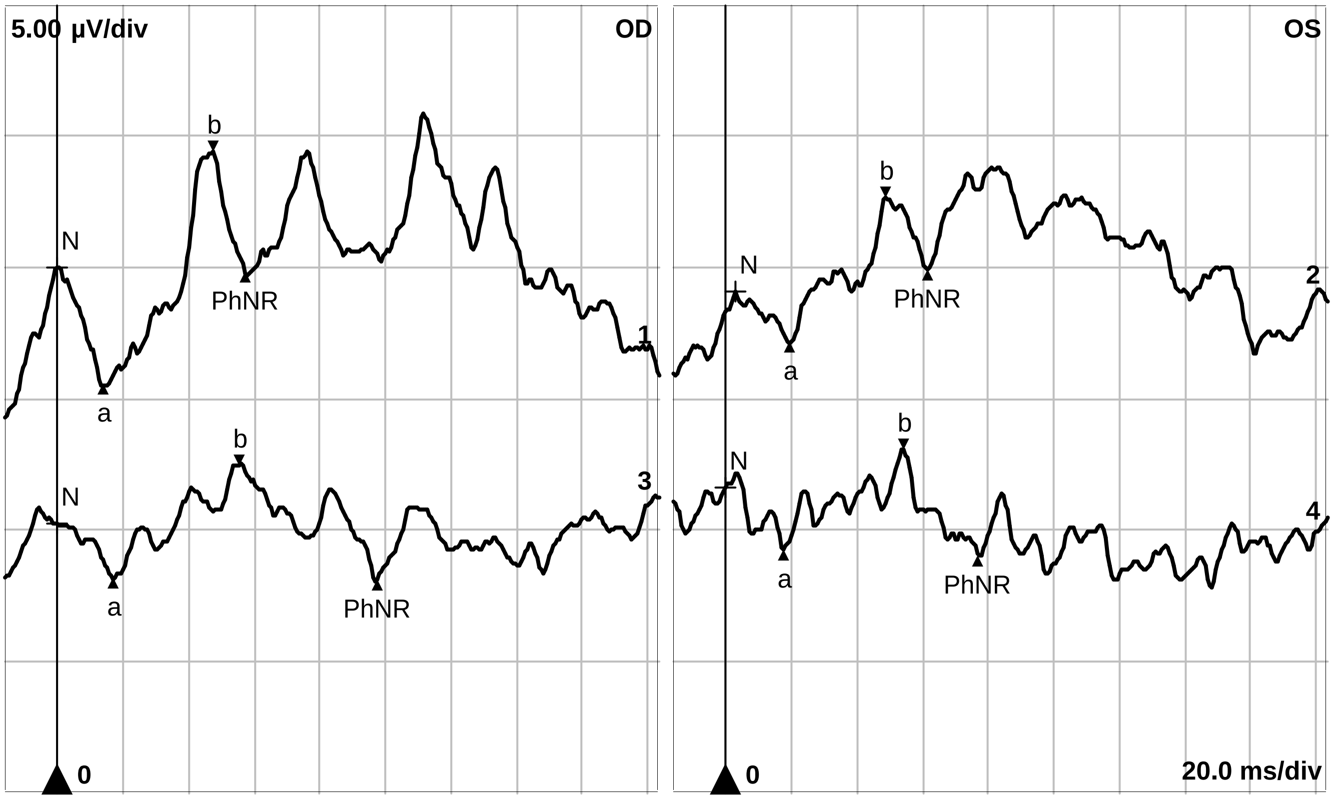

Step	a [ms]	b [ms]	a-wave [µV]	b-wave [µV]	PhNR [µV]	Avgs.
1: OD 1.	14.1	47.7	4.5	9.0	0.2	40
3: OD 2.	17.2	55.5	2.2	4.4	2.2	100
2: OS 1.	19.5	48.8	2.0	5.6	0.8	40
4: OS 2.	17.6	54.3	2.4	3.9	2.7	100

RETIport	Enter Clinic ID<br>Enter your address!					
	Patient:	I-N-4, **5/7/2021**			Electrode:	DTL Thread
	Examined:	3/26/2025 11:15	Sex/Age:	male/3	Dilated:	Yes
	ID:	4	Operator:		Pupil Size:	-

PhNR Stimulator: Ganzfeld

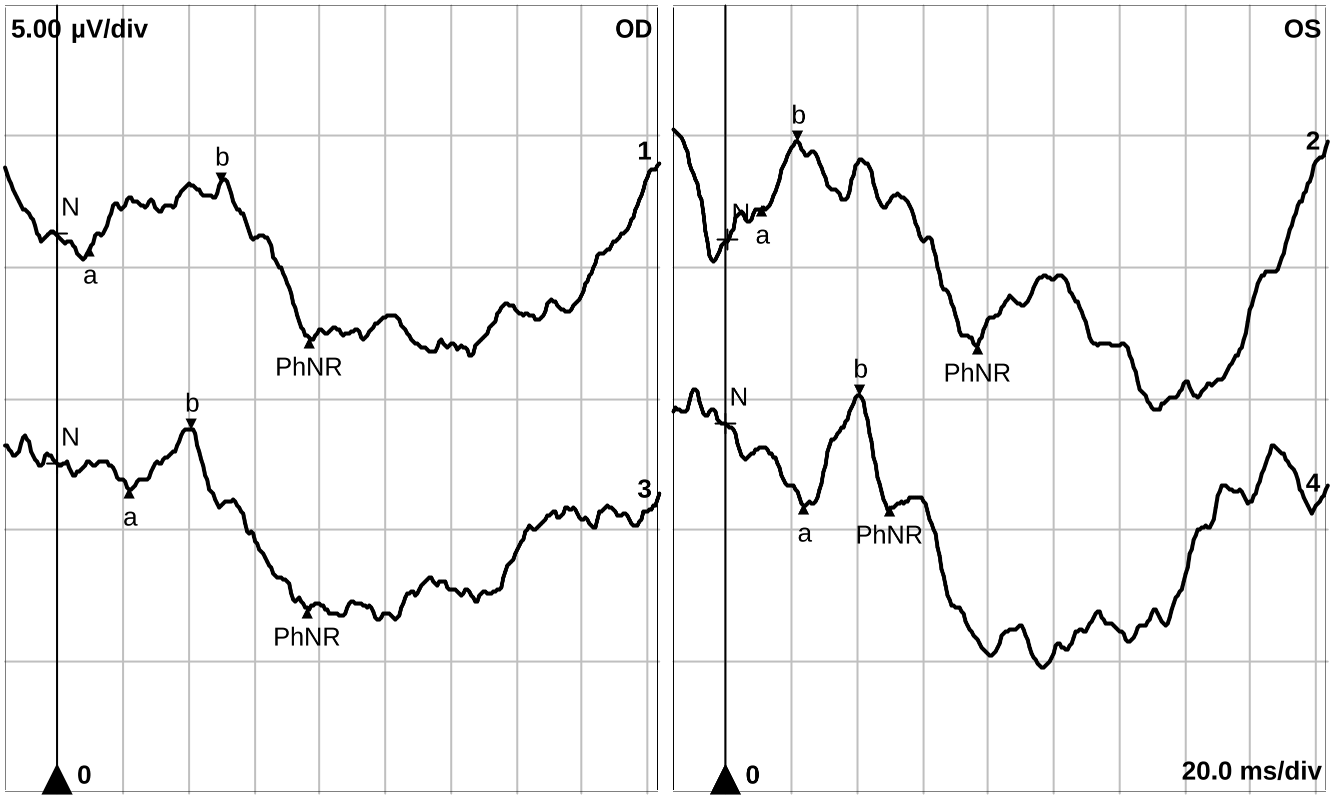

Step	a [ms]	b [ms]	a-wave [µV]	b-wave [µV]	PhNR [µV]	Avgs.
1: OD 1.	10.2	50.0	0.5	2.5	4.0	140
3: OD 2.	22.3	41.0	1.0	2.3	5.6	100
2: OS 1.	10.9	21.9	1.2	2.6	4.1	140
4: OS 2.	23.8	41.0	3.1	4.2	3.3	100

RETIport	Enter Clinic ID<br>Enter your address!					
	Patient:	**1, 11, 5/7/2021**			Electrode:	DTL Thread
	Examined:	3/26/2025 10:52	Sex/Age:	male/3	Dilated:	Yes
	ID:	3	Operator:		Pupil Size:	-

PhNR Stimulator: Ganzfeld

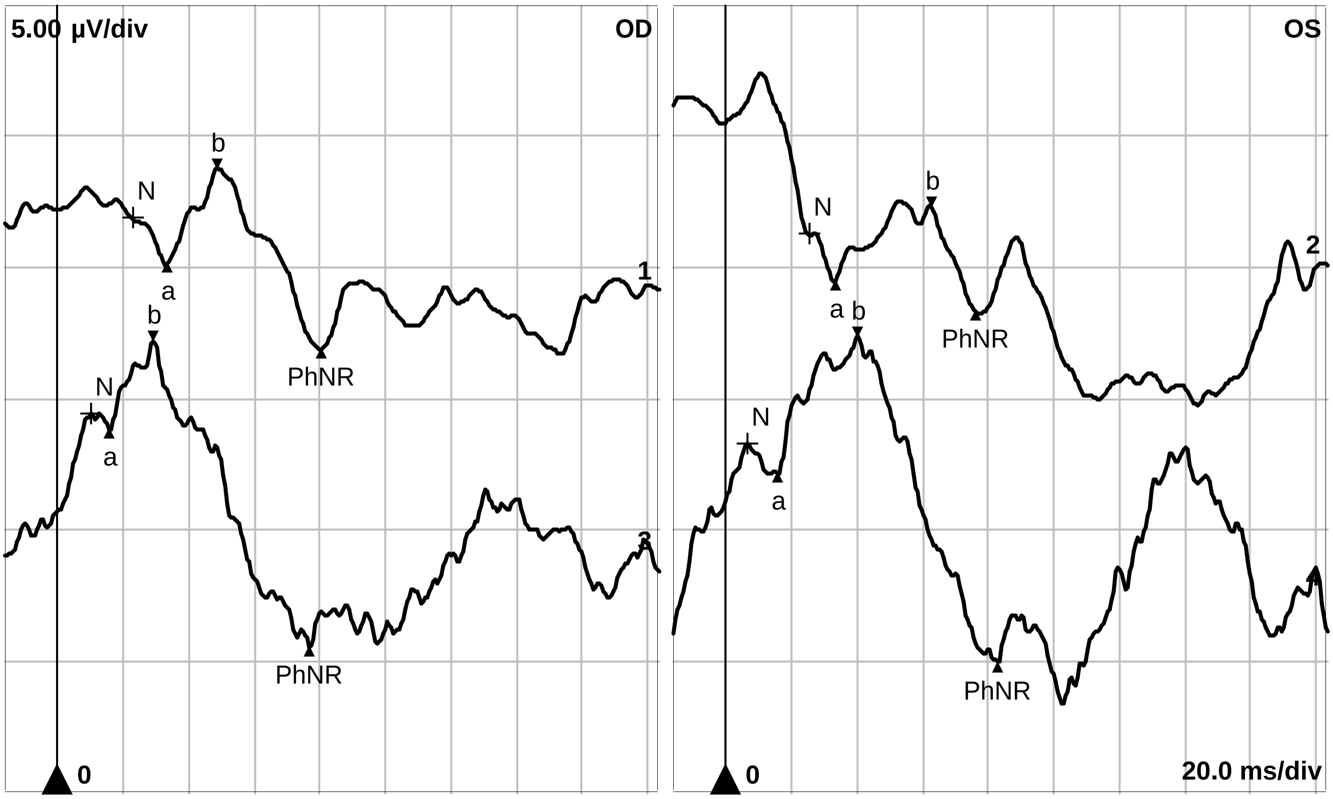

Step	a [ms]	b [ms]	a-wave [µV]	b-wave [µV]	PhNR [µV]	Avgs.
1: OD 1.	33.6	49.2	1.7	3.7	5.0	80
3: OD 2.	16.0	29.3	0.6	3.4	8.9	60
2: OS 1.	33.6	62.9	1.8	2.9	3.0	80
4: OS 2.	16.0	40.6	1.1	5.3	8.4	60

RETIport	Enter Clinic ID<br>Enter your address!					
	Patient:	I-N-2**, 5/7/2021**			Electrode:	DTL Thread
	Examined:	3/26/2025 10:56	Sex/Age:	male/3	Dilated:	Yes
	ID:	3	Operator:		Pupil Size:	-

PhNR Stimulator: Ganzfeld

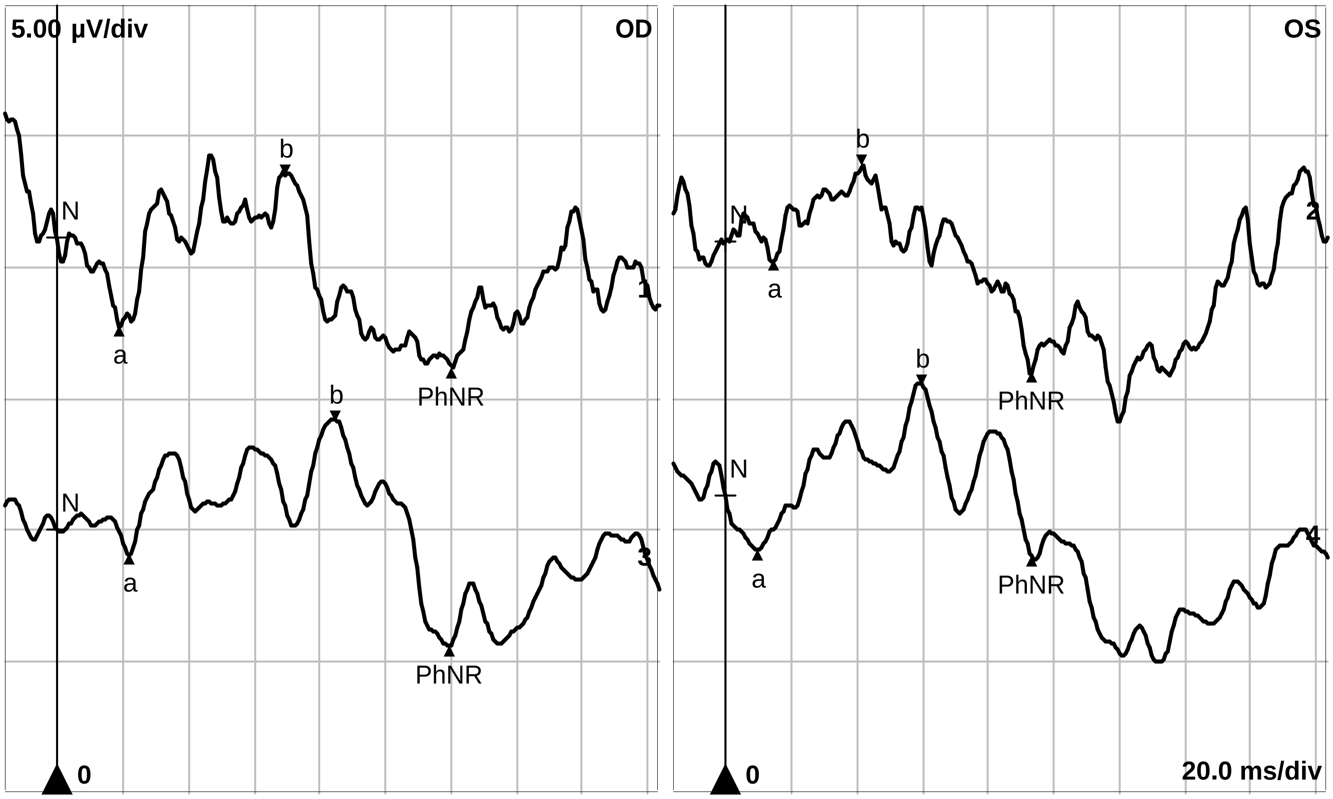

Step	a [ms]	b [ms]	a-wave [µV]	b-wave [µV]	PhNR [µV]	Avgs.
1: OD 1.	19.1	69.9	3.4	5.9	5.0	26
3: OD 2.	21.9	85.2	1.0	5.2	4.5	40
2: OS 1.	14.8	41.8	0.8	3.7	5.1	26
4: OS 2.	10.2	59.8	2.2	6.4	2.4	40

Patient:	d, dd, 5/7/2021			Electrode:	DTL Thread
Examined:	3/26/2025 12:20	Sex/Age:	male/3	Dilated:	Yes
ID:	4	Operator:		Pupil Size:	-

PhNR Stimulator: Ganzfeld

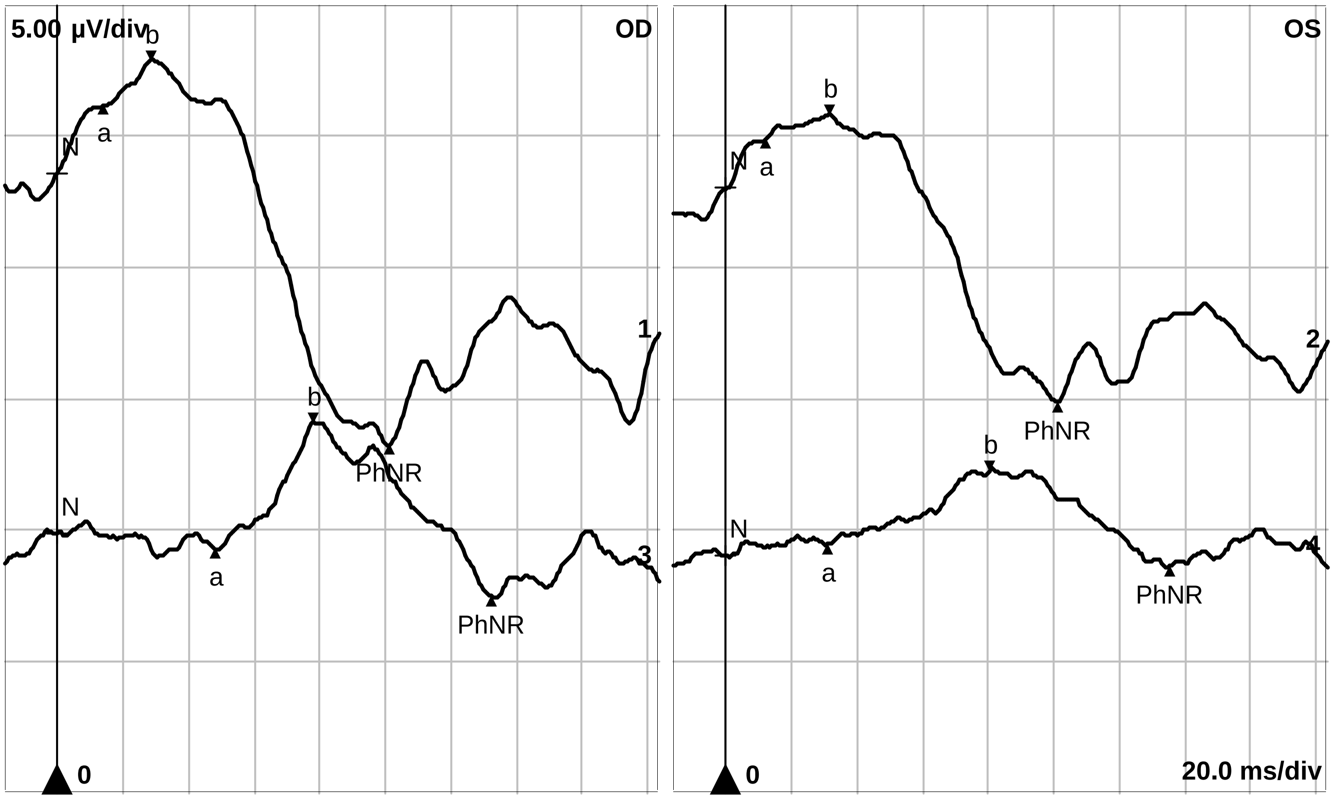

Step	a [ms]	b [ms]	a-wave [µV]	b-wave [µV]	PhNR [µV]	Avgs.
1: OD 1.	14.1	28.9	2.6	1.8	10.4	200
3: OD 2.	48.4	78.5	0.6	4.9	2.4	300
2: OS 1.	12.1	31.6	1.9	1.0	8.2	200
4: OS 2.	31.3	80.5	0.4	2.9	0.4	300

RETIport	Enter Clinic ID<br>Enter your address!					
	Patient:	I-N-6, 5/7/2021			Electrode:	DTL Thread
	Examined:	3/26/2025 12:45	Sex/Age:	male/3	Dilated:	Yes
	ID:	4	Operator:		Pupil Size:	-

PhNR Stimulator: Ganzfeld

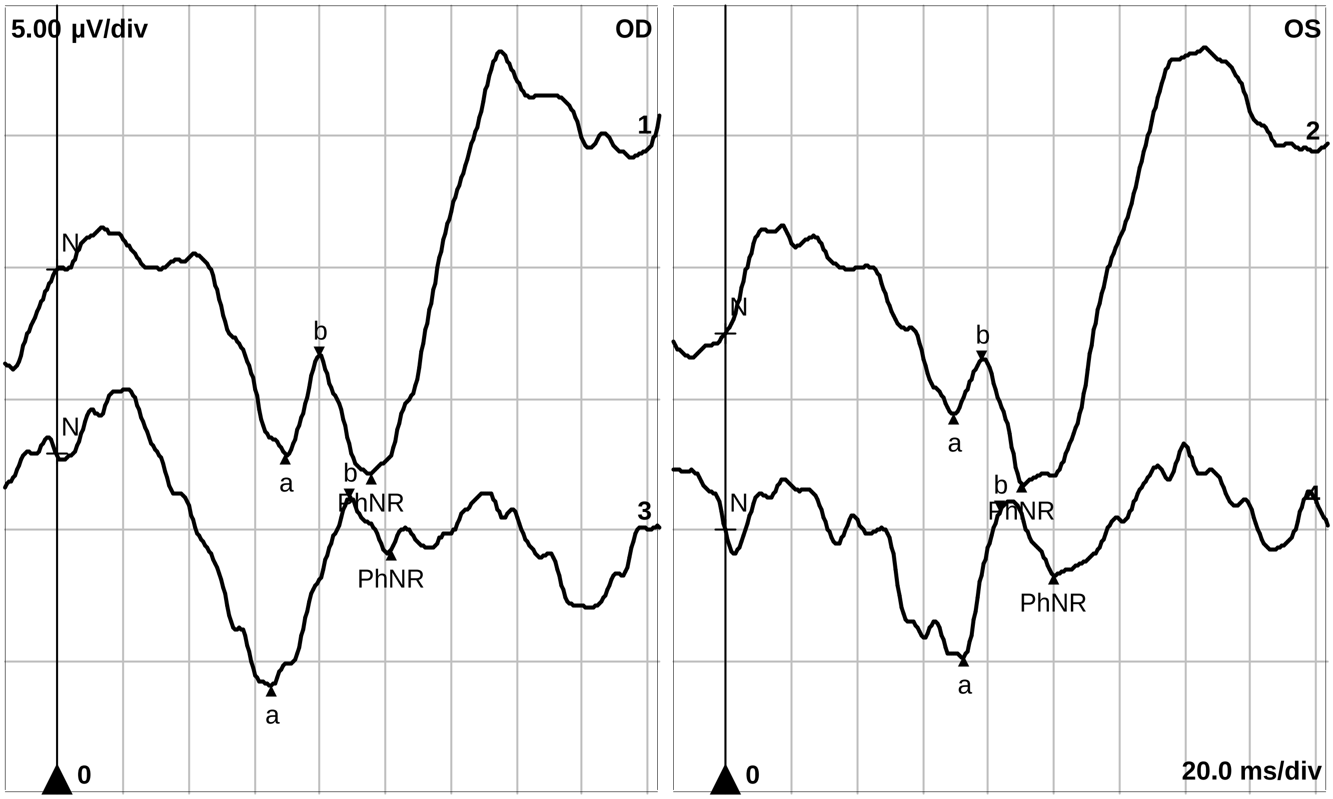

Step	a [ms]	b [ms]	a-wave [µV]	b-wave [µV]	PhNR [µV]	Avgs.
1: OD 1.	69.9	80.1	7.1	3.8	7.8	100
3: OD 2.	65.2	89.5	8.9	7.2	3.7	80
2: OS 1.	69.5	78.5	3.1	2.1	5.7	100
4: OS 2.	73.0	84.0	4.9	5.6	1.8	80

RETIport	Enter Clinic ID<br>Enter your address!					
	Patient:	**2, 2, 4/22/2025**			Electrode:	DTL Thread
	Examined:	4/22/2025 17:35	Sex/Age:	male/0 days	Dilated:	Yes
	ID:	2	Operator:		Pupil Size:	-

PhNR Stimulator: Ganzfeld

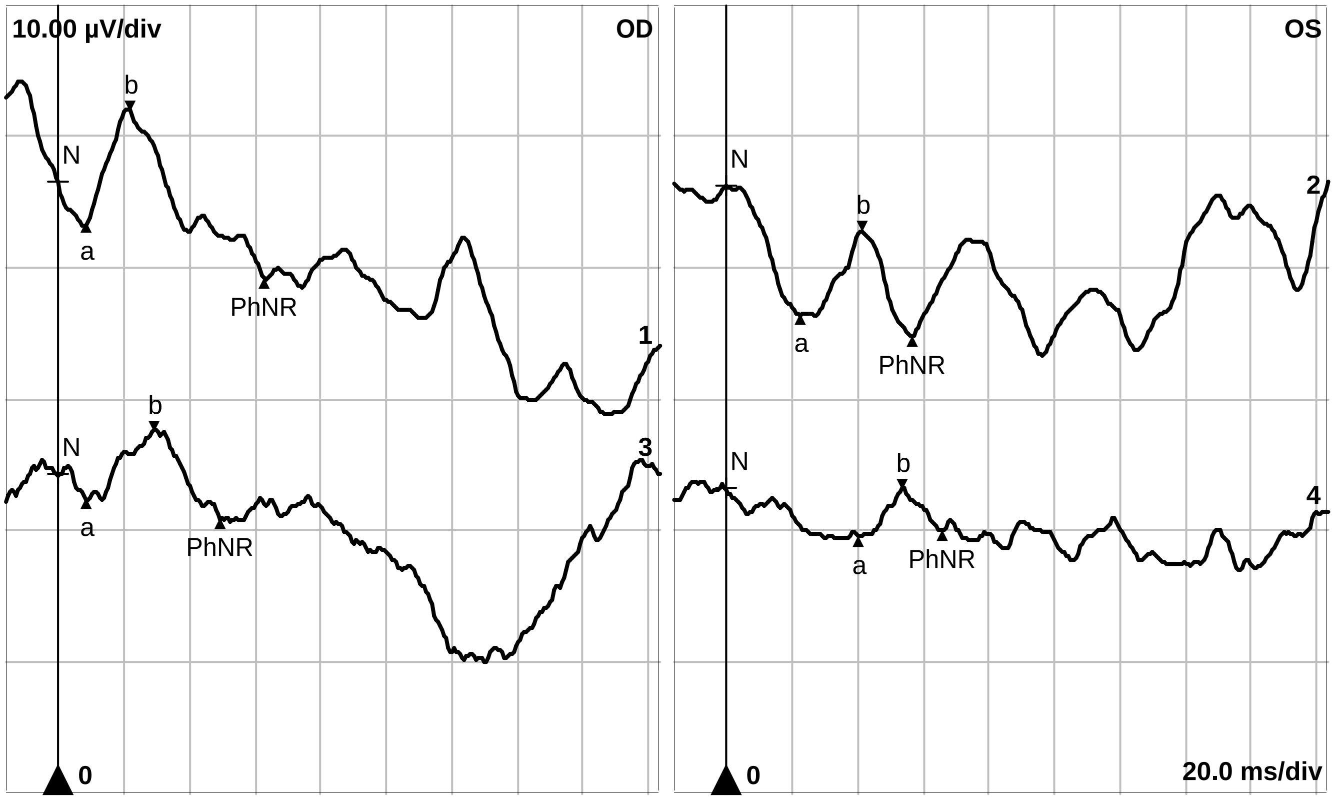

Step	a [ms]	b [ms]	a-wave [µV]	b-wave [µV]	PhNR [µV]	Avgs.
1: OD 1.	8.98	21.9	3.2	8.7	7.5	6
3: OD 2.	8.98	29.3	2.0	5.4	3.4	11
2: OS 1.	22.7	41.4	9.9	6.5	11.5	7
4: OS 2.	40.6	53.9	3.7	3.8	3.2	29

RETIport	Enter Clinic ID<br>Enter your address!				
	Patient: M1 **4/22/2025**			Electrode:	DTL Thread
	Examined: 4/22/2025 17:53	Sex/Age:	male/0 days	Dilated:	Yes
	ID: 2	Operator:		Pupil Size:	-

PhNR Stimulator: Ganzfeld

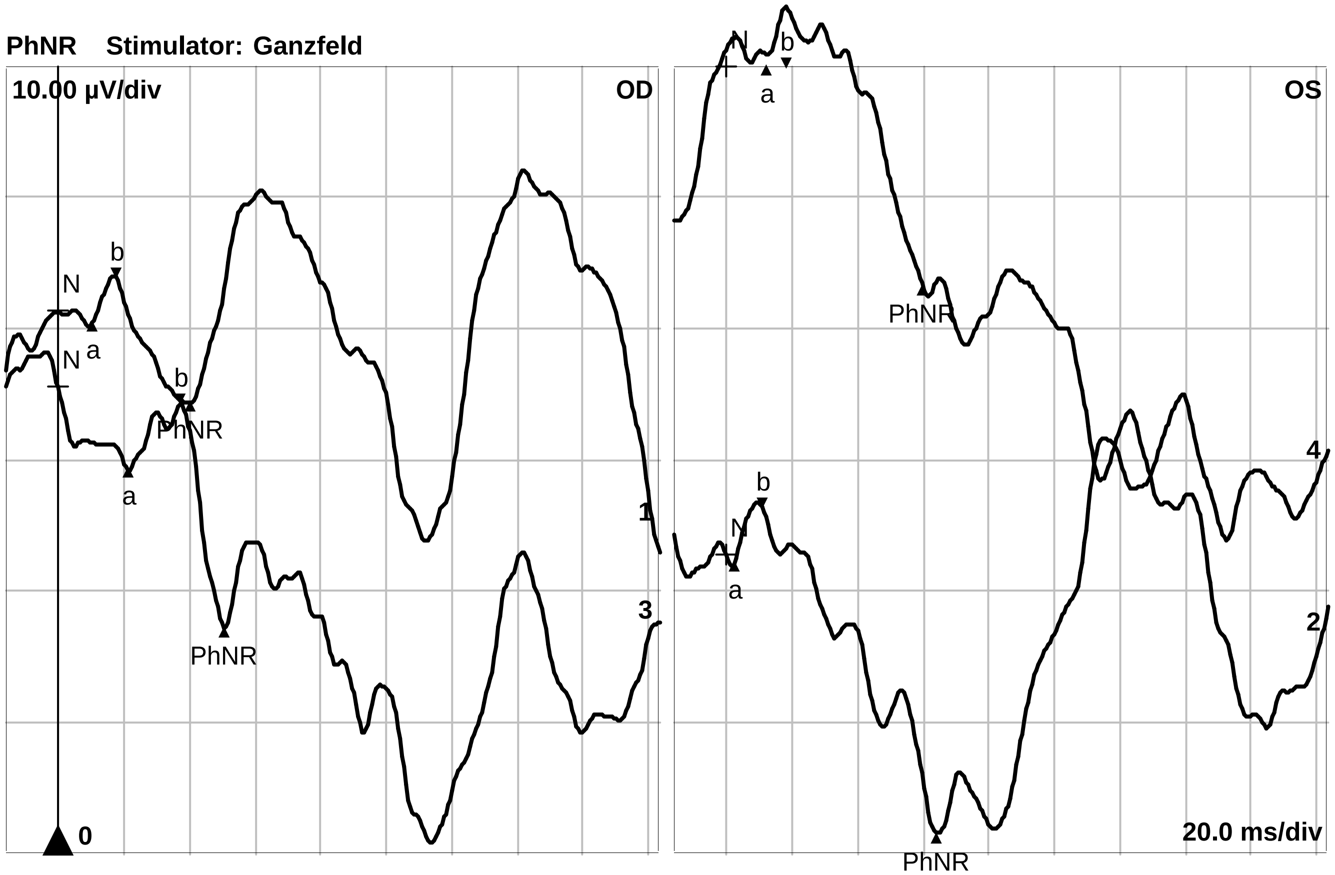

Step	a [ms]	b [ms]	a-wave [µV]	b-wave [µV]	PhNR [µV]	Avgs.
1: OD 1.	10.5	18.0	0.9	3.4	7.0	10
3: OD 2.	21.5	37.5	6.3	5.0	18.5	5
2: OS 1.	12.5	18.4	0.3	3.6	18.0	7
4: OS 2.	2.73	10.9	0.6	4.3	21.3	10

RETIport	Enter Clinic ID<br>Enter your address!					
	Patient:	M2, **4/22/2025**			Electrode:	DTL Thread
	Examined:	4/22/2025 17:59	Sex/Age:	male/0 days	Dilated:	Yes
	ID:	2	Operator:		Pupil Size:	-

PhNR Stimulator: Ganzfeld

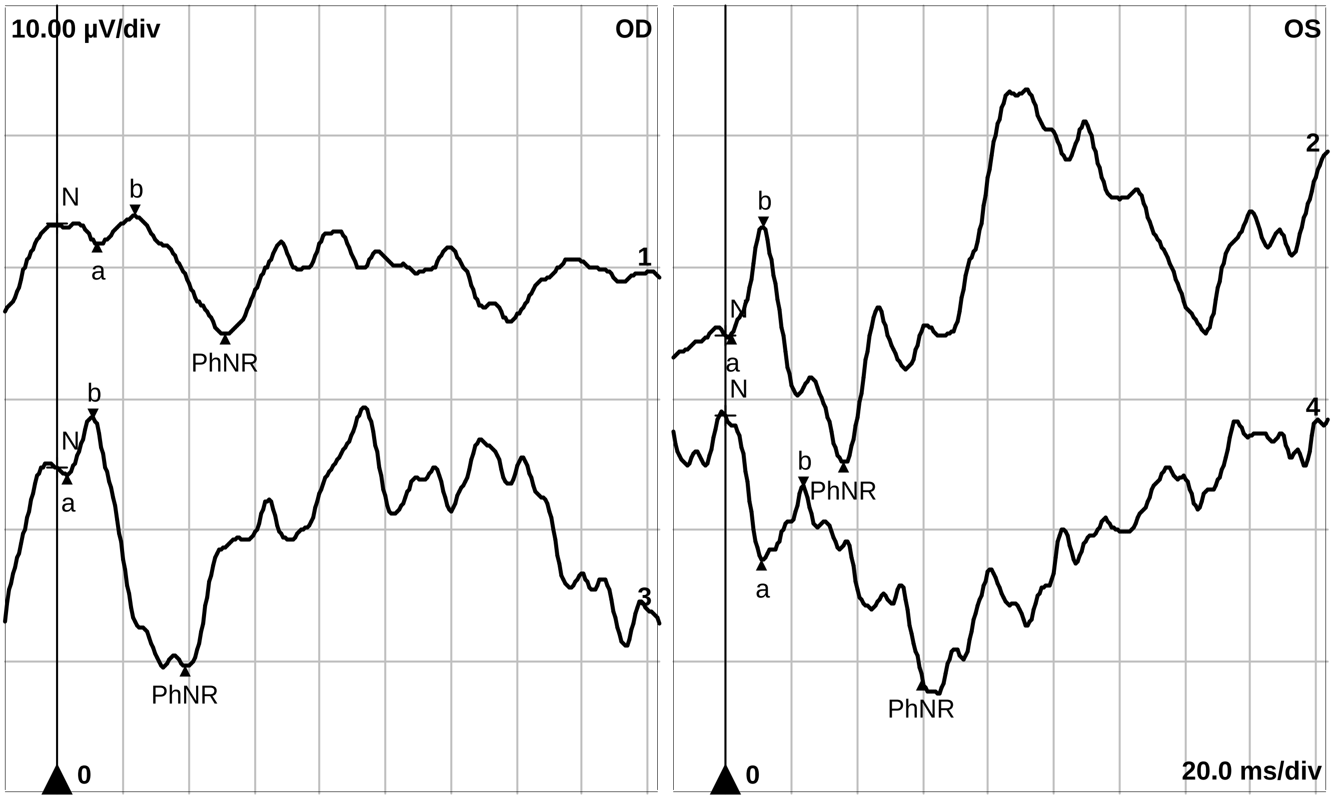

Step	a [ms]	b [ms]	a-wave [µV]	b-wave [µV]	PhNR [µV]	Avgs.
1: OD 1.	12.5	23.8	1.5	2.2	8.5	14
3: OD 2.	3.13	10.9	0.5	4.4	15.1	3
2: OS 1.	1.95	11.7	0.1	8.2	9.7	5
4: OS 2.	11.3	23.8	11.1	5.8	20.2	13

RETIport	Enter Clinic ID<br>Enter your address!					
	Patient:	M3 **4/22/2025**			Electrode:	DTL Thread
	Examined:	4/22/2025 18:18	Sex/Age:	male/0 days	Dilated:	Yes
	ID:	2	Operator:		Pupil Size:	-

PhNR Stimulator: Ganzfeld

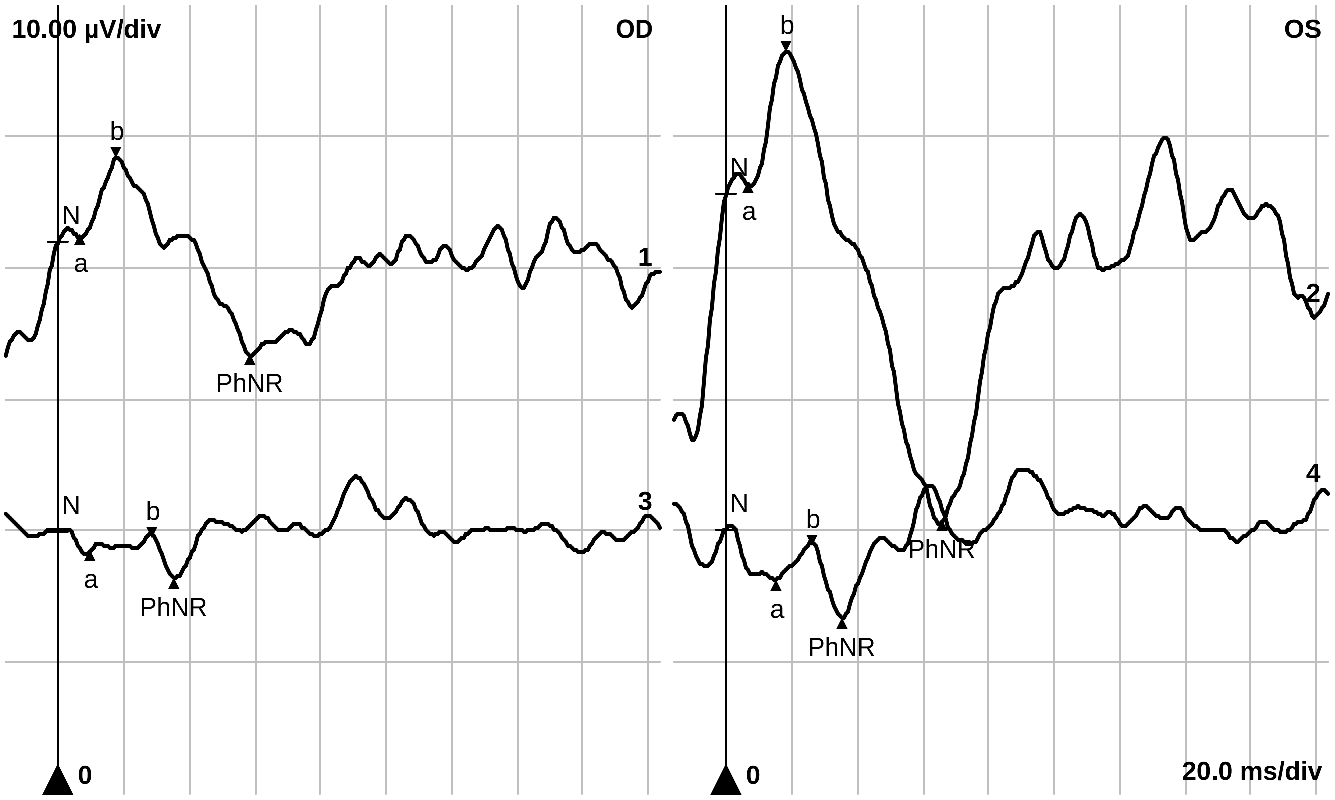

Step	a [ms]	b [ms]	a-wave [µV]	b-wave [µV]	PhNR [µV]	Avgs.
1: OD 1.	7.03	18.0	0.4	6.2	8.7	5
3: OD 2.	10.2	28.9	1.6	1.3	3.6	20
2: OS 1.	6.64	18.8	0.8	10.2	25.0	5
4: OS 2.	15.2	26.6	3.9	2.8	6.8	20

RETIport	Enter Clinic ID<br>Enter your address!					
	Patient:	M4, 4/22/2025			Electrode:	DTL Thread
	Examined:	4/22/2025 18:47	Sex/Age:	male/0 days	Dilated:	Yes
	ID:	2	Operator:		Pupil Size:	-

PhNR Stimulator: Ganzfeld

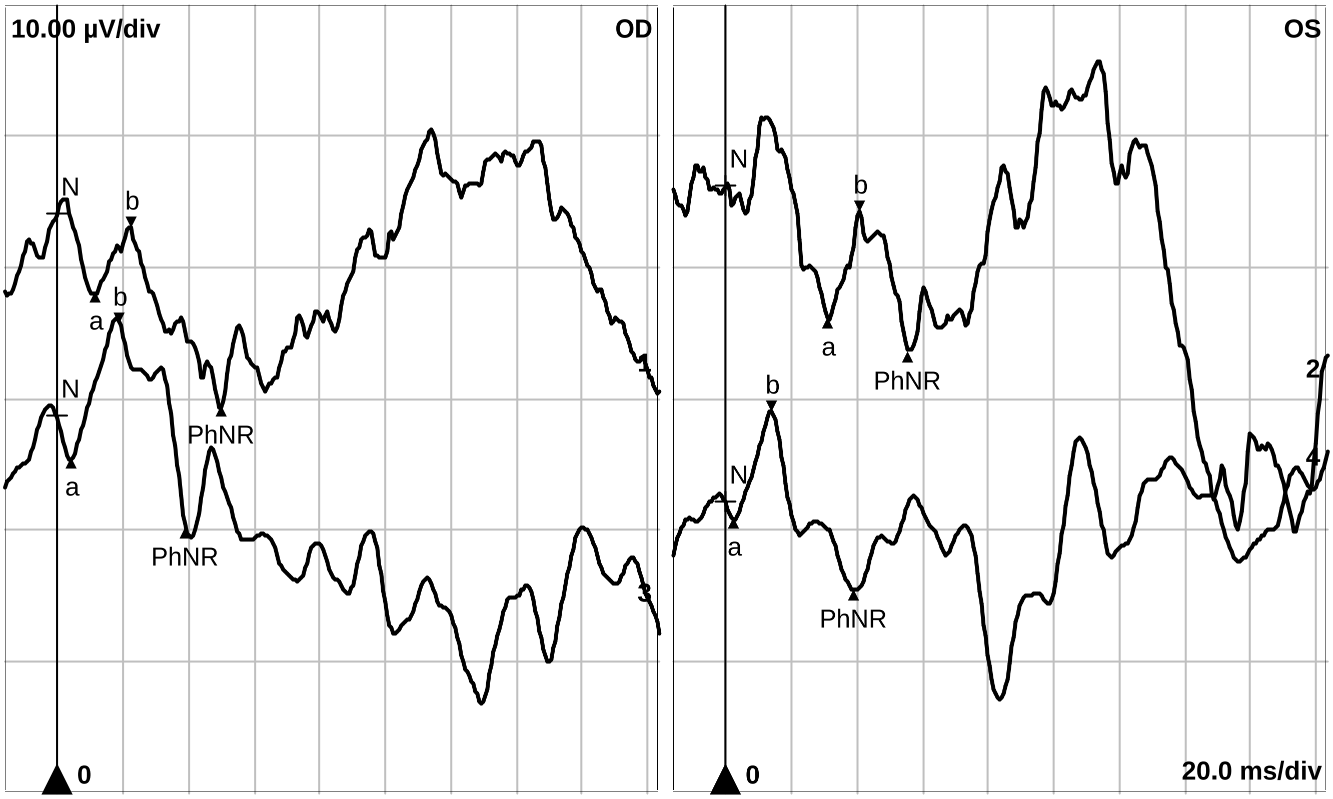

Step	a [ms]	b [ms]	a-wave [µV]	b-wave [µV]	PhNR [µV]	Avgs.
1: OD 1.	11.7	22.7	6.1	5.2	14.8	6
3: OD 2.	4.30	19.1	3.3	10.5	8.6	3
2: OS 1.	31.3	41.0	10.2	8.3	12.8	3
4: OS 2.	2.73	14.1	1.3	8.3	6.9	5

RETIport	Enter Clinic ID<br>Enter your address!					
	Patient:	M5 **4/22/2025**			Electrode:	DTL Thread
	Examined:	4/22/2025 18:25	Sex/Age:	male/0 days	Dilated:	Yes
	ID:	2	Operator:		Pupil Size:	-

PhNR Stimulator: Ganzfeld

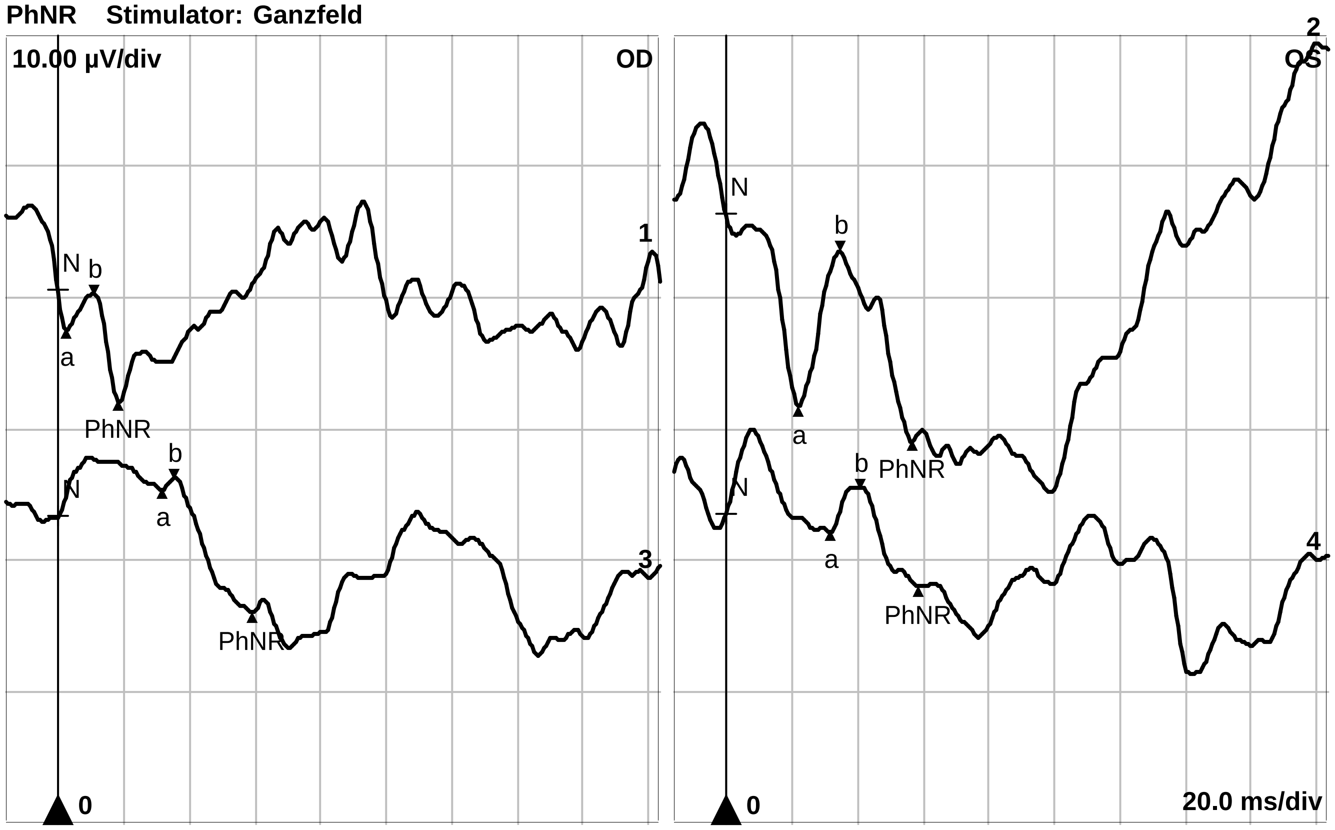

Step	a [ms]	b [ms]	a-wave [µV]	b-wave [µV]	PhNR [µV]	Avgs.
1: OD 1.	2.73	11.3	3.1	2.8	8.5	7
3: OD 2.	32.0	35.5	2.1	0.9	7.4	20
2: OS 1.	22.3	34.8	14.6	11.9	17.4	9
4: OS 2.	31.6	41.0	1.4	3.4	5.6	20

RETIport	Enter Clinic ID<br>Enter your address!					
	Patient:	M6, **4/22/2025**			Electrode:	DTL Thread
	Examined:	4/22/2025 19:15	Sex/Age:	male/0 days	Dilated:	Yes
	ID:	2	Operator:		Pupil Size:	-

PhNR Stimulator: Ganzfeld

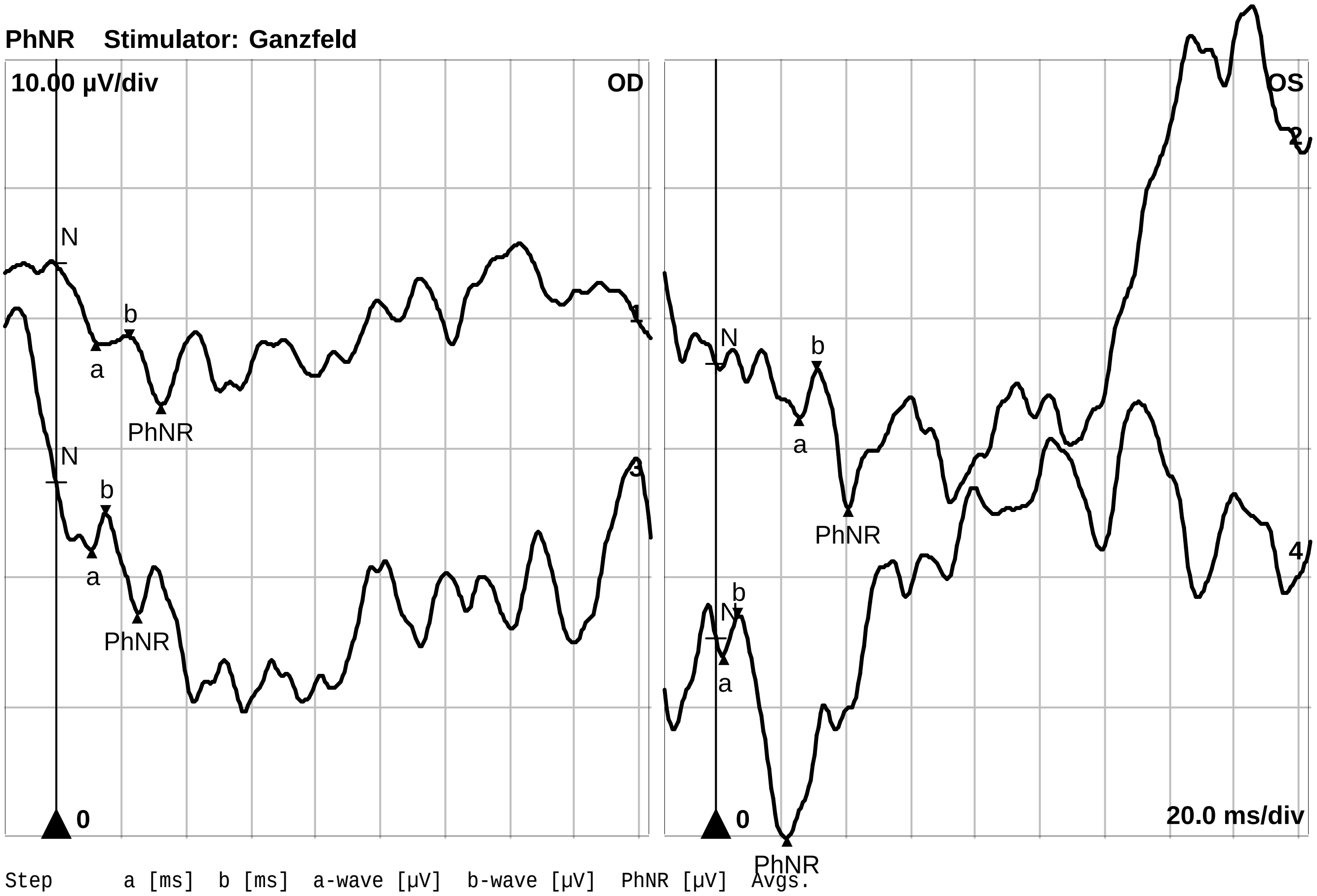


```
Step       a [ms]   b [ms]   a-wave [µV]   b-wave [µV]   PhNR [µV]   Avgs.
1: OD 1.    12.1     23.0      6.1            0.4          10.9        10
3: OD 2.    10.9     15.6      5.2            2.8          10.1         3
2: OS 1.    25.8     31.3      4.2            3.7          11.2         2
4: OS 2.    2.34     7.03      1.3            3.0          15.5         3
```

RETIport	Enter Clinic ID<br>Enter your address!

Patient:	M-N-1, **4/22/2025**			Electrode:	DTL Thread
Examined:	4/22/2025 17:21	Sex/Age:	male/0 days	Dilated:	Yes
ID:	2	Operator:		Pupil Size:	-

PhNR Stimulator: Ganzfeld

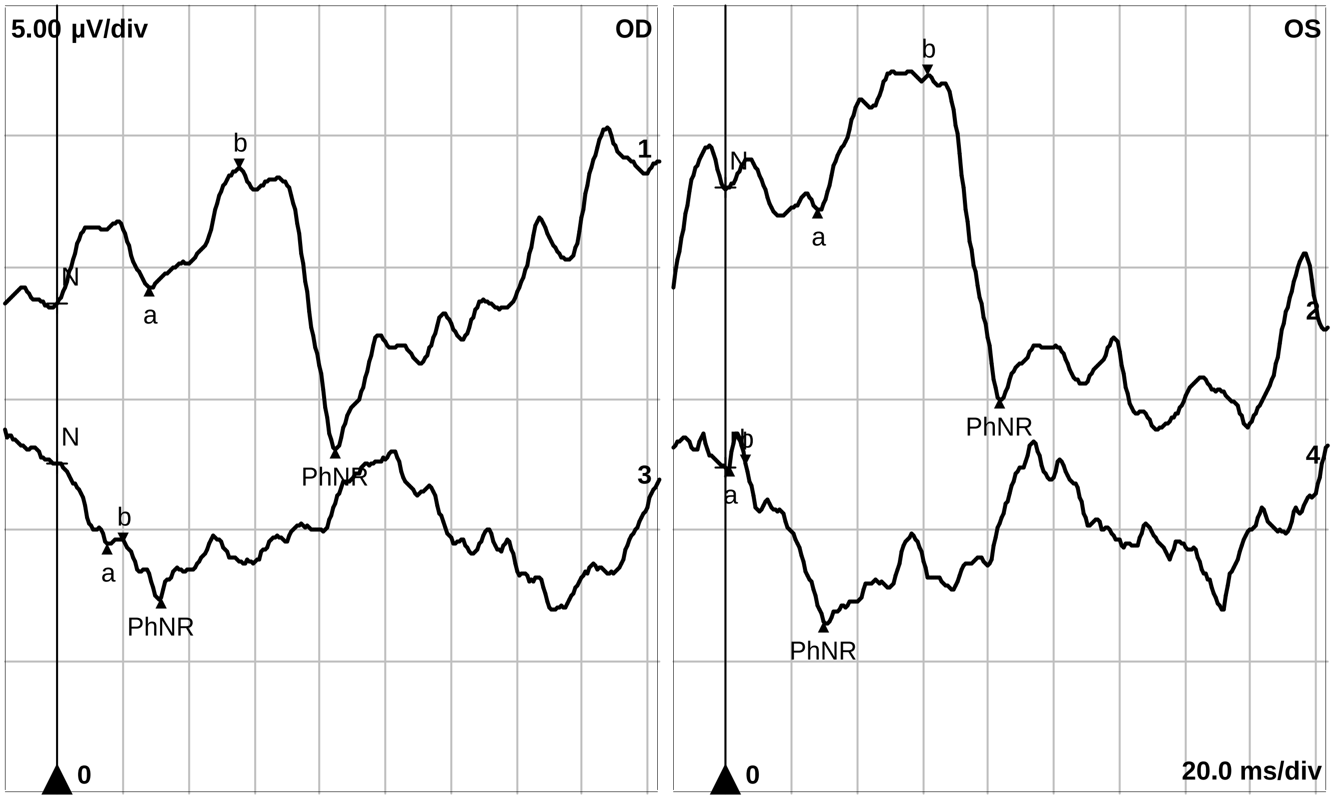

Step	a [ms]	b [ms]	a-wave [µV]	b-wave [µV]	PhNR [µV]	Avgs.
1: OD 1.	28.5	55.9	0.6	4.6	5.6	20
3: OD 2.	15.6	20.3	3.1	0.1	5.2	60
2: OS 1.	28.5	61.7	0.8	5.1	8.1	20
4: OS 2.	1.17	6.25	0.0	0.2	5.9	60

RETIport	Enter Clinic ID<br>Enter your address!					
	Patient:	M-N-2 **4/22/2025**			Electrode:	DTL Thread
	Examined:	4/22/2025 17:25	Sex/Age:	male/0 days	Dilated:	Yes
	ID:	2	Operator:		Pupil Size:	-

PhNR Stimulator: Ganzfeld

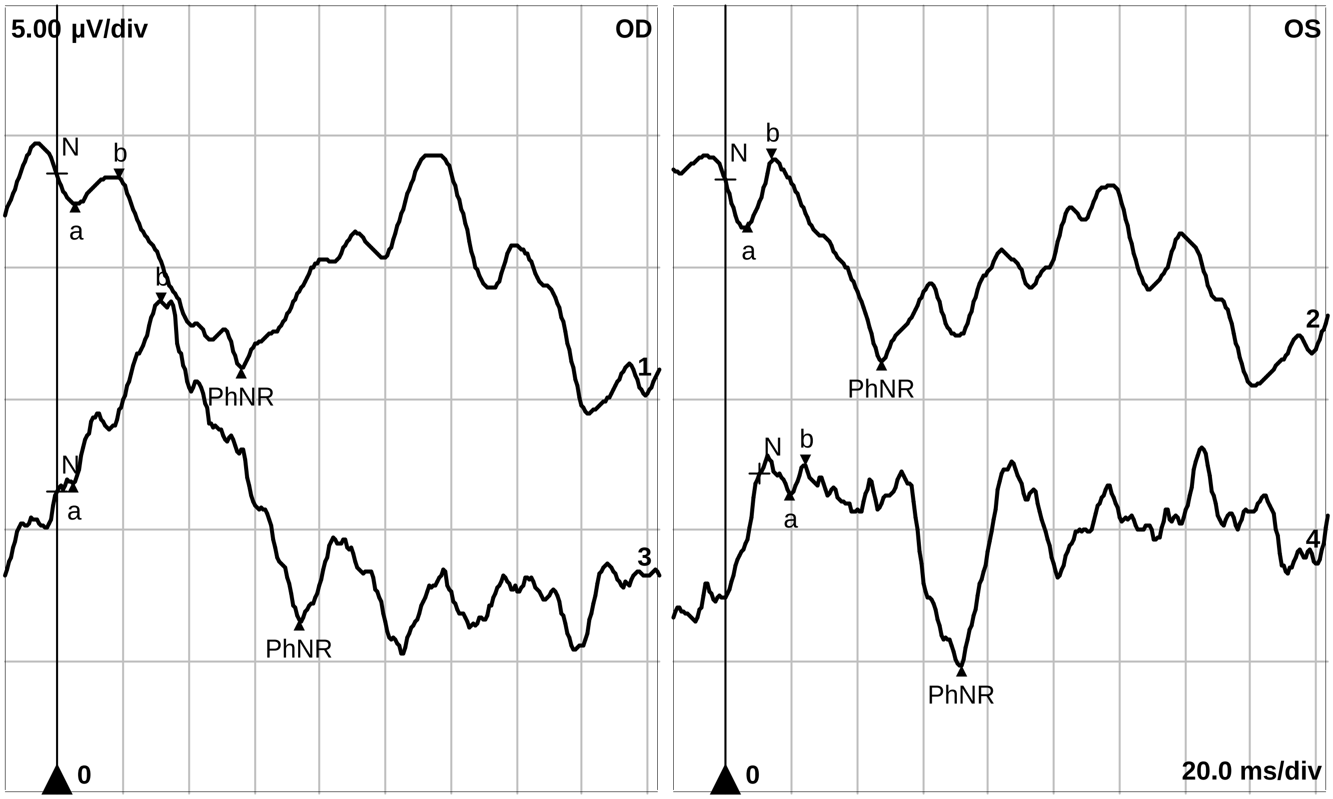

Step	a [ms]	b [ms]	a-wave [µV]	b-wave [µV]	PhNR [µV]	Avgs.
1: OD 1.	5.86	19.1	1.1	1.0	7.4	20
3: OD 2.	5.08	32.0	0.3	6.9	5.0	20
2: OS 1.	7.03	14.5	1.7	2.5	7.0	20
4: OS 2.	19.5	24.6	0.7	1.1	7.3	20

**RETIport**	**Enter Clinic ID**<br>Enter your address!			
	Patient: M-N-3, **4/22/2025**		Electrode:	DTL Thread
	Examined: 4/22/2025 17:30	Sex/Age: male/0 days	Dilated:	Yes
	ID: 2	Operator:	Pupil Size:	-

PhNR Stimulator: Ganzfeld

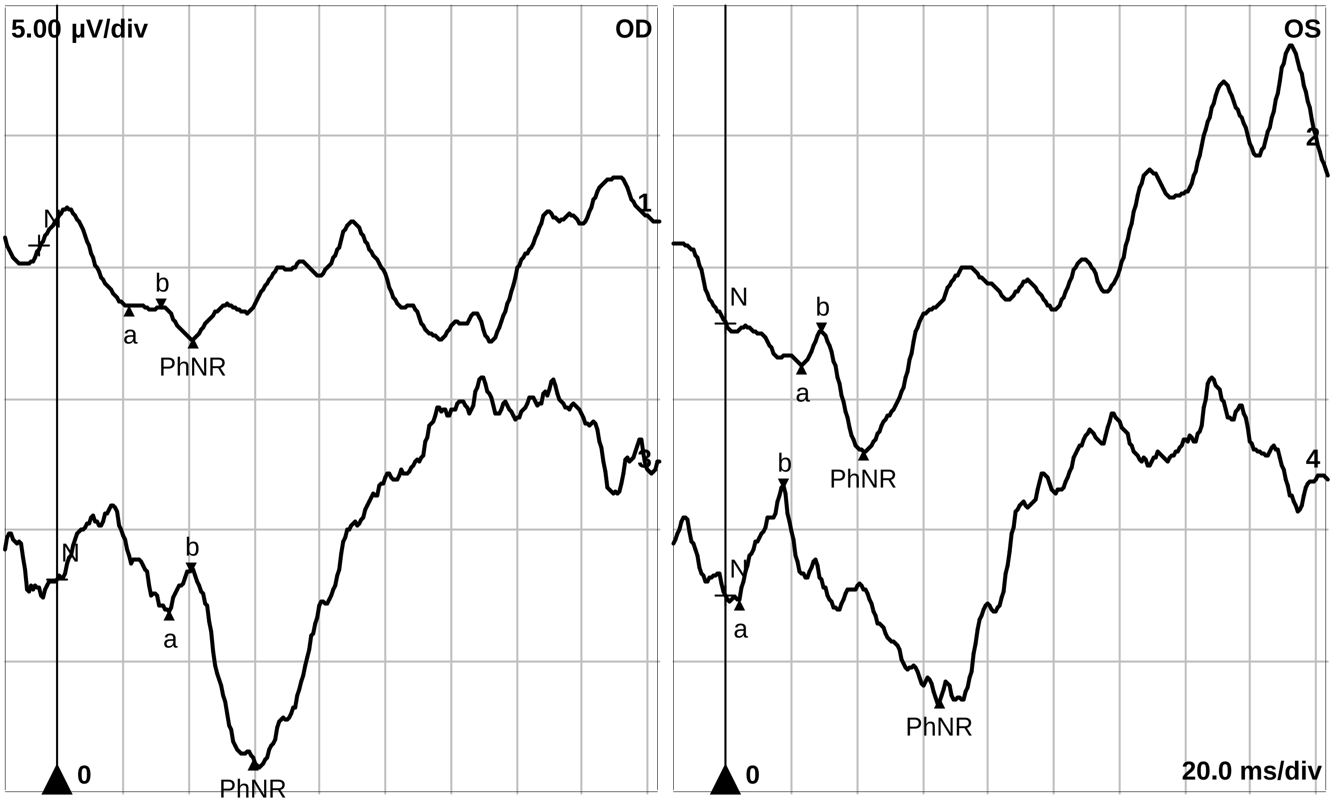

Step	a [ms]	b [ms]	a-wave [µV]	b-wave [µV]	PhNR [µV]	Avgs.
1: OD 1.	22.3	32.0	2.4	0.0	3.6	27
3: OD 2.	34.4	41.0	1.2	1.6	6.9	20
2: OS 1.	23.4	29.3	1.6	1.3	4.9	27
4: OS 2.	4.30	18.0	0.2	4.3	4.0	20

RETIport	Enter Clinic ID<br>Enter your address!					
	Patient:	M-N-4, **4/22/2025**			Electrode:	DTL Thread
	Examined:	4/22/2025 19:12	Sex/Age:	male/0 days	Dilated:	Yes
	ID:	2	Operator:		Pupil Size:	-

PhNR Stimulator: Ganzfeld

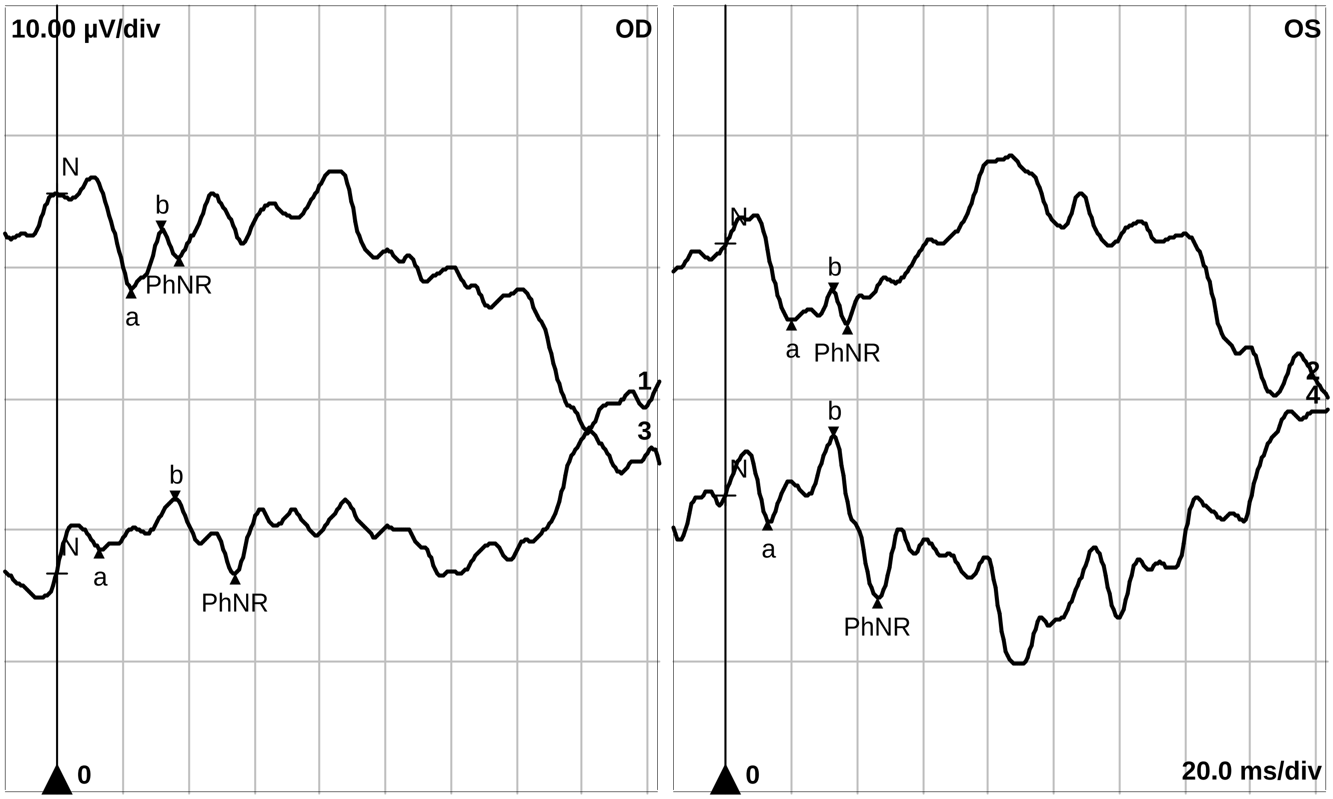

Step	a [ms]	b [ms]	a-wave [µV]	b-wave [µV]	PhNR [µV]	Avgs.
1: OD 1.	22.7	32.0	7.4	4.6	4.9	6
3: OD 2.	12.9	35.9	1.7	3.9	0.2	10
2: OS 1.	20.3	33.2	5.9	2.2	6.2	6
4: OS 2.	13.3	33.2	2.0	6.6	7.9	4

RETIport	Enter Clinic ID<br>Enter your address!

Patient:	M-N-6, **5/7/2021**			Electrode:	DTL Thread
Examined:	3/26/2025 12:35	Sex/Age:	male/3	Dilated:	Yes
ID:	4	Operator:		Pupil Size:	-

PhNR Stimulator: Ganzfeld

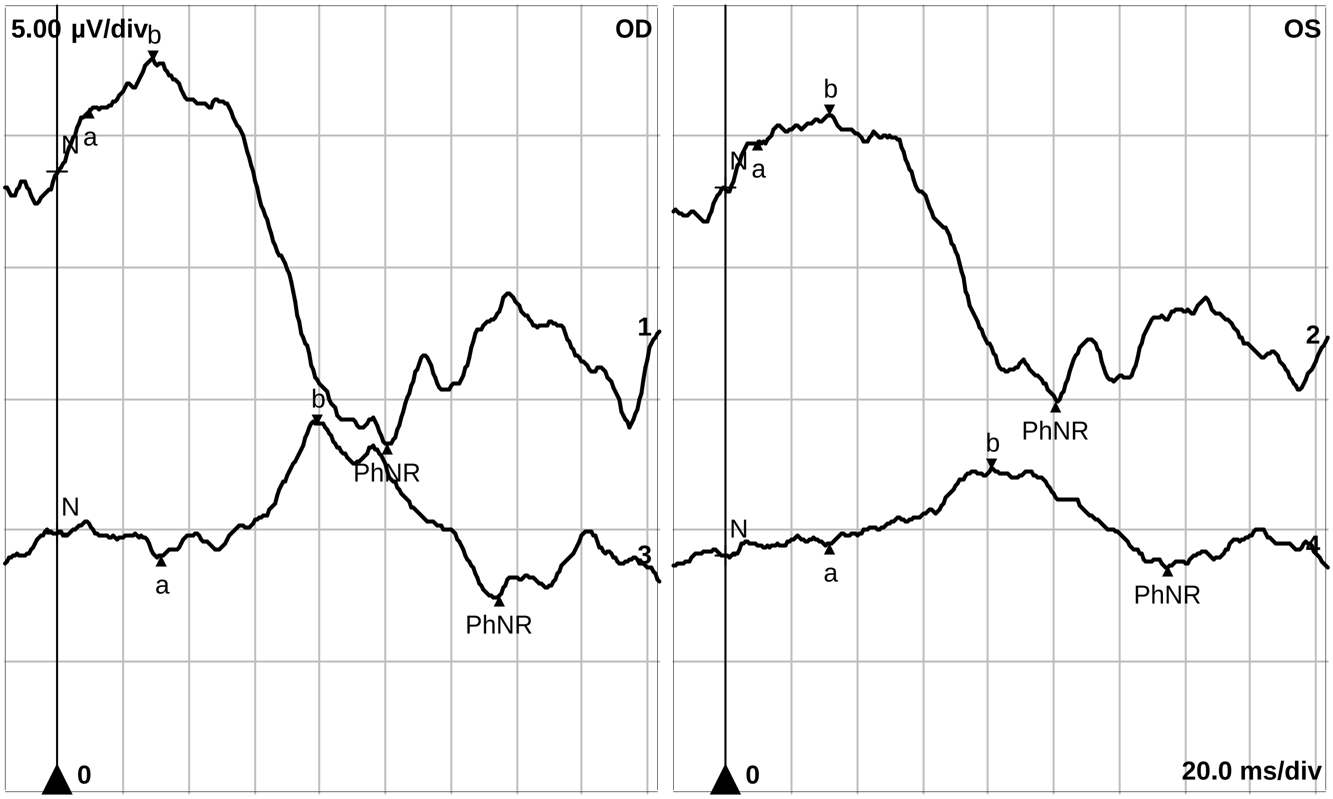

Step	a [ms]	b [ms]	a-wave [µV]	b-wave [µV]	PhNR [µV]	Avgs.
1: OD 1.	10.2	29.3	2.4	1.9	10.4	200
3: OD 2.	31.6	79.3	0.9	5.1	2.4	300
2: OS 1.	10.2	32.0	1.7	1.1	8.2	200
4: OS 2.	31.6	81.6	0.4	3.0	0.5	300

RETIport	Enter Clinic ID<br>Enter your address!					
	Patient:	M-N-6, **5/7/2021**			Electrode:	DTL Thread
	Examined:	3/26/2025 12:57	Sex/Age:	male/3	Dilated:	Yes
	ID:	4	Operator:		Pupil Size:	-

PhNR Stimulator: Ganzfeld

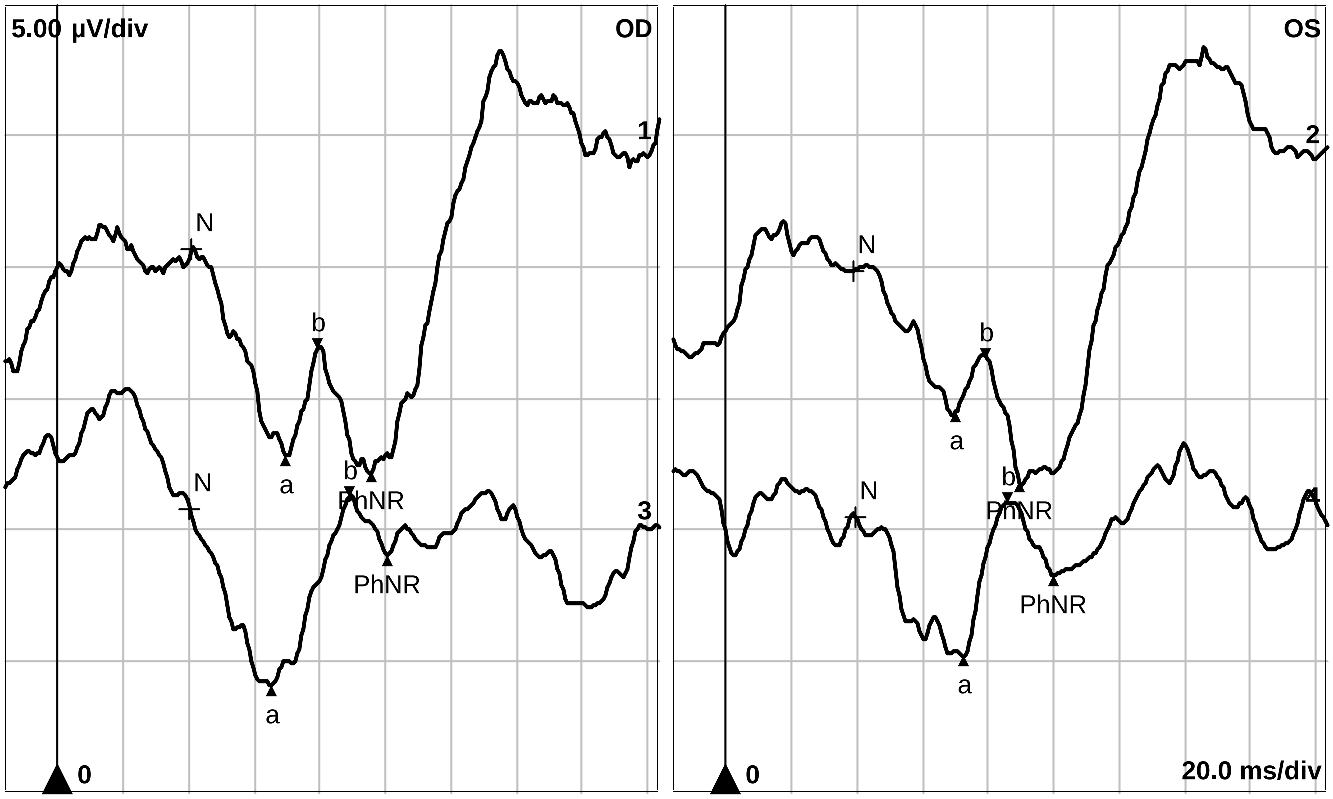

Step	a [ms]	b [ms]	a-wave [µV]	b-wave [µV]	PhNR [µV]	Avgs.
1: OD 1.	69.9	79.7	7.9	4.2	8.5	100
3: OD 2.	65.6	89.1	6.8	7.3	1.8	80
2: OS 1.	70.3	79.3	5.4	2.1	8.1	100
4: OS 2.	73.0	85.9	5.4	5.9	2.3	80
