# Supplementary material for: Adipose stem cells derived extracellular vesicles alleviate retinal excitotoxicity via miR-23a-5p/PLCD1/PKCA/GluA2 axis: a potential therapeutic strategy
Source: Stem Cell Res Ther. 2026 Jun 30;17:321. doi: 10.1186/s13287-026-05140-z (PMC13587503; doi:10.1186/s13287-026-05140-z)
Supplement: Supplementary file 1 [file 13287_2026_5140_MOESM1_ESM.zip › 13287_2026_5140_MOESM1_ESM/13287_2026_5140_MOESM8_ESM.pdf]

NOTE: Please save this file locally before filling in the table, DO NOT work on the file within your internet browser as changes will not be saved. Adobe Acrobat Reader (available free here) is recommended for completion.

The ARRIVE guidelines 2.0: author checklist

The ARRIVE Essential 10

These items are the basic minimum to include in a manuscript. Without this information, readers and reviewers cannot assess the reliability of the findings.

Item		Recommendation	Section/line number, or reason for not reporting
**Study design**	1	For each experiment, provide brief details of study design including:	
		a. The groups being compared, including control groups. If no control group has been used, the rationale should be stated.	Line 171-176
		b. The experimental unit (e.g. a single animal, litter, or cage of animals).	A single animal
**Sample size**	2	a. Specify the exact number of experimental units allocated to each group, and the total number in each experiment. Also indicate the total number of animals used.	line 176-177
		b. Explain how the sample size was decided. Provide details of any *a priori* sample size calculation, if done.	ct sizes for visual function and RGC survival. Accordingly, a sample size of 14 animals per group was chosen to ensure adequate statistical power while minimizing unnecessary animal usage, in compliance with the 3R principles.
**Inclusion and exclusion criteria**	3	a. Describe any criteria used for including and excluding animals (or experimental units) during the experiment, and data points during the analysis. Specify if these criteria were established *a priori*. If no criteria were set, state this explicitly.	Line 169-170
		b. For each experimental group, report any animals, experimental units or data points not included in the analysis and explain why. If there were no exclusions, state so.	Line 170-171
		c. For each analysis, report the exact value of *n* in each experimental group.	Figure legends : line 497-571
**Randomisation**	4	a. State whether randomisation was used to allocate experimental units to control and treatment groups. If done, provide the method used to generate the randomisation sequence.	Line 171-172
		b. Describe the strategy used to minimise potential confounders such as the order of treatments and measurements, or animal/cage location. If confounders were not controlled, state this explicitly.	ent, as these factors were not considered to be significant sources of variation in this study design. However, all animals were housed under identical environmental conditions (12-h light/dark cycle, 22 ± 2 °C, ad libitum access to food and water) to minimize environmental variability.
**Blinding**	5	Describe who was aware of the group allocation at the different stages of the experiment (during the allocation, the conduct of the experiment, the outcome assessment, and the data analysis).	Line 178-179
**Outcome measures**	6	a. Clearly define all outcome measures assessed (e.g. cell death, molecular markers, or behavioural changes).	This study set 24 hours post-modeling as the definitive endpoint of the experiment, based on the timing of RGC death reported in previous studies [26]. Line 168-169
		b. For hypothesis-testing studies, specify the primary outcome measure, i.e. the outcome measure that was used to determine the sample size.	-
**Statistical methods**	7	a. Provide details of the statistical methods used for each analysis, including software used.	Line 270-277
		b. Describe any methods used to assess whether the data met the assumptions of the statistical approach, and what was done if the assumptions were not met.	Line 270-277
**Experimental animals**	8	a. Provide species-appropriate details of the animals used, including species, strain and substrain, sex, age or developmental stage, and, if relevant, weight.	Line 88-96
		b. Provide further relevant information on the provenance of animals, health/immune status, genetic modification status, genotype, and any previous procedures.	Line 88-96
**Experimental procedures**	9	For each experimental group, including controls, describe the procedures in enough detail to allow others to replicate them, including:	
		a. What was done, how it was done and what was used.	Fig 1
		b. When and how often.	Line 141-151;Line 164-180
		c. Where (including detail of any acclimatisation periods).	Line 141-151;Line 164-180
		d. Why (provide rationale for procedures).	Line 141-151;Line 164-180
**Results**	10	For each experiment conducted, including independent replications, report:	
		a. Summary/descriptive statistics for each experimental group, with a measure of variability where applicable (e.g. mean and SD, or median and range).	The results of each experimental group in each experiment are presented as bar graphs showing the mean and standard deviation, with the specific P-values for statistical differences described separately.
		b. If applicable, the effect size with a confidence interval.	-

The Recommended Set

These items complement the Essential 10 and add important context to the study. Reporting the items in both sets represents best practice.

Item		Recommendation	Section/line number, or reason for not reporting
**Abstract**	11	Provide an accurate summary of the research objectives, animal species, strain and sex, key methods, principal findings, and study conclusions.	Line 22-51
**Background**	12	a. Include sufficient scientific background to understand the rationale and context for the study, and explain the experimental approach.	Line 57-86
		b. Explain how the animal species and model used address the scientific objectives and, where appropriate, the relevance to human biology.	Line 64-68
**Objectives**	13	Clearly describe the research question, research objectives and, where appropriate, specific hypotheses being tested.	Line 82-86
**Ethical statement**	14	Provide the name of the ethical review committee or equivalent that has approved the use of animals in this study, and any relevant licence or protocol numbers (if applicable). If ethical approval was not sought or granted, provide a justification.	Line 574-580
**Housing and husbandry**	15	Provide details of housing and husbandry conditions, including any environmental enrichment.	Line 90-92
**Animal care and monitoring**	16	a. Describe any interventions or steps taken in the experimental protocols to reduce pain, suffering and distress.	Line 89-96
		b. Report any expected or unexpected adverse events.	None
		c. Describe the humane endpoints established for the study, the signs that were monitored and the frequency of monitoring. If the study did not have humane endpoints, state this.	The experimental model had a short duration, and no hu mane endpoint criteria were established.
**Interpretation/ scientific implications**	17	a. Interpret the results, taking into account the study objectives and hypotheses, current theory and other relevant studies in the literature.	Results secti on
		b. Comment on the study limitations including potential sources of bias, limitations of the animal model, and imprecision associated with the results.	Line 447-456
**Generalisability/ translation**	18	Comment on whether, and how, the findings of this study are likely to generalise to other species or experimental conditions, including any relevance to human biology (where appropriate).	Line 448-450
**Protocol registration**	19	Provide a statement indicating whether a protocol (including the research question, key design features, and analysis plan) was prepared before the study, and if and where this protocol was registered.	Line 406-407[22]
**Data access**	20	Provide a statement describing if and where study data are available.	The small RNA sequencing data have been up
**Declaration of interests**	21	a. Declare any potential conflicts of interest, including financial and non-financial. If none exist, this should be stated.	Line 588-600
		b. List all funding sources (including grant identifier) and the role of the funder(s) in the design, analysis and reporting of the study.	Line 601-604
