# Supplementary material for: Adipose stem cells derived extracellular vesicles alleviate retinal excitotoxicity via miR-23a-5p/PLCD1/PKCA/GluA2 axis: a potential therapeutic strategy
Source: Stem Cell Res Ther. 2026 Jun 30;17:321. doi: 10.1186/s13287-026-05140-z (PMC13587503; doi:10.1186/s13287-026-05140-z)
Supplement: Supplementary file 1 [file 13287_2026_5140_MOESM1_ESM.zip › 13287_2026_5140_MOESM1_ESM/Fig.1.docx]

**Supplementary Fig. 1** Dose-response effects of ADSC-EVs and miR-23a-5p modulators. **(A and B)** Uptake of ADSC-EVs by R28 cells shows a dose-dependent increase, sample size = 3, ***p*  <  0.01. **(C and D)** Protective effects of varying concentrations of ADSC-EVs against glutamate-induced cell death in R28 cells, sample size = 3, **p*  <  0.05. **(E and F)** Transfection efficiency of a miR-23a-5p mimic or inhibitor across a range of concentrations in R28 cells, determined by qRT-PCR, sample size = 3, ****p*  <  0.001, *****p*  <  0.0001 versus mimic-NC or inhibitor-NC group by one-way ANOVA followed by Tukey’ s or Dunnett's test. Values are expressed as mean ± SD.
