# Supplementary material for: Adipose stem cells derived extracellular vesicles alleviate retinal excitotoxicity via miR-23a-5p/PLCD1/PKCA/GluA2 axis: a potential therapeutic strategy
Source: Stem Cell Res Ther. 2026 Jun 30;17:321. doi: 10.1186/s13287-026-05140-z (PMC13587503; doi:10.1186/s13287-026-05140-z)
Supplement: Supplementary file 1 [file 13287_2026_5140_MOESM1_ESM.zip › 13287_2026_5140_MOESM1_ESM/13287_2026_5140_MOESM7_ESM.zip!/The RNA-seq data/1.2.QCReport/EVs_1.duplication.distribution.pdf]

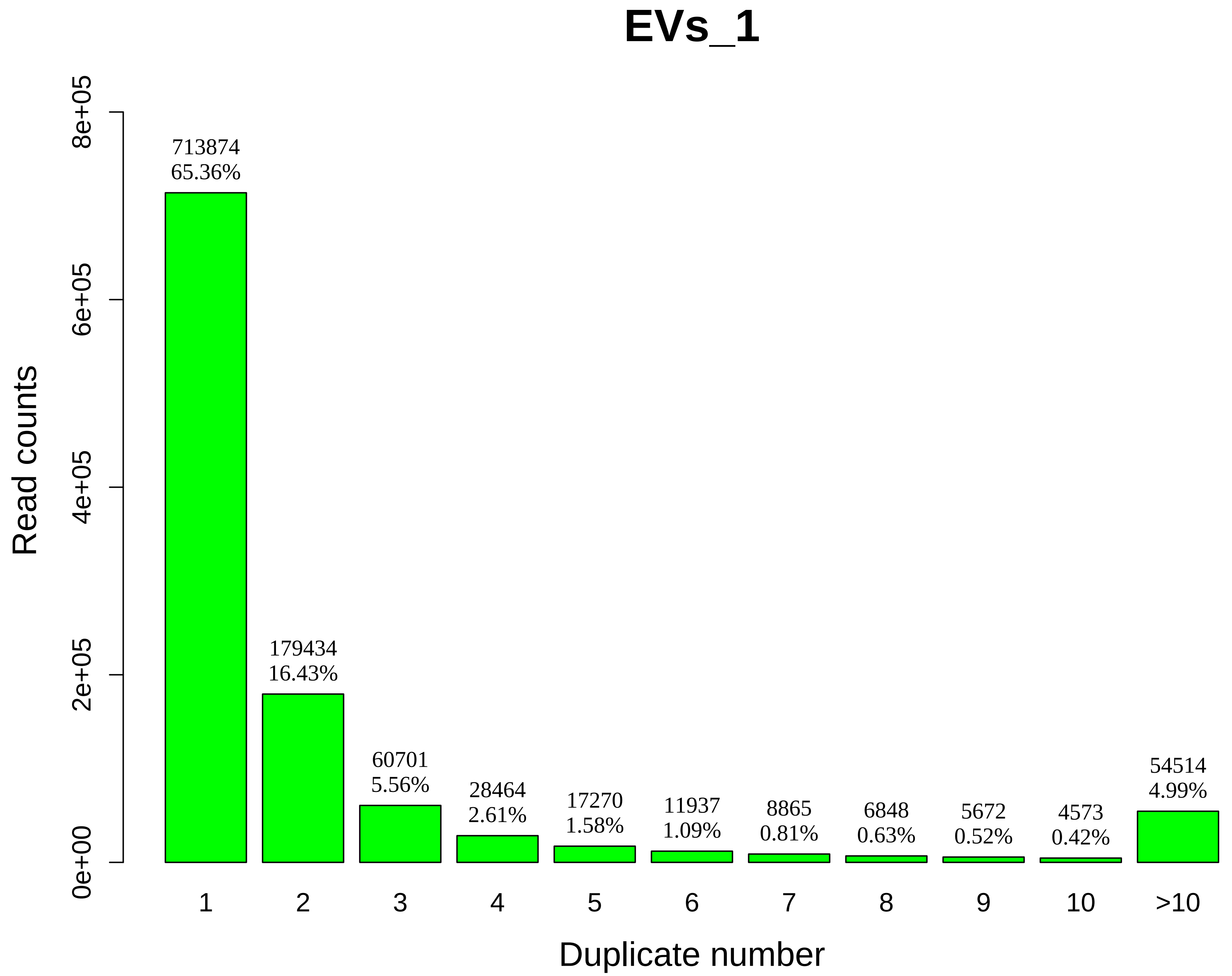
EVs_1
Read counts
8e+05
6e+05
4e+05
2e+05
0e+00
713874
65.36%
179434
16.43%
60701
5.56%
28464
2.61%
17270
1.58%
11937
1.09%
8865
0.81%
6848
0.63%
5672
0.52%
4573
0.42%
54514
4.99%
1
2
3
4
5
6
7
8
9
10
>10
Duplicate number
