# Supplementary material for: Adipose stem cells derived extracellular vesicles alleviate retinal excitotoxicity via miR-23a-5p/PLCD1/PKCA/GluA2 axis: a potential therapeutic strategy
Source: Stem Cell Res Ther. 2026 Jun 30;17:321. doi: 10.1186/s13287-026-05140-z (PMC13587503; doi:10.1186/s13287-026-05140-z)
Supplement: Supplementary file 1 [file 13287_2026_5140_MOESM1_ESM.zip › 13287_2026_5140_MOESM1_ESM/13287_2026_5140_MOESM7_ESM.zip!/The RNA-seq data/1.2.QCReport/EVs_2.duplication.distribution.pdf]

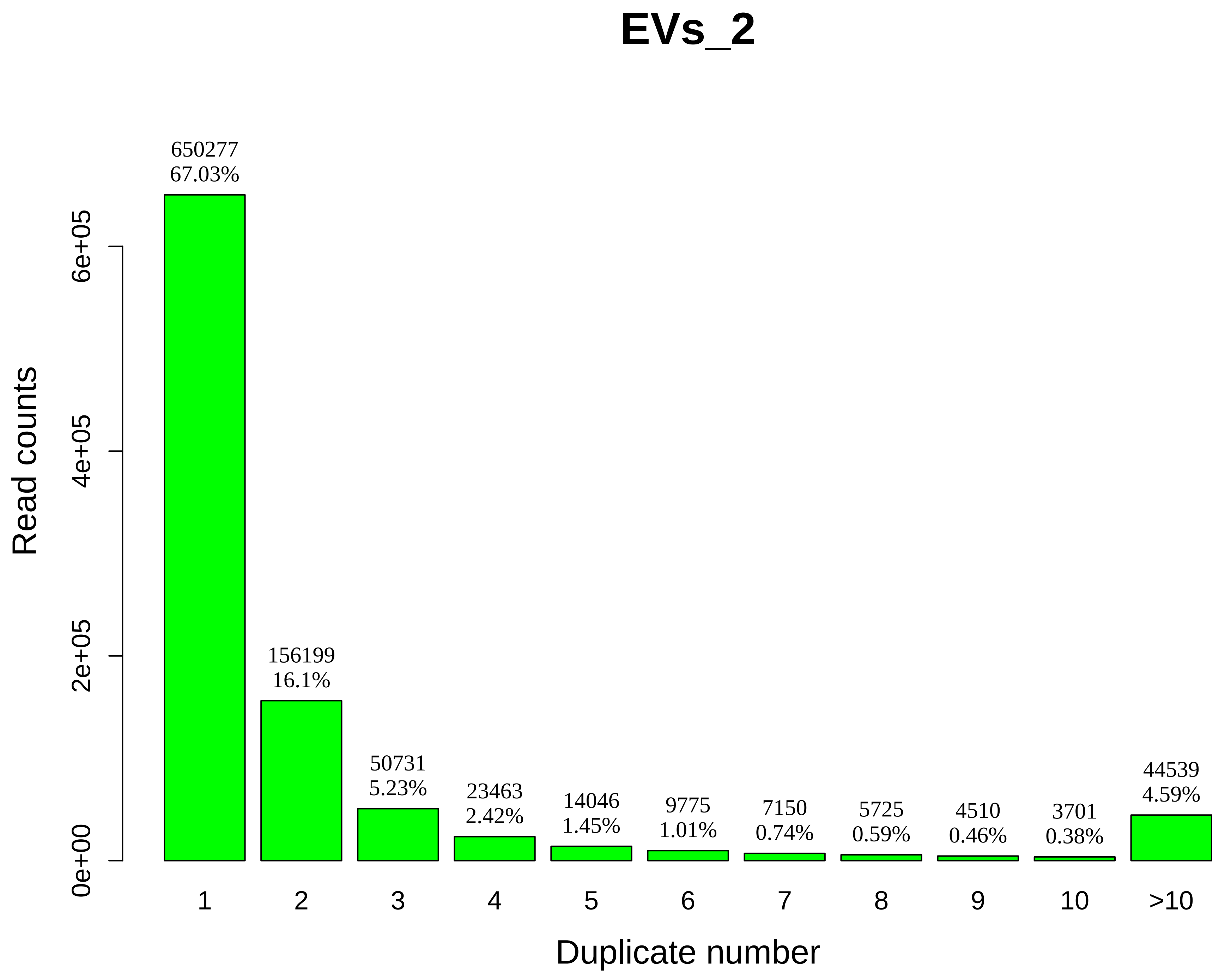
EVs_2
Read counts
0e+00
2e+05
4e+05
6e+05
650277
67.03%
156199
16.1%
50731
5.23%
23463
2.42%
14046
1.45%
9775
1.01%
7150
0.74%
5725
0.59%
4510
0.46%
3701
0.38%
44539
4.59%
1
2
3
4
5
6
7
8
9
10
>10
Duplicate number
