# Supplementary material for: Adipose stem cells derived extracellular vesicles alleviate retinal excitotoxicity via miR-23a-5p/PLCD1/PKCA/GluA2 axis: a potential therapeutic strategy
Source: Stem Cell Res Ther. 2026 Jun 30;17:321. doi: 10.1186/s13287-026-05140-z (PMC13587503; doi:10.1186/s13287-026-05140-z)
Supplement: Supplementary file 1 [file 13287_2026_5140_MOESM1_ESM.zip › 13287_2026_5140_MOESM1_ESM/13287_2026_5140_MOESM7_ESM.zip!/The RNA-seq data/1.2.QCReport/EVs_2.length.distribution.pdf]

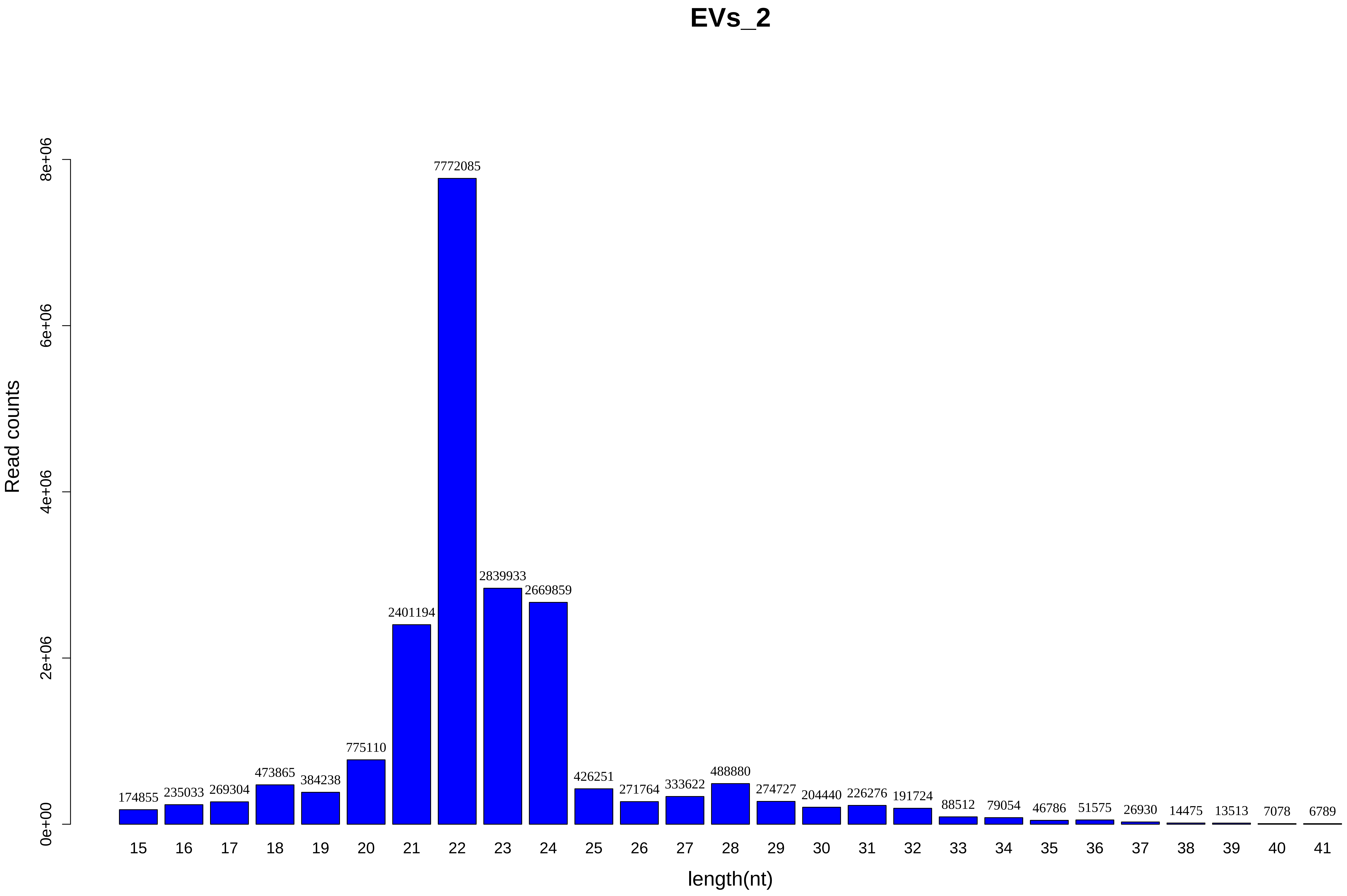
EVs_2
Read counts
length(nt)
0e+00
2e+06
4e+06
6e+06
8e+06
15
16
17
18
19
20
21
22
23
24
25
26
27
28
29
30
31
32
33
34
35
36
37
38
39
40
41
174855
235033
269304
473865
384238
775110
2401194
7772085
2839933
2669859
426251
271764
333622
488880
274727
204440
226276
191724
88512
79054
46786
51575
26930
14475
13513
7078
6789
