# Supplementary material for: Adipose stem cells derived extracellular vesicles alleviate retinal excitotoxicity via miR-23a-5p/PLCD1/PKCA/GluA2 axis: a potential therapeutic strategy
Source: Stem Cell Res Ther. 2026 Jun 30;17:321. doi: 10.1186/s13287-026-05140-z (PMC13587503; doi:10.1186/s13287-026-05140-z)
Supplement: Supplementary file 1 [file 13287_2026_5140_MOESM1_ESM.zip › 13287_2026_5140_MOESM1_ESM/13287_2026_5140_MOESM7_ESM.zip!/The RNA-seq data/1.2.QCReport/EVs_3.duplication.distribution.pdf]

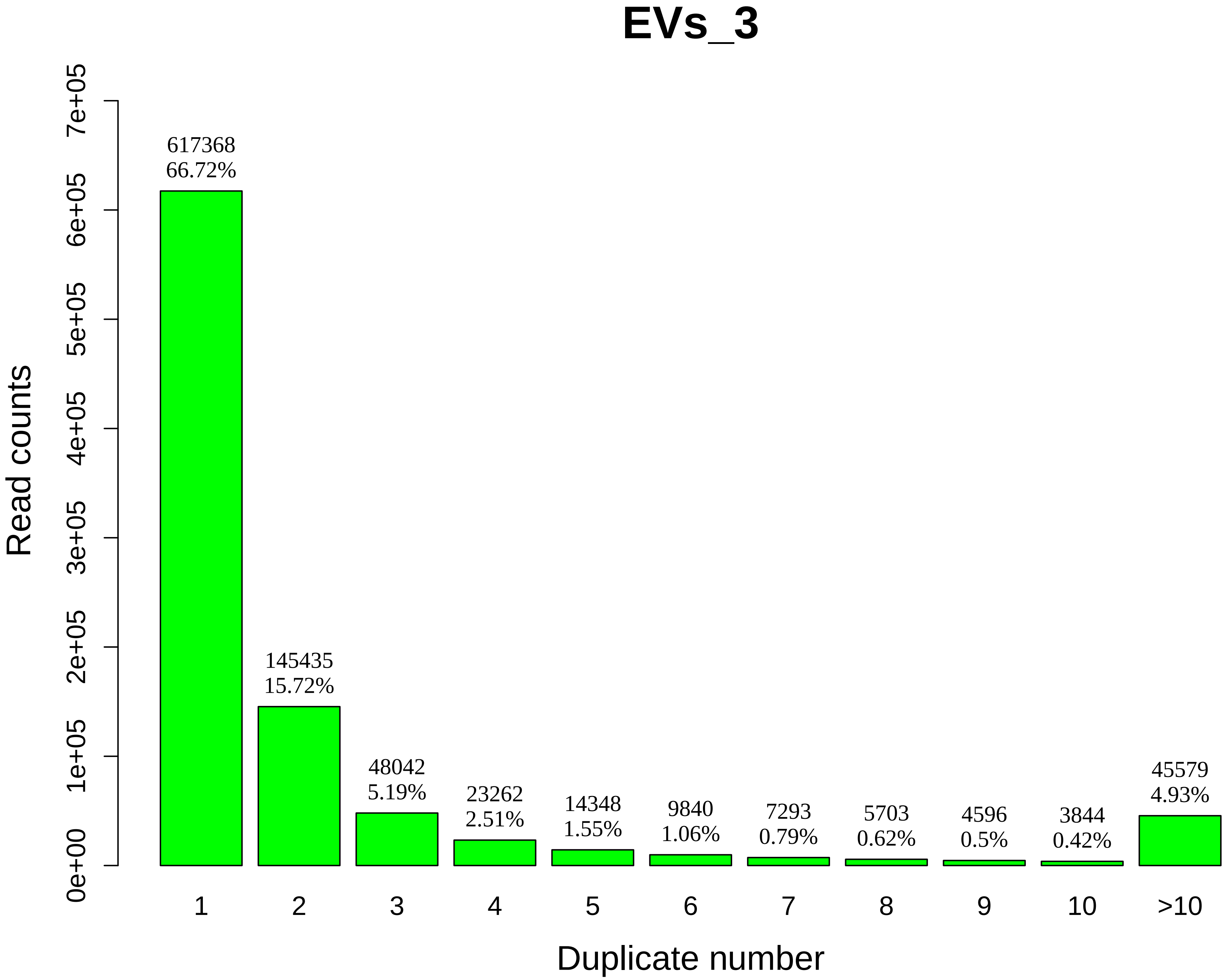
EVs_3
Read counts
0e+00
1e+05
2e+05
3e+05
4e+05
5e+05
6e+05
7e+05
617368
66.72%
145435
15.72%
48042
5.19%
23262
2.51%
14348
1.55%
9840
1.06%
7293
0.79%
5703
0.62%
4596
0.5%
3844
0.42%
45579
4.93%
1
2
3
4
5
6
7
8
9
10
>10
Duplicate number
