# Supplementary material for: Adipose stem cells derived extracellular vesicles alleviate retinal excitotoxicity via miR-23a-5p/PLCD1/PKCA/GluA2 axis: a potential therapeutic strategy
Source: Stem Cell Res Ther. 2026 Jun 30;17:321. doi: 10.1186/s13287-026-05140-z (PMC13587503; doi:10.1186/s13287-026-05140-z)
Supplement: Supplementary file 1 [file 13287_2026_5140_MOESM1_ESM.zip › 13287_2026_5140_MOESM1_ESM/13287_2026_5140_MOESM7_ESM.zip!/The RNA-seq data/1.2.QCReport/EVs_3.length.distribution.pdf]

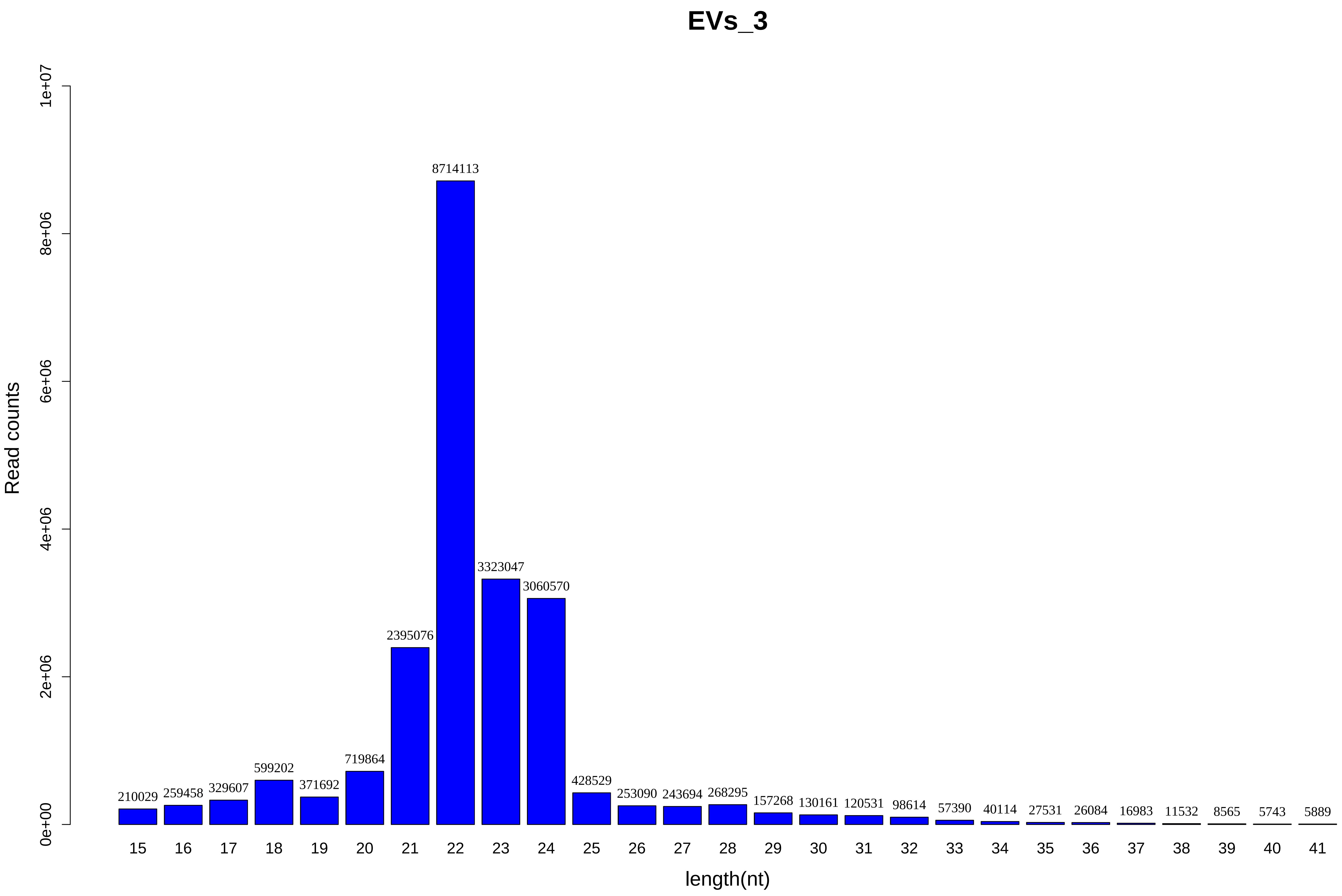

EVs_3
Read counts
0e+00
2e+06
4e+06
6e+06
8e+06
1e+07
210029
259458
329607
599202
371692
719864
2395076
8714113
3323047
3060570
428529
253090
243694
268295
157268
130161
120531
98614
57390
40114
27531
26084
16983
11532
8565
5743
5889
15
16
17
18
19
20
21
22
23
24
25
26
27
28
29
30
31
32
33
34
35
36
37
38
39
40
41
length(nt)
