# Supplementary material for: Adipose stem cells derived extracellular vesicles alleviate retinal excitotoxicity via miR-23a-5p/PLCD1/PKCA/GluA2 axis: a potential therapeutic strategy
Source: Stem Cell Res Ther. 2026 Jun 30;17:321. doi: 10.1186/s13287-026-05140-z (PMC13587503; doi:10.1186/s13287-026-05140-z)
Supplement: Supplementary file 1 [file 13287_2026_5140_MOESM1_ESM.zip › 13287_2026_5140_MOESM1_ESM/13287_2026_5140_MOESM7_ESM.zip!/The RNA-seq data/1.2.QCReport/Glu_1.duplication.distribution.pdf]

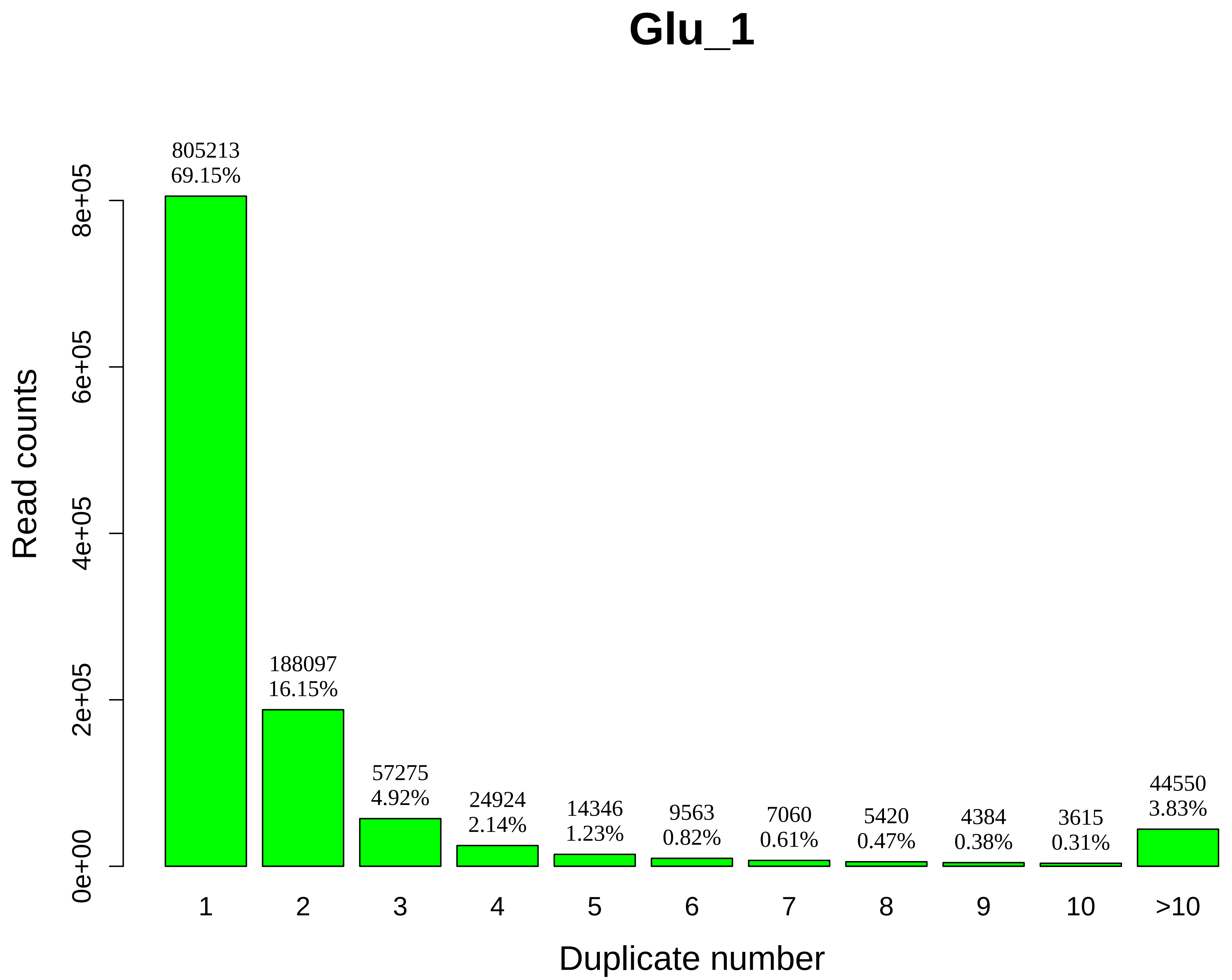

Glu_1
Read counts
0e+00
2e+05
4e+05
6e+05
8e+05
805213
69.15%
188097
16.15%
57275
4.92%
24924
2.14%
14346
1.23%
9563
0.82%
7060
0.61%
5420
0.47%
4384
0.38%
3615
0.31%
44550
3.83%
1
2
3
4
5
6
7
8
9
10
>10
Duplicate number
