# Supplementary material for: Adipose stem cells derived extracellular vesicles alleviate retinal excitotoxicity via miR-23a-5p/PLCD1/PKCA/GluA2 axis: a potential therapeutic strategy
Source: Stem Cell Res Ther. 2026 Jun 30;17:321. doi: 10.1186/s13287-026-05140-z (PMC13587503; doi:10.1186/s13287-026-05140-z)
Supplement: Supplementary file 1 [file 13287_2026_5140_MOESM1_ESM.zip › 13287_2026_5140_MOESM1_ESM/13287_2026_5140_MOESM7_ESM.zip!/The RNA-seq data/1.2.QCReport/Glu_1.length.distribution.pdf]

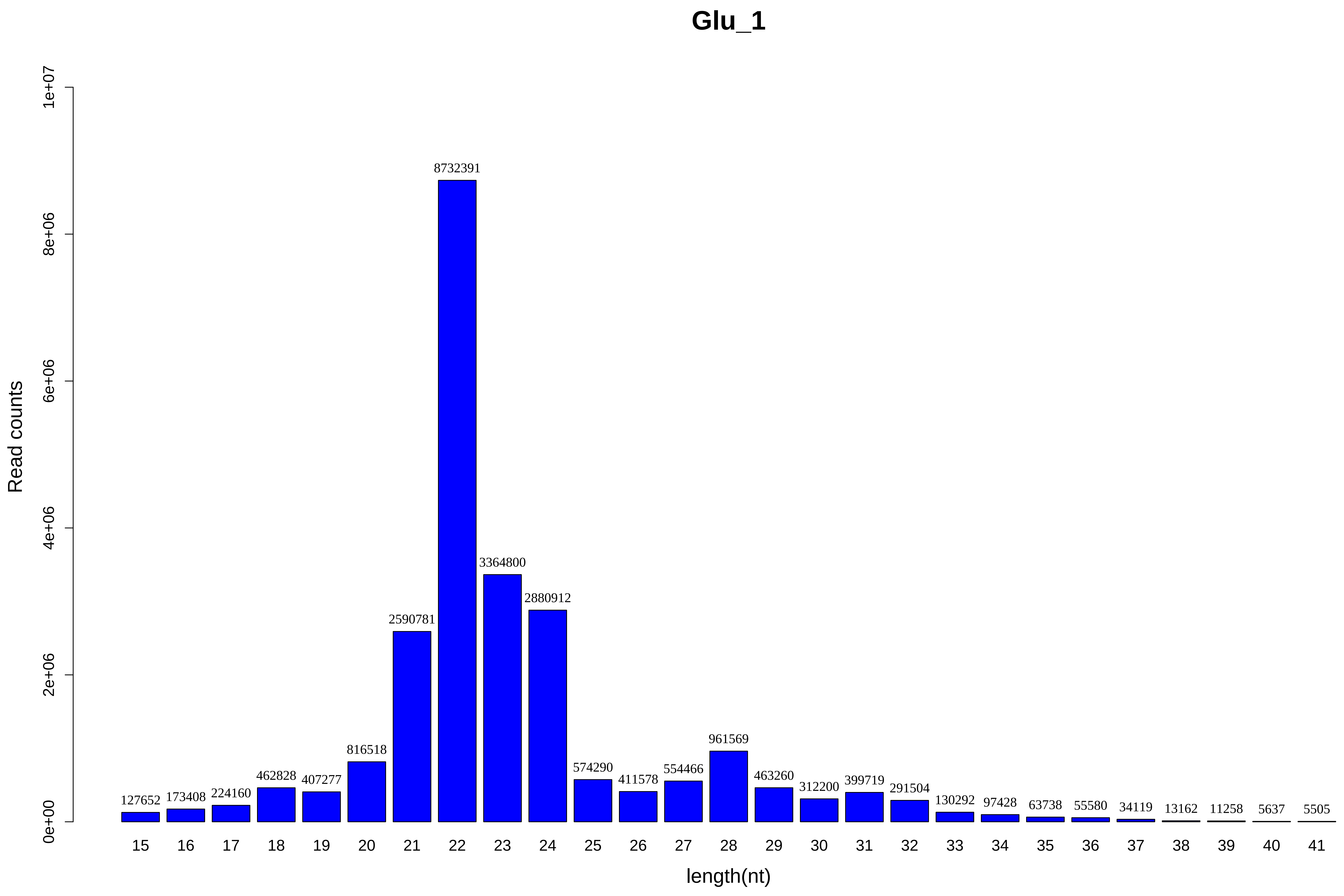
Glu_1
Read counts
0e+00
2e+06
4e+06
6e+06
8e+06
1e+07
127652
173408
224160
462828
407277
816518
2590781
8732391
3364800
2880912
574290
411578
554466
961569
463260
312200
399719
291504
130292
97428
63738
55580
34119
13162
11258
5637
5505
15
16
17
18
19
20
21
22
23
24
25
26
27
28
29
30
31
32
33
34
35
36
37
38
39
40
41
length(nt)
