# Supplementary material for: Adipose stem cells derived extracellular vesicles alleviate retinal excitotoxicity via miR-23a-5p/PLCD1/PKCA/GluA2 axis: a potential therapeutic strategy
Source: Stem Cell Res Ther. 2026 Jun 30;17:321. doi: 10.1186/s13287-026-05140-z (PMC13587503; doi:10.1186/s13287-026-05140-z)
Supplement: Supplementary file 1 [file 13287_2026_5140_MOESM1_ESM.zip › 13287_2026_5140_MOESM1_ESM/13287_2026_5140_MOESM7_ESM.zip!/The RNA-seq data/1.2.QCReport/Glu_2.duplication.distribution.pdf]

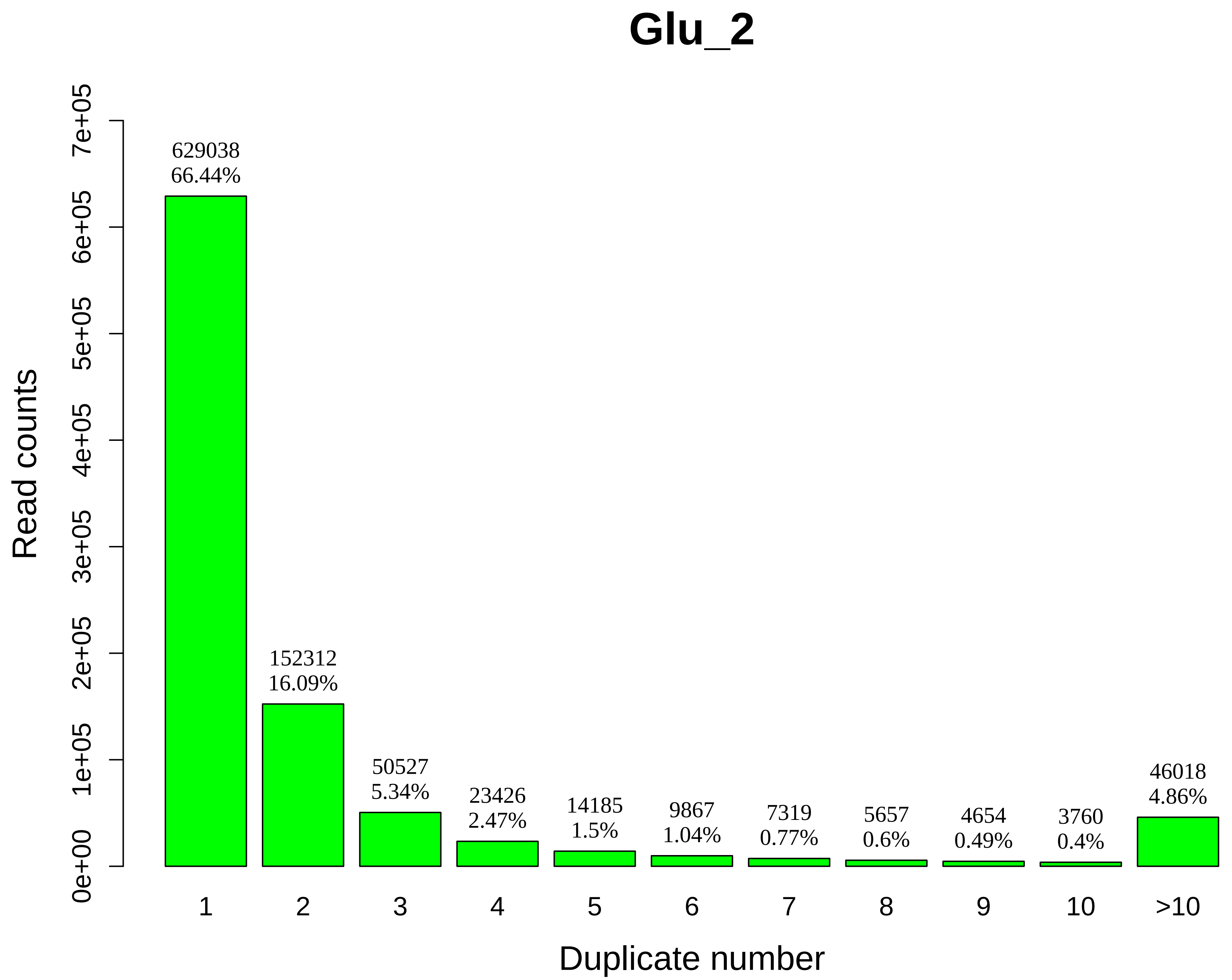

Glu_2
Read counts
Duplicate number
0e+00
1e+05
2e+05
3e+05
4e+05
5e+05
6e+05
7e+05
629038
66.44%
152312
16.09%
50527
5.34%
23426
2.47%
14185
1.5%
9867
1.04%
7319
0.77%
5657
0.6%
4654
0.49%
3760
0.4%
46018
4.86%
1
2
3
4
5
6
7
8
9
10
>10
