# Supplementary material for: Adipose stem cells derived extracellular vesicles alleviate retinal excitotoxicity via miR-23a-5p/PLCD1/PKCA/GluA2 axis: a potential therapeutic strategy
Source: Stem Cell Res Ther. 2026 Jun 30;17:321. doi: 10.1186/s13287-026-05140-z (PMC13587503; doi:10.1186/s13287-026-05140-z)
Supplement: Supplementary file 1 [file 13287_2026_5140_MOESM1_ESM.zip › 13287_2026_5140_MOESM1_ESM/13287_2026_5140_MOESM7_ESM.zip!/The RNA-seq data/1.2.QCReport/Glu_2.length.distribution.pdf]

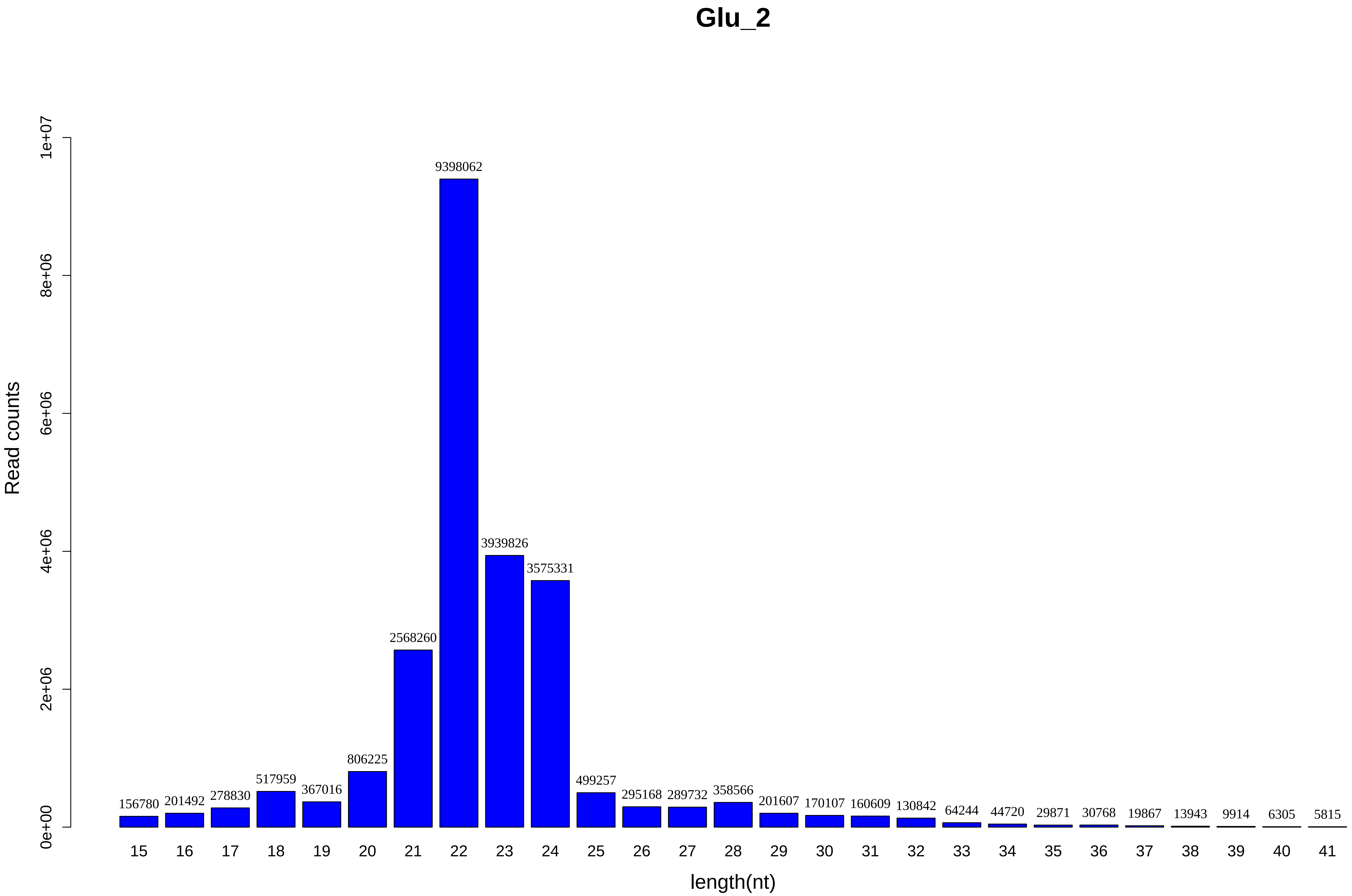
Glu_2
Read counts
0e+00
2e+06
4e+06
6e+06
8e+06
1e+07
156780
201492
278830
517959
367016
806225
2568260
9398062
3939826
3575331
499257
295168
289732
358566
201607
170107
160609
130842
64244
44720
29871
30768
19867
13943
9914
6305
5815
15
16
17
18
19
20
21
22
23
24
25
26
27
28
29
30
31
32
33
34
35
36
37
38
39
40
41
length(nt)
