# Supplementary material for: Adipose stem cells derived extracellular vesicles alleviate retinal excitotoxicity via miR-23a-5p/PLCD1/PKCA/GluA2 axis: a potential therapeutic strategy
Source: Stem Cell Res Ther. 2026 Jun 30;17:321. doi: 10.1186/s13287-026-05140-z (PMC13587503; doi:10.1186/s13287-026-05140-z)
Supplement: Supplementary file 1 [file 13287_2026_5140_MOESM1_ESM.zip › 13287_2026_5140_MOESM1_ESM/13287_2026_5140_MOESM7_ESM.zip!/The RNA-seq data/1.2.QCReport/Glu_3.duplication.distribution.pdf]

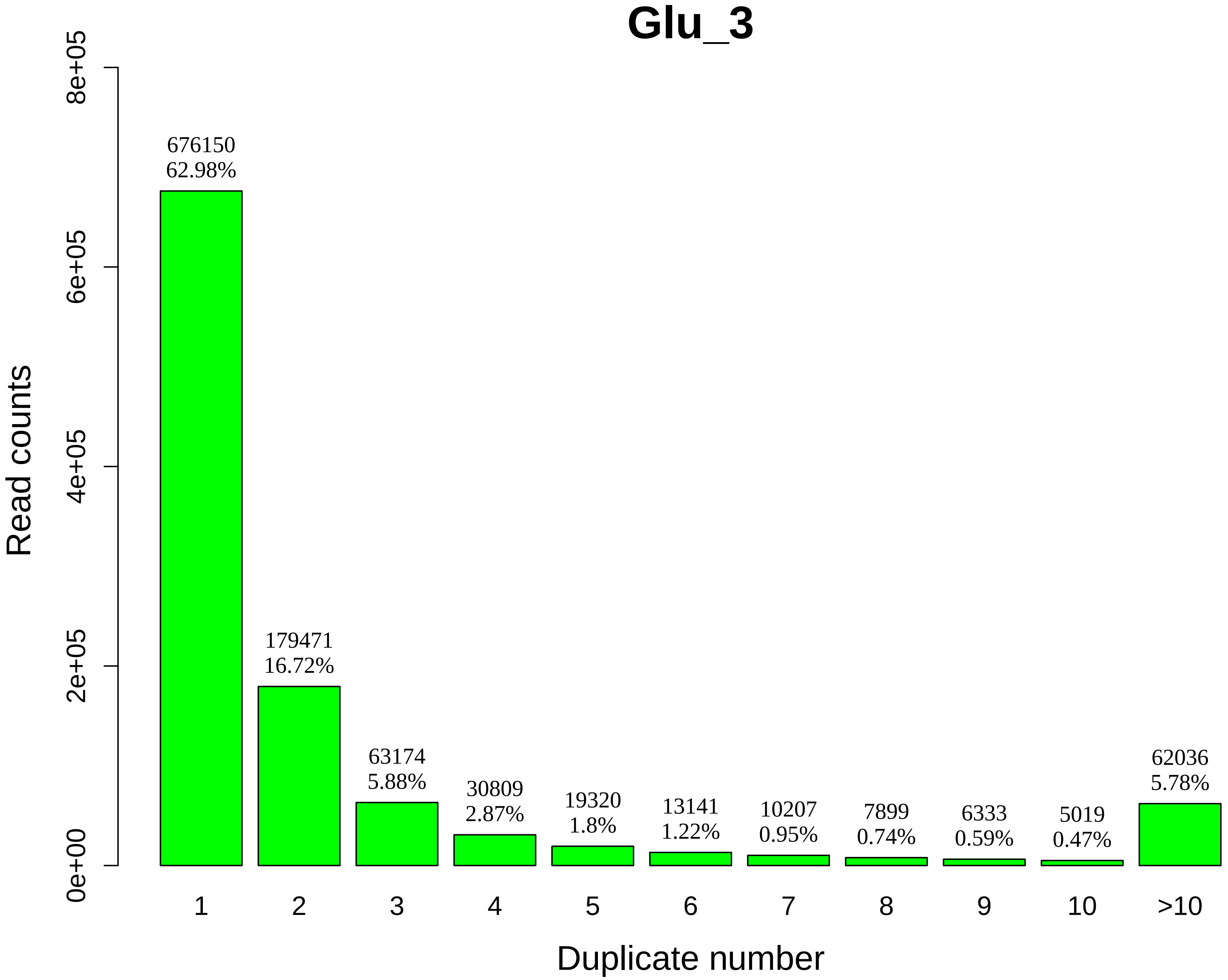
Glu_3
Read counts
Duplicate number
0e+00
2e+05
4e+05
6e+05
8e+05
676150
62.98%
179471
16.72%
63174
5.88%
30809
2.87%
19320
1.8%
13141
1.22%
10207
0.95%
7899
0.74%
6333
0.59%
5019
0.47%
62036
5.78%
1
2
3
4
5
6
7
8
9
10
>10
