# Supplementary material for: Adipose stem cells derived extracellular vesicles alleviate retinal excitotoxicity via miR-23a-5p/PLCD1/PKCA/GluA2 axis: a potential therapeutic strategy
Source: Stem Cell Res Ther. 2026 Jun 30;17:321. doi: 10.1186/s13287-026-05140-z (PMC13587503; doi:10.1186/s13287-026-05140-z)
Supplement: Supplementary file 1 [file 13287_2026_5140_MOESM1_ESM.zip › 13287_2026_5140_MOESM1_ESM/13287_2026_5140_MOESM7_ESM.zip!/The RNA-seq data/1.2.QCReport/Glu_3.length.distribution.pdf]

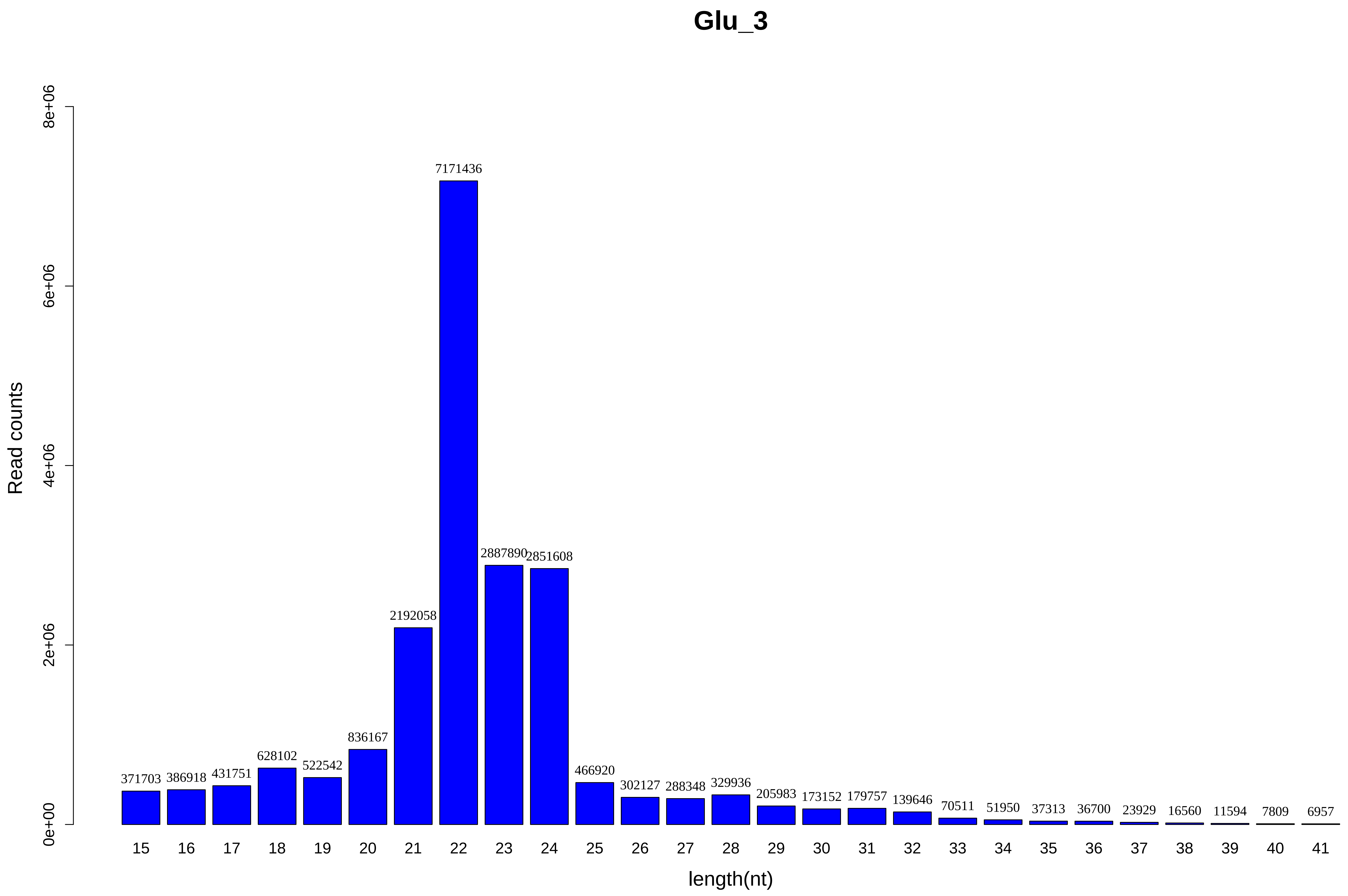

Glu_3
Read counts
0e+00
2e+06
4e+06
6e+06
8e+06
371703
386918
431751
628102
522542
836167
2192058
7171436
2887890
2851608
466920
302127
288348
329936
205983
173152
179757
139646
70511
51950
37313
36700
23929
16560
11594
7809
6957
15
16
17
18
19
20
21
22
23
24
25
26
27
28
29
30
31
32
33
34
35
36
37
38
39
40
41
length(nt)
