# Supplementary material for: Adipose stem cells derived extracellular vesicles alleviate retinal excitotoxicity via miR-23a-5p/PLCD1/PKCA/GluA2 axis: a potential therapeutic strategy
Source: Stem Cell Res Ther. 2026 Jun 30;17:321. doi: 10.1186/s13287-026-05140-z (PMC13587503; doi:10.1186/s13287-026-05140-z)
Supplement: Supplementary file 1 [file 13287_2026_5140_MOESM1_ESM.zip › 13287_2026_5140_MOESM1_ESM/13287_2026_5140_MOESM7_ESM.zip!/The RNA-seq data/2.4.Repbase_annotation/Glu_3_repeat_element_distribution_total.pdf]

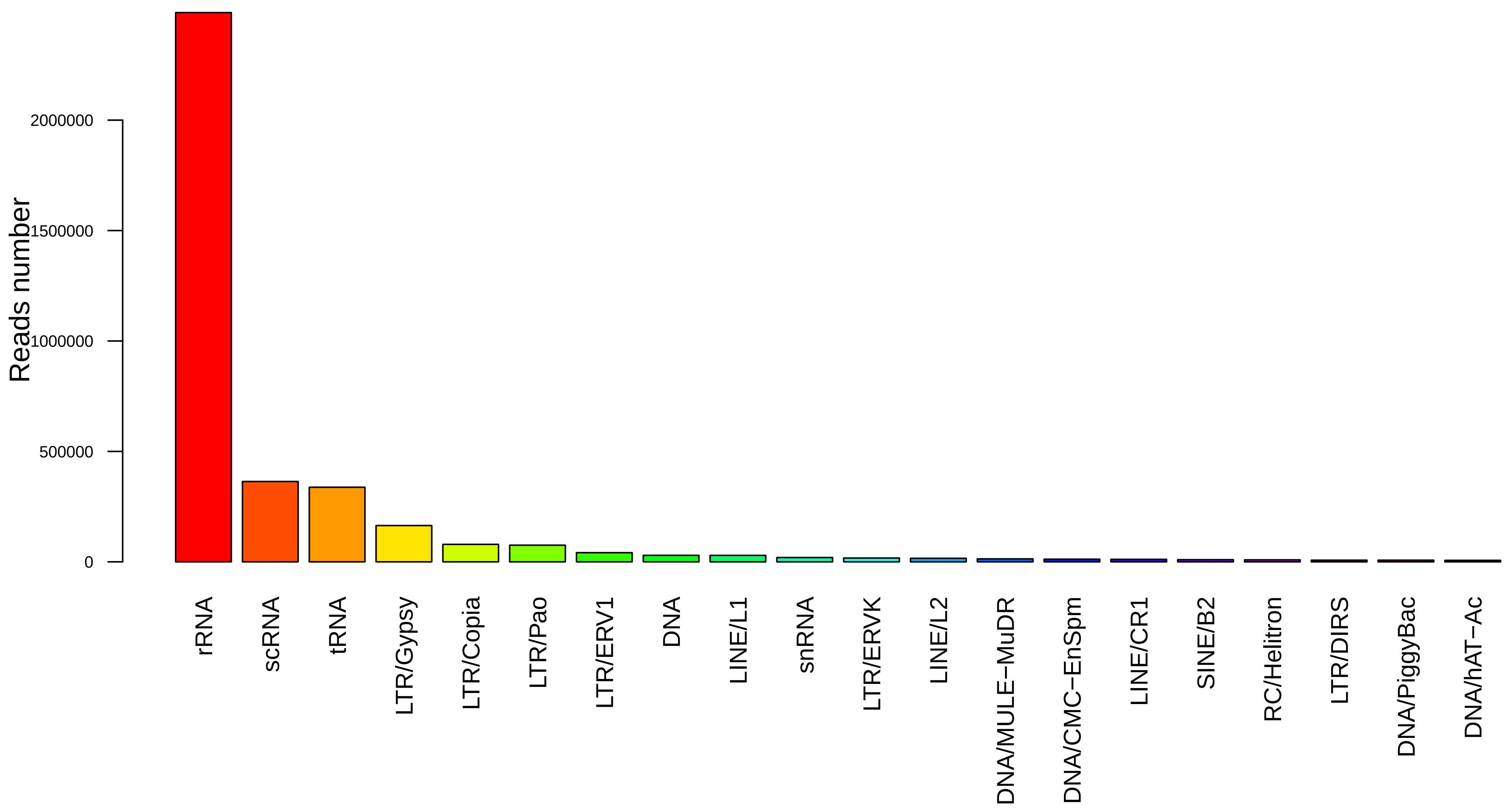
Reads number
2000000
1500000
1000000
500000
0
rRNA
scRNA
tRNA
LTR/Gypsy
LTR/Copia
LTR/Pao
LTR/ERV1
DNA
LINE/L1
snRNA
LTR/ERVK
LINE/L2
DNA/MULE−MuDR
DNA/CMC−EnSpm
LINE/CR1
SINE/B2
RC/Helitron
LTR/DIRS
DNA/PiggyBac
DNA/hAT−Ac
