# Supplementary material for: Adipose stem cells derived extracellular vesicles alleviate retinal excitotoxicity via miR-23a-5p/PLCD1/PKCA/GluA2 axis: a potential therapeutic strategy
Source: Stem Cell Res Ther. 2026 Jun 30;17:321. doi: 10.1186/s13287-026-05140-z (PMC13587503; doi:10.1186/s13287-026-05140-z)
Supplement: Supplementary file 1 [file 13287_2026_5140_MOESM1_ESM.zip › 13287_2026_5140_MOESM1_ESM/13287_2026_5140_MOESM7_ESM.zip!/The RNA-seq data/3.1.miRNA_expression/box-TPM.pdf]

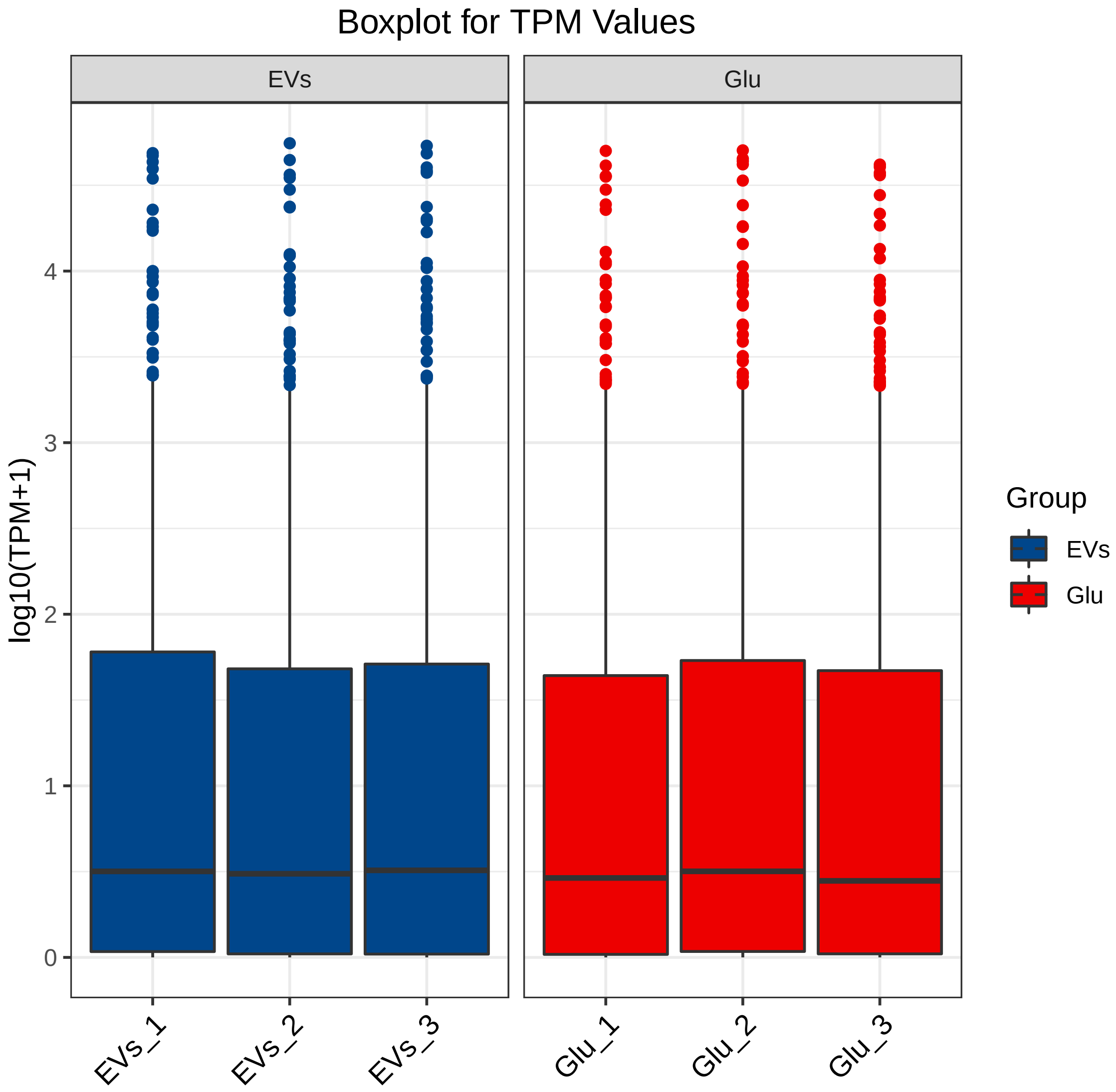

Boxplot for TPM Values
EVs
Glu
log10(TPM+1)
0
1
2
3
4
EVs_1
EVs_2
EVs_3
Glu_1
Glu_2
Glu_3
Group
EVs
Glu
