# Supplementary material for: Adipose stem cells derived extracellular vesicles alleviate retinal excitotoxicity via miR-23a-5p/PLCD1/PKCA/GluA2 axis: a potential therapeutic strategy
Source: Stem Cell Res Ther. 2026 Jun 30;17:321. doi: 10.1186/s13287-026-05140-z (PMC13587503; doi:10.1186/s13287-026-05140-z)
Supplement: Supplementary file 1 [file 13287_2026_5140_MOESM1_ESM.zip › 13287_2026_5140_MOESM1_ESM/13287_2026_5140_MOESM7_ESM.zip!/The RNA-seq data/3.1.miRNA_expression/heatmap-coefficient_matrix.pdf]

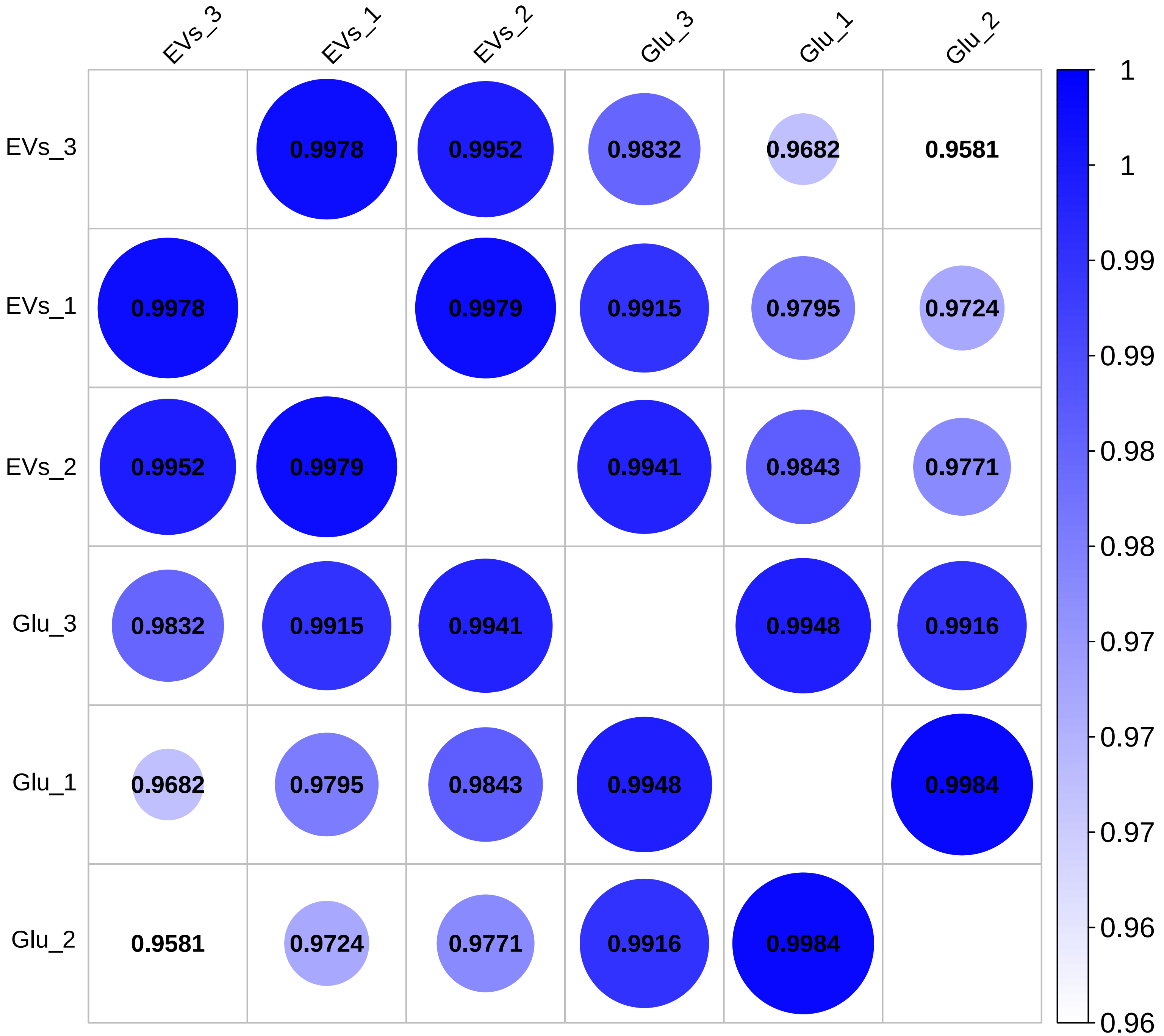
EVs_3
EVs_1
EVs_2
Glu_3
Glu_1
Glu_2
EVs_3
0.9978
0.9952
0.9832
0.9682
0.9581
EVs_1
0.9978
0.9979
0.9915
0.9795
0.9724
EVs_2
0.9952
0.9979
0.9941
0.9843
0.9771
Glu_3
0.9832
0.9915
0.9941
0.9948
0.9916
Glu_1
0.9682
0.9795
0.9843
0.9948
0.9984
Glu_2
0.9581
0.9724
0.9771
0.9916
0.9984
1
1
0.99
0.99
0.98
0.98
0.97
0.97
0.97
0.96
0.96
