# Supplementary material for: Adipose stem cells derived extracellular vesicles alleviate retinal excitotoxicity via miR-23a-5p/PLCD1/PKCA/GluA2 axis: a potential therapeutic strategy
Source: Stem Cell Res Ther. 2026 Jun 30;17:321. doi: 10.1186/s13287-026-05140-z (PMC13587503; doi:10.1186/s13287-026-05140-z)
Supplement: Supplementary file 1 [file 13287_2026_5140_MOESM1_ESM.zip › 13287_2026_5140_MOESM1_ESM/13287_2026_5140_MOESM7_ESM.zip!/The RNA-seq data/4.different_expressed_miRNA/Glu-vs-EVs/Glu-vs-EVs-heatmap-pval-0.05-FC-2.miRNA.pdf]

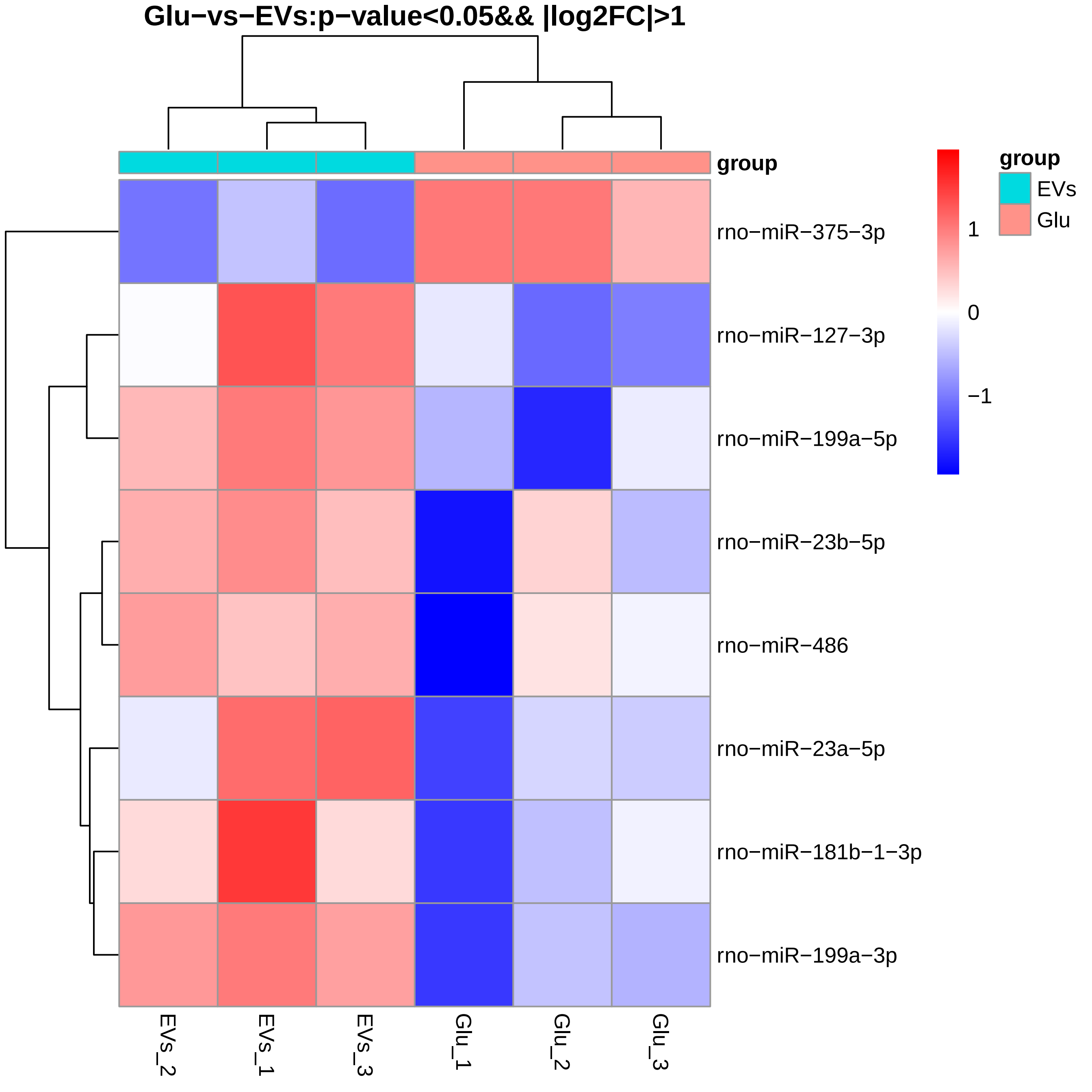
Glu−vs−EVs:p−value<0.05&& |log2FC|>1
group
rno−miR−375−3p
rno−miR−127−3p
rno−miR−199a−5p
rno−miR−23b−5p
rno−miR−486
rno−miR−23a−5p
rno−miR−181b−1−3p
rno−miR−199a−3p
EVs_2
EVs_1
EVs_3
Glu_1
Glu_2
Glu_3
1
0
−1
group
EVs
Glu
