# Supplementary material for: Adipose stem cells derived extracellular vesicles alleviate retinal excitotoxicity via miR-23a-5p/PLCD1/PKCA/GluA2 axis: a potential therapeutic strategy
Source: Stem Cell Res Ther. 2026 Jun 30;17:321. doi: 10.1186/s13287-026-05140-z (PMC13587503; doi:10.1186/s13287-026-05140-z)
Supplement: Supplementary file 1 [file 13287_2026_5140_MOESM1_ESM.zip › 13287_2026_5140_MOESM1_ESM/13287_2026_5140_MOESM7_ESM.zip!/The RNA-seq data/4.different_expressed_miRNA/Glu-vs-EVs/Glu-vs-EVs-volcano-pval-0.05-FC-2.miRNA.pdf]

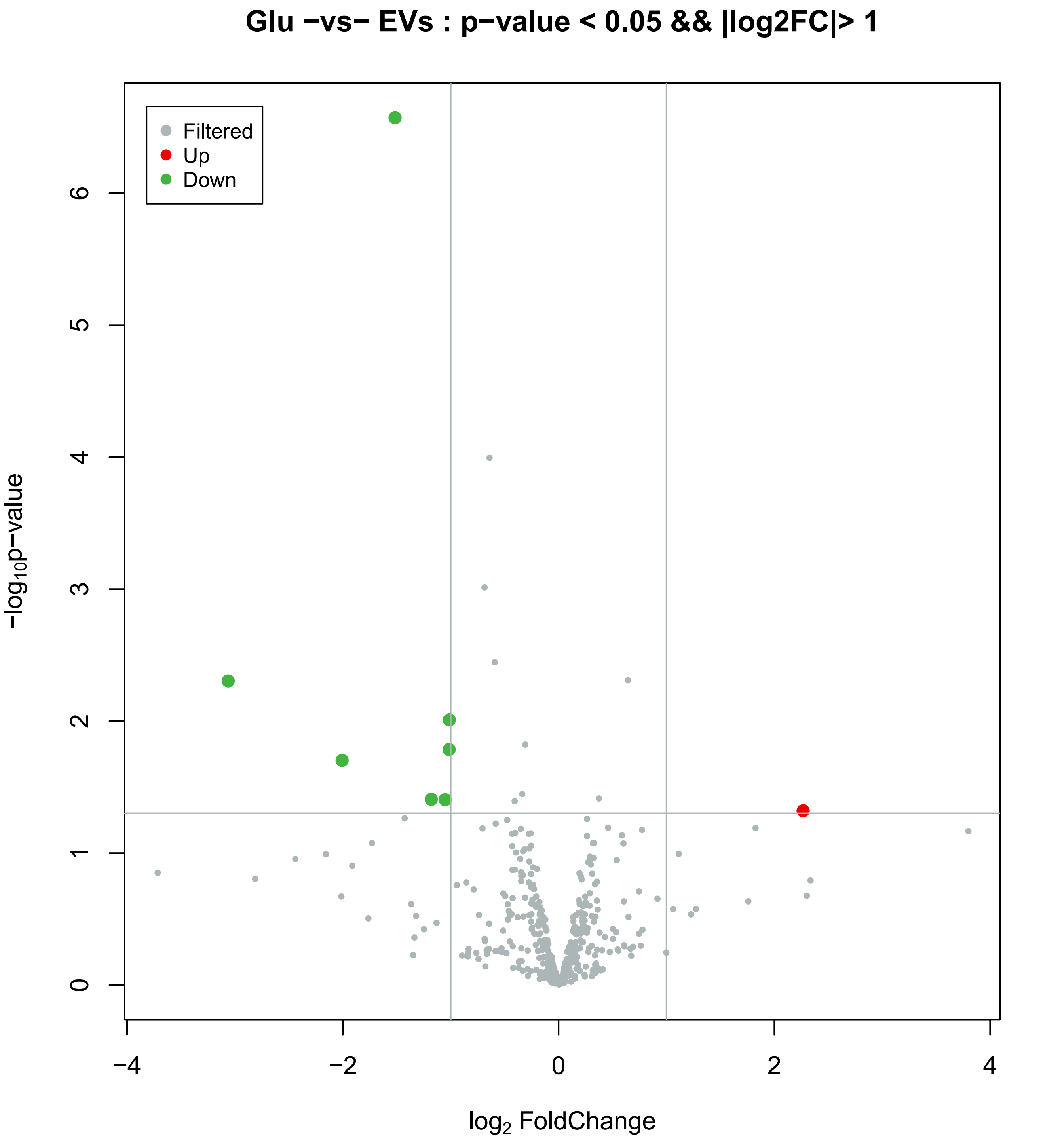
Glu −vs− EVs : p−value < 0.05 && |log2FC|> 1
Filtered
Up
Down
−log10p−value
0
1
2
3
4
5
6
−4
−2
0
2
4
log2 FoldChange
