# Supplementary material for: Adipose stem cells derived extracellular vesicles alleviate retinal excitotoxicity via miR-23a-5p/PLCD1/PKCA/GluA2 axis: a potential therapeutic strategy
Source: Stem Cell Res Ther. 2026 Jun 30;17:321. doi: 10.1186/s13287-026-05140-z (PMC13587503; doi:10.1186/s13287-026-05140-z)
Supplement: Supplementary file 1 [file 13287_2026_5140_MOESM1_ESM.zip › 13287_2026_5140_MOESM1_ESM/13287_2026_5140_MOESM7_ESM.zip!/The RNA-seq data/5.target_predict_analysis/23a-5p.doc]

＞rno-miR-23a-5p

GGGGTTCCTGGGGATGGGATTT
